# DNA methylation and gene expression profiles show novel regulatory pathways in hepatocellular carcinoma

**DOI:** 10.1186/s13148-015-0077-1

**Published:** 2015-04-14

**Authors:** Silvia Udali, Patrizia Guarini, Andrea Ruzzenente, Alberto Ferrarini, Alfredo Guglielmi, Valentina Lotto, Paola Tononi, Patrizia Pattini, Sara Moruzzi, Tommaso Campagnaro, Simone Conci, Oliviero Olivieri, Roberto Corrocher, Massimo Delledonne, Sang-Woon Choi, Simonetta Friso

**Affiliations:** Department of Medicine, University of Verona School of Medicine, Policlinico ‘G.B. Rossi’, P.le L.A. Scuro, 10, 37134 Verona, Italy; Department of Surgery, University of Verona School of Medicine, Policlinico ‘G.B. Rossi’, P.le L.A. Scuro, 10, 37134 Verona, Italy; Department of Biotechnology, Genetics and Heredity Section, University of Verona School of Agroindustrial Biotechnology, Ca’ Vignal 1, Strada Le Grazie 15, 37134 Verona, Italy; Friedman School of Nutrition Science and Policy Tufts University, 150 Harrison Ave, Boston, MA 02111 USA; Chaum Life Center, CHA University, 4-1, Cheongdam-dong, Gangnam-gu, 135-948 Seoul, Korea

**Keywords:** Alcohol, Candidate tumor-suppressor genes, DNA methylation, Epigenetics, Gene expression array, Hepatocellular carcinoma, MeDIP-chip, One-carbon metabolism, Retinol metabolism

## Abstract

**Background:**

Alcohol is a well-known risk factor for hepatocellular carcinoma (HCC), but the mechanisms underlying the alcohol-related hepatocarcinogenesis are still poorly understood. Alcohol alters the provision of methyl groups within the hepatic one-carbon metabolism, possibly inducing aberrant DNA methylation. Whether specific pathways are epigenetically regulated in alcohol-associated HCC is, however, unknown. The aim of the present study was to investigate the genome-wide promoter DNA methylation and gene expression profiles in non-viral, alcohol-associated HCC. From eight HCC patients undergoing curative surgery, array-based DNA methylation and gene expression data of all annotated genes were analyzed by comparing HCC tissue and homologous cancer-free liver tissue.

**Results:**

After merging the DNA methylation with gene expression data, we identified 159 hypermethylated-repressed, 30 hypomethylated-induced, 49 hypermethylated-induced, and 56 hypomethylated-repressed genes. Notably, promoter DNA methylation emerged as a novel regulatory mechanism for the transcriptional repression of genes controlling the retinol metabolism (*ADH1A, ADH1B, ADH6, CYP3A43, CYP4A22, RDH16*), iron homeostasis (*HAMP*)*,* one-carbon metabolism (*SHMT1*), and genes with a putative, newly identified function as tumor suppressors (*FAM107A, IGFALS, MT1G, MT1H, RNF180*).

**Conclusions:**

A genome-wide DNA methylation approach merged with array-based gene expression profiles allowed identifying a number of novel, epigenetically regulated candidate tumor-suppressor genes in alcohol-associated hepatocarcinogenesis. Retinol metabolism genes and *SHMT1* are also epigenetically regulated through promoter DNA methylation in alcohol-associated HCC.

Due to the reversibility of epigenetic mechanisms by environmental/nutritional factors, these findings may open up to novel interventional strategies for hepatocarcinogenesis prevention in HCC related to alcohol, a modifiable dietary component.

**Electronic supplementary material:**

The online version of this article (doi:10.1186/s13148-015-0077-1) contains supplementary material, which is available to authorized users.

## Background

Chronic alcohol consumption is one of the main etiological factors of hepatocellular carcinoma (HCC) [1]. The mechanisms by which ethanol promotes liver carcinogenesis are still not completely known, but alcohol is recognized interfering with several pathways including that of acetaldehyde, the first metabolic product of ethanol oxidation, that acts as a carcinogen [2]. Ethanol also affects one-carbon metabolism by altering the provision of methyl groups for biological methylation reactions, therefore proposing the alteration of methylation of DNA as a possible underlying mechanism for the alcohol-mediated carcinogenesis [3]. DNA methylation is catalyzed by DNA methyltransferases through the transfer of one-carbon units from S-adenosylmethionine (AdoMet) to the 5′ carbon of cytosines at CpG sequences in promoter and gene regulatory regions [4]. DNA methylation is the main epigenetic feature of DNA with a main function in gene transcriptional regulation as well as preservation of genome stability, and a wide variety of malignancies are characterized by aberrancies in DNA methylation [4,5]. Both a global DNA hypomethylation has been described as an almost universal finding in cancer [6,7], and a concurrent gene-specific hypermethylation has been observed at specific tumor-suppressor gene sites [7,8].

In a rodent model of HCC induced by methyl-deficient diet, DNA methylation was abnormally regulated [9]. Since alcohol exposure exerts effects that are similar to those induced by a methyl-deficient diet, it appears of interest to analyze the alcohol-induced epigenetic modifications, with the aim to shed light on alcohol-associated hepatocarcinogenesis. Alcohol is therefore both a major carcinogenic trigger and a factor altering one-carbon metabolism [10]. Considering the reversibility of epigenetic mechanisms by modifying nutritional factors such as alcohol intake [3], it is of particular interest to evaluate profiles of DNA methylation in alcohol-related HCC. Previous studies evaluating DNA methylation signature of HCC have mainly focused on HCC of viral etiology [11,12]. DNA methylation mechanisms could be more involved in hepatocarcinogenesis associated with alcohol, chronic consumption of which is known to significantly alter DNA methylation [3], relative to viral hepatocarcinogenesis in which genetic mechanisms have been extensively explored.

The aim of the present study was to define promoter DNA methylation and gene expression profiles in HCC tissues compared to homologous cancer-free liver tissues, by genome-wide, array-based approaches with the purpose of identifying possible novel epigenetically regulated pathways in alcohol-associated HCC.

## Results

### Clinical characteristics of HCC patients

The main clinical and biochemical characteristics of the patients are described in Table 1. Patients were males with an age ranging from 60 to 82 years. All of them were habitual drinkers for a period ≥20 years with a daily alcohol intake ≥3 units. The assessment of stage Child-Pugh score A, normal transaminases and GGT confirmed the absence of decompensate liver disease. Hematologic tests did not show abnormalities. IgA was also within the normal range. As expected, due to selection criteria, viral serologic tests for HBV and HCV were negative. Alphafetoprotein was higher than normal in all but one patient (Table 1).

**Table 1:** **Clinical and biochemical characteristics of HCC patients**

**Subject**	**Age (years)**	**Alcohol (units)** ^**a**^	**Smoking**	**Child-Pugh score**	**HBsAg**	**HCVAb**	**Hb (g/dL)**	**MCV (fL)**	**IgA (g/L)**	**GGT (U/L)**	**CHE (U/L)**	**AST (U/L)**	**ALT (U/L)**	**aFP (mg/L)**
1	66	>20	yes	A6	Neg	Neg	12.1	88.0	2.22	67	4,138	25	57	5,871
2	70	6	yes	A5	Neg	Neg	12.0	69.1	0.91	32	4,606	23	37	411
3	66	16	yes	A5	Neg	Neg	15.6	89.9	4.08	48	4,624	30	33	967
4	82	11	yes	A5	Neg	Neg	11.2	88.8	1.66	43	7,353	29	28	62
5	68	5	no	A5	Neg	Neg	16.0	98.1	3.98	52	7,856	25	29	190
6	60	6	yes	A5	Neg	Neg	13.8	98.6	1.60	223	3,564	38	51	5
7	75	4	yes	A5	Neg	Neg	13.7	90.3	3.28	30	7,225	28	15	431
8	71	10	yes	A5	Neg	Neg	13.3	91.4	1.56	88	8,430	24	29	21

^a^1 unit is defined as 12 g of ethanol = 125-ml wine or 330-ml beer or 40-ml spirit.

Abbreviations: HBV Ag, hepatitis B virus antigen ; HCVAb, hepatitis C virus antibody; Hb, hemoglobin; MCV, mean corpuscolar volume; IgA, immunoglobulin fraction A; GGT, gamma-glutamyl transpeptidase; CHE, cholinesterase; AST, aspartate transaminase; ALT, alanine transaminase; aFP, alphafetoprotein.

Alcohol drinking defined as ≥36 g ethanol/day for males and ≥24 g ethanol/day for females.

Reference values: Hb (g/dl) 13.5 to 16; MCV (fl) 86 to 98; IgA (g/L) 0.7 to 4.0; GGT (U/l) <50; CHE (U/l) 4,650 to 14,400; AST (U/l) 8 to 50; ALT (U/l) 8 to 45; aFP (mg/L) <7.

### Promoter methylation profiles in HCC *versus* cancer-free tissues

**Figure 1:**
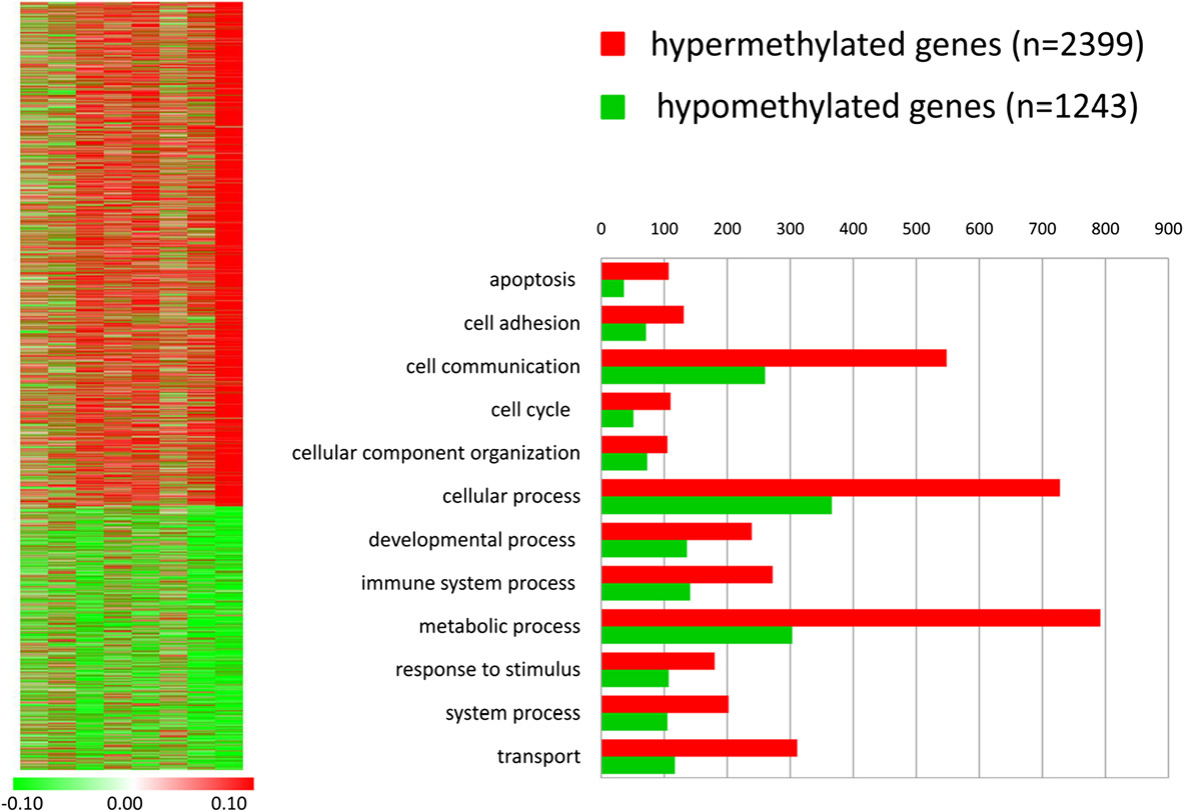
HeatMap of hypermethylated and hypomethylated genes by MeDIP-chip analysis. The figure shows the differences in DNA methylation values between HCC and cancer-free tissues. The chromatic scale (left side) represents values from −0.10 (green, hypomethylated in HCC) to +0.10 (red, hypermethylated in HCC). Bar chart (right side) representation of hypermethylated (red) and hypomethylated (green) genes clustered by PANTHER classification system according to biological processes.

The MeDIP-chip analysis showed 2,399 hypermethylated (Additional file 1: Table S1) and 1,243 hypomethylated gene promoters (Additional file 2: Table S2) in HCC compared to cancer-free tissues. The differentially methylated genes are represented by a HeatMap in Figure 1. The PANTHER classification system identified a large number of differentially methylated genes belonging to pathways involved in carcinogenesis as apoptosis, cell communication and adhesion, cell cycle regulation, and immune system (Figure 1) (see complete data in Additional file 1: Table S1 and in Additional file 2: Table S2).

### Gene expression in HCC *versus* cancer-free tissues

The array-based analysis of gene expression of HCC *versus* cancer-free tissue showed 1,004 downregulated and 668 upregulated genes (see complete set in Additional file 3: Table S3 and in Additional file 4: Table S4). Among the repressed genes, several pertain to retinol metabolism (*ADH1A, ADH1B, ADH6, CYP1A1, CYP1A2, CYP2B6, CYP2C9, CYP26A1, CYP3A4, CYP3A43, CYP4A11, CYP4A22, RDH16, RDH5, LRAT, ALDH1A3, ALDH8A1, BCO2*) and to one-carbon metabolism (*BHMT1, BHMT2, CBS, GNMT, MTHFD2L, CTH, SDS, SHMT1*).

### Promoter DNA methylation profile according to array-based gene expression in HCC *versus* cancer-free tissues

The promoter DNA methylation was merged with the array-based gene expression data in HCC *versus* cancer-free tissues. The analysis allowed distinguishing four groups of genes, according to the status of both promoter DNA methylation and gene expression. Such analysis highlighted 159 hypermethylated-repressed, 30 hypomethylated-induced, 49 hypermethylated-induced, and 56 hypomethylated-repressed genes.

### Hypermethylated-repressed genes

Table 2 shows the list of hypermethylated-repressed genes subdivided according to their biological function by means of PANTHER classification system. Twenty-six genes involved in cell cycle, growth, proliferation, and apoptosis. Five genes (*FAM107A, IGFALS, MT1G, MT1H, RNF180*) were highly methylated in the promoter region. Forty-three of those hypermethylated-repressed genes pertain to cellular processes regulation and six to retinol metabolism (*ADH1A, ADH1B, ADH6, CYP3A43, CYP4A22, RDH16*). A key gene of one-carbon metabolism, serine hydroxymethyltransferase 1 (*SHMT1*), involved in the methyl groups formation and transfer reactions also clustered in this group (Table 2). Further 23 genes involved in immune response clustered within this group among which hepcidin (*HAMP*), a molecule with hormone functions that controls the absorption of dietary iron and its distribution in different cells.

**Table 2:** **Hypermethylated and transcriptionally repressed genes (** ***n*** **= 159) in HCC as compared to cancer-free tissue**

**Cell communication (17)** ^**a**^								
Gene Name	*P* value	Coefficient	Gene Name	*P* value	Coefficient	Gene Name	*P* value	Coefficient
*AKAP2*	0.014	−1.5	*INHBC*	0.003	−1.4	*RND3*	0.002	−2.3
*AMHR2*	0.019	−1.4	*MORN4*	0.025	−1.2	*SH3D19*	0.004	−1.2
*BZRAP1*	0.039	−1.0	*OLFML3*	0.015	−2.3	*SORBS2*	0.013	−1.1
*C1orf168*	0.009	−1.5	*PDE2A*	0.011	−1.7	*SUCNR1*	0.006	−1.8
*CLDN1*	0.003	−1.0	*PPL*	0.01	−1.6	*VNN1*	0.031	−1.5
*FES*	0.00001	−1.5	*RIC3*	0.004	−1.8			
**Immune response (23)** ^**a**^								
Gene Name	*P* value	Coefficient	Gene Name	*P* value	Coefficient	Gene Name	*P* value	Coefficient
*ANTXR2*	0.012	−1.4	*CFI*	0.009	−1.0	*KLKB1*	0.007	−1.8
*BLNK*	0.002	−1.0	*CFP*	0.00002	−3.3	*LILRA1*	0.007	−1.2
*C1QTNF1*	0.035	−2.3	*FCGR2B*	0.047	−1.2	*MBL2*	0.046	−1.9
*C1RL*	0.016	−1.0	*FCN2*	0.00001	−3.7	*MEFV*	0.01	−1.4
*C5AR1*	0.037	−1.1	*HAMP*	0.01	−3.4	*PGLYRP2*	0.001	−1.9
*CCL14*	0.029	−1.5	*IL13RA2*	0.00001	−3.8	*TINAGL1*	0.043	−1.3
*CCL15*	0.029	−1.5	*IL1B*	0.005	−1.8	*VSIG4*	0.025	−2.4
*CD302*	0.003	−1.2	*IL1RN*	0.007	−1.1			
**Transport (19)** ^**a**^								
Gene Name	*P* value	Coefficient	Gene Name	*P* value	Coefficient	Gene Name	*P* value	Coefficient
*ANXA8*	0.043	−2.4	*SLC10A1*	0.019	−2.2	*SLC5A1*	0.012	−3.2
*APOA5*	0.004	−1.7	*SLC22A1*	0.002	−2.1	*SLC6A12*	0.007	−1.7
*APOL6*	0.005	−1.5	*SLC22A10*	0.009	−2.5	*SLCO1B3*	0.012	−3.7
*AQP7*	0.041	−1.0	*SLC25A25*	0.045	−1.3	*SLCO2B1*	0.001	−1.4
*CETP*	0.00001	−3.2	*SLC25A47*	0.011	−2.0	*TRPV4*	0.005	−2.1
*MIP*	0.006	−1.0	*SLC45A3*	0.012	−1.2			
*RGN*	0.009	−1.7	*SLC47A1*	0.033	−1.2			
**Metabolic and cellular process (43)** ^**a**^								
Gene Name	*P* value	Coefficient	Gene Name	*P* value	Coefficient	Gene Name	*P* value	Coefficient
*ACADS*	0.002	−1.3	*FBXO3*	0.004	−1.3	*MOGAT2*	0.00001	−3.2
*ACSM5*	0.026	−1.0	*FMO3*	0.019	−1.0	*OAT*	0.046	−2.0
*AGMO*	0.028	−1.3	*GLUD2*	0.004	−1.3	*PLIN*	0.012	−1.8
*AMDHD1*	0.048	−1.1	*GPT*	0.009	−1.0	*PSD4*	0.002	−1.2
*ANK2*	0.019	−1.0	*GYS2*	0.005	−3.0	*TBXA2R*	0.0004	−1.3
*ANK3*	0.026	−1.8	*HGFAC*	0.033	−3.1	*UROC1*	0.014	−2.5
*ARSD*	0.022	−1.4	*HK3*	0.003	−1.3	Retinol metabolism		
*ATP11C*	0.047	−1.0	*HOGA1*	0.002	−1.8	*ADH1A*	0.021	−1.4
*BCO2*	0.007	−3.1	*IDO2*	0.002	−3.7	*ADH1B*	0.021	−1.4
*CES4A*	0.023	−1.2	*INMT*	0.021	−1.2	*ADH6*	0.016	−1.9
*CHST9*	0.026	−1.5	*IYD*	0.003	−1.8	*CYP3A43*	0.027	−2.2
*CPN1*	0.014	−1.1	*KDM5D*	0.048	−1.5	*CYP4A22*	0.02	−1.1
*CYP8B1*	0.005	−2.7	*LCAT*	0.001	−1.8	*RDH16*	0.024	−1.4
*DSE*	0.022	−1.3	*LDHD*	0.018	−1.1	One-carbon metabolism		
*EPHX2*	0.025	−1.1	*LPAL2*	0.003	−1.4	*SHMT1*	0.003	−1.1
**Cell growth, cell cycle and apoptosis (26)** ^**a**^								
Gene Name	*P* value	Coefficient	Gene Name	*P* value	Coefficient	Gene Name	*P* value	Coefficient
*ADORA3*	0.014	−1.9	*GDF2*	0.00002	−2.7	*TBX15*	0.019	−1.7
*AGTR1*	0.006	−1.5	*JDP2*	0.007	−1.1	*TNFRSF10D*	0.025	−1.2
*AR*	0.038	−1.4	*MAP2K3*	0.007	−1.1	*ZBED1*	0.003	−1.1
*AXL*	0.049	−1.5	*NAP1L5*	0.004	−1.1	Candidate tumor-suppressor genes		
*CAT*	0.006	−1.1	*NR4A1*	0.043	−1.3	*FAM107A*	0.018	−1.3
*DBH*	0.002	−2.7	*NUGGC*	0.001	−1.8	*IGFALS*	0.00005	−3.2
*DMD*	0.043	−1.2	*PTH1R*	0.006	−2.5	*MT1G*	0.002	−2.8
*ESR1*	0.002	−3.0	*PTPN3*	0.001	−1.2	*MT1H*	0.004	−1.6
*FGD4*	0.01	−1.1	*SMAD6*	0.001	−1.3	*RNF180*	0.021	−1.2
**Miscellaneous (31)** ^**a**^								
Gene Name	*P* value	Coefficient	Gene Name	*P* value	Coefficient	Gene Name	*P* value	Coefficient
*ADAMTSL2*	0.04	−2.1	*FAM13A*	0.037	−1.6	*PID1*	0.019	−1.1
*ALPL*	0.003	−1.4	*FAM65C*	0.001	−1.4	*PRSS53*	0.026	−1.1
*ANKRD55*	0.001	−1.6	*FAM83F*	0.002	−1.4	*SMOC1*	0.015	−1.2
*C10orf26*	0.0001	−1.3	*FXYD7*	0.021	−1.1	*SPATA18*	0.005	−2.6
*C10orf58*	0.01	−1.2	*HAPLN4*	0.027	−1.6	*SYNE1*	0.021	−1.1
*C17orf91*	0.003	−1.2	*INS-IGF2*	0.0002	−4.2	*TCTEX1D1*	0.023	−1.3
*C21orf84*	0.04	−1.6	*LINC00574*	0.008	−1.1	*TMEM125*	0.039	−1.3
*CCDC68*	0.002	−1.6	*LOC339240*	0.014	−1.9	*TMEM26*	0.025	−1.2
*CILP*	0.0001	−2.1	*LRRC25*	0.039	−1.3	*UNC93A*	0.04	−1.2
*DNALI1*	0.003	−1.8	*MYO15A*	0.007	−1.2	*WDR66*	0.0001	−2.1
*EXPH5*	0.017	−2.3						

^a^Number of genes hypermethylated and transcriptionally repressed. Coefficient and *P* value refer to gene expression data.

### Hypomethylated-induced genes

Thirty genes belonged to this group (Table 3) among which: NADPH oxidase 4 (*NOX4*), linked to the production of various reactive oxygen species; the serine protease inhibitor, Kazal-type 1 (*SPINK1*), known as tumor-associated trypsin inhibitor (*TATI*) and endothelial cell-specific molecule 1 (*ESM1*), involved in angiogenesis.

**Table 3:** **Hypomethylated and transcriptionally induced genes (** ***n*** **= 30) in HCC as compared to cancer-free tissue**

**Cell communication (6)** ^**a**^		
Gene Name	*P* value	Coefficient
*ASAP1*	0.015	1.3
*CD34*	0.03	1.5
*GLDN*	0.006	1.2
*MYBPC1*	0.026	1.4
*RIMS2*	0.03	2.2
*TRIM55*	0.0002	2.8
**Immune response (8)** ^**a**^		
Gene Name	*P* value	Coefficient
*CD200*	0.038	2.1
*CTLA4*	0.018	2.2
*CXCL10*	0.048	1.6
*DCSTAMP*	0.03	1.1
*LRRC69*	0.015	1.0
*NOX4*	0.018	1.6
*SSX6*	0.03	3.2
*SSX8*	0.019	3.4
**Transport (2)** ^**a**^		
Gene Name	*P* value	Coefficient
*KIF4A*	0.006	2.2
*SLC7A11*	0.006	2.6
**Metabolic and cellular process (3)** ^**a**^		
Gene Name	*P* value	Coefficient
*DTNA*	0.044	2.0
*HIST1H4F*	0.022	1.7
*SPINK1*	0.0004	4.2
**Cell growth, cell cycle and apoptosis (4)** ^**a**^		
Gene Name	*P* value	Coefficient
*ESM1*	0.002	2.0
*GINS4*	0.013	1.4
*LTA*	0.009	1.0
*MAP2*	0.009	1.0
**Miscellaneous (7)** ^**a**^		
Gene Name	*P* value	Coefficient
*C15orf42*	0.002	1.9
*FBXO32*	0.003	2.1
*KIAA1688*	0.001	1.6
*POTEA*	0.009	1.7
*POTEC*	0.018	1.5
*VCX2*	0.017	2.0
*VCX3A*	0.012	2.4

^a^Number of genes hypermethylated and transcriptionally repressed. Coefficient and *P* value refer to gene expression data.

### Hypermethylated-induced and hypomethylated-repressed genes

Among the 49 hypermethylated-induced genes (Table 4) clustered, the matrix metallopeptidase 9 (*MMP9*) and 12 (*MMP12*) were involved in the breakdown of extracellular matrix, and nine genes implicated in cell growth and apoptosis. Among the 56 hypomethylated-repressed genes (Table 5), two candidate tumor-suppressor genes were identified, the hepatic and glial cell adhesion molecule (*HEPACAM*) and the ABI family, member 3 (NESH) binding protein (*ABI3BP*).

**Table 4:** **Hypermethylated and transcriptionally induced genes (** ***n*** **= 49) in HCC as compared to cancer-free tissue**

**Cell communication (7)** ^**a**^		
Gene Name	*P* value	Coefficient
*BAIAP2L2*	0.005	1.9
*EPS8L3*	0.006	3.1
*MCHR1*	0.019	1.4
*PMCH*	0.002	1.8
*RASL12*	0.039	1.4
*SEMA3G*	0.004	1.1
*TNNC1*	0.033	1.2
**Immune response (3)** ^**a**^		
Gene Name	*P* value	Coefficient
*MICB*	0.004	1.5
*SLAMF8*	0.015	1.6
*VWF*	0.041	1.0
**Transport (5)** ^**a**^		
Gene Name	*P* value	Coefficient
*KIF4A*	0.006	2.2
*KPNA2*	0.043	1.0
*SCN4A*	0.014	1.4
*SLC26A6*	0.00004	2.3
*TRIM16L*	0.041	1.6
**Metabolic and cellular process (15)** ^**a**^		
Gene Name	*P* value	Coefficient
*CELF6*	0.019	1.0
*COX7B2*	0.034	2.4
*DNMT3B*	0.026	1.1
*HIST1H4l*	0.035	1.0
*HKDC1*	0.012	2.5
*MMP12*	0.014	1.9
*MMP9*	0.039	1.8
*NEIL3*	0.012	2.0
*PDE4C*	0.017	1.1
*PIF1*	0.002	1.8
*PLA2G1B*	0.042	1.2
*RAB3B*	0.008	3.6
*S100P*	0.031	2.7
*UBE2T*	0.0001	2.8
*ZP3*	0.006	2.0
**Cell growth, cell cycle and apoptosis (9)** ^**a**^		
Gene Name	*P* value	Coefficient
*BAX*	0.031	1.1
*BOLA2*	0.002	1.6
*BOLA2B*	0.002	1.6
*KIAA0101*	0.024	1.9
*MAGEA5*	0.007	4.0
*PLK4*	0.011	1.3
*TRAF5*	0.018	1.2
*TRAIP*	0.016	1.9
*VRK1*	0.013	1.0
**Miscellaneous (10)** ^**a**^		
Gene Name	*P* value	Coefficient
*AIM1L*	0.003	2.0
*C16orf59*	0.027	1.4
*CSAG1*	0.032	2.7
*FAM189B*	0.022	1.0
*HRCT1*	0.001	2.2
*MND1*	0.001	2.5
*PLVAP*	0.009	1.6
*TRIM31*	0.00001	2.2
*VCY*	0.049	1.8
*ZWINT*	0.006	1.7

^a^Number of genes hypermethylated and transcriptionally repressed. Coefficient and *P* value refer to gene expression data.

**Table 5:** **Hypomethylated and transcriptionally repressed genes (** ***n*** **= 56) in HCC as compared to cancer-free tissue**

**Cell communication (8)** ^**a**^		
Gene Name	*P* value	Coefficient
*CRHBP*	0.001	−5.0
*DCN*	0.027	−2.8
*DLG2*	0.035	−1.2
*EMR1*	0.009	−1.5
*GPR128*	0.002	−2.9
*GRM8*	0.004	−2.0
*IGF1*	0.046	−1.9
*SPG20*	0.006	−1.1
**Immune response (9)** ^**a**^		
Gene Name	*P* value	Coefficient
*CLEC1B*	0.002	−2.6
*COLEC10*	0.00002	−3.5
*FCRL6*	0.034	−1.1
*FPR1*	0.025	−1.8
*IL1RL1*	0.046	−1.6
*LILRA5*	0.028	−1.5
*MARCO*	0.0006	−3.2
*NLRP12*	0.003	−1.1
*RAG1*	0.022	−1.2
**Transport (7)** ^**a**^		
Gene Name	*P* value	Coefficient
*AQP4*	0.043	−1.1
*EHD3*	0.008	−2.4
*LST-3TM12*	0.021	−2.3
*LYVE1*	0.0002	−3.7
*SLC38A4*	0.038	−1.9
*SLC6A19*	0.018	−1.2
*SYTL3*	0.035	−1.2
**Metabolic and cellular process (15)** ^**a**^		
Gene Name	*P* value	Coefficient
*DERA*	0.016	−1.1
*FBXL5*	0.012	−1.1
*FOLH1*	0.029	−1.5
*FRMD4B*	0.02	−1.2
*GALC*	0.016	−1.3
*GCNT2*	0.005	−1.3
*GLYATL1*	0.029	−1.3
*HEPACAM*	0.019	−3.2
*HSD11B1*	0.023	−1.1
*KLHL3*	0.007	−1.9
*NME5*	0.017	−1.3
*PBX1*	0.012	−1.1
*POU6F2*	0.012	−1.2
*RDH14*	0.021	−1.3
*TBXAS1*	0.031	−1.5
**Cell growth, cell cycle and apoptosis (6)** ^**a**^		
Gene Name	*P* value	Coefficient
*ABI3BP*	0.01	−1.4
*CNTN3*	0.008	−1.6
*MACF1*	0.003	−1.1
*PDE4DIP*	0.001	−1.5
*PTPN13*	0.021	−1.9
*TBRG1*	0.018	−1.5
**Miscellaneous (10)** ^**a**^		
Gene Name	*P* value	Coefficient
*C14orf105*	0.013	−1.3
*DOCK8*	0.042	−1.4
*ITLN1*	0.016	−1.7
*MBNL2*	0.021	−1.9
*NEBL*	0.045	−1.1
*PAMR1*	0.01	−2.1
*PLCXD3*	0.038	−1.7
*RNF217*	0.009	−1.1
*TMEM100*	0.013	−1.5
*TMEM133*	0.001	−1.2
*ZNF385B*	0.035	−1.8

^a^Number of genes hypermethylated and transcriptionally repressed. Coefficient and *P* value refer to gene expression data.

### Validation of array-based methylation and expression data

MeDIP-chip analysis was validated by direct bisulfite sequencing of three hypermethylated (*ESR1*, *RDH16,* and *SHMT1*) and one hypomethylated (*ESM1*) gene. The sequencing analysis thoroughly confirmed the methylation differences between HCC and cancer-free tissue achieved by the high-throughput technique (Additional file 5: Figure S1). The validation of array-based expression data by RealTime RT-PCR was performed on seven repressed genes (*ADH6, BCO2, ESR1, GDF2, HAMP, RDH16, SHMT1*) and four induced genes (*DNMT3B, ESM1, NOX4, SPINK1*), and all the outcomes fully supported the results obtained by the array (Additional file 6: Figure S2).

## Discussion

DNA methylation is the main epigenetic mechanism of gene expression regulation in humans, and alterations of this mechanism are regarded among the major molecular aberrations in malignancies [4,5]. The etiologic role of alcohol in hepatocarcinogenesis is well known [3], although the mechanisms underlying the link between alcohol and HCC are yet not completely defined [3]. Increasing evidence claims the importance of epigenetic features in alcohol-associated disorders including cancer [3] where appears crucial the effects of ethanol on one-carbon metabolism, ultimately leading toward an altered provision of methyl groups for methylation reactions [13] including those of DNA and histone proteins. Alcohol interferes with key enzymes in one-carbon metabolism and eventually causes impaired AdoMet levels [13] and inhibition of DNA methyltransferases by S-adenosylhomocysteine (AdoHcy) [14,15]. It is, therefore, likely to hypothesize that alcohol acts *via* aberrant DNA methylation for carcinogenesis [16]. Chronic alcohol consumption has been demonstrated to alter genomic DNA [15,17] and *p16* specific methylation in rodent models [15]. Furthermore, in cystathionine-beta-synthase deficiency mouse model exposed to high ethanol feeding, the altered methionine metabolism caused modifications both in DNA [18] and histone methylation profiles [19] while there is very little evidence for the effects of alcohol on epigenetic mechanisms in humans, thus far. In the present investigation, promoter DNA methylation and gene expression profiling were assessed at 22,532 promoter sites by genome-wide, array-based techniques in paired human HCC tissue compared to surrounding cancer-free liver tissue, after excluding the major known etiologic factors for HCC except a history of significant chronic alcohol intake. Previous studies evaluated the methylation signatures in HCC by a similar genome-wide approach and by evaluating tumor *versus* adjacent non-tumor tissues [11,12,20,21]. They differed for study design and enrolment criteria as for HCC etiologies [11,12,20,21] and for the purpose of either evaluating predictive HCC markers [11,20] or for the scope of identifying DNA methylation patterns specifically associated with disease progression [21].

All of the patients enrolled in this study were selected precisely to exclude HCC of viral etiology and for the absence of a severe liver derangement according to the Child-Pugh score.

By comparing the methylation profile of cancer *versus* homologous cancer-free liver tissues, 2,399 hypermethylated and 1,243 hypomethylated genes were identified in neoplastic tissue (Additional file 1: Table S1 and Additional file 2: Table S2). The differentially methylated genes, both hyper- and hypomethylated, represent an epigenetic peculiarity of alcohol-related HCC and may deserve future studies to identify new possible epigenetic markers of this specific type of cancer. In particular, the methylation status of specific genes has been proposed as a non-invasive tumor marker for HCC, by the analysis of circulating DNA derived from tumor cells [12,22]. In the field of DNA methylation, this technique represents a new and promising application that needs to be further explored. The aim of this study was to evaluate the role of promoter DNA methylation at gene transcriptional levels, then the promoter methylation patterns were merged with gene expression profiles allowing the identification of four groups of genes, being hyper- or hypomethylated and either repressed or induced in terms of gene expression. Only few studies evaluated the promoter methylation and gene expression profiling in HCC [23,24] and none yet in alcohol-related HCC. Among the cluster of hypermethylated-transcriptionally repressed genes (Table 2), five genes were identified as of potential interest for HCC, that is, *FAM107A, RNF180, MT1H, MT1G, IGFALS*. Although *FAM107A, RNF180,* and *MT1H* have been previously described for their implication in cancer affecting other tissues [25-27], the methylation-mediated repression of these genes was previously unknown for a possible association with HCC.

*FAM107A* was described in renal cell carcinoma as a putative tumor-suppressor gene according to its role in the regulation of apoptotic processes [25,28]. Reports on *RNF180* are scarce, though this gene was recently characterized as hypermethylated and silenced in gastric cancer [27] with a potential function in apoptosis [27]. As for *MT1H* and *MT1G*, it is known that metallothioneins (MTs) represent a class of proteins involved in processes of cellular detoxification from ROS and heavy metals and, through this mechanism, they might act as tumor-suppressor genes [29]. The methylation-mediated repression of *MT1G* has already been described in HCC [23] and hepatoblastoma in children [30]. The *MT1H*, another gene of the metallothionein family, from results of the present study is transcriptionally downregulated by promoter hypermethylation. Our results also confirm previous findings showing *IGFALS* as a possible tumor-suppressor gene silenced by methylation in HCC [23,24]. While it is not possible, from the present results, to define that the hypermethylated-repressed genes have a definite role as tumor suppressors, a hypothesis can be formulated in this regard, although it certainly needs further investigation. The largest number of hypermethylated-repressed genes was clustered in the category of metabolic and cellular processes (Table 2). Interestingly, the analysis showed the presence of six genes associated to retinol metabolism, that is*, ADH1A, ADH1B, ADH6, CYP3A43, CYP4A22,* and *RDH16*. Retinoid compounds, namely vitamin A and its derivatives, are known to be involved in the regulation of cellular growth, cellular differentiation, and apoptosis, and chronic ethanol intake was described to impair retinoic acid homeostasis in the development of alcohol-related cancers [31]. The present results suggest that the downregulation of those genes by DNA methylation may be among the mechanisms responsible for the derangement of retinol metabolism associated to chronic alcohol consumption. Interestingly, results from the present study show also a *SHMT1* gene repression by promoter hypermethylation. *SHMT1* is a key gene within one-carbon metabolism that operates as a metabolic switch between nucleotide synthesis and biological methylation pathways [32]. By depleting provision of 5-methyl-tetrahydrofolate for AdoMet synthesis, the SHMT1 gives higher metabolic priority to the thymidylate than AdoMet biosynthesis [32]. Thus, one can speculate that the *SHMT1* repression by DNA methylation depletes the AdoMet synthesis and eventually maintains a lower DNA methylation, a universal finding in cancer [7]. Other genes involved in one-carbon metabolism were found transcriptionally repressed in HCC tissue, although the methylation pattern was unchanged in *BHMT1, BHMT2, CBS, GNMT,* and *MTHFD2L* in cancer as compared to cancer-free tissue (Additional file 3: Table S3) or decreased in *FOLH1* (Table 5). All these genes exert their activity at different crucial nodes of the methyl unit transfer pathway for biological methylation [33]. Indeed, through this analysis, it is not possible to clarify whether the disregulation of one-carbon metabolism genes by promoter methylation is a cause or a result of hepatocarcinogenesis. Nevertheless, data are in accordance with the alcohol-induced alterations of methyl transfer reactions known to have a role in alcohol-related HCC [3] and, remarkably, show that such alterations are reflected in significant changes in promoter DNA methylation signature patterns in HCC tissue related to alcohol exposure. Among the hypermethylated-repressed genes, there was *HAMP*, the gene encoding for hepcidin, a liver peptide hormone involved in iron homeostasis and immune response [34]. *HAMP* has been shown to be transcriptionally repressed in HCC [35] and in the liver of alcoholics [36]. The transcriptional repression of *HAMP* by promoter DNA methylation in alcohol-related HCC is a novel, intriguing finding.

Several hypomethylated and transcriptionally activated genes were also detected in the present study. Of interest is the finding of overexpression of *NOX4*, *SPINK1*, and *ESM1* epigenetically regulated by promoter methylation. The increased expression of those genes has been previously observed in HCC for their implication in oxidative stress defense, regulation of tumor growth, and angiogenetic processes [37-39]. The observation of the hypermethylated-repressed and hypomethylated-upregulated genes confirmed the conventional notion for the role of DNA methylation at promoter for transcriptional complex regulation [40]. More uncertain is the significance of upregulation of hypermethylated genes and gene repression in hypomethylated genes, even though such phenomena were previously reported [7,8]. As it refers to hypermethylated and induced genes, it is well known that methylation of CpG sites blocks the binding of regulatory proteins, resulting in transcription modulation [41]. This mechanism may imply that hypermethylation in a silencer region could determine an increase in gene expression by preventing the binding of a putative repressor. In a study on a murine model, we observed a positive correlation between p16 promoter methylation and p16 expression in the old mouse colon [15,42]. The finding of hypomethylated and repressed genes is of more difficult interpretation, however one could hypothesize that, in certain genes, the regulation of transcription is independent from promoter methylation or that DNA methylation might affect transcription by different epigenetic mechanisms [40].

## Conclusions

In this study, a genome-wide DNA methylation approach merged with array-based gene expression profiles allowed identifying a number of novel, epigenetically regulated candidate tumor-suppressor genes in alcohol-associated hepatocarcinogenesis. Moreover, retinol metabolism genes and *SHMT1* resulted epigenetically regulated through promoter DNA methylation in alcohol-associated HCC.

Considering the reversibility of epigenetic mechanisms by nutritional factors [33,43], the interest of the present study lies precisely on the definition of promoter DNA methylation and gene expression profiles in HCC associated to alcohol, a modifiable dietary component. Nutrition interventional strategies may therefore be offered for hepatic carcinogenesis prevention through DNA methylation modulation.

## Availability of supporting data

The data sets supporting the results of this article are available in the NCBI’s Gene Expression Omnibus repository through GEO Series accession number GSE59261 (http://www.ncbi.nlm.nih.gov/geo/query/acc.cgi?acc=GSE59261).

## Methods

### Study patients and biochemical analyses

The study protocol conformed to the ethical guidelines of the 1975 Declaration of Helsinki and was approved by the Ethical Review Board of the University of Verona School of Medicine Hospital (Verona, Italy). Written informed consent was obtained from each patient after a detailed explanation of the study. The key eligibility criteria included an age ≥18 years, a diagnosis of histologically confirmed HCC in patients referring to the Divisions of Surgery and Internal Medicine. Surgical resectability criteria were a preserved liver function, Child-Pugh class A, the presence of a resectable single tumor or oligofocal resectable nodules (maximum three nodules), and the absence of extrahepatic metastases. The resectability assessment also included the tumor local stage, major vascular invasion, and the presence of affected lymphonodes. Exclusion criteria included coexisting hepatitis B (HBV) or C virus (HCV) infections as well as Epstein-Barr (EBV), cytomegalovirus (CMV), human immunodeficiency virus type 1 (HIV) positive serology; presence of relevant concurrent medical conditions such as chronic inflammatory diseases and hematological disorders, including autoimmune liver diseases and hereditary hemochromatosis, presence of acute inflammatory diseases, decompensate liver cirrhosis (Child-Pugh B, C). Patients under B vitamin supplementation and/or using drugs interfering with one-carbon metabolism in the 3 months before study enrolment were also excluded. For preoperative staging, chest-abdomen computerized tomography (CT) scan or nuclear magnetic resonance imaging (MRI) was used or positron emission tomography (PET-CT) or diagnostic laparoscopy in selected cases.

Venous blood samples were drawn from each subject in the free-living state, for routine laboratory tests including a complete blood count, serum concentrations of aspartate transaminase (AST), alanine transaminase (ALT), gamma-glutamyl transpeptidase (GGT), cholinesterase (CHE) and immunoglobulin (Ig) fractions including IgA, and alphafetoprotein. Serological tests for HBV, HCV, EBV, CMV, and HIV and tests to exclude an autoimmune etiology of the liver disease, that is, anti-smooth muscle, anti-nuclear, anti-mitochondrial, and anti-liver-kidney microsomal type 1 antibodies, were also performed. A chronic alcohol-drinking condition was defined as the intake of ≥36 g ethanol/day for the male sex, according to the guidelines of Italian INRAN (Istituto Nazionale di Ricerca per gli Alimenti e la Nutrizione, *National Institute for the Research on Foods and Nutrition*). HCC tissue and cancer-free liver tissues were excised during the surgical procedure and analyzed for histology by a pathologist unaware of the patient inclusion into the study. Among 33 patients enrolled for curative surgical resection for HCC, eight male patients were evaluated for epigenetic analyses based on availability of all biochemical data, adequate liver specimens with confirmed unequivocal HCC diagnosis and homologous cancer-free liver tissue, and a clear history of alcohol-drinking habit.

### DNA and RNA extraction

After surgical excision, tissue samples for nucleic acid extraction were immediately sliced into aliquots of about 100 mg, snap-frozen in liquid nitrogen, and stored at −80°C until use. Aliquots for DNA extraction were homogenized in 2 ml of chilled NaCl 0.9% w/v; cell lysis was achieved by using Igepal CA-630 (Sigma-Aldrich, St. Louis, MO, USA) 0.1% and lysis solution (NaCl 100 mM, EDTA 25 mM, SDS 1.6%, pH 8). Samples were treated with proteinase K/RNase, and DNA was extracted with a standard phenol/chloroform procedure.

For RNA extraction, each liver tissue aliquot (100 mg) was kept on ice, immediately homogenized in 2 ml of TRIReagent® (Sigma-Aldrich, St. Louis, MO, USA) and the homogenate stored at −80°C until use. RNA was extracted by guanidinium thiocyanate-phenol-chloroform-based method using TRIReagent® (Sigma-Aldrich, St. Louis, MO, USA) following manufacturer’s protocol, and the integrity was assessed by 2100 Bioanalyzer (Agilent, Santa Clara, CA, USA). RNA samples were used in array-based gene expression analysis only when the RNA Integrity Number was ≥7. Nucleic acid concentration and purity were assessed by NanoDrop 1000 spectrophotometer (Thermo Fisher Scientific, Wilmington, DE, USA).

### MeDIP-chip analysis

The methylated DNA immunoprecipitation (MeDIP) was performed with MeDIP kit™ (Diagenode, Liège, Belgium) [44,45] following the manufacturer’s protocol. After optimization of the fragmentation procedure: genomic DNA was sheared by nebulization using the GS Nebulizers Kit (Roche Applied Science, Basel, Switzerland) with argon pressure of 3.5 bar for 1 min, achieving uniform DNA fragments ranging from 300 to 1,000 bp in size, as confirmed by 2100 Bioanalyzer (Agilent, Santa Clara, CA, USA). A 1 μg of fragmented DNA was denatured (95°C for 7 min), then a one-fifth aliquot was drawn as untreated control (non-immunoprecipitated, INPUT); the remaining sample (immunoprecipitated, IP) was incubated overnight with anti-5methyl cytidine antibody. Immunoprecipitation enrichment was checked by RealTime PCR (7500 Real-Time PCR System, Applied Biosystems by Life Technologies, Carlsbad, CA, USA) with SYBR Green as fluorophore, both on internal and external controls supplied with the MeDIP kit™ (Diagenode, Liège, Belgium). Internal controls were human genomic regions either methylated (X-linked α satellites, AlphaX1) or unmethylated glyceraldehyde-3-phosphate dehydrogenase (*GAPDH*), while external controls were DNA specimens totally methylated or totally unmethylated that were added to the sample prior to the immunoprecipitation reaction. IP and INPUT samples were amplified by GenomePlex Complete Genome Amplification (WGA) kit (Sigma-Aldrich, St. Louis, MO, USA) following the producer’s protocol. A 1.5 μg of IP and 1.5 μg INPUT samples were labeled with Cy5 and Cy3 respectively by Dual-Color DNA Labeling Kit (NimbleGen-Roche, Madison, WI, USA) and co-hybridized on the Human DNA Methylation 3x720K CpG Island Plus RefSeq Promoter Array (NimbleGen-Roche). The microarray contained 22,532 promoters of RefSeq genes; each promoter region, ranging from −2.44 kb to +0.61 kb from the transcription start site, was covered by 50-75-mer probe 100 bp spacing (all coordinates are for genome build NCBI Hs36.3/HG18). The arrays were scanned at 2.5-μm resolution on a Axon GenePix 4400A scanner (Axon Instruments Inc., Union City, CA, USA), and the fluorescence intensity raw data were obtained by means of Nimblescan 2.5 extraction software (NimbleGen-Roche, Madison, WI, USA).

### Gene expression analysis by microarrays

The gene expression analysis was performed by means of Human Gene Expression 12x135K Arrays (Nimblegen-Roche, Madison, WI, USA) that analyzed 45,033 target genes with 60-mer probes (three probes/target), following the producer’s procedure. Briefly, 10 μg RNA was utilized to synthesize double-stranded cDNA by Superscript® Double-Stranded cDNA Synthesis Kit (Invitrogen, Carlsbad, CA, USA); 1 μg of cDNA was labeled by One-Color DNA Labeling Kit (NimbleGen-Roche), and 4 μg of Cy3-labeled cDNA was hybridized on the array. The slide was scanned using the Axon GenePix 4400A scanner, and scanned images (TIFF format) were then imported into NimbleScan 2.5 software for grid alignment and expression data analyses.

### Validation of array-based DNA methylation and gene expression data

DNA methylation data were validated on three hypermethylated, namely estrogen receptor 1 (*ESR1*), retinol dehydrogenase 16 (*RDH16*), serine hydroxymethyltransferase 1 (*SHMT1*), and one hypomethylated gene, that is, endothelial cell-specific molecule 1 (*ESM1*) by direct bisulfite sequencing. Bisulfite treatment was performed using the EpiTect® Bisulfite Kit (QIAGEN, Germantown, MD, USA). For each gene, the differentially methylated regions of interest (ROIs) were amplified by *ad hoc*-designed primers and optimized PCR conditions. The PCR-products were purified by GenElute^TM^ PCR Clean-UP kit (Sigma-Aldrich, St. Louis, MO, USA) and then sequenced by Capillary Electrophoretic Nucleic Acid Sequencer CEQ 8800 (Beckman Coulter, Brea, CA, USA). Gene expression results were validated on seven repressed and four induced genes: alcohol dehydrogenase 6 (*ADH6*), beta-carotene oxygenase 2 (*BCO2*), *ESR1*, growth differentiation factor 2 (*GDF2*), hepcidin antimicrobial peptide (*HAMP*), *RDH16, SHMT1* and DNA (cytosine-5-)-methyltransferase 3 beta (*DNMT3B*), *ESM1*, NADPH oxidase 4 (*NOX4*), serine peptidase inhibitor, and Kazal type 1 (*SPINK1*), respectively by RealTime RT-PCR using TaqMan assays. The 18S rRNA was used as the endogenous control [46].

### Computational and statistical methods

MeDIP-chip raw data were analyzed by Batman program [47], a cross-platform algorithm (https://github.com/dasmoth/batman) under the GNU Lesser General Public License that permits to calculate absolute methylation values. In the Batman analysis, the tissue samples, distinguished in HCC and cancer-free hepatic tissues, were considered as biological replicates. The promoter region of each gene was subdivided in 500 bp-long ROI, and an absolute methylation value was associated to each ROI. A ROI was considered differentially methylated when the difference of absolute methylation values between HCC and cancer-free tissue was ≥30% [48]. Expression data were normalized through quintile normalization and the Robust Multichip Average (RMA) algorithm [49] included in the NimbleScan software. Statistical analysis on gene expression array-based results was performed with Limma R package [50] considering a log2-fold change ≥1 or ≤ −1 and a *P* value adjusted for multiple testing (FDR) ≤0.05 as threshold to define differentially expressed genes. Protein ANalysis THrough Evolutionary Relationship (PANTHER) classification system was utilized to cluster genes of interest on the basis of their biological process involvement [51]. Bisulfite sequencing methylation data were obtained by calculating a methylation index for the CpG sites present in the ROI of each gene, as previously reported [52]. Gene expression data obtained by RealTime RT-PCR were analyzed by evaluating the difference in mRNA levels between cancer and cancer-free tissue. The calculation formula was the following: ΔΔCt = (Ct_target_-Ct_18s_)_HCC_ − (Ct_target_-Ct_18s_)_cancer-free._

## Additional files

Additional file 1: Table S1. Hypermethylated genes in HCC tissue: 2,399 genes found hypermethylated in HCC as compared to cancer-free tissue. Additional file 2: Table S2. Hypomethylated genes in HCC tissue: 1,243 genes found hypomethylated in HCC as compared to cancer-free tissue. Additional file 3: Table S3. Repressed genes in HCC tissue: 1,524 transcripts corresponding to 1,004 repressed genes in HCC as compared to cancer-free tissue (p.value.adj < 0.05). Additional file 4: Table S4. Induced genes in HCC tissue: 1,027 transcripts corresponding to 668 induced genes in HCC as compared to cancer-free tissue (p.value.adj < 0.05). Additional file 5: Figure S1. Validation of DNA methylation results: bisulfite Sanger sequencing of four differentially methylated genes in HCC and cancer-free liver tissue. Additional file 6: Figure S2. Validation of gene expression results: RealTime RT-PCR data of seven repressed and four induced genes in HCC tissue.

## Abbreviations

HCC: hepatocellular carcinoma
AdoMet: S-adenosylmethionine
HBV: hepatitis B virus
HCV: hepatitis C virus
EBV: Epstein-Barr virus
CMV: cytomegalovirus
HIV: human immunodeficiency virus type 1
CT: computerized tomography
MRI: nuclear magnetic resonance imaging
PET-CT: positron emission tomography
AST: aspartate transaminase
ALT: alanine transaminase
GGT: gamma-glutamyl transpeptidase
CHE: cholinesterase
Ig: immunoglobulin
INRAN (Istituto Nazionale di Ricerca per gli Alimenti e la Nutrizione: *National Institute for the Research on Foods and Nutrition*)
MeDIP: methylated DNA immunoprecipitation
INPUT: non-immunoprecipitated
IP: immunoprecipitated
ROI: region of interest
PANTHER: Protein ANalysis THrough Evolutionary Relationship
AdoHcy: S-adenosylhomocysteine
