# Supplementary material for: DNA methylation and gene expression profiles show novel regulatory pathways in hepatocellular carcinoma
Source: Clin Epigenetics. 2015 Apr 14;7(1):43. doi: 10.1186/s13148-015-0077-1 (PMC4419480; doi:10.1186/s13148-015-0077-1)
Supplement: Additional file 1: Table S1. — Hypermethylated genes in HCC tissue: 2,399 genes found hypermethylated in HCC as compared to cancer-free tissue. [file 13148_2015_77_MOESM1_ESM.doc]

| **Table S1** | |  |  |  |  |  |  |  |
| --- | --- | --- | --- | --- | --- | --- | --- | --- |
| **HYPERMETHYLATED GENES (n=2399) in HCC TISSUE** | | |  |  |  |  |  |  |
|  |  |  | **ROI coordinates** | | | **DNA methylation value** | | |
| **Gene symbol** | **RefSeq accession number** | **Chromosome** | **START** | **STOP** | **Transcription Start Site** | **HCC** | **cancer-free** | **difference** |
| *A1BG* | NM_130786 | chr19 | 63556738 | 63557220 | -302 | 0.77 | 0.44 | 0.33 |
| *A2M* | NM_000014 | chr12 | 9162181 | 9162284 | -2407 | 0.72 | 0.28 | 0.45 |
| *A3GALT2* | NM_001080438 | chr1 | 33560247 | 33560725 | -1200 | 0.76 | 0.42 | 0.34 |
| *AADACL3* | NM_001103169 | chr1 | 12697784 | 12698264 | -680 | 0.90 | 0.50 | 0.40 |
| *ABCA10* | NM_080282 | chr17 | 64754064 | 64754575 | -1768 | 0.81 | 0.41 | 0.41 |
| *ABCA5* | NM_018672 | chr17 | 64825318 | 64825798 | -2189 | 0.88 | 0.44 | 0.44 |
| *ABCA6* | NM_080284 | chr17 | 64651450 | 64651956 | -2093 | 0.76 | 0.35 | 0.41 |
| *ABCC2* | NM_000392 | chr10 | 101531145 | 101531537 | -1111 | 0.89 | 0.32 | 0.57 |
| *ABCG1* | NM_207629 | chr21 | 42508888 | 42509198 | -212 | 0.63 | 0.09 | 0.54 |
| *ABCG4* | NM_001142505 | chr11 | 118523586 | 118524070 | -1680 | 0.70 | 0.21 | 0.49 |
| *ABCG5* | NM_022436 | chr2 | 43921008 | 43921500 | -1792 | 0.65 | 0.20 | 0.46 |
| *ABHD11* | NM_001145363 | chr7 | 72792900 | 72793391 | -2019 | 0.78 | 0.43 | 0.35 |
| *ABL2* | NM_001100108 | chr1 | 177380895 | 177381299 | -2263 | 0.86 | 0.42 | 0.43 |
| *ABLIM1* | NM_001003407 | chr10 | 116435367 | 116435891 | -1225 | 0.88 | 0.56 | 0.33 |
| *ACADS* | NM_000017 | chr12 | 119647094 | 119647604 | -604 | 0.70 | 0.30 | 0.39 |
| *ACAP1* | NM_014716 | chr17 | 7179155 | 7179654 | -1166 | 0.81 | 0.32 | 0.48 |
| *ACCN3* | NM_004769 | chr7 | 150374649 | 150375167 | -1629 | 0.76 | 0.24 | 0.53 |
| *ACCN4* | NM_018674 | chr2 | 220085417 | 220085927 | -1623 | 0.79 | 0.33 | 0.46 |
| *ACE* | NM_152830 | chr17 | 58915439 | 58915923 | -227 | 0.74 | 0.35 | 0.39 |
| *ACOT7* | NM_181865 | chr1 | 6343050 | 6343536 | 58 | 0.51 | 0.09 | 0.42 |
| *ACSBG2* | NM_030924 | chr19 | 6086436 | 6086868 | -57 | 0.77 | 0.29 | 0.48 |
| *ACSF2* | NM_025149 | chr17 | 45857056 | 45857573 | -1202 | 0.82 | 0.34 | 0.48 |
| *ACSM1* | NM_052956 | chr16 | 20611074 | 20611491 | -1203 | 0.83 | 0.32 | 0.51 |
| *ACSM5* | NM_017888 | chr16 | 20328264 | 20328772 | 162 | 0.67 | 0.33 | 0.34 |
| *ACTG2* | NM_001615 | chr2 | 73972119 | 73972434 | -1323 | 0.74 | 0.38 | 0.36 |
| *ACTL7A* | NM_006687 | chr9 | 110664057 | 110664557 | -116 | 0.86 | 0.48 | 0.39 |
| *ACTRT2* | NM_080431 | chr1 | 2927024 | 2927522 | -632 | 0.81 | 0.34 | 0.46 |
| *ACY1* | NM_000666 | chr3 | 51990177 | 51990628 | -2199 | 0.60 | 0.26 | 0.35 |
| *ACYP1* | NM_001107 | chr14 | 74601878 | 74602402 | -1651 | 0.68 | 0.21 | 0.48 |
| *ADAM28* | NM_014265 | chr8 | 24206337 | 24206812 | -949 | 0.61 | 0.28 | 0.34 |
| *ADAM32* | NM_145004 | chr8 | 39081771 | 39082246 | -2197 | 0.90 | 0.47 | 0.43 |
| *ADAM3A* | NR_001569 | chr8 | 39500622 | 39501109 | -1238 | 0.77 | 0.38 | 0.39 |
| *ADAMTSL2* | NM_014694 | chr9 | 135387586 | 135388089 | -1957 | 0.59 | 0.28 | 0.30 |
| *ADAMTSL5* | NM_213604 | chr19 | 1465945 | 1466451 | -2010 | 0.81 | 0.50 | 0.31 |
| *ADH1A* | NM_000667 | chr4 | 100433053 | 100433529 | -2126 | 0.41 | 0.12 | 0.30 |
| *ADH1B* | NM_000668 | chr4 | 100461557 | 100462054 | -210 | 0.74 | 0.43 | 0.31 |
| *ADH6* | NM_000672 | chr4 | 100359866 | 100360399 | -706 | 0.89 | 0.58 | 0.30 |
| *ADORA2A* | NM_000675 | chr22 | 23151742 | 23152256 | -1530 | 0.74 | 0.23 | 0.51 |
| *ADORA3* | NM_000677 | chr1 | 111848115 | 111848629 | -106 | 0.62 | 0.21 | 0.41 |
| *ADSSL1* | NM_152328 | chr14 | 104259789 | 104260287 | -1540 | 0.60 | 0.30 | 0.30 |
| *AFF3* | NM_002285 | chr2 | 100125929 | 100126433 | -712 | 0.59 | 0.28 | 0.31 |
| *AGAP2* | NM_014770 | chr12 | 56423127 | 56423420 | -1066 | 0.93 | 0.62 | 0.31 |
| *AGGF1* | NM_018046 | chr5 | 76360052 | 76360525 | -1698 | 0.83 | 0.40 | 0.42 |
| *AGPAT3* | NM_001037553 | chr21 | 44167824 | 44168322 | -1633 | 0.75 | 0.38 | 0.37 |
| *AGR3* | NM_176813 | chr7 | 16889367 | 16889899 | -1495 | 0.88 | 0.46 | 0.42 |
| *AGTR1* | NM_032049 | chr3 | 149929299 | 149929778 | -1117 | 0.78 | 0.29 | 0.49 |
| *AGXT* | NM_000030 | chr2 | 241454415 | 241454958 | -2147 | 0.72 | 0.37 | 0.35 |
| *AHCYL1* | NM_006621 | chr1 | 110326944 | 110327159 | -1778 | 0.89 | 0.54 | 0.35 |
| *AHNAK2* | NM_138420 | chr14 | 104516189 | 104516703 | -707 | 0.67 | 0.24 | 0.44 |
| *AHSG* | NM_001622 | chr3 | 187812632 | 187813132 | -661 | 0.94 | 0.57 | 0.36 |
| *AIF1* | NM_001623 | chr6 | 31690454 | 31690951 | -308 | 0.75 | 0.12 | 0.63 |
| *AIFM1* | NM_001130846 | chrX | 129101247 | 129101737 | -1796 | 0.72 | 0.27 | 0.45 |
| *AIM1L* | NM_001039775 | chr1 | 26543518 | 26543994 | -726 | 0.76 | 0.26 | 0.50 |
| *AIPL1* | NM_001033054 | chr17 | 6280373 | 6280869 | -1378 | 0.73 | 0.40 | 0.33 |
| *AKAP2* | NM_001136562 | chr9 | 111927599 | 111928113 | 253 | 0.62 | 0.32 | 0.30 |
| *AKIRIN1* | NM_001136275 | chr1 | 39227633 | 39228097 | -1637 | 0.85 | 0.54 | 0.31 |
| *AKNA* | NM_030767 | chr9 | 116196431 | 116196926 | -172 | 0.79 | 0.40 | 0.39 |
| *AKR1B1* | NM_001628 | chr7 | 133795733 | 133796205 | -1541 | 0.86 | 0.41 | 0.45 |
| *AKR1C3* | NM_003739 | chr10 | 5124682 | 5125203 | -1624 | 0.60 | 0.23 | 0.37 |
| *ALAS1* | NM_000688 | chr3 | 52205273 | 52205464 | -1814 | 0.59 | 0.26 | 0.33 |
| *ALB* | NM_000477 | chr4 | 74488305 | 74488829 | -268 | 0.72 | 0.38 | 0.33 |
| *ALDH18A1* | NM_001017423 | chr10 | 97408851 | 97409045 | -2391 | 0.95 | 0.57 | 0.38 |
| *ALDH1B1* | NM_000692 | chr9 | 38380386 | 38380846 | -2085 | 0.74 | 0.42 | 0.32 |
| *ALDH1L1* | NM_012190 | chr3 | 127382814 | 127383317 | -890 | 0.50 | 0.16 | 0.34 |
| *ALDH3B1* | NM_000694 | chr11 | 67532475 | 67532983 | -1636 | 0.63 | 0.22 | 0.41 |
| *ALDH3B2* | NM_000695 | chr11 | 67198541 | 67199017 | -101 | 0.71 | 0.27 | 0.44 |
| *ALDOC* | NM_005165 | chr17 | 23929041 | 23929541 | -1213 | 0.86 | 0.51 | 0.36 |
| *ALG13* | NM_018466 | chrX | 110809850 | 110810226 | -1030 | 0.80 | 0.49 | 0.31 |
| *ALG6* | NM_013339 | chr1 | 63604983 | 63605490 | -611 | 0.75 | 0.45 | 0.30 |
| *ALKBH6* | NM_032878 | chr19 | 41197842 | 41198347 | -1113 | 0.94 | 0.53 | 0.41 |
| *ALPK1* | NM_001102406 | chr4 | 113436008 | 113436437 | -1724 | 0.83 | 0.52 | 0.30 |
| *ALPL* | NM_000478 | chr1 | 21706360 | 21706825 | -1851 | 0.77 | 0.39 | 0.38 |
| *ALPPL2* | NM_031313 | chr2 | 232979419 | 232979929 | -121 | 0.66 | 0.33 | 0.32 |
| *ALS2CL* | NM_147129 | chr3 | 46711536 | 46712014 | -1600 | 0.79 | 0.44 | 0.35 |
| *AMDHD1* | NM_152435 | chr12 | 94859257 | 94859764 | -1690 | 0.78 | 0.32 | 0.46 |
| *AMFR* | NM_001144 | chr16 | 55018225 | 55018717 | -1526 | 0.91 | 0.58 | 0.32 |
| *AMHR2* | NM_020547 | chr12 | 52103495 | 52104000 | -159 | 0.81 | 0.29 | 0.51 |
| *AMICA1* | NM_153206 | chr11 | 117591650 | 117591753 | -2417 | 0.83 | 0.47 | 0.36 |
| *AMPD2* | NM_203404 | chr1 | 109967691 | 109968201 | -1625 | 0.82 | 0.51 | 0.31 |
| *AMPH* | NM_001635 | chr7 | 38639907 | 38640016 | -2416 | 0.70 | 0.39 | 0.31 |
| *AMT* | NM_000481 | chr3 | 49435358 | 49435856 | -591 | 0.77 | 0.34 | 0.43 |
| *AMY2B* | NM_020978 | chr1 | 103897565 | 103898078 | -1022 | 0.75 | 0.34 | 0.41 |
| *AMZ1* | NM_133463 | chr7 | 2683798 | 2684345 | -1616 | 0.72 | 0.29 | 0.43 |
| *ANGPTL3* | NM_014495 | chr1 | 62833346 | 62833786 | -2208 | 0.74 | 0.39 | 0.35 |
| *ANK2* | NM_001148 | chr4 | 114188367 | 114188857 | -1621 | 0.87 | 0.43 | 0.44 |
| *ANK3* | NM_001149 | chr10 | 61571117 | 61571632 | -622 | 0.90 | 0.55 | 0.35 |
| *ANKRD2* | NM_001129981 | chr10 | 99320824 | 99321346 | -1160 | 0.91 | 0.56 | 0.35 |
| *ANKRD23* | NM_144994 | chr2 | 96873900 | 96874422 | -676 | 0.56 | 0.25 | 0.31 |
| *ANKRD39* | NM_016466 | chr2 | 96888922 | 96889424 | -1690 | 0.81 | 0.37 | 0.44 |
| *ANKRD55* | NM_001039935 | chr5 | 55449377 | 55449919 | -1113 | 0.74 | 0.38 | 0.35 |
| *ANKS4B* | NM_145865 | chr16 | 21151140 | 21151693 | -1099 | 0.85 | 0.45 | 0.39 |
| *ANO2* | NM_020373 | chr12 | 5926625 | 5927134 | -1220 | 0.94 | 0.61 | 0.33 |
| *ANO7* | NM_001001666 | chr2 | 241775896 | 241776396 | -450 | 0.66 | 0.32 | 0.34 |
| *ANTXR2* | NM_001145794 | chr4 | 81215386 | 81215893 | -2138 | 0.55 | 0.21 | 0.35 |
| *ANTXRL* | NR_003601 | chr10 | 47128214 | 47128732 | 234 | 0.86 | 0.49 | 0.38 |
| *ANXA1* | NM_000700 | chr9 | 74955139 | 74955670 | -1195 | 0.81 | 0.42 | 0.39 |
| *ANXA2P3* | NR_001446 | chr10 | 66253305 | 66253805 | -1735 | 0.89 | 0.57 | 0.32 |
| *ANXA8* | NM_001040084 | chr10 | 46593990 | 46594414 | -74 | 0.48 | 0.18 | 0.30 |
| *ANXA8L1* | NM_001098845 | chr10 | 46593990 | 46594414 | -156 | 0.48 | 0.18 | 0.30 |
| *ANXA8L2* | NM_001630 | chr10 | 47216609 | 47217101 | -70 | 0.48 | 0.17 | 0.31 |
| *AP1G2* | NM_003917 | chr14 | 23108823 | 23109305 | -1945 | 0.73 | 0.37 | 0.36 |
| *APLN* | NM_017413 | chrX | 128617251 | 128617745 | -903 | 0.75 | 0.45 | 0.30 |
| *APOA4* | NM_000482 | chr11 | 116201600 | 116201702 | -2430 | 0.69 | 0.22 | 0.47 |
| *APOA5* | NM_052968 | chr11 | 116168235 | 116168733 | -690 | 0.49 | 0.17 | 0.32 |
| *APOBEC3A* | NM_145699 | chr22 | 37682489 | 37682991 | -732 | 0.93 | 0.11 | 0.81 |
| *APOBEC3B* | NM_004900 | chr22 | 37707320 | 37707834 | -773 | 0.59 | 0.16 | 0.43 |
| *APOC2* | NM_000483 | chr19 | 50139369 | 50139915 | -1440 | 0.59 | 0.30 | 0.30 |
| *APOC3* | NM_000040 | chr11 | 116205414 | 116205892 | -180 | 0.74 | 0.18 | 0.55 |
| *APOC4* | NM_001646 | chr19 | 50135344 | 50135674 | -1825 | 0.80 | 0.42 | 0.38 |
| *APOL1* | NM_001136540 | chr22 | 34978740 | 34979246 | -69 | 0.74 | 0.25 | 0.50 |
| *APOL2* | NM_030882 | chr22 | 34965576 | 34966090 | -198 | 0.63 | 0.23 | 0.40 |
| *APOL4* | NM_030643 | chr22 | 34930671 | 34931213 | -117 | 0.55 | 0.13 | 0.42 |
| *APOL6* | NM_030641 | chr22 | 34373180 | 34373682 | -938 | 0.62 | 0.28 | 0.33 |
| *APP* | NM_001136016 | chr21 | 26434104 | 26434635 | 170 | 0.75 | 0.22 | 0.54 |
| *AQP1* | NM_198098 | chr7 | 30916003 | 30916519 | -1731 | 0.90 | 0.51 | 0.39 |
| *AQP6* | NM_001652 | chr12 | 48651996 | 48652498 | -639 | 0.67 | 0.30 | 0.36 |
| *AQP7* | NM_001170 | chr9 | 33394656 | 33394952 | -2287 | 0.87 | 0.57 | 0.30 |
| *AQP7P3* | NR_026558 | chr9 | 42848086 | 42848582 | 187 | 0.53 | 0.12 | 0.41 |
| *AR* | NM_000044 | chrX | 66678181 | 66678735 | -2140 | 0.86 | 0.56 | 0.30 |
| *ARAP1* | NM_001135190 | chr11 | 72112683 | 72113171 | -1876 | 0.82 | 0.36 | 0.46 |
| *ARAP3* | NM_022481 | chr5 | 141042837 | 141043366 | -1117 | 0.63 | 0.20 | 0.43 |
| *ARFGAP3* | NM_001142293 | chr22 | 41584047 | 41584366 | -854 | 0.78 | 0.47 | 0.31 |
| *ARG1* | NM_000045 | chr6 | 131935677 | 131936175 | -131 | 0.85 | 0.54 | 0.31 |
| *ARGFXP2* | NR_002222 | chr17 | 27505021 | 27505138 | -2376 | 0.60 | 0.20 | 0.40 |
| *ARHGAP15* | NM_018460 | chr2 | 143602484 | 143603002 | -625 | 0.68 | 0.26 | 0.43 |
| *ARHGAP23* | NM_020876 | chr17 | 33865279 | 33865803 | -1628 | 0.70 | 0.39 | 0.31 |
| *ARHGAP27* | NM_199282 | chr17 | 40859235 | 40859743 | -694 | 0.77 | 0.22 | 0.56 |
| *ARHGAP30* | NM_001025598 | chr1 | 159308551 | 159308765 | -2274 | 0.85 | 0.38 | 0.47 |
| *ARHGAP8* | NM_001017526 | chr22 | 43525355 | 43525685 | -1581 | 0.91 | 0.59 | 0.33 |
| *ARHGEF10L* | NM_001011722 | chr1 | 17779042 | 17779518 | -354 | 0.82 | 0.28 | 0.54 |
| *ARHGEF18* | NM_015318 | chr19 | 7364424 | 7364826 | -1373 | 0.88 | 0.50 | 0.38 |
| *ARHGEF4* | NM_015320 | chr2 | 131388725 | 131389227 | -1717 | 0.68 | 0.30 | 0.38 |
| *ARID3C* | NM_001017363 | chr9 | 34620176 | 34620482 | -2318 | 0.78 | 0.21 | 0.57 |
| *ARL13A* | NM_001012990 | chrX | 100108918 | 100109430 | -2181 | 0.84 | 0.38 | 0.47 |
| *ARL4D* | NM_001661 | chr17 | 38830378 | 38830679 | -1349 | 0.68 | 0.39 | 0.30 |
| *ARMC2* | NM_032131 | chr6 | 109274320 | 109274834 | -1734 | 0.51 | 0.08 | 0.43 |
| *ARMCX1* | NM_016608 | chrX | 100690080 | 100690608 | -1825 | 0.81 | 0.48 | 0.33 |
| *ARPC1A* | NM_006409 | chr7 | 98759513 | 98759921 | -1728 | 0.79 | 0.42 | 0.38 |
| *ARSD* | NM_001669 | chrX | 2858789 | 2859324 | -1664 | 0.83 | 0.49 | 0.35 |
| *ARSH* | NM_001011719 | chrX | 2934387 | 2934892 | -13 | 0.62 | 0.32 | 0.30 |
| *ARSI* | NM_001012301 | chr5 | 149663394 | 149663895 | -926 | 0.69 | 0.29 | 0.40 |
| *ASB11* | NM_001012428 | chrX | 15243066 | 15243566 | -726 | 0.68 | 0.30 | 0.37 |
| *ASB12* | NM_130388 | chrX | 63363172 | 63363689 | -1202 | 0.90 | 0.57 | 0.33 |
| *ASB13* | NM_024701 | chr10 | 5750536 | 5750978 | -2193 | 0.71 | 0.30 | 0.41 |
| *ASB15* | NM_080928 | chr7 | 123033924 | 123034466 | -2152 | 0.93 | 0.59 | 0.35 |
| *ASB16* | NM_080863 | chr17 | 39602880 | 39603384 | -467 | 0.64 | 0.33 | 0.31 |
| *ASF1A* | NM_014034 | chr6 | 119261719 | 119262227 | -1654 | 0.79 | 0.41 | 0.38 |
| *ASFMR1* | NR_024499 | chrX | 146813214 | 146813622 | -2050 | 0.78 | 0.33 | 0.45 |
| *ASGR1* | NM_001671 | chr17 | 7024002 | 7024325 | -556 | 0.73 | 0.41 | 0.32 |
| *ASIP* | NM_001672 | chr20 | 32309641 | 32310102 | -1959 | 0.91 | 0.58 | 0.34 |
| *ASPH* | NM_020164 | chr8 | 62765805 | 62766322 | -1138 | 0.47 | 0.11 | 0.35 |
| *ASPRV1* | NM_152792 | chr2 | 70043344 | 70043846 | -694 | 0.79 | 0.29 | 0.50 |
| *ASS1* | NM_000050 | chr9 | 132309042 | 132309524 | -631 | 0.68 | 0.34 | 0.33 |
| *ASTN2* | NM_198188 | chr9 | 118491564 | 118491753 | -2324 | 0.99 | 0.59 | 0.39 |
| *ATAD4* | NM_024320 | chr17 | 43390451 | 43390953 | -593 | 0.67 | 0.34 | 0.33 |
| *ATF3* | NM_001030287 | chr1 | 210804986 | 210805495 | -78 | 0.88 | 0.58 | 0.30 |
| *ATF7IP2* | NM_024997 | chr16 | 10428662 | 10428954 | -1417 | 0.94 | 0.64 | 0.30 |
| *ATL3* | NM_015459 | chr11 | 63196501 | 63196974 | -1078 | 0.87 | 0.46 | 0.41 |
| *ATN1* | NM_001007026 | chr12 | 6901451 | 6901916 | -2202 | 0.75 | 0.12 | 0.63 |
| *ATP11C* | NM_001010986 | chrX | 138742575 | 138743072 | -710 | 0.80 | 0.39 | 0.40 |
| *ATP13A3* | NM_024524 | chr3 | 195672576 | 195672683 | -2372 | 0.79 | 0.40 | 0.39 |
| *ATP2A1* | NM_004320 | chr16 | 28796363 | 28796860 | -697 | 0.78 | 0.47 | 0.30 |
| *ATP2B2* | NM_001001331 | chr3 | 10523713 | 10524207 | -1692 | 0.77 | 0.37 | 0.40 |
| *ATP6V0A2* | NM_012463 | chr12 | 122761383 | 122761901 | -1175 | 0.69 | 0.21 | 0.48 |
| *ATP6V1B1* | NM_001692 | chr2 | 71014955 | 71015439 | -1308 | 0.85 | 0.49 | 0.36 |
| *ATP7A* | NM_000052 | chrX | 77050428 | 77050929 | -2170 | 0.66 | 0.34 | 0.32 |
| *ATRN* | NM_139321 | chr20 | 3398197 | 3398743 | -1194 | 0.76 | 0.46 | 0.30 |
| *ATXN7L1* | NM_138495 | chr7 | 105106687 | 105107203 | -100 | 0.55 | 0.25 | 0.31 |
| *AVP* | NM_000490 | chr20 | 3013834 | 3014334 | -714 | 0.78 | 0.43 | 0.35 |
| *AWAT2* | NM_001002254 | chrX | 69186456 | 69186958 | -194 | 0.80 | 0.47 | 0.33 |
| *AXL* | NM_001699 | chr19 | 46416902 | 46417390 | 199 | 0.56 | 0.24 | 0.31 |
| *AYP1p1* | NR_002820 | chrY | 2716740 | 2717253 | -790 | 0.93 | 0.43 | 0.50 |
| *B3GALT2* | NM_003783 | chr1 | 191422824 | 191423359 | -725 | 0.51 | 0.20 | 0.31 |
| *BAIAP2L2* | NM_025045 | chr22 | 36837668 | 36838184 | -1304 | 0.73 | 0.28 | 0.45 |
| *BANK1* | NM_001083907 | chr4 | 102953034 | 102953518 | -729 | 0.85 | 0.04 | 0.82 |
| *BATF2* | NM_138456 | chr11 | 64522592 | 64523083 | -1744 | 0.85 | 0.33 | 0.52 |
| *BAX* | NM_004324 | chr19 | 54147712 | 54147949 | -2097 | 0.88 | 0.59 | 0.30 |
| *BCL2A1* | NM_001114735 | chr15 | 78051092 | 78051310 | -503 | 0.76 | 0.41 | 0.35 |
| *BCL2L10* | NM_020396 | chr15 | 50192932 | 50193382 | -893 | 0.64 | 0.21 | 0.43 |
| *BCL6* | NM_001706 | chr3 | 188947162 | 188947662 | -1243 | 0.66 | 0.30 | 0.36 |
| *BCO2* | NM_001037290 | chr11 | 111552109 | 111552604 | 58 | 0.61 | 0.26 | 0.35 |
| *BCORL2* | NR_002923 | chrY | 20126329 | 20126821 | -2148 | 0.77 | 0.36 | 0.41 |
| *BDH1* | NM_203315 | chr3 | 198784563 | 198785069 | -225 | 0.49 | 0.17 | 0.32 |
| *BEST1* | NM_001139443 | chr11 | 61471501 | 61471965 | -2198 | 0.82 | 0.43 | 0.39 |
| *BEST4* | NM_153274 | chr1 | 45026052 | 45026530 | -278 | 0.83 | 0.44 | 0.38 |
| *BFSP1* | NM_001195 | chr20 | 17461255 | 17461664 | -1485 | 0.81 | 0.48 | 0.34 |
| *BGLAP* | NM_199173 | chr1 | 154478228 | 154478708 | -106 | 0.67 | 0.31 | 0.36 |
| *BHLHB9* | NM_001142524 | chrX | 101862103 | 101862605 | 57 | 0.47 | 0.09 | 0.39 |
| *BHMT2* | NM_017614 | chr5 | 78399396 | 78399841 | -1719 | 0.48 | 0.16 | 0.33 |
| *BIN3* | NM_018688 | chr8 | 22583067 | 22583567 | -711 | 0.75 | 0.32 | 0.42 |
| *BIRC7* | NM_022161 | chr20 | 61336753 | 61337251 | -718 | 0.67 | 0.38 | 0.30 |
| *BLNK* | NM_001114094 | chr10 | 98022267 | 98022823 | -1222 | 0.88 | 0.43 | 0.45 |
| *BMF* | NM_001003942 | chr15 | 38185518 | 38186018 | -189 | 0.69 | 0.33 | 0.36 |
| *BOLA2* | NM_001031827 | chr16 | 29376234 | 29376722 | -2692 | 0.66 | 0.23 | 0.44 |
| *BOLA2B* | NM_001039182 | chr16 | 29376234 | 29376722 | -2692 | 0.66 | 0.23 | 0.44 |
| *BPIL1* | NM_025227 | chr20 | 31058816 | 31059322 | 2 | 0.87 | 0.44 | 0.43 |
| *BRD4* | NM_014299 | chr19 | 15253513 | 15253991 | -1490 | 0.77 | 0.44 | 0.33 |
| *BRUNOL6* | NM_052840 | chr15 | 70400018 | 70400468 | -664 | 0.69 | 0.36 | 0.33 |
| *BTBD11* | NM_001017523 | chr12 | 106498300 | 106498777 | -224 | 0.62 | 0.32 | 0.30 |
| *BTK* | NM_000061 | chrX | 100530380 | 100530889 | -2766 | 0.61 | 0.26 | 0.35 |
| *BTNL9* | NM_152547 | chr5 | 180397391 | 180397841 | -2214 | 0.64 | 0.30 | 0.34 |
| *BUB3* | NM_001007793 | chr10 | 124902581 | 124903067 | -925 | 0.77 | 0.24 | 0.53 |
| *BZRAP1* | NM_004758 | chr17 | 53761994 | 53762510 | -1101 | 0.83 | 0.28 | 0.55 |
| *BZRPL1* | NM_001010873 | chr6 | 41116751 | 41117269 | -1260 | 0.90 | 0.20 | 0.70 |
| *C10orf10* | NM_007021 | chr10 | 44794676 | 44795190 | -597 | 0.85 | 0.20 | 0.66 |
| *C10orf129* | NM_207321 | chr10 | 96941509 | 96941993 | -2195 | 0.82 | 0.51 | 0.31 |
| *C10orf26* | NM_017787 | chr10 | 104525639 | 104526135 | 10 | 0.81 | 0.29 | 0.52 |
| *C10orf27* | NM_152710 | chr10 | 72215107 | 72215627 | -204 | 0.76 | 0.38 | 0.38 |
| *C10orf40* | NR_024340 | chr10 | 61391125 | 61391663 | -717 | 0.43 | 0.09 | 0.34 |
| *C10orf50* | NR_026793 | chr10 | 26916374 | 26916921 | -2151 | 0.82 | 0.50 | 0.32 |
| *C10orf54* | NM_022153 | chr10 | 73205190 | 73205694 | -2099 | 0.86 | 0.28 | 0.58 |
| *C10orf55* | NM_001001791 | chr10 | 75354563 | 75354732 | -2106 | 0.69 | 0.38 | 0.30 |
| *C10orf58* | NR_024572 | chr10 | 82163184 | 82163702 | -127 | 0.78 | 0.29 | 0.49 |
| *C10orf62* | NM_001009997 | chr10 | 99338865 | 99339361 | -360 | 0.71 | 0.39 | 0.31 |
| *C10orf71* | NM_001135196 | chr10 | 50175723 | 50176223 | -1219 | 0.55 | 0.14 | 0.41 |
| *C10orf72* | NM_001031746 | chr10 | 49994413 | 49994908 | -1095 | 0.90 | 0.12 | 0.77 |
| *C10orf79* | NM_025145 | chr10 | 105984333 | 105984536 | -2324 | 0.56 | 0.25 | 0.31 |
| *C10orf81* | NM_024889 | chr10 | 115501081 | 115501561 | 119 | 0.44 | 0.07 | 0.37 |
| *C11orf2* | NM_013265 | chr11 | 64618357 | 64618906 | -1626 | 0.79 | 0.29 | 0.50 |
| *C11orf21* | NM_001142946 | chr11 | 2279429 | 2279927 | 41 | 0.59 | 0.28 | 0.31 |
| *C11orf42* | NM_173525 | chr11 | 6182934 | 6183429 | -191 | 0.71 | 0.33 | 0.38 |
| *C11orf59* | NM_017907 | chr11 | 71492910 | 71493414 | -1192 | 0.80 | 0.38 | 0.42 |
| *C11orf63* | NM_024806 | chr11 | 122257922 | 122258425 | -508 | 0.58 | 0.28 | 0.30 |
| *C11orf86* | NM_001136485 | chr11 | 66499021 | 66499464 | -86 | 0.85 | 0.42 | 0.44 |
| *C12orf34* | NM_032829 | chr12 | 108655069 | 108655595 | -1622 | 0.84 | 0.53 | 0.32 |
| *C12orf42* | NM_001099336 | chr12 | 102416131 | 102416347 | -2360 | 0.82 | 0.42 | 0.40 |
| *C12orf50* | NM_152589 | chr12 | 86947164 | 86947656 | -103 | 0.64 | 0.34 | 0.30 |
| *C12orf71* | NM_001080406 | chr12 | 27128768 | 27128940 | -2132 | 0.64 | 0.20 | 0.44 |
| *C13orf16* | NM_152324 | chr13 | 110769634 | 110770166 | -1115 | 0.83 | 0.53 | 0.30 |
| *C13orf38* | NM_001144981 | chr13 | 35771987 | 35772389 | -2196 | 0.71 | 0.33 | 0.38 |
| *C14orf115* | NM_018228 | chr14 | 73883763 | 73884261 | -906 | 0.88 | 0.51 | 0.37 |
| *C14orf139* | NR_026779 | chr14 | 94948120 | 94948620 | -2190 | 0.63 | 0.22 | 0.41 |
| *C14orf147* | NM_138288 | chr14 | 34002250 | 34002806 | -1309 | 0.87 | 0.48 | 0.39 |
| *C14orf182* | NM_001012706 | chr14 | 49543842 | 49544338 | -102 | 0.76 | 0.28 | 0.48 |
| *C14orf39* | NM_174978 | chr14 | 60024209 | 60024709 | -1942 | 0.81 | 0.49 | 0.32 |
| *C14orf48* | NR_024183 | chr14 | 93533382 | 93533878 | 236 | 0.91 | 0.50 | 0.41 |
| *C14orf49* | NM_152592 | chr14 | 95012278 | 95012791 | -608 | 0.89 | 0.49 | 0.40 |
| *C14orf68* | NM_207117 | chr14 | 99859318 | 99859826 | 141 | 0.78 | 0.25 | 0.54 |
| *C14orf73* | NM_001077594 | chr14 | 102633811 | 102634348 | -2153 | 0.58 | 0.15 | 0.43 |
| *C15orf28* | NR_026808 | chr15 | 66886447 | 66886956 | -207 | 0.79 | 0.47 | 0.32 |
| *C15orf44* | NM_001136043 | chr15 | 63692813 | 63693005 | -2387 | 0.78 | 0.48 | 0.30 |
| *C15orf5* | NR_026813 | chr15 | 75305237 | 75305792 | -713 | 0.89 | 0.59 | 0.30 |
| *C15orf50* | NR_026764 | chr15 | 67913696 | 67914209 | -673 | 0.59 | 0.29 | 0.31 |
| *C15orf56* | NM_001039905 | chr15 | 38334770 | 38334860 | -2413 | 0.95 | 0.51 | 0.44 |
| *C15orf62* | NM_001130448 | chr15 | 38849005 | 38849503 | -215 | 0.55 | 0.12 | 0.43 |
| *C16orf35* | NM_001039476 | chr16 | 130081 | 130609 | -1735 | 0.81 | 0.49 | 0.33 |
| *C16orf59* | NM_025108 | chr16 | 2447676 | 2448127 | -2213 | 0.82 | 0.50 | 0.32 |
| *C16orf79* | NM_182563 | chr16 | 2202356 | 2202876 | -1604 | 0.77 | 0.43 | 0.34 |
| *C16orf90* | NM_001080524 | chr16 | 3485361 | 3485785 | -151 | 0.92 | 0.43 | 0.48 |
| *C16orf92* | NM_001109659 | chr16 | 29941664 | 29942140 | -253 | 0.72 | 0.38 | 0.33 |
| *C17orf64* | NM_181707 | chr17 | 55854455 | 55854978 | 70 | 0.74 | 0.39 | 0.35 |
| *C17orf67* | NM_001085430 | chr17 | 52249751 | 52250218 | -1735 | 0.85 | 0.55 | 0.30 |
| *C17orf74* | NM_175734 | chr17 | 7268942 | 7269395 | -489 | 0.71 | 0.16 | 0.55 |
| *C17orf91* | NM_001001870 | chr17 | 1568116 | 1568611 | -2108 | 0.78 | 0.46 | 0.32 |
| *C19orf38* | NM_001136482 | chr19 | 10819934 | 10820419 | 71 | 0.66 | 0.29 | 0.37 |
| *C19orf46* | NM_001039876 | chr19 | 41192912 | 41193423 | -1655 | 0.63 | 0.11 | 0.52 |
| *C19orf71* | NM_001135580 | chr19 | 3487715 | 3488180 | -2206 | 0.59 | 0.27 | 0.32 |
| *C1orf101* | NM_001130957 | chr1 | 242689892 | 242690397 | -1150 | 0.65 | 0.30 | 0.35 |
| *C1orf106* | NM_001142569 | chr1 | 199129475 | 199129967 | -900 | 0.78 | 0.27 | 0.51 |
| *C1orf127* | NM_173507 | chr1 | 10946684 | 10947208 | -101 | 0.59 | 0.21 | 0.39 |
| *C1orf129* | NM_025063 | chr1 | 169170289 | 169170779 | -725 | 0.96 | 0.60 | 0.35 |
| *C1orf168* | NM_001004303 | chr1 | 57058692 | 57059198 | -988 | 0.68 | 0.25 | 0.43 |
| *C1orf183* | NM_198926 | chr1 | 112102017 | 112102346 | -2239 | 0.80 | 0.46 | 0.34 |
| *C1orf198* | NM_001136495 | chr1 | 229059782 | 229060270 | -1621 | 0.73 | 0.20 | 0.53 |
| *C1orf2* | NM_006589 | chr1 | 153492718 | 153493198 | -1060 | 0.69 | 0.25 | 0.44 |
| *C1orf204* | NM_001134233 | chr1 | 158093096 | 158093582 | -1578 | 0.91 | 0.54 | 0.37 |
| *C1orf228* | NM_001145636 | chr1 | 44910564 | 44911111 | -2142 | 0.64 | 0.34 | 0.31 |
| *C1orf52* | NM_198077 | chr1 | 85499259 | 85499757 | -1565 | 0.87 | 0.56 | 0.31 |
| *C1orf59* | NM_001102592 | chr1 | 109007212 | 109007709 | -2193 | 0.98 | 0.42 | 0.56 |
| *C1orf61* | NM_006365 | chr1 | 154667148 | 154667648 | -1590 | 0.84 | 0.45 | 0.38 |
| *C1QA* | NM_015991 | chr1 | 22833276 | 22833753 | -2189 | 0.68 | 0.26 | 0.42 |
| *C1QC* | NM_001114101 | chr1 | 22841758 | 22842238 | -706 | 0.87 | 0.45 | 0.42 |
| *C1QTNF1* | NM_198593 | chr17 | 74539651 | 74540198 | -2141 | 0.81 | 0.44 | 0.38 |
| *C1QTNF4* | NM_031909 | chr11 | 47573477 | 47573987 | -1195 | 0.80 | 0.21 | 0.59 |
| *C1QTNF5* | NM_015645 | chr11 | 118722581 | 118723057 | -226 | 0.83 | 0.25 | 0.58 |
| *C1QTNF6* | NM_031910 | chr22 | 35914615 | 35915132 | -597 | 0.85 | 0.47 | 0.38 |
| *C1QTNF9* | NM_178540 | chr13 | 23779294 | 23779725 | -2205 | 0.81 | 0.50 | 0.30 |
| *C1RL* | NM_016546 | chr12 | 7153452 | 7153989 | -651 | 0.50 | 0.18 | 0.32 |
| *C1S* | NM_001734 | chr12 | 7038225 | 7038742 | 243 | 0.69 | 0.35 | 0.35 |
| *C20orf114* | NM_033197 | chr20 | 31332183 | 31332520 | -2249 | 0.66 | 0.31 | 0.35 |
| *C20orf144* | NM_080825 | chr20 | 31712390 | 31712934 | -1118 | 0.85 | 0.54 | 0.31 |
| *C20orf152* | NM_080834 | chr20 | 34019341 | 34019865 | -339 | 0.86 | 0.51 | 0.35 |
| *C20orf173* | NM_001145350 | chr20 | 33580836 | 33581150 | -98 | 0.71 | 0.33 | 0.38 |
| *C20orf185* | NM_182658 | chr20 | 31104453 | 31105002 | -2162 | 0.82 | 0.36 | 0.45 |
| *C20orf191* | NR_003678 | chr20 | 26044070 | 26044525 | -1620 | 0.94 | 0.58 | 0.37 |
| *C20orf29* | NM_018347 | chr20 | 3747820 | 3748344 | -1120 | 0.66 | 0.34 | 0.32 |
| *C20orf71* | NM_001042439 | chr20 | 31268590 | 31269080 | 40 | 0.73 | 0.23 | 0.50 |
| *C21orf109* | NR_027072 | chr21 | 29486684 | 29487172 | -743 | 0.88 | 0.48 | 0.40 |
| *C21orf125* | NR_026960 | chr21 | 43693464 | 43693958 | -620 | 0.63 | 0.31 | 0.32 |
| *C21orf129* | NR_027272 | chr21 | 42010778 | 42011275 | -2022 | 0.73 | 0.33 | 0.40 |
| *C21orf130* | NR_024100 | chr21 | 41443326 | 41443832 | -1718 | 0.69 | 0.27 | 0.42 |
| *C21orf131* | NR_024090 | chr21 | 21099161 | 21099663 | -2115 | 0.83 | 0.21 | 0.62 |
| *C21orf34* | NM_001005732 | chr21 | 16362826 | 16363326 | -1636 | 0.93 | 0.42 | 0.51 |
| *C21orf41* | NR_002214 | chr21 | 29926910 | 29927382 | -2204 | 0.92 | 0.56 | 0.36 |
| *C21orf84* | NR_026863 | chr21 | 43722481 | 43722995 | -207 | 0.65 | 0.34 | 0.32 |
| *C21orf90* | NR_026547 | chr21 | 44761149 | 44761639 | -131 | 0.80 | 0.43 | 0.37 |
| *C22orf31* | NM_015370 | chr22 | 27788497 | 27788997 | -840 | 0.78 | 0.33 | 0.45 |
| *C22orf33* | NM_178552 | chr22 | 35735727 | 35736243 | -2200 | 0.68 | 0.10 | 0.58 |
| *C2CD2L* | NM_014807 | chr11 | 118480928 | 118481236 | -2220 | 0.85 | 0.55 | 0.31 |
| *C2orf14* | NR_023391 | chr2 | 131151699 | 131152154 | -2165 | 0.75 | 0.13 | 0.63 |
| *C2orf43* | NM_021925 | chr2 | 20887815 | 20888331 | -1765 | 0.67 | 0.35 | 0.32 |
| *C2orf52* | NR_024079 | chr2 | 232089293 | 232089563 | -2134 | 0.77 | 0.41 | 0.36 |
| *C2orf54* | NM_001085437 | chr2 | 241486232 | 241486714 | -2227 | 0.61 | 0.31 | 0.30 |
| *C2orf57* | NM_152614 | chr2 | 232165117 | 232165599 | -497 | 0.80 | 0.43 | 0.37 |
| *C2orf61* | NM_173649 | chr2 | 47236419 | 47236922 | -740 | 0.81 | 0.36 | 0.45 |
| *C2orf74* | NM_001143960 | chr2 | 61223497 | 61224010 | -1992 | 0.80 | 0.48 | 0.31 |
| *C2orf86* | NM_001042692 | chr2 | 63519439 | 63519939 | -1099 | 0.82 | 0.38 | 0.45 |
| *C2orf88* | NM_001042521 | chr2 | 190709866 | 190710355 | -619 | 0.86 | 0.35 | 0.52 |
| *C3AR1* | NM_004054 | chr12 | 8110589 | 8111080 | -612 | 0.76 | 0.40 | 0.37 |
| *C3orf25* | NM_207307 | chr3 | 130631925 | 130632412 | -1984 | 0.86 | 0.50 | 0.36 |
| *C3orf27* | NM_007354 | chr3 | 129778470 | 129778808 | -1020 | 0.76 | 0.43 | 0.33 |
| *C3orf36* | NM_025041 | chr3 | 135133694 | 135133792 | -2397 | 0.84 | 0.47 | 0.37 |
| *C3orf39* | NM_032806 | chr3 | 43124406 | 43124887 | -2077 | 0.85 | 0.50 | 0.35 |
| *C3orf62* | NM_198562 | chr3 | 49291852 | 49291954 | -2391 | 0.86 | 0.48 | 0.37 |
| *C3orf74* | NM_001123039 | chr3 | 52075980 | 52076470 | -2057 | 0.75 | 0.32 | 0.44 |
| *C3P1* | NR_027300 | chr19 | 10012608 | 10013115 | -169 | 0.89 | 0.34 | 0.55 |
| *C4orf19* | NM_018302 | chr4 | 37259833 | 37260314 | -2175 | 0.80 | 0.24 | 0.56 |
| *C4orf23* | NM_152544 | chr4 | 8505954 | 8506463 | -833 | 0.70 | 0.27 | 0.43 |
| *C4orf40* | NM_214711 | chr4 | 71052071 | 71052438 | -2237 | 0.96 | 0.54 | 0.43 |
| *C4orf44* | NM_001012982 | chr4 | 3219179 | 3219697 | -1126 | 0.69 | 0.39 | 0.30 |
| *C4orf7* | NM_152997 | chr4 | 71124964 | 71125474 | -1184 | 0.98 | 0.67 | 0.31 |
| *C4orf8* | NM_003704 | chr4 | 2595039 | 2595515 | -1679 | 0.79 | 0.49 | 0.30 |
| *C5* | NM_001735 | chr9 | 122852319 | 122852835 | -202 | 0.77 | 0.40 | 0.37 |
| *C5AR1* | NM_001736 | chr19 | 52503464 | 52503578 | -1422 | 0.88 | 0.58 | 0.31 |
| *C5orf24* | NM_001135586 | chr5 | 134207492 | 134208040 | -1790 | 0.76 | 0.46 | 0.30 |
| *C5orf32* | NM_032412 | chr5 | 139533948 | 139534444 | -640 | 0.80 | 0.47 | 0.33 |
| *C5orf4* | NM_016348 | chr5 | 154182075 | 154182546 | -1031 | 0.84 | 0.50 | 0.34 |
| *C5orf53* | NM_001007189 | chr5 | 139485288 | 139485813 | -153 | 0.61 | 0.18 | 0.43 |
| *C6orf122* | NR_026781 | chr6 | 169942794 | 169943287 | -2194 | 0.75 | 0.45 | 0.30 |
| *C6orf136* | NM_001109938 | chr6 | 30721794 | 30722296 | -734 | 0.64 | 0.25 | 0.38 |
| *C6orf141* | NM_001145652 | chr6 | 49625262 | 49625713 | -583 | 0.75 | 0.38 | 0.38 |
| *C6orf165* | NM_001031743 | chr6 | 88173055 | 88173566 | -1128 | 0.72 | 0.41 | 0.31 |
| *C6orf208* | NR_026780 | chr6 | 169931202 | 169931702 | -641 | 0.58 | 0.24 | 0.33 |
| *C6orf223* | NM_153246 | chr6 | 44076078 | 44076580 | 13 | 0.86 | 0.30 | 0.56 |
| *C6orf25* | NM_025260 | chr6 | 31797249 | 31797751 | -1639 | 0.52 | 0.12 | 0.40 |
| *C6orf47* | NM_021184 | chr6 | 31737519 | 31738016 | -1239 | 0.77 | 0.27 | 0.51 |
| *C6orf81* | NM_145028 | chr6 | 35810594 | 35811143 | -1967 | 0.76 | 0.35 | 0.41 |
| *C7* | NM_000587 | chr5 | 40943692 | 40944204 | -1407 | 0.86 | 0.41 | 0.44 |
| *C7orf42* | NM_017994 | chr7 | 66021777 | 66022062 | -1717 | 0.72 | 0.37 | 0.35 |
| *C7orf53* | NM_001134468 | chr7 | 111905728 | 111906280 | -2139 | 0.89 | 0.42 | 0.47 |
| *C7orf67* | NM_032222 | chr7 | 30775664 | 30776148 | -1651 | 0.87 | 0.34 | 0.53 |
| *C7orf69* | NM_025031 | chr7 | 47800719 | 47800957 | -575 | 0.84 | 0.35 | 0.49 |
| *C8orf44* | NM_019607 | chr8 | 67749620 | 67749949 | -1222 | 0.81 | 0.46 | 0.34 |
| *C8orf58* | NM_001013842 | chr8 | 22510651 | 22511176 | -2152 | 0.67 | 0.32 | 0.35 |
| *C8orf73* | NM_001100878 | chr8 | 144727622 | 144728128 | -1804 | 0.80 | 0.44 | 0.36 |
| *C8orf80* | NM_001010906 | chr8 | 27998460 | 27998929 | -1387 | 0.73 | 0.31 | 0.42 |
| *C8orf83* | NR_015339 | chr8 | 94048483 | 94048874 | -1163 | 0.81 | 0.51 | 0.30 |
| *C8orf86* | NM_207412 | chr8 | 38507086 | 38507551 | -1981 | 0.88 | 0.56 | 0.32 |
| *C9orf106* | NM_001012715 | chr9 | 131120692 | 131121234 | -2152 | 0.70 | 0.16 | 0.54 |
| *C9orf11* | NM_020641 | chr9 | 27288356 | 27288882 | -1482 | 0.84 | 0.44 | 0.40 |
| *C9orf114* | NM_016390 | chr9 | 130633844 | 130634317 | -2174 | 0.78 | 0.37 | 0.41 |
| *C9orf117* | NM_001012502 | chr9 | 129508150 | 129508670 | -681 | 0.77 | 0.37 | 0.40 |
| *C9orf131* | NM_001040410 | chr9 | 35032064 | 35032562 | 91 | 0.59 | 0.09 | 0.50 |
| *C9orf142* | NM_183241 | chr9 | 139004273 | 139004722 | -2192 | 0.80 | 0.48 | 0.33 |
| *C9orf24* | NM_032596 | chr9 | 34387788 | 34388318 | -204 | 0.60 | 0.21 | 0.39 |
| *C9orf44* | NR_027341 | chr9 | 93941586 | 93942092 | -1730 | 0.73 | 0.41 | 0.32 |
| *C9orf53* | NR_024274 | chr9 | 21956959 | 21957463 | 74 | 0.88 | 0.41 | 0.47 |
| *C9orf61* | NM_004816 | chr9 | 71133618 | 71134105 | -198 | 0.79 | 0.47 | 0.32 |
| *C9orf64* | NM_032307 | chr9 | 85763092 | 85763438 | -1782 | 0.76 | 0.31 | 0.44 |
| *C9orf79* | NM_178828 | chr9 | 89687496 | 89687976 | 145 | 0.74 | 0.41 | 0.33 |
| *C9orf80* | NM_021218 | chr9 | 114521756 | 114522247 | -1793 | 0.88 | 0.52 | 0.36 |
| *CABP2* | NM_016366 | chr11 | 67048815 | 67049339 | -1602 | 0.72 | 0.40 | 0.32 |
| *CABP4* | NM_145200 | chr11 | 66977866 | 66978367 | -1276 | 0.81 | 0.49 | 0.33 |
| *CACNA1I* | NM_001003406 | chr22 | 38296442 | 38296926 | -19 | 0.75 | 0.37 | 0.38 |
| *CACNB3* | NM_000725 | chr12 | 47496338 | 47496787 | -2215 | 0.88 | 0.43 | 0.45 |
| *CADM2* | NM_153184 | chr3 | 85857366 | 85857876 | -700 | 0.89 | 0.31 | 0.58 |
| *CALHM1* | NM_001001412 | chr10 | 105209818 | 105210342 | -1442 | 0.91 | 0.40 | 0.51 |
| *CALHM2* | NM_015916 | chr10 | 105202516 | 105202938 | -575 | 0.65 | 0.30 | 0.35 |
| *CALHM3* | NM_001129742 | chr10 | 105230321 | 105230803 | -1575 | 0.76 | 0.41 | 0.35 |
| *CALML3* | NM_005185 | chr10 | 5555954 | 5556452 | -720 | 0.77 | 0.35 | 0.42 |
| *CALML4* | NM_001031733 | chr15 | 66286357 | 66286865 | -1109 | 0.82 | 0.49 | 0.34 |
| *CALML6* | NM_138705 | chr1 | 1835335 | 1835857 | -529 | 0.77 | 0.40 | 0.37 |
| *CAMK1* | NM_003656 | chr3 | 9787125 | 9787625 | -714 | 0.57 | 0.19 | 0.38 |
| *CAMKV* | NM_024046 | chr3 | 49883690 | 49884192 | -1568 | 0.82 | 0.38 | 0.43 |
| *CAMP* | NM_004345 | chr3 | 48239819 | 48240301 | 195 | 0.67 | 0.35 | 0.32 |
| *CAMSAP1L1* | NM_203459 | chr1 | 198972870 | 198972928 | -2409 | 0.76 | 0.39 | 0.38 |
| *CAPN1* | NM_005186 | chr11 | 64703975 | 64704435 | -1713 | 0.77 | 0.40 | 0.38 |
| *CAPN11* | NM_007058 | chr6 | 44234071 | 44234572 | -203 | 0.82 | 0.36 | 0.46 |
| *CAPN12* | NM_144691 | chr19 | 43926894 | 43927430 | -208 | 0.72 | 0.41 | 0.32 |
| *CAPNS2* | NM_032330 | chr16 | 54156721 | 54157202 | -1122 | 0.86 | 0.34 | 0.52 |
| *CAPS* | NM_004058 | chr19 | 5864427 | 5864896 | -530 | 0.81 | 0.48 | 0.34 |
| *CARD9* | NM_052813 | chr9 | 138389919 | 138390431 | -2221 | 0.80 | 0.44 | 0.36 |
| *CASKIN2* | NM_001142643 | chr17 | 71017892 | 71018207 | -496 | 0.80 | 0.38 | 0.42 |
| *CASP12* | NR_000035 | chr11 | 104276621 | 104277032 | -2219 | 0.89 | 0.46 | 0.42 |
| *CASP8* | NM_001080125 | chr2 | 201828583 | 201829064 | -2174 | 0.83 | 0.53 | 0.30 |
| *CASR* | NM_000388 | chr3 | 123383240 | 123383740 | -1729 | 0.63 | 0.24 | 0.39 |
| *CAST* | NM_173061 | chr5 | 96104538 | 96105057 | -201 | 0.84 | 0.50 | 0.34 |
| *CAT* | NM_001752 | chr11 | 34414613 | 34414864 | -2314 | 0.96 | 0.56 | 0.40 |
| *CATSPER4* | NM_198137 | chr1 | 26387838 | 26388314 | -1629 | 0.58 | 0.21 | 0.38 |
| *CBFA2T3* | NM_005187 | chr16 | 87572747 | 87573264 | -2103 | 0.70 | 0.41 | 0.30 |
| *CBR1* | NM_001757 | chr21 | 36363163 | 36363654 | -745 | 0.63 | 0.28 | 0.34 |
| *CBR3* | NM_001236 | chr21 | 36428366 | 36428850 | -524 | 0.77 | 0.45 | 0.32 |
| *CCDC106* | NM_013301 | chr19 | 60849977 | 60850500 | -526 | 0.62 | 0.32 | 0.31 |
| *CCDC113* | NM_001142302 | chr16 | 56839450 | 56839930 | -1650 | 0.79 | 0.45 | 0.34 |
| *CCDC120* | NM_033626 | chrX | 48802186 | 48802712 | -1010 | 0.92 | 0.54 | 0.38 |
| *CCDC136* | NM_022742 | chr7 | 128217355 | 128217857 | -1728 | 0.64 | 0.32 | 0.32 |
| *CCDC141* | NM_173648 | chr2 | 179459267 | 179459795 | -1608 | 0.71 | 0.34 | 0.37 |
| *CCDC151* | NM_145045 | chr19 | 11408407 | 11408603 | -1525 | 0.69 | 0.34 | 0.35 |
| *CCDC152* | NM_001134848 | chr5 | 42790804 | 42791331 | -1608 | 0.87 | 0.45 | 0.42 |
| *CCDC158* | NM_001042784 | chr4 | 77549197 | 77549738 | -1985 | 0.80 | 0.44 | 0.36 |
| *CCDC17* | NM_001114938 | chr1 | 45861847 | 45862333 | 226 | 0.80 | 0.34 | 0.46 |
| *CCDC33* | NM_182791 | chr15 | 72397458 | 72397946 | -250 | 0.73 | 0.27 | 0.46 |
| *CCDC42* | NM_001158261 | chr17 | 8590866 | 8591351 | -2229 | 0.76 | 0.44 | 0.33 |
| *CCDC43* | NM_001099225 | chr17 | 40125005 | 40125110 | -2366 | 0.82 | 0.43 | 0.39 |
| *CCDC65* | NM_033124 | chr12 | 47583862 | 47584371 | -42 | 0.48 | 0.14 | 0.34 |
| *CCDC67* | NM_181645 | chr11 | 92702614 | 92703099 | -673 | 0.86 | 0.55 | 0.30 |
| *CCDC68* | NM_001143829 | chr18 | 50776230 | 50776730 | -203 | 0.76 | 0.39 | 0.37 |
| *CCDC71* | NM_022903 | chr3 | 49179151 | 49179647 | -610 | 0.86 | 0.37 | 0.49 |
| *CCDC86* | NM_024098 | chr11 | 60363588 | 60363913 | -2253 | 0.74 | 0.33 | 0.40 |
| *CCDC91* | NM_018318 | chr12 | 28299806 | 28300292 | -1350 | 0.63 | 0.30 | 0.33 |
| *CCL14* | NM_004166 | chr17 | 31355112 | 31355621 | -2153 | 0.83 | 0.47 | 0.36 |
| *CCL15* | NM_004167 | chr17 | 31355112 | 31355621 | -2153 | 0.83 | 0.47 | 0.36 |
| *CCL17* | NM_002987 | chr16 | 55994296 | 55994519 | -1771 | 0.63 | 0.13 | 0.49 |
| *CCL19* | NM_006274 | chr9 | 34681213 | 34681715 | -190 | 0.83 | 0.13 | 0.70 |
| *CCL28* | NM_148672 | chr5 | 43449239 | 43449669 | -1209 | 0.77 | 0.45 | 0.32 |
| *CCL4L1* | NM_001001435 | chr17 | 31662241 | 31662635 | -1708 | 0.83 | 0.38 | 0.45 |
| *CCL4L2* | NM_207007 | chr17 | 31662241 | 31662635 | -1708 | 0.83 | 0.38 | 0.45 |
| *CCNB1IP1* | NM_182851 | chr14 | 19869424 | 19869818 | -2245 | 0.92 | 0.56 | 0.36 |
| *CCND3* | NM_001136125 | chr6 | 42018234 | 42018497 | -835 | 0.85 | 0.51 | 0.34 |
| *CCNDBP1* | NM_012142 | chr15 | 41263901 | 41264382 | -615 | 0.83 | 0.53 | 0.30 |
| *CCR7* | NM_001838 | chr17 | 35975096 | 35975590 | -93 | 0.82 | 0.30 | 0.52 |
| *CCRL1* | NM_178445 | chr3 | 133799350 | 133799831 | -2079 | 0.84 | 0.52 | 0.33 |
| *CCRL2* | NM_001130910 | chr3 | 46422776 | 46423266 | -1031 | 0.64 | 0.23 | 0.41 |
| *CCT2* | NM_006431 | chr12 | 68264706 | 68265212 | -515 | 0.59 | 0.26 | 0.34 |
| *CD248* | NM_020404 | chr11 | 65843002 | 65843487 | -2153 | 0.80 | 0.40 | 0.40 |
| *CD28* | NM_006139 | chr2 | 204277558 | 204278069 | -1628 | 0.87 | 0.44 | 0.43 |
| *CD300E* | NM_181449 | chr17 | 70132262 | 70132795 | -1132 | 0.63 | 0.27 | 0.36 |
| *CD300LF* | NM_139018 | chr17 | 70223019 | 70223143 | -2378 | 0.78 | 0.40 | 0.38 |
| *CD302* | NM_014880 | chr2 | 160364435 | 160364980 | -1695 | 0.84 | 0.53 | 0.31 |
| *CD37* | NM_001040031 | chr19 | 54528531 | 54529074 | -1685 | 0.59 | 0.28 | 0.31 |
| *CD58* | NM_001144822 | chr1 | 116915932 | 116916446 | -951 | 0.69 | 0.32 | 0.37 |
| *CD59* | NM_001127223 | chr11 | 33702473 | 33702963 | -1869 | 0.88 | 0.33 | 0.54 |
| *CD72* | NM_001782 | chr9 | 35608986 | 35609477 | -807 | 0.86 | 0.56 | 0.30 |
| *CD84* | NM_003874 | chr1 | 158816352 | 158816832 | -717 | 0.55 | 0.23 | 0.32 |
| *CD8A* | NM_001768 | chr2 | 86874488 | 86874810 | -2301 | 0.91 | 0.54 | 0.37 |
| *CD93* | NM_012072 | chr20 | 23015852 | 23016366 | -1132 | 0.60 | 0.21 | 0.39 |
| *CD96* | NM_005816 | chr3 | 112741724 | 112742236 | -1635 | 0.69 | 0.35 | 0.34 |
| *CDA* | NM_001785 | chr1 | 20787075 | 20787367 | -809 | 0.71 | 0.33 | 0.39 |
| *CDC20B* | NM_001145734 | chr5 | 54507125 | 54507211 | -2408 | 0.85 | 0.48 | 0.38 |
| *CDC42EP5* | NM_145057 | chr19 | 59676722 | 59677228 | -741 | 0.51 | 0.15 | 0.36 |
| *CDCP2* | NM_201546 | chr1 | 54394055 | 54394518 | -2255 | 0.80 | 0.43 | 0.37 |
| *CDH20* | NM_031891 | chr18 | 57306819 | 57307313 | -1688 | 0.66 | 0.34 | 0.32 |
| *CDH5* | NM_001795 | chr16 | 64956659 | 64957141 | -1125 | 0.67 | 0.18 | 0.49 |
| *CDH9* | NM_016279 | chr5 | 27075921 | 27076450 | -1739 | 0.77 | 0.45 | 0.33 |
| *CDK3* | NM_001258 | chr17 | 71508275 | 71508765 | -61 | 0.77 | 0.39 | 0.38 |
| *CDK5RAP1* | NM_016082 | chr20 | 31453870 | 31454169 | -1021 | 0.86 | 0.24 | 0.61 |
| *CDKL5* | NM_001037343 | chrX | 18370129 | 18370502 | 51 | 0.72 | 0.30 | 0.42 |
| *CDR1* | NM_004065 | chrX | 139694738 | 139695235 | -597 | 0.89 | 0.53 | 0.36 |
| *CDY1* | NM_004680 | chrY | 24604909 | 24605420 | -1615 | 0.76 | 0.36 | 0.40 |
| *CDY1B* | NM_001003894 | chrY | 24604909 | 24605420 | -1615 | 0.76 | 0.36 | 0.40 |
| *CDYL* | NR_026590 | chr6 | 4650586 | 4651107 | -544 | 0.74 | 0.43 | 0.31 |
| *CEACAM1* | NM_001024912 | chr19 | 47724938 | 47725418 | -699 | 0.95 | 0.24 | 0.72 |
| *CEACAM16* | NM_001039213 | chr19 | 49893079 | 49893579 | -868 | 0.88 | 0.22 | 0.66 |
| *CEACAM20* | NM_001102597 | chr19 | 49726559 | 49727071 | -1427 | 0.52 | 0.17 | 0.34 |
| *CEACAM4* | NM_001817 | chr19 | 46825646 | 46826140 | -611 | 0.47 | 0.11 | 0.36 |
| *CEACAM5* | NM_004363 | chr19 | 46903487 | 46903979 | -636 | 0.74 | 0.23 | 0.51 |
| *CEACAM8* | NM_001816 | chr19 | 47791886 | 47792410 | -1226 | 0.63 | 0.29 | 0.33 |
| *CELP* | NR_001275 | chr9 | 134946769 | 134947275 | -724 | 0.51 | 0.20 | 0.31 |
| *CEND1* | NM_016564 | chr11 | 780489 | 781032 | -634 | 0.53 | 0.23 | 0.30 |
| *CENPC1* | NM_001812 | chr4 | 68096238 | 68096316 | -2426 | 0.85 | 0.53 | 0.32 |
| *CEP72* | NM_018140 | chr5 | 663513 | 664025 | -1635 | 0.64 | 0.25 | 0.39 |
| *CES3* | NM_024922 | chr16 | 65550542 | 65551081 | -1826 | 0.92 | 0.36 | 0.57 |
| *CES4* | NR_003276 | chr16 | 54349635 | 54350096 | -2145 | 0.76 | 0.37 | 0.40 |
| *CES8* | NM_173815 | chr16 | 65579443 | 65579984 | -419 | 0.83 | 0.46 | 0.36 |
| *CETP* | NM_000078 | chr16 | 55552967 | 55553466 | -118 | 0.70 | 0.36 | 0.35 |
| *CFB* | NM_001710 | chr6 | 32021575 | 32022065 | 121 | 0.56 | 0.13 | 0.43 |
| *CFHR1* | NM_002113 | chr1 | 195053067 | 195053528 | -2185 | 0.87 | 0.42 | 0.46 |
| *CFI* | NM_000204 | chr4 | 110944814 | 110945203 | -2224 | 0.64 | 0.14 | 0.50 |
| *CFP* | NM_001145252 | chrX | 47374757 | 47374959 | -545 | 0.74 | 0.43 | 0.32 |
| *CG030* | NR_026928 | chr13 | 31981387 | 31981928 | -125 | 0.89 | 0.40 | 0.49 |
| *CHCHD10* | NM_213720 | chr22 | 22440981 | 22441460 | -1079 | 0.80 | 0.48 | 0.32 |
| *CHCHD3* | NM_017812 | chr7 | 132417831 | 132418217 | -656 | 0.74 | 0.28 | 0.46 |
| *CHCHD8* | NM_016565 | chr11 | 73267248 | 73267741 | -1956 | 0.53 | 0.18 | 0.35 |
| *CHIC1* | NM_001039840 | chrX | 72697279 | 72697830 | -2153 | 0.76 | 0.29 | 0.47 |
| *CHRNA6* | NM_004198 | chr8 | 42743138 | 42743427 | -506 | 0.72 | 0.32 | 0.40 |
| *CHRNB1* | NM_000747 | chr17 | 7287239 | 7287741 | -1639 | 0.69 | 0.19 | 0.50 |
| *CHRNB3* | NM_000749 | chr8 | 42669852 | 42670376 | -1604 | 0.81 | 0.47 | 0.35 |
| *CHRNG* | NM_005199 | chr2 | 233112263 | 233112763 | -167 | 0.74 | 0.42 | 0.32 |
| *CHST14* | NM_130468 | chr15 | 38548328 | 38548870 | -1905 | 0.72 | 0.41 | 0.31 |
| *CHST9* | NM_031422 | chr18 | 23020262 | 23020684 | -1186 | 0.83 | 0.27 | 0.56 |
| *CILP* | NM_003613 | chr15 | 63290745 | 63291233 | -96 | 0.66 | 0.22 | 0.44 |
| *CKM* | NM_001824 | chr19 | 50518914 | 50519436 | -1201 | 0.46 | 0.14 | 0.32 |
| *CKMT2* | NM_001099735 | chr5 | 80564022 | 80564528 | -619 | 0.81 | 0.49 | 0.31 |
| *CLCN5* | NM_000084 | chrX | 49718158 | 49718673 | -538 | 0.85 | 0.53 | 0.32 |
| *CLCNKA* | NM_001042704 | chr1 | 16220851 | 16221355 | 31 | 0.83 | 0.45 | 0.38 |
| *CLCNKB* | NM_000085 | chr1 | 16240866 | 16241342 | -1729 | 0.75 | 0.28 | 0.47 |
| *CLDN1* | NM_021101 | chr3 | 191524366 | 191524673 | -1610 | 0.64 | 0.32 | 0.32 |
| *CLDN14* | NM_012130 | chr21 | 36762164 | 36762471 | -1722 | 0.79 | 0.41 | 0.37 |
| *CLDN19* | NM_001123395 | chr1 | 42979352 | 42979876 | -1102 | 0.60 | 0.25 | 0.35 |
| *CLDN4* | NM_001305 | chr7 | 72882236 | 72882734 | -643 | 0.64 | 0.21 | 0.43 |
| *CLDN9* | NM_020982 | chr16 | 3000041 | 3000366 | -2253 | 0.80 | 0.45 | 0.34 |
| *CLEC11A* | NM_002975 | chr19 | 55916525 | 55917025 | -1641 | 0.84 | 0.40 | 0.44 |
| *CLEC12A* | NM_138337 | chr12 | 10012873 | 10013346 | -2164 | 0.81 | 0.44 | 0.37 |
| *CLEC14A* | NM_175060 | chr14 | 37795480 | 37795978 | -404 | 0.77 | 0.47 | 0.30 |
| *CLEC17A* | NM_207390 | chr19 | 14554860 | 14555369 | 219 | 0.77 | 0.30 | 0.47 |
| *CLEC3B* | NM_003278 | chr3 | 45042524 | 45043066 | 33 | 0.42 | 0.11 | 0.31 |
| *CLEC4A* | NM_016184 | chr12 | 8165519 | 8166009 | -1730 | 0.88 | 0.15 | 0.74 |
| *CLEC4F* | NM_173535 | chr2 | 70901605 | 70902127 | -626 | 0.69 | 0.34 | 0.35 |
| *CLEC5A* | NM_013252 | chr7 | 141294223 | 141294703 | -1211 | 0.67 | 0.36 | 0.31 |
| *CLEC7A* | NM_022570 | chr12 | 10174322 | 10174827 | -439 | 0.74 | 0.32 | 0.42 |
| *CLEC9A* | NM_207345 | chr12 | 10072607 | 10073095 | -1691 | 0.75 | 0.36 | 0.39 |
| *CLIC3* | NM_004669 | chr9 | 139011684 | 139012208 | -1101 | 0.68 | 0.33 | 0.35 |
| *CLIP3* | NM_015526 | chr19 | 41216558 | 41217076 | -1202 | 0.85 | 0.44 | 0.41 |
| *CLK3* | NM_003992 | chr15 | 72686315 | 72686801 | -1207 | 0.91 | 0.57 | 0.35 |
| *CLLU1OS* | NM_001025232 | chr12 | 91347976 | 91348477 | -2171 | 0.76 | 0.43 | 0.33 |
| *CLPS* | NM_001832 | chr6 | 35873745 | 35874267 | -926 | 0.75 | 0.26 | 0.49 |
| *CLSTN3* | NM_014718 | chr12 | 7172846 | 7173364 | -1128 | 0.86 | 0.30 | 0.56 |
| *CLU* | NM_203339 | chr8 | 27525294 | 27525694 | -639 | 0.85 | 0.35 | 0.50 |
| *CLUAP1* | NM_024793 | chr16 | 3497578 | 3498114 | -2048 | 0.87 | 0.32 | 0.55 |
| *CMIP* | NM_030629 | chr16 | 80085993 | 80086466 | -224 | 0.75 | 0.31 | 0.43 |
| *CNDP2* | NM_018235 | chr18 | 70312623 | 70313169 | -1680 | 0.81 | 0.42 | 0.39 |
| *CNFN* | NM_032488 | chr19 | 47587110 | 47587624 | -1083 | 0.84 | 0.43 | 0.41 |
| *CNGB1* | NM_001135639 | chr16 | 56564234 | 56564510 | -1851 | 0.68 | 0.32 | 0.36 |
| *CNIH2* | NM_182553 | chr11 | 65800280 | 65800814 | -1724 | 0.70 | 0.40 | 0.30 |
| *CNTNAP4* | NM_138994 | chr16 | 74898796 | 74899317 | -2178 | 0.74 | 0.33 | 0.41 |
| *COL17A1* | NM_000494 | chr10 | 105836010 | 105836496 | -625 | 0.76 | 0.44 | 0.33 |
| *COL18A1* | NM_030582 | chr21 | 45697871 | 45698385 | -1723 | 0.74 | 0.38 | 0.36 |
| *COL20A1* | NM_020882 | chr20 | 61394336 | 61394840 | -394 | 0.80 | 0.45 | 0.35 |
| *COL23A1* | NM_173465 | chr5 | 177952219 | 177952640 | -2267 | 0.52 | 0.16 | 0.36 |
| *COL24A1* | NM_152890 | chr1 | 86396897 | 86397183 | -2331 | 0.76 | 0.40 | 0.36 |
| *COL7A1* | NM_000094 | chr3 | 48609460 | 48609936 | -2101 | 0.84 | 0.25 | 0.60 |
| *COL9A2* | NM_001852 | chr1 | 40555865 | 40556378 | -595 | 0.75 | 0.19 | 0.56 |
| *COMMD1* | NM_152516 | chr2 | 61985091 | 61985612 | -954 | 0.60 | 0.18 | 0.41 |
| *COMMD6* | NM_203495 | chr13 | 75010942 | 75011358 | -1158 | 0.57 | 0.22 | 0.36 |
| *COPS4* | NM_016129 | chr4 | 84173164 | 84173704 | -1828 | 0.87 | 0.54 | 0.33 |
| *COPS7B* | NM_022730 | chr2 | 232357138 | 232357613 | -2029 | 0.74 | 0.43 | 0.31 |
| *CORO6* | NM_032854 | chr17 | 24972570 | 24973060 | -195 | 0.61 | 0.30 | 0.32 |
| *COX5A* | NM_004255 | chr15 | 73019120 | 73019321 | -1672 | 0.81 | 0.48 | 0.33 |
| *COX7B2* | NM_130902 | chr4 | 46607710 | 46608248 | -1970 | 0.51 | 0.21 | 0.30 |
| *CPB1* | NM_001871 | chr3 | 150026344 | 150026847 | -1681 | 0.61 | 0.31 | 0.30 |
| *CPB2* | NM_001872 | chr13 | 45578482 | 45579000 | -1529 | 0.84 | 0.47 | 0.37 |
| *CPLX3* | NM_001030005 | chr15 | 72905034 | 72905530 | -721 | 0.73 | 0.39 | 0.34 |
| *CPM* | NM_001874 | chr12 | 67644734 | 67645245 | -1702 | 0.87 | 0.45 | 0.42 |
| *CPN1* | NM_001308 | chr10 | 101832927 | 101833426 | -1544 | 0.75 | 0.33 | 0.42 |
| *CPNE3* | NM_003909 | chr8 | 87593784 | 87594334 | -1712 | 0.67 | 0.35 | 0.31 |
| *CPNE6* | NM_006032 | chr14 | 23609704 | 23610210 | -638 | 0.64 | 0.31 | 0.33 |
| *CPSF4L* | NM_001129885 | chr17 | 68771050 | 68771528 | -1675 | 0.84 | 0.30 | 0.54 |
| *CPXCR1* | NM_033048 | chrX | 87886465 | 87887014 | -2141 | 0.77 | 0.45 | 0.32 |
| *CPXM2* | NM_198148 | chr10 | 125643188 | 125643701 | -1954 | 0.68 | 0.38 | 0.30 |
| *CR1L* | NM_175710 | chr1 | 205883123 | 205883597 | -1720 | 0.94 | 0.52 | 0.42 |
| *CRABP1* | NM_004378 | chr15 | 76417836 | 76418356 | -1624 | 0.77 | 0.36 | 0.41 |
| *CRB2* | NM_173689 | chr9 | 125157902 | 125158378 | -128 | 0.68 | 0.18 | 0.50 |
| *CRCP* | NM_001040647 | chr7 | 65215348 | 65215869 | -1630 | 0.73 | 0.42 | 0.32 |
| *CREB3L3* | NM_032607 | chr19 | 4103793 | 4104087 | -688 | 0.69 | 0.27 | 0.41 |
| *CREG2* | NM_153836 | chr2 | 101371333 | 101371842 | -1190 | 0.70 | 0.40 | 0.30 |
| *CRHR2* | NM_001883 | chr7 | 30689448 | 30689954 | -1036 | 0.84 | 0.52 | 0.32 |
| *CROCCL1* | NR_026752 | chr1 | 16830827 | 16831331 | -1091 | 0.82 | 0.26 | 0.55 |
| *CRYAA* | NM_000394 | chr21 | 43460895 | 43461343 | -1090 | 0.66 | 0.34 | 0.32 |
| *CRYAB* | NM_001885 | chr11 | 111287198 | 111287688 | 240 | 0.81 | 0.48 | 0.33 |
| *CSAG1* | NM_001102576 | chrX | 151651910 | 151652422 | -1717 | 0.82 | 0.47 | 0.35 |
| *CSF1R* | NM_005211 | chr5 | 149473511 | 149473995 | -625 | 0.89 | 0.57 | 0.32 |
| *CSF2RA* | NM_006140 | chrX | 1345784 | 1346244 | -1686 | 0.79 | 0.43 | 0.36 |
| *CSF3* | NM_000759 | chr17 | 35424194 | 35424694 | -769 | 0.60 | 0.30 | 0.30 |
| *CSGALNACT1* | NM_018371 | chr8 | 19585219 | 19585661 | -1066 | 0.51 | 0.20 | 0.31 |
| *CSH2* | NM_020991 | chr17 | 59305282 | 59305560 | -600 | 0.65 | 0.07 | 0.58 |
| *CSHL1* | NM_001318 | chr17 | 59342690 | 59343216 | -603 | 0.61 | 0.26 | 0.35 |
| *CSN1S1* | NM_001025104 | chr4 | 70830097 | 70830628 | -1024 | 0.76 | 0.41 | 0.35 |
| *CSN3* | NM_005212 | chr4 | 71142035 | 71142560 | -623 | 0.69 | 0.07 | 0.62 |
| *CSPG4* | NM_001897 | chr15 | 73794534 | 73794640 | -2343 | 0.69 | 0.28 | 0.42 |
| *CSPG4LYP1* | NR_001554 | chrY | 26036501 | 26037021 | -1681 | 0.84 | 0.48 | 0.36 |
| *CSPG4LYP2* | NR_002194 | chrY | 26036501 | 26037021 | -1681 | 0.84 | 0.48 | 0.36 |
| *CSRP1* | NM_001144773 | chr1 | 199743474 | 199743981 | -717 | 0.72 | 0.34 | 0.38 |
| *CSRP2BP* | NM_020536 | chr20 | 18064610 | 18065109 | -1666 | 0.89 | 0.44 | 0.45 |
| *CST2* | NM_001322 | chr20 | 23757652 | 23757752 | -2390 | 0.64 | 0.19 | 0.45 |
| *CST3* | NM_000099 | chr20 | 23567950 | 23568458 | -1630 | 0.77 | 0.43 | 0.35 |
| *CST5* | NM_001900 | chr20 | 23810219 | 23810741 | -2100 | 0.61 | 0.26 | 0.35 |
| *CSTB* | NM_000100 | chr21 | 44023027 | 44023151 | -2402 | 0.80 | 0.39 | 0.41 |
| *CSTL1* | NM_138283 | chr20 | 23368277 | 23368766 | 200 | 0.86 | 0.52 | 0.34 |
| *CT45A1* | NM_001017417 | chrX | 134673883 | 134674257 | -780 | 0.77 | 0.47 | 0.30 |
| *CTCFL* | NM_080618 | chr20 | 55534024 | 55534524 | -714 | 0.85 | 0.26 | 0.59 |
| *CTRB1* | NM_001906 | chr16 | 73810221 | 73810739 | 96 | 0.72 | 0.42 | 0.30 |
| *CTRC* | NM_007272 | chr1 | 15635087 | 15635262 | -2349 | 0.82 | 0.42 | 0.40 |
| *CTRL* | NM_001907 | chr16 | 66525473 | 66525667 | -2304 | 0.67 | 0.20 | 0.47 |
| *CTSC* | NM_001114173 | chr11 | 87711930 | 87712459 | -1605 | 0.82 | 0.26 | 0.56 |
| *CUEDC1* | NM_017949 | chr17 | 53336939 | 53337459 | -1450 | 0.75 | 0.40 | 0.35 |
| *CUL4B* | NM_001079872 | chrX | 119580227 | 119580709 | -1623 | 0.78 | 0.17 | 0.60 |
| *CUZD1* | NM_022034 | chr10 | 124596121 | 124596620 | -689 | 0.62 | 0.13 | 0.49 |
| *CX3CL1* | NM_002996 | chr16 | 55962997 | 55963489 | -671 | 0.70 | 0.32 | 0.37 |
| *CXCL1* | NM_001511 | chr4 | 74951581 | 74952124 | -2119 | 0.75 | 0.40 | 0.35 |
| *CXCR3* | NM_001142797 | chrX | 70754957 | 70755477 | -125 | 0.66 | 0.20 | 0.46 |
| *CXorf42* | NR_027131 | chrX | 119264248 | 119264754 | -1351 | 0.69 | 0.13 | 0.56 |
| *CXorf57* | NM_018015 | chrX | 105740854 | 105741358 | -709 | 0.68 | 0.25 | 0.43 |
| *CXorf59* | NM_173695 | chrX | 35974402 | 35974939 | -302 | 0.88 | 0.47 | 0.41 |
| *CXorf61* | NM_001017978 | chrX | 115509991 | 115510502 | -2081 | 0.85 | 0.56 | 0.30 |
| *CYB561D1* | NM_001134400 | chr1 | 109836690 | 109837208 | -1274 | 0.70 | 0.37 | 0.32 |
| *CYB5R4* | NM_016230 | chr6 | 84623669 | 84624212 | -2147 | 0.78 | 0.35 | 0.43 |
| *CYBB* | NM_000397 | chrX | 37522352 | 37522897 | -1588 | 0.99 | 0.47 | 0.52 |
| *CYFIP2* | NM_001037332 | chr5 | 156624288 | 156624400 | -1420 | 0.91 | 0.54 | 0.37 |
| *CYP11A1* | NM_000781 | chr15 | 72447466 | 72447945 | -571 | 0.91 | 0.22 | 0.69 |
| *CYP21A2* | NM_000500 | chr6 | 32112092 | 32112580 | -1724 | 0.65 | 0.10 | 0.55 |
| *CYP2A13* | NM_000766 | chr19 | 46284241 | 46284719 | -1727 | 0.49 | 0.08 | 0.41 |
| *CYP2D6* | NM_000106 | chr22 | 40856782 | 40857270 | -199 | 0.69 | 0.20 | 0.49 |
| *CYP2D7P1* | NR_002570 | chr22 | 40870459 | 40870971 | -196 | 0.69 | 0.29 | 0.40 |
| *CYP3A43* | NM_022820 | chr7 | 99261306 | 99261848 | -1994 | 0.90 | 0.52 | 0.38 |
| *CYP4A22* | NM_001010969 | chr1 | 47374213 | 47374751 | -1211 | 0.91 | 0.61 | 0.30 |
| *CYP4F11* | NM_001128932 | chr19 | 15906539 | 15907021 | -104 | 0.77 | 0.42 | 0.35 |
| *CYP4F12* | NM_023944 | chr19 | 15644456 | 15644973 | -170 | 0.68 | 0.20 | 0.49 |
| *CYP4F3* | NM_000896 | chr19 | 15612324 | 15612838 | -125 | 0.84 | 0.43 | 0.41 |
| *CYP4F8* | NM_007253 | chr19 | 15586506 | 15586988 | -281 | 0.83 | 0.39 | 0.44 |
| *CYP4X1* | NM_178033 | chr1 | 47259424 | 47259970 | -2129 | 0.89 | 0.42 | 0.47 |
| *CYP8B1* | NM_004391 | chr3 | 42893577 | 42894110 | -1206 | 0.67 | 0.21 | 0.46 |
| *CYSLTR1* | NM_006639 | chrX | 77470188 | 77470692 | -697 | 0.87 | 0.41 | 0.47 |
| *CYYR1* | NM_052954 | chr21 | 26868305 | 26868820 | -1110 | 0.82 | 0.40 | 0.42 |
| *D2HGDH* | NM_152783 | chr2_random | 149998 | 150196 | -1064 | 0.74 | 0.30 | 0.43 |
| *DAB2IP* | NM_138709 | chr9 | 123542451 | 123542920 | -2198 | 0.63 | 0.22 | 0.41 |
| *DAGLB* | NM_001142936 | chr7 | 6455343 | 6455633 | -1320 | 0.68 | 0.35 | 0.34 |
| *DAO* | NM_001917 | chr12 | 107797692 | 107798170 | -54 | 0.73 | 0.26 | 0.47 |
| *DAZ1* | NM_004081 | chrY | 23755371 | 23755891 | -1004 | 0.80 | 0.48 | 0.33 |
| *DAZ2* | NM_001005785 | chrY | 23774038 | 23774529 | -725 | 0.82 | 0.47 | 0.35 |
| *DAZ3* | NM_020364 | chrY | 23774038 | 23774529 | -806 | 0.82 | 0.47 | 0.35 |
| *DAZ4* | NM_020420 | chrY | 23774038 | 23774529 | -781 | 0.82 | 0.47 | 0.35 |
| *DBH* | NM_000787 | chr9 | 135489838 | 135490342 | -1215 | 0.78 | 0.38 | 0.40 |
| *DBNDD1* | NM_024043 | chr16 | 88605288 | 88605774 | -1501 | 0.59 | 0.25 | 0.34 |
| *DCDC2* | NM_016356 | chr6 | 24466201 | 24466695 | -189 | 0.73 | 0.35 | 0.38 |
| *DCDC2B* | NM_001099434 | chr1 | 32445506 | 32445990 | -1533 | 0.80 | 0.20 | 0.60 |
| *DCP1A* | NM_018403 | chr3 | 53359017 | 53359151 | -2407 | 0.85 | 0.54 | 0.30 |
| *DDEF1IT1* | NR_002765 | chr8 | 131379934 | 131380427 | -2219 | 0.74 | 0.33 | 0.41 |
| *DDOST* | NM_005216 | chr1 | 20861586 | 20861974 | -1156 | 0.74 | 0.38 | 0.36 |
| *DDR1* | NM_013994 | chr6 | 30962003 | 30962474 | -2204 | 0.80 | 0.43 | 0.38 |
| *DDX56* | NM_019082 | chr7 | 44582217 | 44582637 | -1765 | 0.74 | 0.27 | 0.47 |
| *DEF6* | NM_022047 | chr6 | 35371603 | 35372093 | -1724 | 0.91 | 0.35 | 0.57 |
| *DEFA1* | NM_004084 | chr8 | 6846111 | 6846587 | -2227 | 0.62 | 0.22 | 0.39 |
| *DEFB112* | NM_001037498 | chr6 | 50124868 | 50125397 | -809 | 0.79 | 0.43 | 0.37 |
| *DEFB119* | NM_153289 | chr20 | 29441920 | 29442415 | -100 | 0.84 | 0.35 | 0.48 |
| *DEGS2* | NM_206918 | chr14 | 99696546 | 99697040 | -1028 | 0.76 | 0.40 | 0.36 |
| *DENND4C* | NM_017925 | chr9 | 19280408 | 19280894 | -97 | 0.71 | 0.31 | 0.40 |
| *DGKZ* | NM_001105540 | chr11 | 46339073 | 46339566 | -400 | 0.78 | 0.40 | 0.38 |
| *DHRSX* | NM_145177 | chrY | 2429782 | 2430276 | -1014 | 0.76 | 0.32 | 0.44 |
| *DHX32* | NM_018180 | chr10 | 127561663 | 127562140 | -2027 | 0.73 | 0.21 | 0.52 |
| *DHX58* | NM_024119 | chr17 | 37518724 | 37519241 | -705 | 0.76 | 0.34 | 0.41 |
| *DICER1* | NM_030621 | chr14 | 94678672 | 94679173 | -1114 | 0.82 | 0.22 | 0.60 |
| *DIO1* | NM_000792 | chr1 | 54131050 | 54131545 | -1150 | 0.76 | 0.41 | 0.34 |
| *DIXDC1* | NM_033425 | chr11 | 111352403 | 111352876 | -602 | 0.52 | 0.19 | 0.33 |
| *DKFZP434K028* | NR_026882 | chr11 | 61281651 | 61282151 | -189 | 0.65 | 0.25 | 0.40 |
| *DKFZp434L192* | NR_026929 | chr7 | 56528994 | 56529529 | -2147 | 0.85 | 0.34 | 0.51 |
| *DKFZp686O24166* | NR_026750 | chr11 | 17329191 | 17329716 | -438 | 0.61 | 0.25 | 0.35 |
| *DLC1* | NM_024767 | chr8 | 13419146 | 13419254 | -2434 | 0.83 | 0.26 | 0.57 |
| *DMAP1* | NM_001034023 | chr1 | 44450727 | 44451235 | -730 | 0.53 | 0.17 | 0.35 |
| *DMD* | NM_004007 | chrX | 32950366 | 32950682 | -2286 | 0.91 | 0.50 | 0.41 |
| *DMPK* | NM_001081560 | chr19 | 50978864 | 50979362 | -1458 | 0.83 | 0.28 | 0.55 |
| *DMXL2* | NM_015263 | chr15 | 49704030 | 49704512 | -2012 | 0.74 | 0.43 | 0.31 |
| *DNAH1* | NM_015512 | chr3 | 52323483 | 52323987 | -1639 | 0.70 | 0.19 | 0.51 |
| *DNAH11* | NM_003777 | chr7 | 21547896 | 21548403 | -1207 | 0.77 | 0.40 | 0.37 |
| *DNAH14* | NM_001145154 | chr1 | 223183245 | 223183783 | -464 | 0.83 | 0.42 | 0.41 |
| *DNAJB13* | NM_153614 | chr11 | 73338046 | 73338348 | -814 | 0.48 | 0.10 | 0.38 |
| *DNAJB8* | NM_153330 | chr3 | 129670244 | 129670736 | -1709 | 0.83 | 0.51 | 0.32 |
| *DNAJC4* | NM_005528 | chr11 | 63750971 | 63751471 | -3107 | 0.63 | 0.26 | 0.37 |
| *DNALI1* | NM_003462 | chr1 | 37792451 | 37792983 | -2389 | 0.62 | 0.31 | 0.31 |
| *DNASE1L1* | NM_001009932 | chrX | 153295433 | 153295914 | -2052 | 0.78 | 0.47 | 0.32 |
| *DNASE2B* | NM_021233 | chr1 | 84634367 | 84634539 | -2349 | 0.51 | 0.08 | 0.43 |
| *DND1* | NM_194249 | chr5 | 140035630 | 140035841 | -2380 | 0.85 | 0.44 | 0.41 |
| *DNHD1* | NM_144666 | chr11 | 6472683 | 6473156 | -2181 | 0.92 | 0.45 | 0.47 |
| *DNMT3B* | NM_175850 | chr20 | 30830492 | 30830988 | -578 | 0.87 | 0.49 | 0.37 |
| *DNMT3L* | NM_013369 | chr21 | 44506471 | 44506969 | -193 | 0.74 | 0.32 | 0.42 |
| *DOK3* | NM_001144875 | chr5 | 176870853 | 176871349 | -1068 | 0.70 | 0.30 | 0.40 |
| *DPAGT1* | NM_001382 | chr11 | 118480928 | 118481236 | -3087 | 0.85 | 0.55 | 0.31 |
| *DPEP2* | NM_022355 | chr16 | 66591782 | 66592204 | -1128 | 0.78 | 0.48 | 0.30 |
| *DPP6* | NM_001936 | chr7 | 153631804 | 153632306 | -1224 | 0.87 | 0.57 | 0.30 |
| *DRGX* | NM_001080520 | chr10 | 50272301 | 50272387 | -2431 | 0.49 | 0.18 | 0.31 |
| *DRP2* | NM_001939 | chrX | 100360622 | 100361144 | -705 | 0.74 | 0.44 | 0.30 |
| *DSCAM* | NM_001389 | chr21 | 41142759 | 41143258 | -2099 | 0.84 | 0.38 | 0.45 |
| *DSCR9* | NR_026719 | chr21 | 37501902 | 37502408 | -669 | 0.69 | 0.30 | 0.39 |
| *DSE* | NM_013352 | chr6 | 116797850 | 116798338 | -708 | 0.80 | 0.24 | 0.56 |
| *DSN1* | NM_001145315 | chr20 | 34836331 | 34836823 | -933 | 0.62 | 0.31 | 0.31 |
| *DTX1* | NM_004416 | chr12 | 111977629 | 111978176 | -2141 | 0.78 | 0.37 | 0.41 |
| *DUSP10* | NM_144728 | chr1 | 219979287 | 219979768 | -2102 | 0.60 | 0.26 | 0.34 |
| *DUSP13* | NM_016364 | chr10 | 76529603 | 76530115 | -605 | 0.62 | 0.10 | 0.52 |
| *DUSP15* | NM_001012644 | chr20 | 29923378 | 29923684 | -2069 | 0.89 | 0.39 | 0.51 |
| *DUSP26* | NM_024025 | chr8 | 33577335 | 33577532 | -452 | 0.54 | 0.24 | 0.30 |
| *DYTN* | NM_001093730 | chr2 | 207291834 | 207292113 | -608 | 0.79 | 0.39 | 0.40 |
| *ECE1* | NM_001113347 | chr1 | 21479232 | 21479734 | -713 | 0.85 | 0.46 | 0.40 |
| *EDC4* | NM_014329 | chr16 | 66463012 | 66463508 | -1239 | 0.84 | 0.52 | 0.32 |
| *EEF1AL7* | NR_003586 | chr4 | 106623857 | 106624378 | -1193 | 0.84 | 0.44 | 0.40 |
| *EFCAB4A* | NM_173584 | chr11 | 815394 | 815914 | -1930 | 0.82 | 0.36 | 0.45 |
| *EFEMP1* | NM_001039348 | chr2 | 56006501 | 56006932 | -2280 | 0.92 | 0.62 | 0.31 |
| *EGFL8* | NM_030652 | chr6 | 32239797 | 32240309 | -329 | 0.81 | 0.38 | 0.43 |
| *EHD2* | NM_014601 | chr19 | 52907430 | 52907945 | -724 | 0.63 | 0.32 | 0.31 |
| *EIF1AX* | NM_001412 | chrX | 20070845 | 20071299 | -1185 | 0.76 | 0.46 | 0.31 |
| *EIF1AY* | NM_004681 | chrY | 21145150 | 21145642 | -1602 | 0.85 | 0.48 | 0.37 |
| *EIF1B* | NM_005875 | chr3 | 40324842 | 40325344 | -1083 | 0.87 | 0.57 | 0.30 |
| *EIF2B1* | NM_001414 | chr12 | 122685624 | 122685718 | -1471 | 0.61 | 0.31 | 0.30 |
| *EIF3K* | NM_013234 | chr19 | 43800011 | 43800495 | -1308 | 0.62 | 0.27 | 0.35 |
| *EIF4G1* | NM_004953 | chr3 | 185520516 | 185521016 | -190 | 0.68 | 0.32 | 0.36 |
| *ELF1* | NM_001145353 | chr13 | 40454862 | 40455403 | -714 | 0.88 | 0.57 | 0.31 |
| *ELF3* | NM_001114309 | chr1 | 200243874 | 200244343 | -2203 | 0.84 | 0.48 | 0.37 |
| *ELK1* | NM_001114123 | chrX | 47395810 | 47396288 | -1102 | 0.71 | 0.34 | 0.38 |
| *ELMO3* | NM_024712 | chr16 | 65788638 | 65789158 | -1630 | 0.82 | 0.36 | 0.46 |
| *ELN* | NM_000501 | chr7 | 73078874 | 73079385 | -1232 | 0.61 | 0.29 | 0.32 |
| *EML4* | NM_001145076 | chr2 | 42248014 | 42248505 | -1733 | 0.97 | 0.59 | 0.38 |
| *ENG* | NM_000118 | chr9 | 129657894 | 129658416 | -1287 | 0.86 | 0.40 | 0.46 |
| *ENPP2* | NM_001040092 | chr8 | 120721167 | 120721664 | -1128 | 0.85 | 0.50 | 0.34 |
| *ENTPD3* | NM_001248 | chr3 | 40402303 | 40402851 | -1099 | 0.76 | 0.46 | 0.30 |
| *EPB49* | NM_001114138 | chr8 | 21971685 | 21972173 | -702 | 0.67 | 0.28 | 0.39 |
| *EPHX2* | NM_001979 | chr8 | 27403212 | 27403720 | -1095 | 0.77 | 0.31 | 0.46 |
| *EPHX4* | NM_173567 | chr1 | 92265680 | 92265734 | -2413 | 0.80 | 0.45 | 0.35 |
| *EPPK1* | NM_031308 | chr8 | 145020896 | 145021386 | -1719 | 0.68 | 0.38 | 0.30 |
| *EPS8L2* | NM_022772 | chr11 | 693694 | 694152 | -2196 | 0.66 | 0.31 | 0.36 |
| *EPS8L3* | NM_024526 | chr1 | 110107950 | 110108426 | -101 | 0.45 | 0.13 | 0.32 |
| *EPX* | NM_000502 | chr17 | 53623254 | 53623752 | -1617 | 0.89 | 0.46 | 0.43 |
| *ERCC6L* | NM_017669 | chrX | 71377546 | 71378067 | -2223 | 0.91 | 0.60 | 0.31 |
| *ERMAP* | NM_018538 | chr1 | 43061978 | 43062268 | -1712 | 0.74 | 0.41 | 0.32 |
| *ESR1* | NM_001122742 | chr6 | 152052474 | 152052958 | -607 | 0.88 | 0.56 | 0.32 |
| *ESSPL* | NM_183375 | chr4 | 152416734 | 152417267 | -773 | 0.87 | 0.42 | 0.46 |
| *ETNK1* | NM_001039481 | chr12 | 22666943 | 22667383 | -2179 | 0.70 | 0.35 | 0.35 |
| *ETV7* | NM_016135 | chr6 | 36464892 | 36465414 | -1708 | 0.68 | 0.38 | 0.30 |
| *EXPH5* | NM_001144765 | chr11 | 107914006 | 107914489 | -103 | 0.90 | 0.47 | 0.43 |
| *EXTL1* | NM_004455 | chr1 | 26220715 | 26221205 | 103 | 0.76 | 0.42 | 0.34 |
| *EYS* | NM_001142800 | chr6 | 66473706 | 66474204 | -116 | 0.73 | 0.26 | 0.48 |
| *F12* | NM_000505 | chr5 | 176769887 | 176770326 | -923 | 0.88 | 0.54 | 0.34 |
| *F13B* | NM_001994 | chr1 | 195304420 | 195304760 | -1570 | 0.79 | 0.32 | 0.47 |
| *F2* | NM_000506 | chr11 | 46696248 | 46696679 | -854 | 0.53 | 0.12 | 0.41 |
| *FABP1* | NM_001443 | chr2 | 88209906 | 88210382 | -1451 | 0.88 | 0.17 | 0.71 |
| *FAHD2B* | NM_199336 | chr2 | 97125169 | 97125459 | -1005 | 0.71 | 0.33 | 0.38 |
| *FAIM* | NM_001033030 | chr3 | 139808437 | 139808939 | -1543 | 0.85 | 0.31 | 0.54 |
| *FAM107A* | NM_001076778 | chr3 | 58540424 | 58540950 | -2156 | 0.59 | 0.25 | 0.34 |
| *FAM109A* | NM_144671 | chr12 | 110292229 | 110292676 | -1144 | 0.75 | 0.45 | 0.30 |
| *FAM110A* | NM_031424 | chr20 | 772516 | 773018 | -517 | 0.77 | 0.28 | 0.49 |
| *FAM111B* | NM_001142703 | chr11 | 58631120 | 58631641 | 147 | 0.53 | 0.21 | 0.31 |
| *FAM124B* | NM_001122779 | chr2 | 224977142 | 224977426 | -2329 | 0.80 | 0.48 | 0.33 |
| *FAM129B* | NM_001035534 | chr9 | 129381328 | 129381816 | -483 | 0.60 | 0.19 | 0.41 |
| *FAM13A* | NM_001015045 | chr4 | 89963792 | 89964319 | -630 | 0.68 | 0.29 | 0.39 |
| *FAM13C* | NM_001001971 | chr10 | 60793264 | 60793778 | -1163 | 0.66 | 0.22 | 0.44 |
| *FAM151B* | NM_205548 | chr5 | 79817860 | 79818362 | -1444 | 0.86 | 0.53 | 0.33 |
| *FAM166A* | NM_001001710 | chr9 | 139263659 | 139264162 | -1867 | 0.81 | 0.46 | 0.35 |
| *FAM178B* | NM_016490 | chr2 | 96927420 | 96927816 | -60 | 0.79 | 0.31 | 0.47 |
| *FAM181A* | NM_138344 | chr14 | 93453675 | 93454178 | -1083 | 0.64 | 0.34 | 0.30 |
| *FAM22F* | NM_017561 | chr9 | 96130690 | 96131200 | -198 | 0.64 | 0.18 | 0.46 |
| *FAM25A* | NM_001146157 | chr10 | 88769121 | 88769599 | -675 | 0.68 | 0.20 | 0.48 |
| *FAM25B* | NM_001137556 | chr10 | 48878139 | 48878627 | -552 | 0.59 | 0.20 | 0.38 |
| *FAM25C* | NM_001137548 | chr10 | 48878139 | 48878627 | -552 | 0.59 | 0.20 | 0.38 |
| *FAM25G* | NM_001137549 | chr10 | 48878139 | 48878627 | -552 | 0.59 | 0.20 | 0.38 |
| *FAM27L* | NM_203392 | chr17 | 21747061 | 21747531 | -2200 | 0.75 | 0.22 | 0.53 |
| *FAM45A* | NM_207009 | chr10 | 120852408 | 120852588 | -1102 | 0.85 | 0.53 | 0.32 |
| *FAM45B* | NR_027141 | chr10 | 120852408 | 120852588 | -1120 | 0.85 | 0.53 | 0.32 |
| *FAM47C* | NM_001013736 | chrX | 36935038 | 36935550 | -1096 | 0.88 | 0.48 | 0.40 |
| *FAM57B* | NM_031478 | chr16 | 29951040 | 29951433 | -1549 | 0.77 | 0.46 | 0.31 |
| *FAM65A* | NM_024519 | chr16 | 66118371 | 66118859 | -1639 | 0.69 | 0.27 | 0.42 |
| *FAM65C* | NM_080829 | chr20 | 48688520 | 48688901 | -1877 | 0.65 | 0.29 | 0.37 |
| *FAM71A* | NM_153606 | chr1 | 210864422 | 210864944 | 245 | 0.72 | 0.41 | 0.31 |
| *FAM71B* | NM_130899 | chr5 | 156526299 | 156526769 | -677 | 0.90 | 0.26 | 0.64 |
| *FAM71E2* | NM_001145402 | chr19 | 60568243 | 60568777 | -2078 | 0.69 | 0.35 | 0.34 |
| *FAM71F1* | NM_032599 | chr7 | 128141284 | 128141596 | -1238 | 0.87 | 0.45 | 0.42 |
| *FAM83C* | NM_178468 | chr20 | 33345004 | 33345508 | -1617 | 0.74 | 0.06 | 0.68 |
| *FAM83E* | NM_017708 | chr19 | 53808950 | 53809466 | -702 | 0.79 | 0.23 | 0.56 |
| *FAM83F* | NM_138435 | chr22 | 38719856 | 38720356 | -792 | 0.72 | 0.38 | 0.33 |
| *FAM99B* | NR_026642 | chr11 | 1663795 | 1664297 | -611 | 0.77 | 0.31 | 0.46 |
| *FAM9C* | NM_174901 | chrX | 12974428 | 12974932 | -1959 | 0.95 | 0.64 | 0.32 |
| *FASLG* | NM_000639 | chr1 | 170893447 | 170893666 | -1250 | 0.88 | 0.39 | 0.49 |
| *FATE1* | NM_033085 | chrX | 150632728 | 150633275 | -2161 | 0.82 | 0.36 | 0.47 |
| *FBLIM1* | NM_001024215 | chr1 | 15963557 | 15964063 | 230 | 0.60 | 0.15 | 0.45 |
| *FBXL22* | NM_203373 | chr15 | 61675679 | 61676185 | -716 | 0.67 | 0.20 | 0.47 |
| *FBXO3* | NM_012175 | chr11 | 33753949 | 33754426 | -1540 | 0.75 | 0.39 | 0.36 |
| *FBXO41* | NM_001080410 | chr2 | 73352704 | 73353214 | -1408 | 0.83 | 0.26 | 0.57 |
| *FBXO6* | NM_018438 | chr1 | 11644747 | 11645245 | -1740 | 0.82 | 0.51 | 0.31 |
| *FBXO9* | NM_012347 | chr6 | 53043146 | 53043646 | -333 | 0.46 | 0.05 | 0.41 |
| *FCGBP* | NM_003890 | chr19 | 45133321 | 45133817 | -1196 | 0.80 | 0.26 | 0.54 |
| *FCGR2B* | NM_001002273 | chr1 | 159899269 | 159899771 | -43 | 0.79 | 0.41 | 0.39 |
| *FCHO1* | NM_015122 | chr19 | 17721458 | 17721966 | -1623 | 0.70 | 0.22 | 0.48 |
| *FCN2* | NM_004108 | chr9 | 136912050 | 136912560 | -173 | 0.69 | 0.39 | 0.30 |
| *FCRLB* | NM_001002901 | chr1 | 159958737 | 159959233 | -95 | 0.77 | 0.47 | 0.30 |
| *FDX1* | NM_004109 | chr11 | 109803866 | 109804386 | -1677 | 0.83 | 0.52 | 0.31 |
| *FDX1L* | NM_001031734 | chr19 | 10288133 | 10288637 | -694 | 0.69 | 0.39 | 0.30 |
| *FES* | NM_001143783 | chr15 | 89227798 | 89228310 | -1225 | 0.68 | 0.25 | 0.43 |
| *FETUB* | NM_014375 | chr3 | 187838407 | 187838879 | -2199 | 0.86 | 0.50 | 0.36 |
| *FGB* | NM_005141 | chr4 | 155702648 | 155703146 | -684 | 0.53 | 0.22 | 0.31 |
| *FGD4* | NM_139241 | chr12 | 32545094 | 32545620 | -950 | 0.65 | 0.25 | 0.41 |
| *FGF1* | NM_000800 | chr5 | 142045700 | 142046200 | -113 | 0.73 | 0.36 | 0.37 |
| *FGF13* | NM_001139502 | chrX | 137778411 | 137778899 | -1272 | 0.85 | 0.43 | 0.42 |
| *FGF17* | NM_003867 | chr8 | 21954882 | 21955430 | -1217 | 0.74 | 0.31 | 0.43 |
| *FGFR4* | NM_022963 | chr5 | 176449080 | 176449572 | 170 | 0.67 | 0.33 | 0.34 |
| *FGR* | NM_001042747 | chr1 | 27825053 | 27825578 | 22 | 0.63 | 0.19 | 0.43 |
| *FH* | NM_000143 | chr1 | 239750621 | 239751116 | -1191 | 0.81 | 0.46 | 0.35 |
| *FHL2* | NM_201557 | chr2 | 105422605 | 105423160 | -1220 | 0.81 | 0.49 | 0.32 |
| *FHL5* | NM_020482 | chr6 | 97116456 | 97116969 | -442 | 0.70 | 0.28 | 0.42 |
| *FILIP1L* | NM_014890 | chr3 | 101078083 | 101078609 | -610 | 0.86 | 0.41 | 0.45 |
| *FKBP5* | NM_001145775 | chr6 | 35805131 | 35805635 | -1045 | 0.92 | 0.36 | 0.55 |
| *FKBP9L* | NR_003949 | chr7 | 55741846 | 55742246 | -2292 | 0.53 | 0.22 | 0.31 |
| *FLJ22167* | NM_001077416 | chr16 | 74148085 | 74148575 | -693 | 0.78 | 0.45 | 0.33 |
| *FLJ25006* | NM_144610 | chr17 | 23967067 | 23967554 | -1972 | 0.81 | 0.41 | 0.40 |
| *FLJ32063* | NR_026830 | chr2 | 200040579 | 200041099 | -226 | 0.67 | 0.26 | 0.40 |
| *FLJ37201* | NR_026835 | chr10 | 91448257 | 91448775 | -851 | 0.65 | 0.26 | 0.39 |
| *FLJ39609* | NR_026874 | chr1 | 847024 | 847120 | -2392 | 0.60 | 0.23 | 0.36 |
| *FLJ40330* | NR_015424 | chr2 | 88844450 | 88844999 | -1808 | 0.80 | 0.45 | 0.35 |
| *FLJ42393* | NR_024413 | chr3 | 189378061 | 189378559 | -714 | 0.78 | 0.31 | 0.47 |
| *FLJ44048* | NM_207482 | chr2 | 186376650 | 186377146 | -1107 | 0.87 | 0.54 | 0.32 |
| *FLRT1* | NM_013280 | chr11 | 63626069 | 63626571 | -1617 | 0.71 | 0.31 | 0.40 |
| *FMO3* | NM_001002294 | chr1 | 169325708 | 169326220 | -695 | 0.94 | 0.31 | 0.63 |
| *FNDC5* | NM_153756 | chr1 | 33110308 | 33110852 | -1646 | 0.65 | 0.27 | 0.38 |
| *FOLH1B* | NM_153696 | chr11 | 89031514 | 89032048 | -331 | 0.89 | 0.15 | 0.74 |
| *FOLR1* | NM_016729 | chr11 | 71580048 | 71580596 | -498 | 0.83 | 0.44 | 0.39 |
| *FOLR4* | NM_001080486 | chr11 | 93677574 | 93678064 | -631 | 0.75 | 0.44 | 0.32 |
| *FOXN1* | NM_003593 | chr17 | 23873215 | 23873502 | -1726 | 0.71 | 0.33 | 0.38 |
| *FOXP3* | NM_001114377 | chrX | 49010042 | 49010546 | -2062 | 0.73 | 0.29 | 0.44 |
| *FREQ* | NM_001128826 | chr9 | 132002551 | 132003051 | 109 | 0.64 | 0.27 | 0.37 |
| *FRMD7* | NM_194277 | chrX | 131089680 | 131090204 | -211 | 0.83 | 0.45 | 0.38 |
| *FRMD8* | NM_031904 | chr11 | 64908858 | 64909382 | -1496 | 0.78 | 0.45 | 0.33 |
| *FTSJ2* | NM_013393 | chr7 | 2250470 | 2250768 | -2260 | 0.61 | 0.30 | 0.32 |
| *FUT1* | NM_000148 | chr19 | 53951373 | 53951911 | -1183 | 0.73 | 0.41 | 0.32 |
| *FUT6* | NM_000150 | chr19 | 5792631 | 5793119 | -2133 | 0.84 | 0.50 | 0.34 |
| *FUT7* | NM_004479 | chr9 | 139048608 | 139049102 | -1742 | 0.59 | 0.26 | 0.34 |
| *FUZ* | NM_025129 | chr19 | 55009887 | 55010097 | -1712 | 0.76 | 0.44 | 0.31 |
| *FXYD1* | NM_005031 | chr19 | 40319957 | 40320359 | -2073 | 0.54 | 0.17 | 0.36 |
| *FXYD2* | NM_001127489 | chr11 | 117201608 | 117202130 | -1200 | 0.77 | 0.38 | 0.39 |
| *FXYD5* | NM_014164 | chr19 | 40335778 | 40336325 | -1414 | 0.76 | 0.46 | 0.30 |
| *FXYD7* | NM_022006 | chr19 | 40324545 | 40325041 | -1200 | 0.69 | 0.36 | 0.33 |
| *FZR1* | NM_001136197 | chr19 | 3473313 | 3473837 | -378 | 0.71 | 0.39 | 0.32 |
| *G6PC* | NM_000151 | chr17 | 38303671 | 38304220 | -2394 | 0.57 | 0.26 | 0.30 |
| *G6PC2* | NM_001081686 | chr2 | 169465077 | 169465585 | -664 | 0.81 | 0.30 | 0.50 |
| *GAB4* | NM_001037814 | chr22 | 15870849 | 15871341 | -1983 | 0.58 | 0.19 | 0.39 |
| *GABRP* | NM_014211 | chr5 | 170141934 | 170142456 | -1105 | 0.52 | 0.20 | 0.32 |
| *GABRR3* | NM_001105580 | chr3 | 99236970 | 99237479 | -703 | 0.77 | 0.42 | 0.35 |
| *GAL3ST1* | NM_004861 | chr22 | 29290830 | 29291340 | -209 | 0.86 | 0.36 | 0.51 |
| *GAL3ST4* | NM_024637 | chr7 | 99604249 | 99604583 | -107 | 0.57 | 0.23 | 0.34 |
| *GALNT10* | NM_017540 | chr5 | 153762154 | 153762648 | -1128 | 0.77 | 0.47 | 0.30 |
| *GALNT5* | NM_014568 | chr2 | 157821730 | 157822206 | -617 | 0.76 | 0.14 | 0.62 |
| *GALNT8* | NM_017417 | chr12 | 4697595 | 4698043 | -2193 | 0.89 | 0.38 | 0.50 |
| *GALR3* | NM_003614 | chr22 | 36547943 | 36548472 | -1126 | 0.71 | 0.37 | 0.34 |
| *GANAB* | NM_198334 | chr11 | 62173020 | 62173126 | -2393 | 0.73 | 0.43 | 0.30 |
| *GAP43* | NM_001130064 | chr3 | 116824479 | 116824973 | -114 | 0.63 | 0.20 | 0.44 |
| *GARNL4* | NM_001100398 | chr17 | 2644597 | 2644901 | -1732 | 0.86 | 0.47 | 0.38 |
| *GAS2* | NM_001143830 | chr11 | 22642863 | 22643377 | -1615 | 0.87 | 0.54 | 0.33 |
| *GAS6* | NM_001143946 | chr13 | 113576999 | 113577509 | 26 | 0.59 | 0.23 | 0.36 |
| *GAS7* | NM_003644 | chr17 | 9803855 | 9804239 | -556 | 0.66 | 0.25 | 0.40 |
| *GATSL3* | NM_001037666 | chr22 | 29016563 | 29017079 | -1205 | 0.90 | 0.55 | 0.35 |
| *GBA* | NM_000157 | chr1 | 153477520 | 153478041 | -103 | 0.78 | 0.24 | 0.53 |
| *GBP1* | NM_002053 | chr1 | 89304080 | 89304411 | -614 | 0.51 | 0.21 | 0.30 |
| *GBP3* | NM_018284 | chr1 | 89263027 | 89263557 | -2155 | 0.97 | 0.68 | 0.30 |
| *GBP6* | NM_198460 | chr1 | 89600133 | 89600636 | -1638 | 0.66 | 0.36 | 0.30 |
| *GCA* | NM_012198 | chr2 | 162906390 | 162906869 | -2198 | 0.81 | 0.45 | 0.36 |
| *GCK* | NM_033507 | chr7 | 44165366 | 44165876 | -209 | 0.89 | 0.43 | 0.46 |
| *GCN1L1* | NM_006836 | chr12 | 119118384 | 119118909 | -1750 | 0.83 | 0.51 | 0.32 |
| *GDF2* | NM_016204 | chr10 | 48037202 | 48037702 | -593 | 0.68 | 0.27 | 0.42 |
| *GDPD3* | NM_024307 | chr16 | 30032794 | 30033296 | -666 | 0.63 | 0.33 | 0.31 |
| *GFI1B* | NM_001135031 | chr9 | 134843002 | 134843308 | -763 | 0.82 | 0.48 | 0.34 |
| *GGT1* | NM_001032365 | chr22 | 23332339 | 23332888 | -1040 | 0.75 | 0.38 | 0.37 |
| *GGT5* | NM_001099781 | chr22 | 22973213 | 22973519 | -2256 | 0.71 | 0.27 | 0.44 |
| *GGT6* | NM_001122890 | chr17 | 4410568 | 4411076 | -197 | 0.76 | 0.42 | 0.34 |
| *GGT8P* | NR_003503 | chr2 | 91325221 | 91325715 | -1626 | 0.67 | 0.24 | 0.43 |
| *GGTLC1* | NM_178312 | chr20 | 23915850 | 23916088 | -457 | 0.60 | 0.18 | 0.42 |
| *GHRL* | NM_001134941 | chr3 | 10308783 | 10309277 | -1611 | 0.73 | 0.21 | 0.52 |
| *GINS2* | NM_016095 | chr16 | 84281231 | 84281715 | -1384 | 0.75 | 0.44 | 0.32 |
| *GIP* | NM_004123 | chr17 | 44402094 | 44402619 | -1402 | 0.72 | 0.29 | 0.43 |
| *GIPC1* | NM_005716 | chr19 | 14469319 | 14469729 | -1580 | 0.82 | 0.50 | 0.31 |
| *GJA4* | NM_002060 | chr1 | 35029813 | 35030306 | -1125 | 0.81 | 0.27 | 0.54 |
| *GJB7* | NM_198568 | chr6 | 88097588 | 88098106 | -2132 | 0.96 | 0.60 | 0.36 |
| *GJC2* | NM_020435 | chr1 | 226401759 | 226402284 | -2153 | 0.70 | 0.37 | 0.33 |
| *GLA* | NM_000169 | chrX | 100550984 | 100551490 | -1580 | 0.79 | 0.38 | 0.41 |
| *GLB1L* | NM_024506 | chr2 | 219819790 | 219820314 | -1677 | 0.94 | 0.49 | 0.44 |
| *GLI2* | NM_005270 | chr2 | 121268906 | 121269047 | -2359 | 0.69 | 0.39 | 0.30 |
| *GLIS3* | NM_152629 | chr9 | 4143124 | 4143627 | -1192 | 0.95 | 0.62 | 0.33 |
| *GLP2R* | NM_004246 | chr17 | 9667689 | 9668233 | -2144 | 0.82 | 0.50 | 0.32 |
| *GLUD2* | NM_012084 | chrX | 120008189 | 120008675 | -710 | 0.68 | 0.31 | 0.37 |
| *GMCL1L* | NR_003281 | chr5 | 177548127 | 177548623 | -1336 | 0.88 | 0.31 | 0.57 |
| *GMFB* | NM_004124 | chr14 | 54026397 | 54026875 | -1142 | 0.90 | 0.46 | 0.44 |
| *GNAT1* | NM_000172 | chr3 | 50203814 | 50204324 | 23 | 0.83 | 0.15 | 0.68 |
| *GNB3* | NM_002075 | chr12 | 6819168 | 6819644 | -229 | 0.67 | 0.23 | 0.44 |
| *GNG7* | NM_052847 | chr19 | 2654208 | 2654311 | -513 | 0.63 | 0.27 | 0.36 |
| *GNRHR* | NM_000406 | chr4 | 68306394 | 68306805 | -2200 | 0.71 | 0.41 | 0.31 |
| *GOLGA7B* | NM_001010917 | chr10 | 99597780 | 99598226 | -1982 | 0.73 | 0.32 | 0.42 |
| *GP9* | NM_000174 | chr3 | 130262319 | 130262843 | 247 | 0.68 | 0.33 | 0.35 |
| *GPAM* | NM_020918 | chr10 | 113935888 | 113936002 | -2430 | 0.92 | 0.54 | 0.39 |
| *GPBAR1* | NM_001077191 | chr2 | 218833850 | 218834358 | 122 | 0.72 | 0.33 | 0.39 |
| *GPD1* | NM_005276 | chr12 | 48783151 | 48783634 | -674 | 0.67 | 0.32 | 0.34 |
| *GPR110* | NM_025048 | chr6 | 47118992 | 47119491 | -1200 | 0.84 | 0.33 | 0.50 |
| *GPR111* | NM_153839 | chr6 | 47730699 | 47731199 | -1335 | 0.85 | 0.51 | 0.35 |
| *GPR114* | NM_153837 | chr16 | 56133145 | 56133688 | -684 | 0.83 | 0.48 | 0.35 |
| *GPR119* | NM_178471 | chrX | 129348060 | 129348564 | -1210 | 0.89 | 0.44 | 0.45 |
| *GPR142* | NM_181790 | chr17 | 69874573 | 69875094 | -405 | 0.80 | 0.49 | 0.31 |
| *GPR143* | NM_000273 | chrX | 9695376 | 9695884 | -1713 | 0.78 | 0.48 | 0.30 |
| *GPR152* | NM_206997 | chr11 | 66977866 | 66978367 | -1340 | 0.81 | 0.49 | 0.33 |
| *GPR17* | NM_005291 | chr2 | 128118249 | 128118749 | -1717 | 0.67 | 0.27 | 0.40 |
| *GPR175* | NM_016372 | chr3 | 128781814 | 128782312 | -93 | 0.84 | 0.44 | 0.40 |
| *GPR177* | NM_001002292 | chr1 | 68470700 | 68471138 | -78 | 0.51 | 0.18 | 0.32 |
| *GPR20* | NM_005293 | chr8 | 142448389 | 142448889 | -2092 | 0.84 | 0.45 | 0.39 |
| *GPR21* | NM_005294 | chr9 | 124834784 | 124835304 | -1622 | 0.63 | 0.29 | 0.35 |
| *GPR22* | NM_005295 | chr7 | 106896800 | 106897328 | -673 | 0.49 | 0.19 | 0.30 |
| *GPR34* | NM_001097579 | chrX | 41432332 | 41432860 | -573 | 0.86 | 0.29 | 0.57 |
| *GPR35* | NM_005301 | chr2 | 241216983 | 241217497 | -227 | 0.69 | 0.31 | 0.38 |
| *GPR52* | NM_005684 | chr1 | 172681397 | 172681856 | -2207 | 0.68 | 0.34 | 0.34 |
| *GPR55* | NM_005683 | chr2 | 231498525 | 231499070 | -612 | 0.68 | 0.38 | 0.30 |
| *GPR56* | NM_001145774 | chr16 | 56229717 | 56230165 | -766 | 0.82 | 0.33 | 0.50 |
| *GPR62* | NM_080865 | chr3 | 51963635 | 51964157 | -473 | 0.78 | 0.33 | 0.45 |
| *GPR68* | NM_003485 | chr14 | 90790872 | 90791358 | -1138 | 0.70 | 0.26 | 0.44 |
| *GPR77* | NM_018485 | chr19 | 52531524 | 52532069 | -413 | 0.78 | 0.47 | 0.31 |
| *GPR97* | NM_170776 | chr16 | 56257233 | 56257676 | -2202 | 0.87 | 0.51 | 0.36 |
| *GPRC5A* | NM_003979 | chr12 | 12933277 | 12933799 | -1684 | 0.76 | 0.19 | 0.57 |
| *GPRIN3* | NM_198281 | chr4 | 90449937 | 90450412 | -1990 | 0.61 | 0.28 | 0.33 |
| *GPSM1* | NM_001145639 | chr9 | 138365847 | 138366357 | -1227 | 0.62 | 0.32 | 0.30 |
| *GPSM3* | NM_022107 | chr6 | 32272627 | 32273127 | -1599 | 0.60 | 0.27 | 0.33 |
| *GPT* | NM_005309 | chr8 | 145698500 | 145698984 | -1530 | 0.68 | 0.30 | 0.38 |
| *GRAMD1A* | NM_001136199 | chr19 | 40181374 | 40181893 | -1451 | 0.88 | 0.44 | 0.44 |
| *GRAMD2* | NM_001012642 | chr15 | 70279475 | 70279677 | -2386 | 0.82 | 0.34 | 0.48 |
| *GRAMD3* | NM_001146320 | chr5 | 125785726 | 125786250 | -779 | 0.66 | 0.27 | 0.39 |
| *GRAMD4* | NM_015124 | chr22 | 45399931 | 45400448 | -1131 | 0.59 | 0.15 | 0.44 |
| *GRB14* | NM_004490 | chr2 | 165187358 | 165187878 | -1012 | 0.76 | 0.44 | 0.33 |
| *GRB7* | NM_001030002 | chr17 | 35145969 | 35146485 | -1888 | 0.79 | 0.48 | 0.31 |
| *GREB1* | NM_033090 | chr2 | 11599964 | 11600464 | -87 | 0.81 | 0.50 | 0.32 |
| *GRHL1* | NM_198182 | chr2 | 10016859 | 10017413 | -1608 | 0.92 | 0.62 | 0.30 |
| *GRHL2* | NM_024915 | chr8 | 102573251 | 102573732 | -351 | 0.71 | 0.31 | 0.40 |
| *GRHL3* | NM_198173 | chr1 | 24517578 | 24518095 | -630 | 0.87 | 0.38 | 0.49 |
| *GRM2* | NM_000839 | chr3 | 51714245 | 51714734 | -1630 | 0.89 | 0.45 | 0.43 |
| *GRN* | NM_002087 | chr17 | 39776615 | 39777113 | -1152 | 0.61 | 0.13 | 0.48 |
| *GRRP1* | NM_024869 | chr1 | 26355681 | 26356116 | -2198 | 0.50 | 0.16 | 0.34 |
| *GRXCR1* | NM_001080476 | chr4 | 42587652 | 42588109 | -2159 | 0.83 | 0.49 | 0.34 |
| *GRXCR2* | NM_001080516 | chr5 | 145234901 | 145234997 | -2225 | 0.94 | 0.62 | 0.33 |
| *GSDMB* | NM_018530 | chr17 | 35327708 | 35328198 | -634 | 0.82 | 0.42 | 0.40 |
| *GSN* | NM_000177 | chr9 | 123100524 | 123101009 | -1132 | 0.57 | 0.23 | 0.34 |
| *GSTM1* | NM_000561 | chr1 | 110029969 | 110030474 | -1718 | 0.66 | 0.20 | 0.47 |
| *GSTM5* | NM_000851 | chr1 | 110054418 | 110054918 | -1718 | 0.75 | 0.32 | 0.44 |
| *GSTT1* | NM_000853 | chr22 | 22716524 | 22716730 | -2343 | 0.85 | 0.55 | 0.30 |
| *GUCA2A* | NM_033553 | chr1 | 42404321 | 42404731 | -1544 | 0.93 | 0.57 | 0.36 |
| *GUCY2E* | NR_024042 | chr11 | 76110968 | 76111456 | -731 | 0.78 | 0.36 | 0.42 |
| *GUK1* | NM_001159391 | chr1 | 226396607 | 226397138 | -2153 | 0.65 | 0.20 | 0.45 |
| *GYS2* | NM_021957 | chr12 | 21651038 | 21651530 | -2236 | 0.66 | 0.26 | 0.39 |
| *H1FOO* | NM_153833 | chr3 | 130744330 | 130744854 | -154 | 0.68 | 0.34 | 0.34 |
| *HACE1* | NM_020771 | chr6 | 105416217 | 105416719 | -1981 | 0.67 | 0.25 | 0.42 |
| *HAMP* | NM_021175 | chr19 | 40462811 | 40463280 | -2203 | 0.59 | 0.21 | 0.38 |
| *HAPLN3* | NM_178232 | chr15 | 87240616 | 87241134 | -1101 | 0.72 | 0.33 | 0.38 |
| *HAPLN4* | NM_023002 | chr19 | 19236662 | 19236778 | -2124 | 0.68 | 0.30 | 0.38 |
| *HAS2AS* | NR_002835 | chr8 | 122718330 | 122718804 | -2199 | 0.80 | 0.48 | 0.32 |
| *HAUS7* | NM_017518 | chrX | 152391291 | 152391675 | -2200 | 0.78 | 0.43 | 0.35 |
| *HBII-52-24* | NR_003495 | chr15 | 23009074 | 23009566 | -578 | 0.72 | 0.21 | 0.51 |
| *HBII-52-45* | NR_003498 | chr15 | 23059907 | 23060434 | -595 | 0.68 | 0.24 | 0.44 |
| *HBZ* | NM_005332 | chr16 | 142337 | 142839 | -265 | 0.63 | 0.26 | 0.37 |
| *HCG9* | NM_005844 | chr6 | 30049955 | 30050459 | -663 | 0.90 | 0.57 | 0.33 |
| *HCP5* | NM_006674 | chr6 | 31538023 | 31538521 | -665 | 0.70 | 0.36 | 0.33 |
| *HCRT* | NM_001524 | chr17 | 37590752 | 37591246 | -3 | 0.85 | 0.45 | 0.40 |
| *HCRTR1* | NM_001525 | chr1 | 31854414 | 31854920 | -1220 | 0.89 | 0.59 | 0.30 |
| *HCST* | NM_001007469 | chr19 | 41084735 | 41085251 | -228 | 0.64 | 0.14 | 0.50 |
| *HDAC1* | NM_004964 | chr1 | 32528805 | 32529105 | -1339 | 0.83 | 0.51 | 0.32 |
| *HDAC10* | NM_001159286 | chr22 | 49033860 | 49034354 | -2146 | 0.66 | 0.27 | 0.39 |
| *HDX* | NM_144657 | chrX | 83644582 | 83645071 | -712 | 0.68 | 0.35 | 0.33 |
| *HERC4* | NM_015601 | chr10 | 69505373 | 69505786 | -470 | 0.63 | 0.26 | 0.37 |
| *HERPUD1* | NM_001010989 | chr16 | 55520851 | 55521224 | -2210 | 0.86 | 0.53 | 0.33 |
| *HFE2* | NM_145277 | chr1 | 144122584 | 144123042 | -1734 | 0.85 | 0.13 | 0.72 |
| *HGFAC* | NM_001528 | chr4 | 3411547 | 3412037 | -1731 | 0.68 | 0.38 | 0.31 |
| *HHATL* | NM_020707 | chr3 | 42719878 | 42720356 | -2100 | 0.73 | 0.27 | 0.46 |
| *HIF3A* | NM_022462 | chr19 | 51491545 | 51492048 | -1701 | 0.43 | 0.13 | 0.30 |
| *HIPK4* | NM_144685 | chr19 | 45587669 | 45588195 | 2 | 0.80 | 0.48 | 0.32 |
| *HIST1H4I* | NM_003495 | chr6 | 27213731 | 27214209 | -1096 | 0.70 | 0.30 | 0.40 |
| *HK3* | NM_002115 | chr5 | 176259886 | 176260384 | -1196 | 0.83 | 0.52 | 0.31 |
| *HKDC1* | NM_025130 | chr10 | 70649553 | 70650023 | -276 | 0.60 | 0.27 | 0.33 |
| *HLA-DPA1* | NM_033554 | chr6 | 33152195 | 33152395 | -2939 | 0.79 | 0.37 | 0.41 |
| *HMGB3L1* | NR_002165 | chr20 | 32886231 | 32886777 | -578 | 0.91 | 0.28 | 0.63 |
| *HMGXB4* | NM_001003681 | chr22 | 33981091 | 33981565 | -2160 | 0.76 | 0.35 | 0.41 |
| *HMOX1* | NM_002133 | chr22 | 34106194 | 34106690 | -644 | 0.76 | 0.44 | 0.32 |
| *HMOX2* | NM_001127205 | chr16 | 4484397 | 4484580 | -1370 | 0.86 | 0.50 | 0.36 |
| *HNMT* | NM_001024074 | chr2 | 138435862 | 138436337 | -2177 | 0.86 | 0.39 | 0.47 |
| *HOXA10* | NM_153715 | chr7 | 27187179 | 27187704 | -1040 | 0.74 | 0.21 | 0.52 |
| *HOXA11AS* | NR_002795 | chr7 | 27188222 | 27188728 | -3076 | 0.51 | 0.20 | 0.31 |
| *HOXA6* | NM_024014 | chr7 | 27156370 | 27156860 | -2722 | 0.51 | 0.18 | 0.33 |
| *HOXB2* | NM_002145 | chr17 | 43978354 | 43978854 | -1212 | 0.66 | 0.26 | 0.40 |
| *HOXC4* | NM_153633 | chr12 | 52731501 | 52731956 | -2198 | 0.77 | 0.28 | 0.49 |
| *HPCAL4* | NM_016257 | chr1 | 39931630 | 39932039 | -2158 | 0.77 | 0.43 | 0.33 |
| *HPD* | NM_002150 | chr12 | 120782615 | 120783015 | -1663 | 0.82 | 0.33 | 0.49 |
| *HPS6* | NM_024747 | chr10 | 103813720 | 103814245 | -1153 | 0.89 | 0.58 | 0.30 |
| *HRCT1* | NM_001039792 | chr9 | 35894364 | 35894872 | -1570 | 0.75 | 0.33 | 0.43 |
| *HRH1* | NM_001098213 | chr3 | 11151811 | 11152360 | -1692 | 0.84 | 0.53 | 0.31 |
| *HRH4* | NM_001143828 | chr18 | 20293954 | 20294481 | -372 | 0.73 | 0.43 | 0.31 |
| *HSD17B2* | NM_002153 | chr16 | 80624490 | 80624974 | -1626 | 0.65 | 0.17 | 0.48 |
| *HSD3B7* | NM_001142777 | chr16 | 30901599 | 30902128 | -2155 | 0.68 | 0.20 | 0.48 |
| *HSF2* | NM_001135564 | chr6 | 122761411 | 122761942 | -717 | 0.78 | 0.46 | 0.32 |
| *HSP90AB4P* | NR_002927 | chr15 | 56774140 | 56774609 | -1758 | 0.82 | 0.36 | 0.46 |
| *HSP90B3P* | NR_003130 | chr1 | 91871334 | 91871820 | -1578 | 0.85 | 0.37 | 0.48 |
| *HSPB2* | NM_001541 | chr11 | 111287198 | 111287688 | -1226 | 0.81 | 0.48 | 0.33 |
| *HSPB7* | NM_014424 | chr1 | 16220851 | 16221355 | -3231 | 0.83 | 0.45 | 0.38 |
| *HSPBL2* | NR_024392 | chr9 | 73813434 | 73813921 | -805 | 0.52 | 0.14 | 0.38 |
| *HTR1E* | NM_000865 | chr6 | 87702364 | 87702907 | -1106 | 0.95 | 0.55 | 0.40 |
| *HTR1F* | NM_000866 | chr3 | 88112524 | 88113024 | -1641 | 0.83 | 0.43 | 0.40 |
| *HTR2B* | NM_000867 | chr2 | 231697934 | 231698417 | -107 | 0.81 | 0.21 | 0.60 |
| *HTR3A* | NM_000869 | chr11 | 113350960 | 113351474 | 98 | 0.71 | 0.35 | 0.36 |
| *HTR3D* | NM_001145143 | chr3 | 185231084 | 185231327 | -1017 | 0.60 | 0.16 | 0.44 |
| *HTRA3* | NM_053044 | chr4 | 8320905 | 8321413 | -1232 | 0.67 | 0.37 | 0.30 |
| *HYAL1* | NM_153281 | chr3 | 50326679 | 50327187 | -2117 | 0.81 | 0.42 | 0.39 |
| *HYAL2* | NM_003773 | chr3 | 50335937 | 50336429 | -898 | 0.76 | 0.35 | 0.40 |
| *IAPP* | NM_000415 | chr12 | 21416429 | 21416940 | -383 | 0.85 | 0.38 | 0.47 |
| *ICAM2* | NM_000873 | chr17 | 59437601 | 59438130 | 149 | 0.81 | 0.47 | 0.35 |
| *ICOS* | NM_012092 | chr2 | 204508369 | 204508923 | -1101 | 0.89 | 0.45 | 0.43 |
| *IDH3A* | NM_005530 | chr15 | 76226907 | 76227425 | -1607 | 0.88 | 0.43 | 0.45 |
| *IDO2* | NM_194294 | chr8 | 39910197 | 39910697 | -1183 | 0.54 | 0.15 | 0.39 |
| *IDS* | NM_000202 | chrX | 148396215 | 148396685 | -1681 | 0.81 | 0.46 | 0.35 |
| *IFFO1* | NM_001039670 | chr12 | 6536288 | 6536786 | -1047 | 0.56 | 0.22 | 0.35 |
| *IFI27* | NM_001130080 | chr14 | 93645489 | 93645973 | -1100 | 0.82 | 0.47 | 0.35 |
| *IFI44* | NM_006417 | chr1 | 78885695 | 78886206 | -2113 | 0.92 | 0.49 | 0.43 |
| *IFI44L* | NM_006820 | chr1 | 78858210 | 78858724 | -208 | 0.56 | 0.19 | 0.37 |
| *IFNA17* | NM_021268 | chr9 | 21218622 | 21219125 | -652 | 0.62 | 0.26 | 0.36 |
| *IFNK* | NM_020124 | chr9 | 27512801 | 27513302 | -1259 | 0.83 | 0.54 | 0.30 |
| *IFRD2* | NM_006764 | chr3 | 50307395 | 50307499 | -2417 | 0.69 | 0.38 | 0.30 |
| *IFT172* | NM_015662 | chr2 | 27568445 | 27568563 | -2429 | 0.85 | 0.36 | 0.49 |
| *IGFALS* | NM_001146006 | chr16 | 1786420 | 1786615 | -2782 | 0.90 | 0.49 | 0.41 |
| *IGFN1* | NM_178275 | chr1 | 199447576 | 199448062 | -573 | 0.81 | 0.21 | 0.60 |
| *IGLL3* | NM_001013618 | chr22 | 24043007 | 24043550 | -608 | 0.89 | 0.58 | 0.30 |
| *IGSF10* | NM_178822 | chr3 | 152659707 | 152660204 | -768 | 0.65 | 0.25 | 0.40 |
| *IGSF11* | NM_001015887 | chr3 | 120238231 | 120238732 | -2115 | 0.65 | 0.34 | 0.31 |
| *IGSF9* | NM_001135050 | chr1 | 158183357 | 158183655 | -1496 | 0.93 | 0.41 | 0.52 |
| *IKBKG* | NM_001099856 | chrX | 153423095 | 153423589 | -310 | 0.75 | 0.32 | 0.43 |
| *IKZF4* | NM_022465 | chr12 | 54700303 | 54700811 | -398 | 0.58 | 0.18 | 0.40 |
| *IL13RA2* | NM_000640 | chrX | 114159955 | 114160469 | -1749 | 0.76 | 0.24 | 0.51 |
| *IL16* | NM_004513 | chr15 | 79372106 | 79372644 | -2146 | 0.64 | 0.20 | 0.45 |
| *IL17B* | NM_014443 | chr5 | 148738892 | 148739375 | -102 | 0.57 | 0.22 | 0.35 |
| *IL17RD* | NM_017563 | chr3 | 57175782 | 57176269 | -1582 | 0.83 | 0.48 | 0.35 |
| *IL17RE* | NM_153480 | chr3 | 9919311 | 9919811 | 50 | 0.58 | 0.18 | 0.39 |
| *IL17REL* | NM_001001694 | chr22 | 48793623 | 48794044 | -651 | 0.86 | 0.53 | 0.33 |
| *IL18BP* | NM_001145055 | chr11 | 71387749 | 71388241 | -625 | 0.89 | 0.16 | 0.73 |
| *IL18RAP* | NM_003853 | chr2 | 102400744 | 102401266 | -680 | 0.58 | 0.15 | 0.43 |
| *IL1B* | NM_000576 | chr2 | 113311261 | 113311762 | -684 | 0.67 | 0.38 | 0.30 |
| *IL1F8* | NM_014438 | chr2 | 113528866 | 113529325 | -2184 | 0.56 | 0.14 | 0.43 |
| *IL1R2* | NM_004633 | chr2 | 101972365 | 101972896 | -2106 | 0.71 | 0.41 | 0.30 |
| *IL1RN* | NM_000577 | chr2 | 113591557 | 113592054 | -134 | 0.72 | 0.30 | 0.43 |
| *IL20RB* | NM_144717 | chr3 | 138159098 | 138159592 | -51 | 0.55 | 0.25 | 0.30 |
| *IL27* | NM_145659 | chr16 | 28425518 | 28426019 | -112 | 0.60 | 0.22 | 0.38 |
| *IL27RA* | NM_004843 | chr19 | 14000301 | 14000601 | -2810 | 0.68 | 0.34 | 0.34 |
| *IL28RA* | NM_170743 | chr1 | 24388101 | 24388191 | -1808 | 0.92 | 0.60 | 0.33 |
| *IL29* | NM_172140 | chr19 | 44478563 | 44479098 | 26 | 0.57 | 0.27 | 0.30 |
| *IL34* | NM_152456 | chr16 | 69235598 | 69236104 | -2117 | 0.79 | 0.48 | 0.31 |
| *IL4* | NM_000589 | chr5 | 132035896 | 132036386 | -1130 | 0.71 | 0.37 | 0.34 |
| *IL9R* | NM_002186 | chrX | 154879974 | 154880513 | -195 | 0.77 | 0.20 | 0.57 |
| *IMP3* | NM_018285 | chr15 | 73721286 | 73721813 | -1899 | 0.79 | 0.40 | 0.40 |
| *IMPDH1* | NM_001142573 | chr7 | 127835064 | 127835562 | -2053 | 0.77 | 0.45 | 0.32 |
| *IMPG1* | NM_001563 | chr6 | 76840962 | 76841448 | -2150 | 0.78 | 0.34 | 0.44 |
| *INADL* | NM_176877 | chr1 | 61978466 | 61978522 | -2242 | 0.99 | 0.64 | 0.35 |
| *INHBC* | NM_005538 | chr12 | 56114696 | 56115218 | 148 | 0.65 | 0.35 | 0.30 |
| *INHBE* | NM_031479 | chr12 | 56134638 | 56135146 | -470 | 0.85 | 0.46 | 0.39 |
| *INMT* | NM_006774 | chr7 | 30757829 | 30758329 | -196 | 0.83 | 0.38 | 0.44 |
| *INPP1* | NM_001128928 | chr2 | 190914000 | 190914523 | -2178 | 0.91 | 0.59 | 0.32 |
| *INPP5J* | NM_001002837 | chr22 | 29847967 | 29848493 | -730 | 0.82 | 0.43 | 0.40 |
| *INSC* | NM_001031853 | chr11 | 15089777 | 15090274 | -519 | 0.70 | 0.19 | 0.50 |
| *INS-IGF2* | NM_001042376 | chr11 | 2140904 | 2141388 | -2131 | 0.80 | 0.35 | 0.45 |
| *INSL5* | NM_005478 | chr1 | 67039882 | 67040350 | -589 | 0.74 | 0.32 | 0.42 |
| *IP6K1* | NM_001006115 | chr3 | 49800616 | 49800940 | -1801 | 0.76 | 0.40 | 0.36 |
| *IP6K3* | NM_001142883 | chr6 | 33823704 | 33824198 | -1211 | 0.76 | 0.30 | 0.45 |
| *IPCEF1* | NM_001130699 | chr6 | 154693563 | 154693858 | -803 | 0.84 | 0.49 | 0.35 |
| *IPO4* | NM_024658 | chr14 | 23728910 | 23729425 | -1203 | 0.77 | 0.43 | 0.34 |
| *IPW* | NR_023915 | chr15 | 22912304 | 22912807 | -228 | 0.89 | 0.59 | 0.30 |
| *IQCF1* | NM_152397 | chr3 | 51912479 | 51913011 | -354 | 0.70 | 0.38 | 0.32 |
| *IQCF5* | NM_001145059 | chr3 | 51886612 | 51887079 | -2205 | 0.49 | 0.13 | 0.35 |
| *IRAK1* | NM_001025242 | chrX | 152939941 | 152940448 | -1658 | 0.78 | 0.39 | 0.39 |
| *IRF9* | NM_006084 | chr14 | 23699604 | 23700108 | -405 | 0.88 | 0.56 | 0.32 |
| *ISG20* | NM_002201 | chr15 | 86980605 | 86981151 | -2164 | 0.75 | 0.45 | 0.30 |
| *ISL2* | NM_145805 | chr15 | 74414685 | 74415168 | -1274 | 0.46 | 0.16 | 0.30 |
| *ISLR* | NM_005545 | chr15 | 72251250 | 72251748 | -1640 | 0.66 | 0.18 | 0.48 |
| *ISX* | NM_001008494 | chr22 | 33790639 | 33791113 | -1253 | 0.83 | 0.37 | 0.46 |
| *ITFG2* | NM_018463 | chr12 | 2789695 | 2790144 | -2203 | 0.86 | 0.30 | 0.56 |
| *ITGAD* | NM_005353 | chr16 | 31309696 | 31310152 | -2209 | 0.76 | 0.41 | 0.35 |
| *ITGAE* | NM_002208 | chr17 | 3651125 | 3651631 | -92 | 0.53 | 0.20 | 0.32 |
| *ITGB2* | NM_001127491 | chr21 | 45173555 | 45174069 | -631 | 0.70 | 0.24 | 0.47 |
| *ITGB3* | NM_000212 | chr17 | 42685224 | 42685720 | -734 | 0.86 | 0.40 | 0.45 |
| *ITGB6* | NM_000888 | chr2 | 160766983 | 160767300 | -2305 | 0.69 | 0.18 | 0.51 |
| *ITIH3* | NM_002217 | chr3 | 52802349 | 52802842 | -1227 | 0.77 | 0.31 | 0.46 |
| *ITIH4* | NM_002218 | chr3 | 52841589 | 52842081 | -2101 | 0.73 | 0.09 | 0.65 |
| *IYD* | NM_203395 | chr6 | 150730735 | 150731224 | -740 | 0.70 | 0.32 | 0.37 |
| *JAGN1* | NM_032492 | chr3 | 9905075 | 9905314 | -2075 | 0.75 | 0.18 | 0.56 |
| *JAK3* | NM_000215 | chr19 | 17821902 | 17822231 | -2225 | 0.77 | 0.42 | 0.34 |
| *JAKMIP3* | NM_001105521 | chr10 | 133766312 | 133766828 | -1732 | 0.85 | 0.55 | 0.30 |
| *JDP2* | NM_001135049 | chr14 | 74967199 | 74967693 | -1143 | 0.80 | 0.27 | 0.53 |
| *JUB* | NM_198086 | chr14 | 22516223 | 22516711 | -195 | 0.73 | 0.31 | 0.42 |
| *KANK1* | NM_153186 | chr9 | 696066 | 696562 | -581 | 0.74 | 0.37 | 0.37 |
| *KANK2* | NM_001136191 | chr19 | 11171270 | 11171484 | -2153 | 0.81 | 0.49 | 0.32 |
| *KBTBD7* | NM_032138 | chr13 | 40666794 | 40667327 | -358 | 0.55 | 0.24 | 0.30 |
| *KCNE1* | NM_000219 | chr21 | 34805422 | 34805876 | -166 | 0.85 | 0.43 | 0.41 |
| *KCNE2* | NM_172201 | chr21 | 34657921 | 34658432 | -15 | 0.75 | 0.39 | 0.35 |
| *KCNE3* | NM_005472 | chr11 | 73856716 | 73857201 | -710 | 0.60 | 0.27 | 0.33 |
| *KCNH2* | NM_172057 | chr7 | 150284213 | 150284737 | -627 | 0.80 | 0.39 | 0.41 |
| *KCNIP2* | NM_173194 | chr10 | 103591048 | 103591560 | -1703 | 0.71 | 0.23 | 0.48 |
| *KCNIP3* | NM_013434 | chr2 | 95326460 | 95326940 | -98 | 0.61 | 0.30 | 0.30 |
| *KCNJ11* | NM_000525 | chr11 | 17368366 | 17368876 | -1839 | 0.64 | 0.25 | 0.40 |
| *KCNJ13* | NM_002242 | chr2 | 233350876 | 233351421 | -1629 | 0.78 | 0.42 | 0.37 |
| *KCNJ4* | NM_004981 | chr22 | 37169828 | 37170329 | -99 | 0.68 | 0.15 | 0.52 |
| *KCNK7* | NM_005714 | chr11 | 65120930 | 65121016 | -930 | 0.81 | 0.22 | 0.59 |
| *KCNS1* | NM_002251 | chr20 | 43163054 | 43163554 | -137 | 0.50 | 0.16 | 0.34 |
| *KCNT2* | NM_198503 | chr1 | 194846512 | 194846610 | -2439 | 0.95 | 0.52 | 0.42 |
| *KCP* | NM_001135914 | chr7 | 128338376 | 128338877 | -617 | 0.74 | 0.41 | 0.34 |
| *KCTD6* | NM_153331 | chr3 | 58458250 | 58458745 | -633 | 0.79 | 0.45 | 0.34 |
| *KDELR3* | NM_006855 | chr22 | 37192139 | 37192461 | -1728 | 0.71 | 0.37 | 0.34 |
| *KDM5D* | NM_001146705 | chrY | 20367606 | 20368126 | -1653 | 0.82 | 0.52 | 0.30 |
| *KDM6B* | NM_001080424 | chr17 | 7683468 | 7683945 | -252 | 0.76 | 0.08 | 0.67 |
| *KEL* | NM_000420 | chr7 | 142369470 | 142369972 | -96 | 0.80 | 0.42 | 0.38 |
| *KIAA0101* | NM_001029989 | chr15 | 62462609 | 62463069 | -2084 | 0.75 | 0.44 | 0.31 |
| *KIAA0467* | NM_015284 | chr1 | 43660413 | 43660893 | -730 | 0.67 | 0.29 | 0.37 |
| *KIAA1109* | NM_015312 | chr4 | 123311075 | 123311617 | 139 | 0.86 | 0.44 | 0.42 |
| *KIAA1211* | NM_020722 | chr4 | 56729339 | 56729885 | -1505 | 0.87 | 0.46 | 0.41 |
| *KIAA1274* | NM_014431 | chr10 | 71906150 | 71906705 | -2141 | 0.83 | 0.33 | 0.50 |
| *KIAA1324L* | NM_001142749 | chr7 | 86528947 | 86529443 | -2245 | 0.67 | 0.36 | 0.31 |
| *KIAA1383* | NM_019090 | chr1 | 231006662 | 231007166 | -346 | 0.88 | 0.54 | 0.34 |
| *KIAA1467* | NM_020853 | chr12 | 13086331 | 13086795 | -2018 | 0.90 | 0.57 | 0.34 |
| *KIAA1522* | NM_020888 | chr1 | 32978401 | 32978948 | -1423 | 0.81 | 0.31 | 0.49 |
| *KIAA1881* | NM_001080400 | chr19 | 4470604 | 4471098 | -2135 | 0.72 | 0.13 | 0.60 |
| *KIF18B* | NM_001080443 | chr17 | 40382100 | 40382598 | -1741 | 0.48 | 0.17 | 0.31 |
| *KIF20B* | NM_016195 | chr10 | 91448257 | 91448775 | -2830 | 0.65 | 0.26 | 0.39 |
| *KIF3B* | NM_004798 | chr20 | 30326806 | 30327145 | -2151 | 0.91 | 0.58 | 0.33 |
| *KIF4A* | NM_012310 | chrX | 69424743 | 69425251 | -1622 | 0.74 | 0.44 | 0.30 |
| *KIFC3* | NM_001130099 | chr16 | 56391293 | 56391780 | -2106 | 0.59 | 0.19 | 0.40 |
| *KIR2DL1* | NM_014218 | chr19 | 59972881 | 59973335 | 32 | 0.76 | 0.30 | 0.46 |
| *KIR2DS4* | NM_012314 | chr19 | 60035873 | 60036347 | 125 | 0.74 | 0.40 | 0.34 |
| *KIR3DL1* | NM_013289 | chr19 | 60019536 | 60020080 | 104 | 0.74 | 0.29 | 0.45 |
| *KISS1* | NM_002256 | chr1 | 202434530 | 202434728 | -2387 | 0.74 | 0.36 | 0.38 |
| *KLF8* | NM_001159296 | chrX | 56273108 | 56273603 | -2238 | 0.81 | 0.42 | 0.38 |
| *KLHDC6* | NM_207335 | chr3 | 129122723 | 129123211 | -1624 | 0.53 | 0.22 | 0.32 |
| *KLHL11* | NM_018143 | chr17 | 37277293 | 37277605 | -2294 | 0.85 | 0.48 | 0.38 |
| *KLHL38* | NM_001081675 | chr8 | 124736403 | 124736809 | -2235 | 0.67 | 0.25 | 0.42 |
| *KLHL4* | NM_019117 | chrX | 86657945 | 86658470 | -1162 | 0.90 | 0.29 | 0.61 |
| *KLK3* | NM_001030047 | chr19 | 56049692 | 56050194 | -39 | 0.43 | 0.12 | 0.31 |
| *KLK6* | NM_001012964 | chr19 | 56166502 | 56166817 | -2800 | 0.55 | 0.20 | 0.36 |
| *KLK8* | NM_007196 | chr19 | 56196711 | 56197173 | -172 | 0.69 | 0.21 | 0.47 |
| *KLKB1* | NM_000892 | chr4 | 187384524 | 187385048 | -879 | 0.89 | 0.19 | 0.70 |
| *KLRA1* | NM_006611 | chr12 | 10646058 | 10646147 | -2401 | 0.47 | 0.14 | 0.33 |
| *KLRC4* | NM_013431 | chr12 | 10453577 | 10454112 | -221 | 0.60 | 0.24 | 0.36 |
| *KPNA2* | NM_002266 | chr17_random | 1725083 | 1725595 | -1108 | 0.86 | 0.56 | 0.30 |
| *KRT16* | NM_005557 | chr17 | 37022545 | 37023057 | -196 | 0.74 | 0.39 | 0.35 |
| *KRT23* | NM_015515 | chr17 | 36349661 | 36349815 | -2376 | 0.69 | 0.36 | 0.33 |
| *KRT5* | NM_000424 | chr12 | 51201964 | 51202456 | -1700 | 0.57 | 0.19 | 0.38 |
| *KRT8* | NM_002273 | chr12 | 51586186 | 51586697 | -1306 | 0.71 | 0.37 | 0.35 |
| *KRT82* | NM_033033 | chr12 | 51087812 | 51088332 | -1629 | 0.53 | 0.16 | 0.37 |
| *KRT83* | NM_002282 | chr12 | 51001797 | 51002298 | -598 | 0.85 | 0.30 | 0.55 |
| *KRTAP10-2* | NM_198693 | chr21 | 44795720 | 44796230 | -159 | 0.60 | 0.29 | 0.30 |
| *KRTAP10-4* | NM_198687 | chr21 | 44817740 | 44818232 | -47 | 0.54 | 0.19 | 0.35 |
| *KRTAP10-6* | NM_198688 | chr21 | 44837197 | 44837701 | -635 | 0.53 | 0.15 | 0.38 |
| *KRTAP11-1* | NM_175858 | chr21 | 31177741 | 31178199 | -2225 | 0.72 | 0.42 | 0.30 |
| *KRTAP19-2* | NM_181608 | chr21 | 30782305 | 30782677 | -953 | 0.86 | 0.48 | 0.38 |
| *KRTAP21-1* | NM_181619 | chr21 | 31050019 | 31050517 | -701 | 0.88 | 0.49 | 0.40 |
| *KRTAP25-1* | NM_001128598 | chr21 | 30585659 | 30586178 | -2215 | 0.88 | 0.55 | 0.32 |
| *KRTAP3-1* | NM_031958 | chr17_random | 342379 | 342897 | -1572 | 0.74 | 0.40 | 0.34 |
| *KRTAP4-12* | NM_031854 | chr17 | 36534464 | 36534943 | -758 | 0.92 | 0.42 | 0.50 |
| *KRTAP5-6* | NM_001012416 | chr11 | 1672762 | 1673274 | -1982 | 0.83 | 0.52 | 0.32 |
| *KRTAP5-7* | NM_001012503 | chr11 | 70913979 | 70914494 | -1723 | 0.56 | 0.24 | 0.32 |
| *KYNU* | NM_001032998 | chr2 | 143349687 | 143350203 | -1719 | 0.87 | 0.41 | 0.46 |
| *L3MBTL* | NM_015478 | chr20 | 41575409 | 41575901 | -811 | 0.64 | 0.30 | 0.35 |
| *LAG3* | NM_002286 | chr12 | 6749493 | 6749964 | -2201 | 0.69 | 0.20 | 0.49 |
| *LAMB2* | NM_002292 | chr3 | 49147456 | 49147952 | -2101 | 0.71 | 0.17 | 0.53 |
| *LAPTM5* | NM_006762 | chr1 | 31004656 | 31005141 | -1628 | 0.83 | 0.39 | 0.44 |
| *LBP* | NM_004139 | chr20 | 36407302 | 36407782 | -756 | 0.86 | 0.46 | 0.40 |
| *LCAP* | NM_001039768 | chrX | 152798826 | 152799366 | -224 | 0.69 | 0.38 | 0.31 |
| *LCAT* | NM_000229 | chr16 | 66535867 | 66536375 | -605 | 0.59 | 0.26 | 0.33 |
| *LCE3A* | NM_178431 | chr1 | 150863543 | 150864075 | -1606 | 0.89 | 0.42 | 0.47 |
| *LCE3B* | NM_178433 | chr1 | 150851449 | 150851948 | -1211 | 0.67 | 0.36 | 0.31 |
| *LCN10* | NM_001001712 | chr9 | 138757679 | 138758191 | -703 | 0.60 | 0.26 | 0.33 |
| *LCN12* | NM_178536 | chr9 | 138965957 | 138966457 | -381 | 0.79 | 0.39 | 0.39 |
| *LCN15* | NM_203347 | chr9 | 138778725 | 138779233 | -193 | 0.71 | 0.32 | 0.38 |
| *LCN2* | NM_005564 | chr9 | 129950855 | 129951361 | -444 | 0.75 | 0.30 | 0.45 |
| *LCNL1* | NM_207510 | chr9 | 138995318 | 138995800 | -1706 | 0.78 | 0.42 | 0.36 |
| *LCTL* | NM_207338 | chr15 | 64645274 | 64645780 | -638 | 0.73 | 0.29 | 0.43 |
| *LDB1* | NM_003893 | chr10 | 103866976 | 103867168 | -2359 | 0.83 | 0.23 | 0.60 |
| *LDB3* | NM_001080114 | chr10 | 88417334 | 88417810 | -833 | 0.81 | 0.51 | 0.30 |
| *LDHD* | NM_153486 | chr16 | 73708505 | 73708745 | -459 | 0.77 | 0.32 | 0.45 |
| *LDLRAD2* | NM_001013693 | chr1 | 22010964 | 22011460 | -132 | 0.62 | 0.29 | 0.33 |
| *LDLRAP1* | NM_015627 | chr1 | 25741292 | 25741792 | -1120 | 0.73 | 0.35 | 0.38 |
| *LECT2* | NM_002302 | chr5 | 135318465 | 135318966 | -93 | 0.58 | 0.18 | 0.40 |
| *LEFTY1* | NM_020997 | chr1 | 224145357 | 224145757 | -2098 | 0.90 | 0.26 | 0.64 |
| *LEFTY2* | NM_003240 | chr1 | 224197731 | 224198032 | -2338 | 0.81 | 0.48 | 0.33 |
| *LGALS1* | NM_002305 | chr22 | 36399134 | 36399667 | -2157 | 0.74 | 0.33 | 0.41 |
| *LGALS12* | NM_001142535 | chr11 | 63028112 | 63028599 | -1743 | 0.94 | 0.45 | 0.49 |
| *LGALS3BP* | NM_005567 | chr17 | 74487586 | 74488070 | -172 | 0.72 | 0.37 | 0.35 |
| *LGALS4* | NM_006149 | chr19 | 43997570 | 43998021 | -2215 | 0.73 | 0.42 | 0.31 |
| *LGALS7* | NM_002307 | chr19 | 43956957 | 43957461 | -1212 | 0.61 | 0.31 | 0.30 |
| *LGALS7B* | NM_001042507 | chr19 | 43971043 | 43971527 | -404 | 0.73 | 0.41 | 0.32 |
| *LGI4* | NM_139284 | chr19 | 40319957 | 40320359 | -2140 | 0.54 | 0.17 | 0.36 |
| *LGR6* | NM_021636 | chr1 | 200439152 | 200439650 | -126 | 0.85 | 0.46 | 0.39 |
| *LHFPL4* | NM_198560 | chr3 | 9572075 | 9572561 | -1832 | 0.82 | 0.26 | 0.56 |
| *LILRA1* | NM_006863 | chr19 | 59794508 | 59795047 | -2146 | 0.51 | 0.09 | 0.41 |
| *LILRP2* | NR_003061 | chr19 | 59911231 | 59911738 | 72 | 0.66 | 0.34 | 0.32 |
| *LIM2* | NM_030657 | chr19 | 56583373 | 56583855 | -605 | 0.91 | 0.26 | 0.65 |
| *LIME1* | NM_017806 | chr20 | 61836016 | 61836473 | -2176 | 0.79 | 0.31 | 0.48 |
| *LIMS2* | NM_017980 | chr2 | 128139439 | 128139925 | -1099 | 0.49 | 0.11 | 0.38 |
| *LIN28B* | NM_001004317 | chr6 | 105510225 | 105510726 | -1139 | 0.53 | 0.14 | 0.39 |
| *LINGO4* | NM_001004432 | chr1 | 150046485 | 150046997 | -2235 | 0.71 | 0.24 | 0.47 |
| *LIPE* | NM_005357 | chr19 | 47623263 | 47623665 | -46 | 0.86 | 0.42 | 0.44 |
| *LMAN2* | NM_006816 | chr5 | 176711933 | 176712152 | -551 | 0.74 | 0.40 | 0.34 |
| *LMCD1* | NM_014583 | chr3 | 8516423 | 8516948 | -1824 | 0.75 | 0.45 | 0.30 |
| *LMOD1* | NM_012134 | chr1 | 200184171 | 200184385 | -1939 | 0.80 | 0.48 | 0.32 |
| *LOC100101116* | NR_003589 | chrY | 6317558 | 6318052 | -636 | 0.89 | 0.46 | 0.43 |
| *LOC100101118* | NR_003591 | chrY | 6402132 | 6402642 | -716 | 0.84 | 0.46 | 0.38 |
| *LOC100128288* | NR_024447 | chr17 | 8206445 | 8206931 | -2104 | 0.70 | 0.38 | 0.32 |
| *LOC100128573* | NR_024491 | chr19 | 7445786 | 7446237 | -1764 | 0.77 | 0.35 | 0.42 |
| *LOC100129354* | NR_024046 | chr3 | 47026964 | 47027456 | -825 | 0.75 | 0.40 | 0.35 |
| *LOC100129534* | NR_024489 | chr1 | 2273826 | 2274342 | -124 | 0.76 | 0.44 | 0.32 |
| *LOC100131496* | NR_024594 | chr20 | 45379993 | 45380487 | -412 | 0.83 | 0.20 | 0.63 |
| *LOC100131551* | NR_024480 | chr3 | 195514256 | 195514776 | -1229 | 0.59 | 0.29 | 0.30 |
| *LOC100132354* | NR_024478 | chr6 | 43965387 | 43965896 | -1100 | 0.86 | 0.50 | 0.36 |
| *LOC100133050* | NR_027503 | chr5 | 99753795 | 99754237 | -2159 | 0.82 | 0.47 | 0.35 |
| *LOC100133893* | NM_001146221 | chr12 | 27816936 | 27817405 | -1694 | 0.72 | 0.34 | 0.38 |
| *LOC100133920* | NR_024443 | chr9 | 68940514 | 68941038 | -404 | 0.45 | 0.13 | 0.32 |
| *LOC100133991* | NR_024434 | chr17 | 40678664 | 40679195 | -2155 | 0.84 | 0.40 | 0.44 |
| *LOC100134259* | NR_024452 | chr2 | 46907125 | 46907615 | -1136 | 0.79 | 0.49 | 0.30 |
| *LOC100169752* | NR_023362 | chr10 | 127252048 | 127252552 | -629 | 0.61 | 0.30 | 0.31 |
| *LOC100190938* | NR_024461 | chr17 | 38169041 | 38169221 | -2330 | 0.67 | 0.24 | 0.43 |
| *LOC100190940* | NR_024457 | chr12 | 129094258 | 129094754 | -1666 | 0.79 | 0.47 | 0.32 |
| *LOC100270710* | NR_026754 | chr10 | 99468254 | 99468748 | -609 | 0.75 | 0.34 | 0.42 |
| *LOC100271831* | NR_027081 | chr16 | 30032794 | 30033296 | 112 | 0.63 | 0.33 | 0.31 |
| *LOC100272216* | NR_027439 | chr5 | 68965161 | 68965677 | -635 | 0.67 | 0.37 | 0.30 |
| *LOC121952* | NR_026965 | chr13 | 102329485 | 102330023 | -695 | 0.82 | 0.49 | 0.33 |
| *LOC143188* | NR_015409 | chr10 | 114598229 | 114598772 | -2161 | 0.89 | 0.51 | 0.38 |
| *LOC143678* | NM_001080446 | chr11 | 45886355 | 45886849 | -1193 | 0.89 | 0.59 | 0.30 |
| *LOC149478* | NM_001136537 | chr1 | 45044784 | 45045293 | -1701 | 0.73 | 0.34 | 0.39 |
| *LOC150197* | NR_026919 | chr22 | 18571414 | 18571887 | -2203 | 0.68 | 0.32 | 0.35 |
| *LOC150622* | NR_026832 | chr2 | 5988293 | 5988802 | -1721 | 0.60 | 0.26 | 0.34 |
| *LOC151300* | NR_015390 | chr2 | 219546823 | 219547281 | -2197 | 0.54 | 0.23 | 0.31 |
| *LOC153910* | NR_027311 | chr6 | 143002912 | 143003202 | -2391 | 0.87 | 0.48 | 0.40 |
| *LOC158376* | NR_024283 | chr9 | 35899301 | 35899801 | 72 | 0.79 | 0.44 | 0.35 |
| *LOC158696* | NR_026935 | chrX | 137528448 | 137528943 | -1230 | 0.60 | 0.30 | 0.30 |
| *LOC164380* | NR_001279 | chr20 | 23447416 | 23447912 | -118 | 0.78 | 0.29 | 0.49 |
| *LOC169834* | NM_001101338 | chr9 | 114814740 | 114815262 | -708 | 0.64 | 0.34 | 0.30 |
| *LOC221442* | NR_026938 | chr6 | 41174765 | 41175273 | -1731 | 0.80 | 0.47 | 0.33 |
| *LOC283332* | NR_026948 | chr12 | 48592797 | 48593293 | -1132 | 0.62 | 0.24 | 0.38 |
| *LOC283856* | NR_027078 | chr16 | 54784935 | 54785385 | -2653 | 0.64 | 0.13 | 0.51 |
| *LOC284023* | NR_024349 | chr17 | 7760430 | 7760929 | -689 | 0.65 | 0.20 | 0.45 |
| *LOC284233* | NR_026756 | chr18 | 14325431 | 14325895 | -1758 | 0.65 | 0.26 | 0.40 |
| *LOC284551* | NR_027085 | chr1 | 31755211 | 31755717 | -1158 | 0.77 | 0.43 | 0.34 |
| *LOC284837* | NR_026961 | chr21 | 44058781 | 44059271 | -2150 | 0.53 | 0.19 | 0.33 |
| *LOC285033* | NM_001037228 | chr2 | 96269188 | 96269712 | -253 | 0.85 | 0.42 | 0.43 |
| *LOC285375* | NR_027103 | chr3 | 13665331 | 13665861 | -1625 | 0.81 | 0.47 | 0.34 |
| *LOC285419* | NR_027105 | chr4 | 124792937 | 124793428 | -206 | 0.72 | 0.33 | 0.39 |
| *LOC285733* | NR_015397 | chr6 | 131188574 | 131189056 | -1201 | 0.73 | 0.40 | 0.32 |
| *LOC285847* | NR_027117 | chr6 | 35812685 | 35813187 | -234 | 0.78 | 0.39 | 0.39 |
| *LOC285954* | NR_027118 | chr7 | 41698556 | 41699074 | -1223 | 0.76 | 0.41 | 0.34 |
| *LOC339240* | NR_001443 | chr17 | 18264897 | 18265403 | -1069 | 0.65 | 0.34 | 0.31 |
| *LOC339524* | NR_026985 | chr1 | 87368173 | 87368693 | -1763 | 0.82 | 0.47 | 0.34 |
| *LOC348262* | NM_001093767 | chr17_random | 2342264 | 2342611 | -1637 | 0.67 | 0.33 | 0.34 |
| *LOC387787* | NM_001144869 | chr11 | 73883255 | 73883791 | -1120 | 0.71 | 0.31 | 0.40 |
| *LOC388503* | NM_001013640 | chr19 | 10012608 | 10013115 | -169 | 0.89 | 0.34 | 0.55 |
| *LOC400713* | NM_001145434 | chr19 | 57563526 | 57563916 | -1260 | 0.87 | 0.58 | 0.30 |
| *LOC401052* | NM_001008737 | chr3 | 10028127 | 10028627 | -598 | 0.63 | 0.23 | 0.41 |
| *LOC440905* | NR_026758 | chr2 | 130526663 | 130527161 | -1738 | 0.41 | 0.06 | 0.34 |
| *LOC441476* | NM_001004353 | chr9 | 139263659 | 139264162 | -1639 | 0.81 | 0.46 | 0.35 |
| *LOC441869* | NM_001145210 | chr1 | 1346974 | 1347458 | -703 | 0.71 | 0.40 | 0.30 |
| *LOC442308* | NR_003598 | chr7 | 55680609 | 55681091 | 45 | 0.81 | 0.45 | 0.36 |
| *LOC442459* | NR_024608 | chrX | 99083516 | 99083926 | -2224 | 0.73 | 0.20 | 0.53 |
| *LOC553137* | NR_015365 | chr6 | 107331099 | 107331596 | -1777 | 0.84 | 0.49 | 0.35 |
| *LOC55908* | NM_018687 | chr19 | 11208853 | 11209355 | -712 | 0.58 | 0.26 | 0.32 |
| *LOC606724* | NR_002454 | chr16 | 29368100 | 29368614 | 191 | 0.87 | 0.40 | 0.47 |
| *LOC643008* | NR_015452 | chr17 | 71144738 | 71145240 | -1119 | 0.78 | 0.30 | 0.48 |
| *LOC643719* | NR_027620 | chr19 | 39761341 | 39761821 | -1104 | 0.74 | 0.23 | 0.51 |
| *LOC644844* | NM_001145643 | chr15 | 38428103 | 38428560 | -2193 | 0.70 | 0.28 | 0.42 |
| *LOC646471* | NR_024498 | chr1 | 26022531 | 26023029 | -96 | 0.82 | 0.22 | 0.60 |
| *LOC646982* | NR_024505 | chr13 | 39954957 | 39955498 | -2084 | 0.78 | 0.37 | 0.41 |
| *LOC647309* | NM_001146686 | chr3 | 192064624 | 192065073 | -1689 | 0.81 | 0.31 | 0.50 |
| *LOC647946* | NR_024391 | chr18 | 35587601 | 35588096 | -1891 | 0.69 | 0.38 | 0.31 |
| *LOC648691* | NR_027426 | chr22 | 21230288 | 21230739 | -1241 | 0.69 | 0.36 | 0.33 |
| *LOC650623* | NR_027512 | chr10 | 81110306 | 81110785 | -2190 | 0.73 | 0.13 | 0.60 |
| *LOC653653* | NR_027408 | chr17 | 55535674 | 55536158 | -854 | 0.74 | 0.45 | 0.30 |
| *LOC678655* | NR_015382 | chr12 | 6431350 | 6431868 | -665 | 0.54 | 0.21 | 0.33 |
| *LOC723809* | NR_027374 | chr7 | 104355144 | 104355663 | -1075 | 0.66 | 0.24 | 0.42 |
| *LOC727811* | NM_001097632 | chr3 | 46422776 | 46423266 | -636 | 0.64 | 0.23 | 0.41 |
| *LOC728358* | NM_001042500 | chr8 | 6846111 | 6846587 | -2215 | 0.62 | 0.22 | 0.39 |
| *LOC728606* | NR_024259 | chr18 | 22539202 | 22539663 | -1832 | 0.58 | 0.22 | 0.36 |
| *LOC729234* | NR_003698 | chr2 | 96038990 | 96039484 | -788 | 0.71 | 0.31 | 0.40 |
| *LOC730668* | NR_027240 | chr22 | 44778730 | 44779271 | -2158 | 0.65 | 0.22 | 0.42 |
| *LOC80154* | NR_026811 | chr15 | 80982087 | 80982336 | -2255 | 0.79 | 0.49 | 0.30 |
| *LOC84931* | NR_027181 | chr2 | 120942692 | 120942870 | -2386 | 0.76 | 0.36 | 0.40 |
| *LOC91450* | NR_026998 | chr15 | 76075607 | 76076103 | -2233 | 0.68 | 0.37 | 0.31 |
| *LPAL2* | NM_024492 | chr6 | 160852522 | 160853036 | -633 | 0.81 | 0.19 | 0.62 |
| *LPAR5* | NM_001142961 | chr12 | 6610935 | 6611337 | -60 | 0.77 | 0.47 | 0.30 |
| *LPIN1* | NM_145693 | chr2 | 11802302 | 11802800 | -1639 | 0.87 | 0.31 | 0.56 |
| *LPP* | NM_005578 | chr3 | 189412808 | 189413277 | -371 | 0.79 | 0.37 | 0.41 |
| *LPPR2* | NM_022737 | chr19 | 11325191 | 11325706 | -1657 | 0.83 | 0.45 | 0.38 |
| *LRG1* | NM_052972 | chr19 | 4490900 | 4491425 | -126 | 0.59 | 0.24 | 0.35 |
| *LRIT2* | NM_001017924 | chr10 | 85975711 | 85976107 | -645 | 0.89 | 0.39 | 0.50 |
| *LRIT3* | NM_198506 | chr4 | 110990968 | 110991513 | -693 | 0.83 | 0.42 | 0.41 |
| *LRP5L* | NM_182492 | chr22 | 24088887 | 24089183 | -511 | 0.84 | 0.31 | 0.52 |
| *LRRC19* | NM_022901 | chr9 | 26996283 | 26996781 | -841 | 0.86 | 0.52 | 0.34 |
| *LRRC23* | NM_001135217 | chr12 | 6883338 | 6883839 | -568 | 0.64 | 0.16 | 0.48 |
| *LRRC25* | NM_145256 | chr19 | 18369802 | 18370288 | -630 | 0.77 | 0.32 | 0.45 |
| *LRRC28* | NM_144598 | chr15 | 97607700 | 97608207 | -1220 | 0.75 | 0.40 | 0.35 |
| *LRRC29* | NM_001004055 | chr16 | 65819640 | 65820136 | -1486 | 0.60 | 0.28 | 0.32 |
| *LRRC32* | NM_001128922 | chr11 | 76060163 | 76060655 | -970 | 0.75 | 0.26 | 0.50 |
| *LRRC4B* | NM_001080457 | chr19 | 55764502 | 55764978 | -1626 | 0.50 | 0.16 | 0.34 |
| *LRRC52* | NM_001005214 | chr1 | 163777674 | 163778222 | -2153 | 0.53 | 0.21 | 0.32 |
| *LRRC61* | NM_001142928 | chr7 | 149648809 | 149649347 | -2150 | 0.72 | 0.38 | 0.34 |
| *LRRC70* | NM_181506 | chr5 | 61909435 | 61909930 | -635 | 0.87 | 0.54 | 0.33 |
| *LRRIQ4* | NM_001080460 | chr3 | 171020425 | 171020827 | -1777 | 0.69 | 0.20 | 0.49 |
| *LRRN4* | NM_152611 | chr20 | 5984729 | 5985133 | -2237 | 0.76 | 0.33 | 0.43 |
| *LSM10* | NM_032881 | chr1 | 36636917 | 36637396 | -1076 | 0.79 | 0.44 | 0.35 |
| *LST1* | NM_007161 | chr6 | 31661584 | 31662085 | -114 | 0.65 | 0.34 | 0.30 |
| *LTBP4* | NM_003573 | chr19 | 45789595 | 45790080 | -1073 | 0.82 | 0.42 | 0.40 |
| *LTC4S* | NM_145867 | chr5 | 179153275 | 179153799 | -54 | 0.62 | 0.18 | 0.43 |
| *LY6D* | NM_003695 | chr8 | 143866457 | 143866955 | -1696 | 0.70 | 0.31 | 0.39 |
| *LY6G5B* | NM_021221 | chr6 | 31745228 | 31745718 | -1233 | 0.54 | 0.22 | 0.32 |
| *LY6G6C* | NM_025261 | chr6 | 31797249 | 31797751 | -11 | 0.52 | 0.12 | 0.40 |
| *LY96* | NM_015364 | chr8 | 75064233 | 75064539 | -1754 | 0.81 | 0.43 | 0.38 |
| *LYPD2* | NM_205545 | chr8 | 143831802 | 143832294 | -1094 | 0.72 | 0.22 | 0.51 |
| *LYPLA2* | NM_007260 | chr1 | 23987795 | 23988344 | -2162 | 0.81 | 0.50 | 0.31 |
| *LZTR1* | NM_006767 | chr22 | 19664666 | 19665168 | -1640 | 0.80 | 0.40 | 0.39 |
| *LZTS1* | NM_021020 | chr8 | 20157446 | 20157940 | -610 | 0.75 | 0.30 | 0.46 |
| *MAFG* | NM_032711 | chr17_random | 2441022 | 2441534 | -175 | 0.64 | 0.32 | 0.31 |
| *MAG* | NM_002361 | chr19 | 40472460 | 40472761 | -2266 | 0.77 | 0.47 | 0.30 |
| *MAGEA10* | NM_001011543 | chrX | 151059067 | 151059577 | -1641 | 0.76 | 0.27 | 0.49 |
| *MAGEA11* | NM_001011544 | chrX | 148604440 | 148604956 | -191 | 0.86 | 0.46 | 0.40 |
| *MAGEA2B* | NM_153488 | chrX | 151672102 | 151672606 | -1599 | 0.72 | 0.37 | 0.35 |
| *MAGEA5* | NM_021049 | chrX | 151037939 | 151038439 | -1089 | 0.65 | 0.23 | 0.41 |
| *MAGEB1* | NM_177404 | chrX | 30173929 | 30174405 | -1024 | 0.80 | 0.47 | 0.34 |
| *MAGEB10* | NM_182506 | chrX | 27734076 | 27734604 | -1705 | 0.81 | 0.42 | 0.39 |
| *MAGED2* | NM_014599 | chrX | 54850615 | 54851079 | -674 | 0.66 | 0.28 | 0.38 |
| *MAGEE1* | NM_020932 | chrX | 75562094 | 75562648 | -2149 | 0.83 | 0.53 | 0.30 |
| *MALL* | NM_005434 | chr2 | 110231273 | 110231773 | -91 | 0.77 | 0.33 | 0.43 |
| *MAP1A* | NM_002373 | chr15 | 41596764 | 41597280 | -75 | 0.54 | 0.19 | 0.35 |
| *MAP2K3* | NM_002756 | chr17 | 21132952 | 21133401 | -2196 | 0.86 | 0.45 | 0.42 |
| *MAP3K13* | NM_004721 | chr3 | 186563308 | 186563828 | -95 | 0.70 | 0.38 | 0.32 |
| *MAP4* | NM_001134365 | chr3 | 47928821 | 47929330 | -2193 | 0.86 | 0.42 | 0.44 |
| *MAP4K1* | NM_001042600 | chr19 | 43800011 | 43800495 | 230 | 0.62 | 0.27 | 0.35 |
| *MAP4K2* | NM_004579 | chr11 | 64327746 | 64328252 | -710 | 0.67 | 0.23 | 0.44 |
| *MAP7D3* | NM_024597 | chrX | 135161535 | 135162033 | -510 | 0.70 | 0.21 | 0.48 |
| *MAPK8* | NM_002750 | chr10 | 49277269 | 49277802 | -2156 | 0.50 | 0.18 | 0.32 |
| *MATN4* | NM_003833 | chr20 | 43370956 | 43371464 | -829 | 0.62 | 0.27 | 0.35 |
| *MB* | NM_005368 | chr22 | 34345645 | 34345955 | -2470 | 0.69 | 0.34 | 0.34 |
| *MBD5* | NM_018328 | chr2 | 148931039 | 148931547 | -1214 | 0.85 | 0.56 | 0.30 |
| *MBL1P1* | NR_002724 | chr10 | 81669160 | 81669690 | -488 | 0.83 | 0.42 | 0.41 |
| *MBL2* | NM_000242 | chr10 | 54201805 | 54202336 | -604 | 0.47 | 0.07 | 0.40 |
| *MBTPS2* | NM_015884 | chrX | 21765644 | 21766088 | -1761 | 0.88 | 0.43 | 0.45 |
| *MCART3P* | NR_026540 | chr6 | 66552058 | 66552613 | -2156 | 0.84 | 0.35 | 0.49 |
| *MCART6* | NM_001012755 | chrX | 103289235 | 103289704 | -1105 | 0.66 | 0.33 | 0.33 |
| *MCF2* | NM_005369 | chrX | 138553495 | 138554033 | -1127 | 0.52 | 0.16 | 0.37 |
| *MCHR1* | NM_005297 | chr22 | 39405097 | 39405607 | 225 | 0.58 | 0.25 | 0.33 |
| *MCTS1* | NM_001137554 | chrX | 119620259 | 119620533 | -2183 | 0.82 | 0.44 | 0.38 |
| *MDGA2* | NM_182830 | chr14 | 46883183 | 46883738 | -1272 | 0.78 | 0.29 | 0.49 |
| *MEA1* | NM_014623 | chr6 | 43091064 | 43091274 | -1573 | 0.69 | 0.36 | 0.33 |
| *MED18* | NM_001127350 | chr1 | 28526215 | 28526518 | -1732 | 0.68 | 0.24 | 0.44 |
| *MED24* | NM_001079518 | chr17 | 35466443 | 35466829 | -2221 | 0.90 | 0.56 | 0.34 |
| *MED25* | NM_030973 | chr19 | 55010097 | 55010519 | -3049 | 0.75 | 0.43 | 0.31 |
| *MED9* | NM_018019 | chr17 | 17319555 | 17320037 | -1228 | 0.56 | 0.21 | 0.35 |
| *MEF2B* | NM_001145785 | chr19 | 19142540 | 19143048 | -696 | 0.47 | 0.10 | 0.37 |
| *MEFV* | NM_000243 | chr16 | 3248352 | 3248665 | -1880 | 0.95 | 0.27 | 0.68 |
| *MEI1* | NM_152513 | chr22 | 40424106 | 40424321 | -1249 | 0.76 | 0.38 | 0.38 |
| *MEOX1* | NM_001040002 | chr17 | 39095838 | 39096324 | -1293 | 0.63 | 0.29 | 0.33 |
| *METT11D1* | NM_001029991 | chr14 | 20526634 | 20527136 | -919 | 0.60 | 0.29 | 0.31 |
| *MFAP4* | NM_002404 | chr17 | 19231530 | 19232036 | -697 | 0.92 | 0.48 | 0.44 |
| *MFNG* | NM_002405 | chr22 | 36212770 | 36213292 | -700 | 0.69 | 0.22 | 0.47 |
| *MFRP* | NM_031433 | chr11 | 118722581 | 118723057 | -226 | 0.83 | 0.25 | 0.58 |
| *MFSD7* | NM_032219 | chr4 | 673322 | 673826 | -601 | 0.48 | 0.15 | 0.33 |
| *MGAT1* | NM_001114619 | chr5 | 180165532 | 180165947 | -2231 | 0.89 | 0.60 | 0.30 |
| *MGAT3* | NM_001098270 | chr22 | 38212041 | 38212431 | -938 | 0.83 | 0.36 | 0.47 |
| *MGAT4C* | NM_013244 | chr12 | 85756764 | 85757268 | -204 | 0.79 | 0.38 | 0.41 |
| *MGAT5* | NM_002410 | chr2 | 134727908 | 134728398 | -146 | 0.90 | 0.54 | 0.36 |
| *MGC16703* | NR_003608 | chr22 | 19699117 | 19699623 | -794 | 0.50 | 0.16 | 0.34 |
| *MGC24975* | NM_001134316 | chr19 | 5735461 | 5735957 | 67 | 0.52 | 0.18 | 0.33 |
| *MGC27382* | NR_027310 | chr1 | 78466027 | 78466534 | -1612 | 0.83 | 0.50 | 0.33 |
| *MICB* | NM_005931 | chr6 | 31572583 | 31573088 | -997 | 0.76 | 0.38 | 0.39 |
| *MID1* | NM_033290 | chrX | 10764111 | 10764221 | -2436 | 0.92 | 0.55 | 0.38 |
| *MIP* | NM_012064 | chr12 | 55134640 | 55135142 | -195 | 0.75 | 0.41 | 0.35 |
| *MLLT11* | NM_006818 | chr1 | 149296853 | 149297397 | -1649 | 0.79 | 0.29 | 0.50 |
| *MLX* | NM_170607 | chr17 | 37970636 | 37971116 | -1727 | 0.78 | 0.30 | 0.48 |
| *MLXIPL* | NM_032951 | chr7 | 72678809 | 72679238 | -2217 | 0.72 | 0.27 | 0.45 |
| *MMACHC* | NM_015506 | chr1 | 45736670 | 45737209 | -1502 | 0.65 | 0.14 | 0.52 |
| *MMD2* | NM_001100600 | chr7 | 4965754 | 4966234 | -624 | 0.66 | 0.31 | 0.35 |
| *MMEL1* | NM_033467 | chr1 | 2554235 | 2554731 | -194 | 0.79 | 0.40 | 0.39 |
| *MMP12* | NM_002426 | chr11 | 102252295 | 102252769 | -1610 | 0.73 | 0.13 | 0.60 |
| *MMP19* | NM_002429 | chr12 | 54522945 | 54523461 | -201 | 0.73 | 0.33 | 0.41 |
| *MMP21* | NM_147191 | chr10 | 127456304 | 127456800 | -2172 | 0.62 | 0.30 | 0.32 |
| *MMP9* | NM_004994 | chr20 | 44070597 | 44071103 | -103 | 0.60 | 0.12 | 0.47 |
| *MMRN2* | NM_024756 | chr10 | 88708813 | 88709320 | -1661 | 0.62 | 0.23 | 0.40 |
| *MND1* | NM_032117 | chr4 | 154483927 | 154484242 | -1165 | 0.79 | 0.42 | 0.37 |
| *MOBKL2C* | NM_145279 | chr1 | 46853737 | 46854245 | -599 | 0.82 | 0.36 | 0.46 |
| *MOGAT2* | NM_025098 | chr11 | 75106218 | 75106718 | -113 | 0.92 | 0.12 | 0.79 |
| *MOGAT3* | NM_178176 | chr7 | 100633099 | 100633511 | -2283 | 0.85 | 0.54 | 0.31 |
| *MON1A* | NM_001142501 | chr3 | 49943259 | 49943781 | -1071 | 0.75 | 0.29 | 0.46 |
| *MORC4* | NM_001085354 | chrX | 106131263 | 106131753 | -1410 | 0.94 | 0.61 | 0.32 |
| *MORN4* | NM_001098831 | chr10 | 99384974 | 99385372 | -1270 | 0.76 | 0.43 | 0.33 |
| *MPHOSPH9* | NM_022782 | chr12 | 122272234 | 122272564 | -5 | 0.71 | 0.33 | 0.37 |
| *MPI* | NM_002435 | chr15 | 72967997 | 72968474 | -1226 | 0.82 | 0.51 | 0.31 |
| *MPO* | NM_000250 | chr17 | 53713656 | 53714141 | -603 | 0.62 | 0.26 | 0.36 |
| *MRAP* | NM_178817 | chr21 | 32584596 | 32585061 | -1165 | 0.93 | 0.61 | 0.33 |
| *MRGPRE* | NM_001039165 | chr11 | 3211131 | 3211631 | -1189 | 0.57 | 0.11 | 0.46 |
| *MRI1* | NM_001031727 | chr19 | 13734993 | 13735471 | -1104 | 0.63 | 0.33 | 0.30 |
| *MRPL23* | NM_021134 | chr11 | 1923586 | 1924110 | -1229 | 0.70 | 0.39 | 0.32 |
| *MRPL42P5* | NR_002208 | chr15 | 38614230 | 38614520 | -2334 | 0.93 | 0.55 | 0.38 |
| *MRPL55* | NM_181441 | chr1 | 226364087 | 226364623 | -719 | 0.55 | 0.25 | 0.30 |
| *MRPS22* | NM_020191 | chr3 | 140543671 | 140544139 | -1645 | 0.57 | 0.24 | 0.33 |
| *MS4A10* | NM_206893 | chr11 | 60307517 | 60308031 | -1622 | 0.68 | 0.37 | 0.31 |
| *MS4A14* | NM_001079692 | chr11 | 59919603 | 59920125 | -198 | 0.81 | 0.37 | 0.44 |
| *MSGN1* | NM_001105569 | chr2 | 17859821 | 17860319 | -1196 | 0.63 | 0.31 | 0.32 |
| *MSL3L2* | NR_024322 | chr2 | 234443424 | 234443968 | -1902 | 0.89 | 0.58 | 0.31 |
| *MSLNL* | NM_001025190 | chr16 | 774766 | 775290 | -2101 | 0.60 | 0.23 | 0.37 |
| *MSR1* | NM_002445 | chr8 | 16096574 | 16097091 | -2161 | 0.57 | 0.10 | 0.47 |
| *MSRB2* | NM_012228 | chr10 | 23422039 | 23422494 | -2165 | 0.97 | 0.66 | 0.31 |
| *MSRB3* | NM_001031679 | chr12 | 63956871 | 63957374 | -1631 | 0.81 | 0.43 | 0.38 |
| *MST1R* | NM_002447 | chr3 | 49917975 | 49918474 | -1914 | 0.75 | 0.25 | 0.50 |
| *MSTN* | NM_005259 | chr2 | 190637068 | 190637544 | -1606 | 0.72 | 0.40 | 0.33 |
| *MT1G* | NM_005950 | chr16 | 55260341 | 55260855 | -1120 | 0.76 | 0.29 | 0.47 |
| *MT1H* | NM_005951 | chr16 | 55260341 | 55260855 | -628 | 0.76 | 0.29 | 0.47 |
| *MT1IP* | NR_003669 | chr16 | 55266714 | 55267193 | -195 | 0.72 | 0.42 | 0.31 |
| *MTG1* | NM_138384 | chr10 | 135056127 | 135056430 | -1331 | 0.81 | 0.19 | 0.61 |
| *MTM1* | NM_000252 | chrX | 149485538 | 149486080 | -1895 | 0.88 | 0.41 | 0.48 |
| *MTMR1* | NM_003828 | chrX | 149610621 | 149611144 | -1643 | 0.92 | 0.55 | 0.38 |
| *MTMR4* | NM_004687 | chr17 | 53951986 | 53952450 | -1968 | 0.68 | 0.38 | 0.30 |
| *MUC2* | NM_002457 | chr11 | 1064005 | 1064503 | -620 | 0.60 | 0.24 | 0.36 |
| *MURC* | NM_001018116 | chr9 | 102378180 | 102378689 | -1721 | 0.48 | 0.03 | 0.46 |
| *MUSTN1* | NM_205853 | chr3 | 52844602 | 52845124 | -603 | 0.74 | 0.42 | 0.32 |
| *MVP* | NM_005115 | chr16 | 29738721 | 29739171 | -341 | 0.54 | 0.18 | 0.35 |
| *MXD3* | NM_001142935 | chr5 | 176673812 | 176674306 | -2161 | 0.74 | 0.42 | 0.32 |
| *MYADML2* | NM_001145113 | chr17 | 77498164 | 77498691 | -27 | 0.72 | 0.13 | 0.60 |
| *MYBPH* | NM_004997 | chr1 | 201411507 | 201412015 | -196 | 0.75 | 0.39 | 0.36 |
| *MYCBPAP* | NM_032133 | chr17 | 45939862 | 45940405 | -609 | 0.85 | 0.49 | 0.36 |
| *MYCT1* | NM_025107 | chr6 | 153058762 | 153059258 | -1712 | 0.57 | 0.19 | 0.38 |
| *MYEF2* | NM_016132 | chr15 | 46259729 | 46259945 | -1987 | 0.93 | 0.60 | 0.34 |
| *MYEOV* | NM_138768 | chr11 | 68817348 | 68817843 | -601 | 0.87 | 0.33 | 0.54 |
| *MYH6* | NM_002471 | chr14 | 22948183 | 22948685 | -1112 | 0.75 | 0.32 | 0.43 |
| *MYH7* | NM_000257 | chr14 | 22975585 | 22976089 | -1127 | 0.59 | 0.29 | 0.30 |
| *MYH8* | NM_002472 | chr17 | 10266969 | 10267452 | -1218 | 0.77 | 0.38 | 0.38 |
| *MYL4* | NM_001002841 | chr17 | 42639010 | 42639461 | -2190 | 0.95 | 0.31 | 0.64 |
| *MYL7* | NM_021223 | chr7 | 44147884 | 44148404 | -703 | 0.71 | 0.38 | 0.33 |
| *MYO15A* | NM_016239 | chr17 | 17950305 | 17950840 | -2171 | 0.83 | 0.40 | 0.42 |
| *MYO18A* | NM_078471 | chr17 | 24532384 | 24532872 | -1095 | 0.84 | 0.34 | 0.50 |
| *MYO1F* | NM_012335 | chr19 | 8548259 | 8548567 | -106 | 0.81 | 0.46 | 0.35 |
| *MYO7A* | NM_000260 | chr11 | 76514533 | 76515080 | -2150 | 0.78 | 0.43 | 0.36 |
| *MYOZ3* | NM_001122853 | chr5 | 150019106 | 150019630 | -1228 | 0.82 | 0.27 | 0.55 |
| *NAALADL1* | NM_005468 | chr11 | 64582440 | 64582928 | -99 | 0.60 | 0.19 | 0.41 |
| *NAIP* | NM_004536 | chr5 | 70460703 | 70461010 | -520 | 0.79 | 0.38 | 0.41 |
| *NALCN* | NM_052867 | chr13 | 100869145 | 100869285 | -2401 | 0.81 | 0.50 | 0.31 |
| *NANS* | NM_018946 | chr9 | 99857119 | 99857556 | -1441 | 0.79 | 0.41 | 0.38 |
| *NAP1L5* | NM_153757 | chr4 | 89838385 | 89838903 | -598 | 0.83 | 0.44 | 0.40 |
| *NAPRT1* | NM_145201 | chr8 | 144733444 | 144733962 | -2047 | 0.80 | 0.48 | 0.32 |
| *NAPSA* | NM_004851 | chr19 | 55560407 | 55560932 | 73 | 0.91 | 0.46 | 0.45 |
| *NCF4* | NM_000631 | chr22 | 35586095 | 35586589 | -633 | 0.93 | 0.59 | 0.34 |
| *NCOA1* | NM_003743 | chr2 | 24660415 | 24660921 | -181 | 0.64 | 0.28 | 0.36 |
| *NCOA4* | NM_005437 | chr10 | 51239951 | 51240418 | -2184 | 0.86 | 0.38 | 0.47 |
| *NCR2* | NM_004828 | chr6 | 41409068 | 41409617 | -2162 | 0.74 | 0.41 | 0.34 |
| *NCRNA00085* | NR_024330 | chr19 | 56886995 | 56887513 | -1150 | 0.58 | 0.25 | 0.34 |
| *NCRNA00093* | NR_024130 | chr10 | 101675998 | 101676510 | -701 | 0.75 | 0.29 | 0.46 |
| *NCRNA00114* | NR_027067 | chr21 | 39046496 | 39047009 | -701 | 0.85 | 0.53 | 0.31 |
| *NCRNA00160* | NR_024351 | chr21 | 35017185 | 35017687 | -538 | 0.67 | 0.30 | 0.37 |
| *NCRNA00174* | NR_026873 | chr7 | 65503583 | 65504102 | -1012 | 0.88 | 0.39 | 0.49 |
| *NDST1* | NM_001543 | chr5 | 149867304 | 149867820 | -304 | 0.86 | 0.27 | 0.58 |
| *NDUFA3* | NM_004542 | chr19 | 59295350 | 59295899 | -2346 | 0.69 | 0.40 | 0.30 |
| *NDUFB8* | NM_005004 | chr10 | 102281293 | 102281820 | -1930 | 0.86 | 0.52 | 0.34 |
| *NDUFC1* | NM_002494 | chr4 | 140438381 | 140438743 | -2155 | 0.75 | 0.38 | 0.37 |
| *NECAB3* | NM_031231 | chr20 | 31727289 | 31727779 | -1609 | 0.82 | 0.42 | 0.40 |
| *NEDD4L* | NM_001144965 | chr18 | 53966846 | 53967324 | -477 | 0.73 | 0.40 | 0.34 |
| *NEIL2* | NM_001135746 | chr8 | 11662160 | 11662416 | -2292 | 0.89 | 0.45 | 0.44 |
| *NEIL3* | NM_018248 | chr4 | 178466028 | 178466549 | -1695 | 0.67 | 0.26 | 0.42 |
| *NELL2* | NM_001145110 | chr12 | 43593818 | 43594335 | -98 | 0.95 | 0.49 | 0.47 |
| *NET1* | NM_005863 | chr10 | 5477594 | 5478090 | -703 | 0.86 | 0.51 | 0.35 |
| *NEU3* | NM_006656 | chr11 | 74375181 | 74375706 | -2153 | 0.96 | 0.64 | 0.31 |
| *NEU4* | NM_080741 | chr2 | 242399765 | 242400255 | -735 | 0.71 | 0.32 | 0.40 |
| *NEURL3* | NR_026875 | chr2 | 96539727 | 96540045 | -2313 | 0.98 | 0.65 | 0.33 |
| *NFATC2IP* | NM_032815 | chr16 | 28867553 | 28867778 | -2152 | 0.72 | 0.38 | 0.34 |
| *NFIC* | NM_005597 | chr19 | 3316630 | 3317117 | -698 | 0.64 | 0.34 | 0.30 |
| *NFKBID* | NM_139239 | chr19 | 41084735 | 41085251 | -1601 | 0.64 | 0.14 | 0.50 |
| *NFKBIZ* | NM_001005474 | chr3 | 103027108 | 103027567 | -2208 | 0.78 | 0.18 | 0.61 |
| *NFXL1* | NM_152995 | chr4 | 47612175 | 47612692 | -1043 | 0.82 | 0.41 | 0.41 |
| *NGEF* | NM_019850 | chr2 | 233586155 | 233586559 | -162 | 0.61 | 0.24 | 0.37 |
| *NGRN* | NM_001033088 | chr15 | 88607911 | 88608432 | -1726 | 0.81 | 0.40 | 0.41 |
| *NHP2L1* | NM_001003796 | chr22 | 40416900 | 40417183 | -2182 | 0.91 | 0.28 | 0.62 |
| *NKAIN1* | NM_024522 | chr1 | 31435800 | 31435978 | -2302 | 0.84 | 0.22 | 0.61 |
| *NKG7* | NM_005601 | chr19 | 56568136 | 56568618 | -605 | 0.72 | 0.31 | 0.41 |
| *NKPD1* | NM_001131067 | chr19 | 50355169 | 50355673 | -1597 | 0.64 | 0.22 | 0.41 |
| *NKX6-3* | NM_152568 | chr8 | 41623907 | 41624397 | -117 | 0.58 | 0.26 | 0.32 |
| *NLGN3* | NM_018977 | chrX | 70279494 | 70279992 | -1692 | 0.82 | 0.49 | 0.33 |
| *NLRX1* | NM_024618 | chr11 | 118543541 | 118544032 | -862 | 0.74 | 0.38 | 0.36 |
| *NMBR* | NM_002511 | chr6 | 142452985 | 142453525 | -1626 | 0.87 | 0.50 | 0.37 |
| *NMUR1* | NM_006056 | chr2 | 232103788 | 232104266 | -601 | 0.70 | 0.17 | 0.53 |
| *NNMT* | NM_006169 | chr11 | 113671001 | 113671279 | -604 | 0.74 | 0.37 | 0.37 |
| *NOD2* | NM_022162 | chr16 | 49287181 | 49287659 | -1130 | 0.59 | 0.09 | 0.51 |
| *NOL3* | NM_003946 | chr16 | 65764577 | 65765074 | -544 | 0.69 | 0.22 | 0.48 |
| *NOS2* | NM_000625 | chr17 | 23152028 | 23152323 | -493 | 0.89 | 0.20 | 0.70 |
| *NOS3* | NM_000603 | chr7 | 150317094 | 150317593 | -1735 | 0.53 | 0.23 | 0.30 |
| *NOSTRIN* | NM_001039724 | chr2 | 169367389 | 169367890 | 241 | 0.51 | 0.19 | 0.32 |
| *NOX1* | NM_007052 | chrX | 100015844 | 100016334 | -99 | 0.88 | 0.34 | 0.54 |
| *NPC1L1* | NM_001101648 | chr7 | 44547399 | 44547910 | -215 | 0.61 | 0.25 | 0.37 |
| *NPHP1* | NM_000272 | chr2 | 110320768 | 110321277 | -1094 | 0.38 | 0.06 | 0.32 |
| *NPL2* | NM_001134670 | chr10 | 99333608 | 99334100 | -237 | 0.60 | 0.21 | 0.39 |
| *NR0B2* | NM_021969 | chr1 | 27112993 | 27113517 | -101 | 0.76 | 0.38 | 0.39 |
| *NR1D1* | NM_021724 | chr17 | 35510953 | 35511454 | -704 | 0.74 | 0.44 | 0.30 |
| *NR1H3* | NM_001130101 | chr11 | 47235856 | 47236340 | 26 | 0.75 | 0.42 | 0.33 |
| *NR2E3* | NM_014249 | chr15 | 69889807 | 69890321 | 117 | 0.74 | 0.44 | 0.30 |
| *NR4A1* | NM_002135 | chr12 | 50729026 | 50729571 | -2158 | 0.78 | 0.48 | 0.30 |
| *NR5A2* | NM_003822 | chr1 | 198260971 | 198261215 | -2299 | 0.79 | 0.41 | 0.38 |
| *NRGN* | NM_001126181 | chr11 | 124112610 | 124113153 | -2156 | 0.85 | 0.48 | 0.37 |
| *NRXN2* | NM_015080 | chr11 | 64248597 | 64249099 | -1612 | 0.74 | 0.27 | 0.47 |
| *NT5C1A* | NM_032526 | chr1 | 39911124 | 39911626 | -1078 | 0.82 | 0.47 | 0.35 |
| *NTN5* | NM_145807 | chr19 | 53869440 | 53869918 | -1603 | 0.68 | 0.34 | 0.34 |
| *NUDT16* | NM_152395 | chr3 | 132582051 | 132582531 | -1105 | 0.91 | 0.35 | 0.56 |
| *NUDT4* | NM_019094 | chr12 | 92293427 | 92293771 | -2232 | 0.81 | 0.47 | 0.35 |
| *NUDT4P1* | NR_002212 | chr12 | 92293427 | 92293771 | -2277 | 0.81 | 0.47 | 0.35 |
| *NUMBL* | NM_004756 | chr19 | 45890360 | 45890844 | -2206 | 0.86 | 0.48 | 0.38 |
| *NUP155* | NM_004298 | chr5 | 37407308 | 37407806 | -913 | 0.64 | 0.34 | 0.30 |
| *NUP160* | NM_015231 | chr11 | 47828172 | 47828713 | -1809 | 0.89 | 0.56 | 0.33 |
| *NUP35* | NM_138285 | chr2 | 183695964 | 183696462 | -1114 | 0.77 | 0.46 | 0.31 |
| *NUP93* | NM_014669 | chr16 | 55319634 | 55320115 | -1642 | 0.94 | 0.61 | 0.33 |
| *NXT2* | NM_018698 | chrX | 108663789 | 108664275 | -1633 | 0.69 | 0.30 | 0.38 |
| *NYX* | NM_022567 | chrX | 41190511 | 41190987 | -907 | 0.81 | 0.38 | 0.43 |
| *OASL* | NM_003733 | chr12 | 119961114 | 119961608 | -198 | 0.80 | 0.43 | 0.38 |
| *OAT* | NM_000274 | chr10 | 126098851 | 126099360 | -1596 | 0.84 | 0.41 | 0.44 |
| *OBP2B* | NM_014581 | chr9 | 135075307 | 135075811 | -1110 | 0.65 | 0.34 | 0.31 |
| *OCA2* | NM_000275 | chr15 | 26019967 | 26020491 | -2176 | 0.69 | 0.32 | 0.37 |
| *OCIAD2* | NM_001014446 | chr4 | 48604701 | 48605209 | -1383 | 0.67 | 0.30 | 0.37 |
| *ODF2L* | NM_001007022 | chr1 | 86636401 | 86636895 | -2057 | 0.73 | 0.35 | 0.37 |
| *ODF3* | NM_053280 | chr11 | 185838 | 186296 | -693 | 0.66 | 0.33 | 0.33 |
| *ODF3L1* | NM_175881 | chr15 | 73801398 | 73801882 | -1733 | 0.71 | 0.24 | 0.47 |
| *ODF4* | NM_153007 | chr17 | 8181672 | 8182163 | -1994 | 0.89 | 0.43 | 0.46 |
| *ODZ1* | NM_014253 | chrX | 123925716 | 123926206 | -614 | 0.73 | 0.34 | 0.38 |
| *OFCC1* | NM_153003 | chr6 | 10169306 | 10169863 | -676 | 0.85 | 0.43 | 0.42 |
| *OLFML3* | NM_020190 | chr1 | 114321137 | 114321691 | -2138 | 0.60 | 0.29 | 0.31 |
| *OMG* | NM_002544 | chr17 | 26649403 | 26649880 | -1135 | 0.94 | 0.62 | 0.32 |
| *OMP* | NM_006189 | chr11 | 76490640 | 76491140 | -643 | 0.72 | 0.42 | 0.30 |
| *OPALIN* | NM_001040102 | chr10 | 98110446 | 98110927 | -1604 | 0.86 | 0.51 | 0.36 |
| *OR10C1* | NM_013941 | chr6 | 29513832 | 29514313 | -1698 | 0.90 | 0.54 | 0.36 |
| *OR10H4* | NM_001004465 | chr19 | 15918935 | 15919348 | -1675 | 0.86 | 0.54 | 0.32 |
| *OR10J1* | NM_012351 | chr1 | 157674802 | 157675307 | -1117 | 0.76 | 0.38 | 0.38 |
| *OR12D3* | NM_030959 | chr6 | 29452546 | 29453051 | -1751 | 0.90 | 0.50 | 0.40 |
| *OR13A1* | NM_001004297 | chr10 | 45132180 | 45132723 | -1389 | 0.84 | 0.42 | 0.42 |
| *OR13J1* | NM_001004487 | chr9 | 35862772 | 35862862 | -2419 | 0.82 | 0.31 | 0.51 |
| *OR1D2* | NM_002548 | chr17 | 2943505 | 2944004 | -714 | 0.80 | 0.46 | 0.34 |
| *OR1I1* | NM_001004713 | chr19 | 15056951 | 15057493 | -1654 | 0.72 | 0.40 | 0.32 |
| *OR2AG1* | NM_001004489 | chr11 | 6761854 | 6762357 | -738 | 0.82 | 0.48 | 0.33 |
| *OR2AK2* | NM_001004491 | chr1 | 246194696 | 246195192 | -312 | 0.70 | 0.36 | 0.34 |
| *OR2B6* | NM_012367 | chr6 | 28032038 | 28032415 | -770 | 0.66 | 0.31 | 0.35 |
| *OR2L1P* | NR_002145 | chr1 | 246218729 | 246219264 | -1194 | 0.84 | 0.47 | 0.37 |
| *OR2L2* | NM_001004686 | chr1 | 246265658 | 246266140 | -2197 | 0.68 | 0.37 | 0.32 |
| *OR4C11* | NM_001004700 | chr11 | 55130335 | 55130822 | -2153 | 0.58 | 0.28 | 0.30 |
| *OR4X2* | NM_001004727 | chr11 | 48221863 | 48222272 | -1163 | 0.57 | 0.19 | 0.38 |
| *OR51G2* | NM_001005238 | chr11 | 4894329 | 4894808 | -1099 | 0.86 | 0.24 | 0.62 |
| *OR51S1* | NM_001004758 | chr11 | 4828483 | 4829003 | -1729 | 0.75 | 0.36 | 0.39 |
| *OR52A1* | NM_012375 | chr11 | 5130672 | 5131160 | -741 | 0.86 | 0.51 | 0.34 |
| *OR52I1* | NM_001005169 | chr11 | 4570970 | 4571481 | -618 | 0.78 | 0.26 | 0.52 |
| *OR52N2* | NM_001005174 | chr11 | 5797203 | 5797686 | -696 | 0.82 | 0.41 | 0.41 |
| *OR5AC2* | NM_054106 | chr3 | 99286275 | 99286826 | -2155 | 0.86 | 0.47 | 0.39 |
| *OR5AN1* | NM_001004729 | chr11 | 58886083 | 58886635 | -2148 | 0.54 | 0.14 | 0.40 |
| *OR5B3* | NM_001005469 | chr11 | 57927814 | 57928307 | -602 | 0.60 | 0.20 | 0.40 |
| *OR5J2* | NM_001005492 | chr11 | 55699195 | 55699705 | -1219 | 0.96 | 0.42 | 0.54 |
| *OR5L1* | NM_001004738 | chr11 | 55334607 | 55335109 | -660 | 0.58 | 0.07 | 0.51 |
| *OR5M1* | NM_001004740 | chr11 | 56138917 | 56139400 | -1604 | 0.90 | 0.23 | 0.67 |
| *OR5R1* | NM_001004744 | chr11 | 55942631 | 55943146 | -604 | 0.93 | 0.59 | 0.34 |
| *OR6C6* | NM_001005493 | chr12 | 53976130 | 53976651 | -1107 | 0.82 | 0.41 | 0.40 |
| *OR6Q1* | NM_001005186 | chr11 | 57553135 | 57553623 | -1621 | 0.65 | 0.19 | 0.46 |
| *OR6W1P* | NR_002140 | chr7 | 142472418 | 142472959 | -1684 | 0.85 | 0.51 | 0.34 |
| *OR6X1* | NM_001005188 | chr11 | 123131897 | 123132377 | -1701 | 0.96 | 0.59 | 0.37 |
| *OR7D4* | NM_001005191 | chr19 | 9188716 | 9188926 | -2308 | 0.79 | 0.46 | 0.33 |
| *OR8H1* | NM_001005199 | chr11 | 55817192 | 55817581 | -2272 | 0.61 | 0.29 | 0.32 |
| *OR8H2* | NM_001005200 | chr11 | 55627113 | 55627631 | -1722 | 0.80 | 0.31 | 0.50 |
| *OR8K3* | NM_001005202 | chr11 | 55839924 | 55840424 | -2184 | 0.75 | 0.33 | 0.42 |
| *OR8K5* | NM_001004058 | chr11 | 55685338 | 55685841 | -1220 | 0.88 | 0.33 | 0.55 |
| *OR8U1* | NM_001005204 | chr11 | 55898294 | 55898792 | -1132 | 0.59 | 0.29 | 0.30 |
| *OR8U8* | NM_001013356 | chr11 | 55898294 | 55898792 | -1132 | 0.59 | 0.29 | 0.30 |
| *OR9I1* | NM_001005211 | chr11 | 57644443 | 57644948 | -1203 | 0.63 | 0.28 | 0.35 |
| *ORM2* | NM_000608 | chr9 | 116129521 | 116130070 | -2093 | 0.49 | 0.19 | 0.30 |
| *OSBPL1A* | NM_080597 | chr18 | 20232627 | 20233096 | -1073 | 0.72 | 0.34 | 0.38 |
| *OSBPL7* | NM_145798 | chr17 | 43256116 | 43256608 | -2216 | 0.66 | 0.33 | 0.32 |
| *OSTM1* | NM_014028 | chr6 | 108504019 | 108504500 | -1625 | 0.79 | 0.49 | 0.30 |
| *OTC* | NM_000531 | chrX | 38094239 | 38094704 | -2207 | 0.83 | 0.50 | 0.32 |
| *OTOF* | NM_004802 | chr2 | 26554271 | 26554771 | -107 | 0.90 | 0.57 | 0.33 |
| *OXSM* | NM_001145391 | chr3 | 25804687 | 25805186 | -1629 | 0.73 | 0.43 | 0.30 |
| *P2RX6* | NM_001159554 | chr22 | 19699117 | 19699623 | -71 | 0.50 | 0.16 | 0.34 |
| *P2RY10* | NM_014499 | chrX | 78085049 | 78085595 | -2162 | 0.89 | 0.55 | 0.34 |
| *P2RY2* | NM_002564 | chr11 | 72606045 | 72606537 | -700 | 0.68 | 0.37 | 0.30 |
| *P2RY4* | NM_002565 | chrX | 69396218 | 69396742 | -101 | 0.82 | 0.30 | 0.52 |
| *PABPC4L* | NM_001114734 | chr4 | 135343721 | 135344218 | -1616 | 0.82 | 0.26 | 0.56 |
| *PAGE3* | NM_001017931 | chrX | 55308365 | 55308868 | -612 | 0.85 | 0.50 | 0.35 |
| *PAK3* | NM_001128166 | chrX | 110072784 | 110073290 | -1131 | 0.89 | 0.28 | 0.62 |
| *PAK6* | NM_020168 | chr15 | 38317887 | 38318387 | -1189 | 0.60 | 0.18 | 0.41 |
| *PAPPA* | NM_002581 | chr9 | 117955545 | 117956065 | -86 | 0.80 | 0.47 | 0.33 |
| *PAQR5* | NM_017705 | chr15 | 67376467 | 67376983 | -1622 | 0.88 | 0.56 | 0.32 |
| *PAQR6* | NM_024897 | chr1 | 154485932 | 154486448 | -1723 | 0.83 | 0.32 | 0.51 |
| *PAQR7* | NM_178422 | chr1 | 26071272 | 26071758 | -1184 | 0.83 | 0.49 | 0.34 |
| *PAR4* | NR_022010 | chr15 | 23005116 | 23005638 | -2554 | 0.66 | 0.22 | 0.44 |
| *PAX8* | NM_003466 | chr2 | 113755132 | 113755446 | -2321 | 0.71 | 0.30 | 0.41 |
| *PBX2* | NM_002586 | chr6 | 32267230 | 32267750 | -1549 | 0.75 | 0.24 | 0.51 |
| *PBXIP1* | NM_020524 | chr1 | 153196655 | 153197154 | -1713 | 0.73 | 0.18 | 0.55 |
| *PC* | NM_022172 | chr11 | 66431580 | 66432111 | 70 | 0.86 | 0.51 | 0.35 |
| *PCBD1* | NM_000281 | chr10 | 72319277 | 72319771 | -977 | 0.92 | 0.35 | 0.56 |
| *PCBP4* | NM_033008 | chr3 | 51978396 | 51978886 | -2132 | 0.91 | 0.24 | 0.68 |
| *PCDH12* | NM_016580 | chr5 | 141321159 | 141321268 | -2402 | 0.54 | 0.16 | 0.38 |
| *PCDH21* | NM_033100 | chr10 | 85943574 | 85944080 | -669 | 0.70 | 0.35 | 0.35 |
| *PCDHA1* | NM_018900 | chr5 | 140144736 | 140145248 | -1067 | 0.85 | 0.48 | 0.36 |
| *PCDHA10* | NM_018901 | chr5 | 140213923 | 140214415 | -1648 | 0.62 | 0.24 | 0.37 |
| *PCDHA2* | NM_018905 | chr5 | 140154053 | 140154598 | -301 | 0.70 | 0.38 | 0.32 |
| *PCDHA5* | NM_018908 | chr5 | 140179686 | 140180158 | -1622 | 0.86 | 0.50 | 0.36 |
| *PCDHGA12* | NM_003735 | chr5 | 140789595 | 140790086 | -500 | 0.85 | 0.43 | 0.42 |
| *PCDHGA5* | NM_018918 | chr5 | 140722885 | 140723391 | -943 | 0.60 | 0.22 | 0.37 |
| *PCDHGA6* | NM_018919 | chr5 | 140732362 | 140732881 | -1212 | 0.67 | 0.15 | 0.52 |
| *PCDHGA8* | NM_014004 | chr5 | 140750114 | 140750617 | -1300 | 0.93 | 0.53 | 0.41 |
| *PCDHGA9* | NM_018921 | chr5 | 140761829 | 140762363 | -607 | 0.76 | 0.43 | 0.33 |
| *PCDHGB1* | NM_018922 | chr5 | 140708143 | 140708632 | -1623 | 0.89 | 0.54 | 0.35 |
| *PCDHGB3* | NM_018924 | chr5 | 140729328 | 140729808 | -577 | 0.59 | 0.21 | 0.38 |
| *PCDHGB6* | NM_018926 | chr5 | 140766293 | 140766797 | -1408 | 0.85 | 0.43 | 0.42 |
| *PCDHGB8P* | NR_001297 | chr5 | 140783652 | 140784181 | -2119 | 0.82 | 0.43 | 0.39 |
| *PCDHGC4* | NM_018928 | chr5 | 140842486 | 140842974 | -2194 | 0.93 | 0.59 | 0.34 |
| *PCDHGC5* | NM_018929 | chr5 | 140848110 | 140848616 | -628 | 0.69 | 0.37 | 0.32 |
| *PCGF3* | NM_006315 | chr4 | 687581 | 688081 | -1741 | 0.71 | 0.34 | 0.38 |
| *PCOLCE* | NM_002593 | chr7 | 100036848 | 100037226 | -780 | 0.67 | 0.27 | 0.40 |
| *PCSK1* | NM_000439 | chr5 | 95795676 | 95796147 | -1203 | 0.49 | 0.16 | 0.34 |
| *PCTK3* | NM_002596 | chr1 | 203739408 | 203739868 | -711 | 0.81 | 0.41 | 0.40 |
| *PCYT1B* | NM_004845 | chrX | 24577152 | 24577588 | -2096 | 0.78 | 0.39 | 0.39 |
| *PDE2A* | NM_001146209 | chr11 | 72060095 | 72060195 | -2389 | 0.82 | 0.27 | 0.55 |
| *PDE4C* | NM_000923 | chr19 | 18222148 | 18222441 | -2284 | 0.44 | 0.12 | 0.32 |
| *PDE6C* | NM_006204 | chr10 | 95360418 | 95360814 | -1718 | 0.67 | 0.33 | 0.35 |
| *PDE6D* | NM_002601 | chr2 | 232357138 | 232357613 | -3157 | 0.74 | 0.43 | 0.31 |
| *PDE6G* | NM_002602 | chr17 | 77233864 | 77234362 | -101 | 0.59 | 0.22 | 0.37 |
| *PDGFD* | NM_025208 | chr11 | 103542112 | 103542610 | -2124 | 0.63 | 0.28 | 0.35 |
| *PDHB* | NM_000925 | chr3 | 58395944 | 58396221 | -1477 | 0.85 | 0.34 | 0.50 |
| *PDLIM1* | NM_020992 | chr10 | 97042070 | 97042610 | -1569 | 0.87 | 0.53 | 0.35 |
| *PDLIM2* | NM_021630 | chr8 | 22490168 | 22490711 | -2147 | 0.78 | 0.45 | 0.33 |
| *PDLIM4* | NM_001131027 | chr5 | 131618809 | 131619264 | -2212 | 0.66 | 0.31 | 0.35 |
| *PDP2* | NM_020786 | chr16 | 65471020 | 65471530 | -661 | 0.67 | 0.29 | 0.38 |
| *PDYN* | NM_024411 | chr20 | 1924652 | 1925172 | -2210 | 0.73 | 0.32 | 0.41 |
| *PDZD3* | NM_024791 | chr11 | 118559417 | 118559937 | -1727 | 0.69 | 0.34 | 0.35 |
| *PDZK1IP1* | NM_005764 | chr1 | 47429765 | 47430247 | -1648 | 0.75 | 0.29 | 0.46 |
| *PEMT* | NM_007169 | chr17 | 17426311 | 17426809 | -90 | 0.72 | 0.33 | 0.39 |
| *PEPD* | NM_000285 | chr19 | 38705082 | 38705626 | -713 | 0.57 | 0.25 | 0.32 |
| *PEX11A* | NM_003847 | chr15 | 88036096 | 88036597 | -1384 | 0.67 | 0.29 | 0.38 |
| *PF4V1* | NM_002620 | chr4 | 74935511 | 74936013 | -2114 | 0.89 | 0.32 | 0.58 |
| *PFKFB1* | NM_002625 | chrX | 55038600 | 55039102 | -1615 | 0.87 | 0.26 | 0.60 |
| *PFKFB4* | NM_004567 | chr3 | 48571025 | 48571521 | -2042 | 0.52 | 0.21 | 0.31 |
| *PGAM2* | NM_000290 | chr7 | 44072051 | 44072527 | -601 | 0.73 | 0.22 | 0.51 |
| *PGC* | NM_002630 | chr6 | 41824438 | 41824738 | -1489 | 0.85 | 0.46 | 0.39 |
| *PGLYRP2* | NM_052890 | chr19 | 15451756 | 15452254 | -690 | 0.71 | 0.19 | 0.53 |
| *PHACTR2* | NM_001100164 | chr6 | 144039435 | 144039968 | -1092 | 0.79 | 0.42 | 0.38 |
| *PHACTR4* | NM_023923 | chr1 | 28636764 | 28637260 | -235 | 0.85 | 0.44 | 0.41 |
| *PHF11* | NM_001040443 | chr13 | 48965840 | 48966320 | -1721 | 0.82 | 0.52 | 0.30 |
| *PHTF2* | NM_001127357 | chr7 | 77304959 | 77305512 | -2146 | 0.83 | 0.47 | 0.36 |
| *PHYHD1* | NM_001100876 | chr9 | 130721029 | 130721554 | -1702 | 0.93 | 0.56 | 0.37 |
| *PHYHIP* | NM_001099335 | chr8 | 22145661 | 22146185 | -127 | 0.70 | 0.28 | 0.42 |
| *PI4K2B* | NM_018323 | chr4 | 24842837 | 24843373 | -1645 | 0.82 | 0.44 | 0.38 |
| *PI4KA* | NM_002650 | chr22 | 19419806 | 19420318 | -1107 | 0.74 | 0.34 | 0.41 |
| *PICK1* | NM_001039583 | chr22 | 36782225 | 36782741 | -724 | 0.67 | 0.20 | 0.47 |
| *PID1* | NM_001100818 | chr2 | 229845764 | 229846243 | -1702 | 0.68 | 0.35 | 0.32 |
| *PIF1* | NM_025049 | chr15 | 62905665 | 62906116 | -999 | 0.68 | 0.33 | 0.34 |
| *PIGB* | NM_004855 | chr15 | 53397150 | 53397536 | -1081 | 0.57 | 0.24 | 0.33 |
| *PIGK* | NM_005482 | chr1 | 77458639 | 77459130 | -1164 | 0.84 | 0.35 | 0.49 |
| *PIGQ* | NM_004204 | chr16 | 558641 | 559151 | -1108 | 0.81 | 0.47 | 0.34 |
| *PIGR* | NM_002644 | chr1 | 205187378 | 205187917 | -1217 | 0.85 | 0.41 | 0.44 |
| *PIGV* | NM_017837 | chr1 | 26985096 | 26985583 | -1732 | 0.73 | 0.43 | 0.30 |
| *PIGY* | NM_001042616 | chr4 | 89665444 | 89665953 | -1720 | 0.84 | 0.46 | 0.37 |
| *PIK3C2A* | NM_002645 | chr11 | 17148423 | 17148914 | -738 | 0.85 | 0.45 | 0.40 |
| *PIK3C2G* | NM_004570 | chr12 | 18303319 | 18303861 | -2150 | 0.86 | 0.38 | 0.48 |
| *PIK3CB* | NM_006219 | chr3 | 139962604 | 139963011 | -1932 | 0.68 | 0.17 | 0.51 |
| *PIK3CD* | NM_005026 | chr1 | 9632165 | 9632636 | -1975 | 0.86 | 0.50 | 0.35 |
| *PIK3R5* | NM_014308 | chr17 | 8757913 | 8758423 | -1609 | 0.81 | 0.43 | 0.38 |
| *PILRA* | NM_013439 | chr7 | 99808311 | 99808805 | -445 | 0.75 | 0.35 | 0.40 |
| *PILRB* | NM_178238 | chr7 | 99792373 | 99792871 | -939 | 0.88 | 0.53 | 0.35 |
| *PIRT* | NM_001101387 | chr17 | 10682605 | 10683087 | -703 | 0.63 | 0.20 | 0.43 |
| *PISD* | NM_014338 | chr22 | 30357265 | 30357759 | -702 | 0.78 | 0.36 | 0.43 |
| *PITPNM2* | NM_020845 | chr12 | 122161768 | 122162290 | -1101 | 0.71 | 0.35 | 0.36 |
| *PKLR* | NM_000298 | chr1 | 153539418 | 153539970 | -1845 | 0.87 | 0.47 | 0.39 |
| *PKN1* | NM_213560 | chr19 | 14410794 | 14411331 | -1022 | 0.64 | 0.14 | 0.51 |
| *PKP2* | NM_001005242 | chr12 | 32942014 | 32942537 | -1228 | 0.90 | 0.49 | 0.41 |
| *PLA2G12B* | NM_032562 | chr10 | 74385864 | 74386231 | -1531 | 0.65 | 0.30 | 0.35 |
| *PLA2G1B* | NM_000928 | chr12 | 119250012 | 119250304 | -183 | 0.92 | 0.62 | 0.30 |
| *PLA2G2F* | NM_022819 | chr1 | 20336540 | 20337040 | -1619 | 0.57 | 0.13 | 0.44 |
| *PLA2G3* | NM_015715 | chr22 | 29868434 | 29868956 | -2226 | 0.77 | 0.34 | 0.43 |
| *PLA2G4B* | NM_001114633 | chr15 | 39917911 | 39918387 | -153 | 0.84 | 0.28 | 0.56 |
| *PLA2G4C* | NM_001159322 | chr19 | 53307101 | 53307585 | -1422 | 0.79 | 0.49 | 0.30 |
| *PLA2G4D* | NM_178034 | chr15 | 40173992 | 40174506 | -205 | 0.74 | 0.34 | 0.41 |
| *PLA2G4E* | NM_001080490 | chr15 | 40091384 | 40091876 | -1893 | 0.74 | 0.29 | 0.45 |
| *PLA2R1* | NM_001007267 | chr2 | 160629219 | 160629739 | -2112 | 0.55 | 0.20 | 0.36 |
| *PLAC1* | NM_021796 | chrX | 133621589 | 133621771 | -1501 | 0.76 | 0.46 | 0.30 |
| *PLAC4* | NM_182832 | chr21 | 41480387 | 41480875 | -1595 | 0.73 | 0.31 | 0.43 |
| *PLB1* | NM_153021 | chr2 | 28571066 | 28571596 | -1154 | 0.67 | 0.25 | 0.42 |
| *PLBD1* | NM_024829 | chr12 | 14613355 | 14613872 | -1555 | 0.64 | 0.24 | 0.40 |
| *PLCB2* | NM_004573 | chr15 | 38388368 | 38388860 | -1148 | 0.55 | 0.21 | 0.34 |
| *PLCD1* | NM_006225 | chr3 | 38047449 | 38047947 | -1540 | 0.92 | 0.40 | 0.52 |
| *PLCD4* | NM_032726 | chr2 | 219179308 | 219179791 | -1325 | 0.62 | 0.31 | 0.31 |
| *PLCH2* | NM_014638 | chr1 | 2396122 | 2396632 | -1236 | 0.67 | 0.31 | 0.35 |
| *PLD4* | NM_138790 | chr14 | 104461164 | 104461640 | -829 | 0.85 | 0.49 | 0.36 |
| *PLEK2* | NM_016445 | chr14 | 66950094 | 66950570 | -1751 | 0.83 | 0.52 | 0.30 |
| *PLEKHA1* | NM_021622 | chr10 | 124140407 | 124140877 | -1190 | 0.97 | 0.53 | 0.44 |
| *PLEKHA4* | NM_020904 | chr19 | 54064031 | 54064530 | -610 | 0.80 | 0.41 | 0.39 |
| *PLEKHA6* | NM_014935 | chr1 | 202597041 | 202597509 | -1608 | 0.71 | 0.29 | 0.42 |
| *PLEKHF1* | NM_024310 | chr19 | 34846780 | 34847286 | -1133 | 0.68 | 0.31 | 0.36 |
| *PLEKHG4* | NM_001129727 | chr16 | 65868022 | 65868514 | -1308 | 0.77 | 0.33 | 0.44 |
| *PLEKHG5* | NM_198681 | chr1 | 6503035 | 6503444 | -583 | 0.90 | 0.25 | 0.65 |
| *PLEKHG6* | NM_001144857 | chr12 | 6291566 | 6292062 | -263 | 0.78 | 0.39 | 0.39 |
| *PLIN* | NM_001145311 | chr15 | 88023595 | 88024128 | -209 | 0.42 | 0.11 | 0.32 |
| *PLK3* | NM_004073 | chr1 | 45036581 | 45037120 | -1771 | 0.77 | 0.47 | 0.31 |
| *PLK4* | NM_014264 | chr4 | 129020184 | 129020457 | -1173 | 0.78 | 0.48 | 0.30 |
| *PLN* | NM_002667 | chr6 | 118975257 | 118975781 | -615 | 0.83 | 0.38 | 0.46 |
| *PLOD2* | NM_000935 | chr3 | 147362672 | 147363166 | -947 | 0.65 | 0.32 | 0.33 |
| *PLS3* | NM_001136025 | chrX | 114733294 | 114733765 | -545 | 0.80 | 0.34 | 0.46 |
| *PLVAP* | NM_031310 | chr19 | 17350847 | 17351351 | -1962 | 0.62 | 0.24 | 0.37 |
| *PLXNA1* | NM_032242 | chr3 | 128188808 | 128189304 | -1135 | 0.81 | 0.14 | 0.67 |
| *PLXNB2* | NM_012401 | chr22 | 49075299 | 49075582 | -104 | 0.67 | 0.22 | 0.46 |
| *PM20D1* | NM_152491 | chr1 | 204087263 | 204087743 | -1635 | 0.91 | 0.45 | 0.47 |
| *PMCH* | NM_002674 | chr12 | 101115606 | 101116114 | -116 | 0.55 | 0.22 | 0.33 |
| *PNLIPRP1* | NM_006229 | chr10 | 118339920 | 118340392 | -323 | 0.75 | 0.41 | 0.35 |
| *PNMA5* | NM_001103150 | chrX | 151911774 | 151912260 | -600 | 0.77 | 0.25 | 0.52 |
| *PNPLA3* | NM_025225 | chr22 | 42648961 | 42649461 | -1740 | 0.73 | 0.32 | 0.41 |
| *PNPLA5* | NM_138814 | chr22 | 42621121 | 42621629 | -2185 | 0.68 | 0.32 | 0.36 |
| *POL3S* | NM_001039503 | chr16 | 31009336 | 31009830 | -1952 | 0.75 | 0.30 | 0.44 |
| *POLB* | NM_002690 | chr8 | 42312769 | 42313306 | -2148 | 0.84 | 0.53 | 0.30 |
| *POLD1* | NM_002691 | chr19 | 55578270 | 55578784 | -877 | 0.68 | 0.32 | 0.35 |
| *POLD4* | NM_021173 | chr11 | 66879534 | 66880032 | -2190 | 0.96 | 0.44 | 0.51 |
| *POLR2E* | NM_002695 | chr19 | 1047649 | 1048106 | -1486 | 0.76 | 0.45 | 0.31 |
| *POP4* | NM_006627 | chr19 | 34787753 | 34788217 | -1024 | 0.80 | 0.46 | 0.34 |
| *POPDC3* | NR_024539 | chr6 | 105730438 | 105730971 | 118 | 0.82 | 0.45 | 0.37 |
| *POR* | NM_000941 | chr7 | 75379934 | 75380375 | -2200 | 0.90 | 0.59 | 0.31 |
| *POU2AF1* | NM_006235 | chr11 | 110755830 | 110756316 | -706 | 0.83 | 0.48 | 0.35 |
| *POU5F1* | NM_002701 | chr6 | 31247934 | 31248444 | -1759 | 0.80 | 0.29 | 0.50 |
| *POU6F1* | NM_002702 | chr12 | 49879720 | 49880214 | -1750 | 0.92 | 0.56 | 0.35 |
| *PP14571* | NR_024014 | chr2 | 241046397 | 241046921 | -1869 | 0.72 | 0.41 | 0.31 |
| *PPAPDC1B* | NM_001102559 | chr8 | 38246717 | 38247216 | -1071 | 0.78 | 0.44 | 0.35 |
| *PPBPL1* | NM_001144760 | chr4 | 74931257 | 74931760 | -937 | 0.60 | 0.30 | 0.30 |
| *PPIA* | NM_021130 | chr7 | 44800858 | 44801207 | -1732 | 0.89 | 0.58 | 0.31 |
| *PPID* | NM_005038 | chr4 | 159865505 | 159865796 | -1648 | 0.80 | 0.47 | 0.33 |
| *PPIF* | NM_005729 | chr10 | 80775734 | 80776246 | -1235 | 0.87 | 0.49 | 0.38 |
| *PPL* | NM_002705 | chr16 | 4927627 | 4928035 | -694 | 0.59 | 0.29 | 0.31 |
| *PPM1F* | NM_014634 | chr22 | 20638928 | 20639428 | -1961 | 0.64 | 0.24 | 0.40 |
| *PPP1CA* | NM_001008709 | chr11 | 66927744 | 66928232 | -2036 | 0.68 | 0.38 | 0.30 |
| *PPP1R15A* | NM_014330 | chr19 | 54064031 | 54064530 | -3179 | 0.80 | 0.41 | 0.39 |
| *PPP1R16A* | NM_032902 | chr8 | 145690775 | 145691300 | -1878 | 0.83 | 0.35 | 0.48 |
| *PPP1R1A* | NM_006741 | chr12 | 53270651 | 53271149 | -2190 | 0.77 | 0.36 | 0.41 |
| *PPP1R3F* | NM_033215 | chrX | 49010042 | 49010546 | -2966 | 0.73 | 0.29 | 0.44 |
| *PPP2R3A* | NM_002718 | chr3 | 137164837 | 137165194 | -2240 | 0.85 | 0.41 | 0.44 |
| *PPP2R5B* | NM_006244 | chr11 | 64446993 | 64447505 | -1506 | 0.64 | 0.33 | 0.31 |
| *PPP6C* | NM_001123355 | chr9 | 126993152 | 126993589 | -1331 | 0.78 | 0.42 | 0.36 |
| *PPY* | NM_002722 | chr17 | 39375204 | 39375710 | -98 | 0.70 | 0.26 | 0.45 |
| *PPY2* | NR_002181 | chr17 | 23598381 | 23598929 | 59 | 0.73 | 0.42 | 0.31 |
| *PRAF2* | NM_007213 | chrX | 48820469 | 48820945 | -2101 | 0.65 | 0.29 | 0.37 |
| *PRAMEF16* | NM_001045480 | chr1 | 13367739 | 13368150 | 104 | 0.91 | 0.53 | 0.38 |
| *PRAMEF17* | NM_001099851 | chr1 | 13586557 | 13586789 | -2001 | 0.97 | 0.65 | 0.31 |
| *PRAMEF21* | NM_001100114 | chr1 | 13614048 | 13614562 | -1083 | 0.89 | 0.57 | 0.32 |
| *PRAMEF7* | NM_001012277 | chr1 | 13485918 | 13486432 | -3101 | 0.54 | 0.12 | 0.42 |
| *PRAMEF8* | NM_001012276 | chr1 | 13485918 | 13486432 | -2038 | 0.54 | 0.12 | 0.42 |
| *PRICKLE3* | NM_006150 | chrX | 48931840 | 48932142 | -2271 | 0.91 | 0.45 | 0.46 |
| *PRKACA* | NM_207518 | chr19 | 14086953 | 14087438 | -1203 | 0.70 | 0.39 | 0.30 |
| *PRKACB* | NM_002731 | chr1 | 84314371 | 84314856 | -1718 | 0.87 | 0.57 | 0.30 |
| *PRO0611* | NR_002762 | chr1 | 31233484 | 31233980 | 234 | 0.47 | 0.06 | 0.40 |
| *PROCA1* | NM_152465 | chr17 | 24063747 | 24064247 | -998 | 0.85 | 0.50 | 0.35 |
| *PRODH2* | NM_021232 | chr19 | 40997655 | 40997921 | -1747 | 0.85 | 0.48 | 0.37 |
| *PROK1* | NM_032414 | chr1 | 110794820 | 110795314 | -243 | 0.92 | 0.55 | 0.37 |
| *PROM2* | NM_144707 | chr2 | 95302061 | 95302537 | -1628 | 0.86 | 0.50 | 0.37 |
| *ProSAPiP1* | NM_014731 | chr20 | 3098052 | 3098597 | -1117 | 0.82 | 0.39 | 0.44 |
| *PRR5* | NM_001017530 | chr22 | 43451051 | 43451547 | -52 | 0.67 | 0.34 | 0.33 |
| *PRSS22* | NM_022119 | chr16 | 2850136 | 2850652 | -2222 | 0.68 | 0.32 | 0.36 |
| *PRSS23* | NM_007173 | chr11 | 86187813 | 86188294 | -1084 | 0.82 | 0.43 | 0.40 |
| *PRSS35* | NM_153362 | chr6 | 84276572 | 84276924 | -2244 | 0.93 | 0.37 | 0.57 |
| *PRSS36* | NM_173502 | chr16 | 31068763 | 31069275 | -103 | 0.87 | 0.37 | 0.49 |
| *PRX* | NM_020956 | chr19 | 45612321 | 45612801 | -1450 | 0.60 | 0.30 | 0.30 |
| *PSD4* | NM_012455 | chr2 | 113645614 | 113646166 | -2140 | 0.73 | 0.34 | 0.39 |
| *PSG5* | NM_001130014 | chr19 | 48383167 | 48383703 | -907 | 0.48 | 0.15 | 0.33 |
| *PSMB7* | NM_002799 | chr9 | 126218394 | 126218702 | -1006 | 0.62 | 0.28 | 0.34 |
| *PSMD10* | NM_002814 | chrX | 107222389 | 107222929 | -1155 | 0.81 | 0.38 | 0.43 |
| *PSMD12* | NM_002816 | chr17 | 62794829 | 62795381 | -1922 | 0.80 | 0.35 | 0.45 |
| *PTGER1* | NM_000955 | chr19 | 14447631 | 14448127 | -705 | 0.64 | 0.29 | 0.35 |
| *PTGIR* | NM_000960 | chr19 | 51820385 | 51820903 | -450 | 0.54 | 0.20 | 0.35 |
| *PTGS1* | NM_000962 | chr9 | 124170626 | 124171175 | -2148 | 0.82 | 0.23 | 0.59 |
| *PTH1R* | NM_000316 | chr3 | 46893349 | 46893873 | -628 | 0.84 | 0.32 | 0.52 |
| *PTPN20A* | NM_001042387 | chr10 | 48449991 | 48450376 | -2253 | 0.54 | 0.23 | 0.31 |
| *PTPN20B* | NM_001042358 | chr10 | 48449991 | 48450376 | -2596 | 0.54 | 0.23 | 0.31 |
| *PTPN3* | NM_001145369 | chr9 | 111253382 | 111253896 | -154 | 0.70 | 0.27 | 0.43 |
| *PTPN6* | NM_002831 | chr12 | 6928390 | 6928833 | -2082 | 0.82 | 0.40 | 0.42 |
| *PTPN7* | NM_002832 | chr1 | 200397698 | 200398182 | -1566 | 0.76 | 0.38 | 0.38 |
| *PTPRA* | NM_002836 | chr20 | 2790649 | 2791147 | -1942 | 0.69 | 0.33 | 0.35 |
| *PTPRCAP* | NM_005608 | chr11 | 66961573 | 66962069 | -92 | 0.76 | 0.39 | 0.37 |
| *PTPRN* | NM_002846 | chr2 | 219884404 | 219884797 | -2213 | 0.98 | 0.49 | 0.49 |
| *PVRL4* | NM_030916 | chr1 | 159327952 | 159328448 | -2191 | 0.81 | 0.45 | 0.36 |
| *PXDNL* | NM_144651 | chr8 | 52886495 | 52886993 | -2186 | 0.75 | 0.31 | 0.44 |
| *PYCARD* | NM_013258 | chr16 | 31123755 | 31124053 | -2152 | 0.87 | 0.47 | 0.40 |
| *PYCR1* | NM_006907 | chr17 | 77489952 | 77490444 | -1939 | 0.77 | 0.46 | 0.31 |
| *PYGL* | NM_002863 | chr14 | 50483072 | 50483471 | -2287 | 0.62 | 0.29 | 0.34 |
| *PYGM* | NM_005609 | chr11 | 64286400 | 64286901 | -1887 | 0.82 | 0.27 | 0.55 |
| *RAB17* | NM_022449 | chr2 | 238164437 | 238164931 | -209 | 0.53 | 0.20 | 0.33 |
| *RAB25* | NM_020387 | chr1 | 154297297 | 154297744 | -68 | 0.51 | 0.18 | 0.33 |
| *RAB26* | NM_014353 | chr16 | 2137518 | 2138030 | -877 | 0.68 | 0.33 | 0.35 |
| *RAB38* | NM_022337 | chr11 | 87549188 | 87549693 | -1193 | 0.77 | 0.40 | 0.38 |
| *RAB3B* | NM_002867 | chr1 | 52230250 | 52230768 | -1573 | 0.69 | 0.24 | 0.45 |
| *RAB3IL1* | NM_013401 | chr11 | 61443444 | 61443938 | -2118 | 0.46 | 0.16 | 0.30 |
| *RAB41* | NM_001032726 | chrX | 69416801 | 69417307 | -1738 | 0.82 | 0.30 | 0.51 |
| *RAB42* | NM_152304 | chr1 | 28790805 | 28791306 | -242 | 0.72 | 0.38 | 0.34 |
| *RAB5C* | NM_004583 | chr17 | 37561819 | 37562321 | -1522 | 0.77 | 0.47 | 0.31 |
| *RABAC1* | NM_006423 | chr19 | 47155709 | 47156015 | -494 | 0.66 | 0.16 | 0.51 |
| *RAD23A* | NM_005053 | chr19 | 12916272 | 12916769 | -1132 | 0.66 | 0.24 | 0.42 |
| *RAD54L2* | NM_015106 | chr3 | 51549694 | 51550206 | -685 | 0.83 | 0.53 | 0.30 |
| *RAET1L* | NM_130900 | chr6 | 150390297 | 150390775 | -2175 | 0.78 | 0.41 | 0.37 |
| *RALGDS* | NM_006266 | chr9 | 134988276 | 134988748 | -2130 | 0.83 | 0.53 | 0.31 |
| *RAMP3* | NM_005856 | chr7 | 45162076 | 45162596 | -1555 | 0.81 | 0.36 | 0.46 |
| *RAPGEFL1* | NM_016339 | chr17 | 35586136 | 35586647 | -1375 | 0.67 | 0.25 | 0.42 |
| *RAPSN* | NM_005055 | chr11 | 47428294 | 47428490 | -1086 | 0.74 | 0.08 | 0.66 |
| *RARG* | NM_000966 | chr12 | 51912653 | 51913165 | -606 | 0.62 | 0.30 | 0.32 |
| *RASAL1* | NM_004658 | chr12 | 112059993 | 112060486 | -1835 | 0.57 | 0.25 | 0.32 |
| *RASL10B* | NM_033315 | chr17 | 31080963 | 31081258 | -1680 | 0.84 | 0.54 | 0.30 |
| *RASL11A* | NM_206827 | chr13 | 26741523 | 26742025 | -689 | 0.67 | 0.21 | 0.46 |
| *RASL12* | NM_016563 | chr15 | 63148399 | 63148885 | -1201 | 0.75 | 0.35 | 0.40 |
| *RASSF2* | NM_014737 | chr20 | 4753687 | 4754170 | -1637 | 0.86 | 0.56 | 0.30 |
| *RASSF4* | NM_032023 | chr10 | 44772802 | 44773298 | -2174 | 0.81 | 0.48 | 0.34 |
| *RASSF9* | NM_005447 | chr12 | 84755838 | 84756370 | -1655 | 0.86 | 0.48 | 0.38 |
| *RBM44* | NM_001080504 | chr2 | 238370328 | 238370860 | -1532 | 0.72 | 0.33 | 0.39 |
| *RBM47* | NM_019027 | chr4 | 40213465 | 40213801 | -886 | 0.62 | 0.22 | 0.40 |
| *RBM9* | NM_001031695 | chr22 | 34568527 | 34569059 | -2217 | 0.87 | 0.42 | 0.45 |
| *RBP2* | NM_004164 | chr3 | 140677890 | 140678410 | -108 | 0.54 | 0.21 | 0.33 |
| *RBP3* | NM_002900 | chr10 | 48012845 | 48013343 | -2097 | 0.77 | 0.20 | 0.58 |
| *RBP5* | NM_031491 | chr12 | 7172846 | 7173364 | -372 | 0.86 | 0.30 | 0.56 |
| *RCCD1* | NM_001017919 | chr15 | 89297943 | 89298443 | -916 | 0.83 | 0.38 | 0.45 |
| *RDH16* | NM_003708 | chr12 | 55638036 | 55638580 | -623 | 0.67 | 0.32 | 0.35 |
| *REEP2* | NM_016606 | chr5 | 137801698 | 137802245 | -702 | 0.78 | 0.34 | 0.44 |
| *REG1A* | NM_002909 | chr2 | 79200276 | 79200758 | -574 | 0.64 | 0.20 | 0.43 |
| *REG3A* | NM_002580 | chr2 | 79240748 | 79241149 | -561 | 0.90 | 0.41 | 0.49 |
| *REM2* | NM_173527 | chr14 | 22422084 | 22422580 | 61 | 0.54 | 0.24 | 0.30 |
| *RENBP* | NM_002910 | chrX | 152865015 | 152865515 | -1839 | 0.71 | 0.30 | 0.40 |
| *RETNLB* | NM_032579 | chr3 | 109960689 | 109960967 | -2008 | 0.84 | 0.33 | 0.51 |
| *RFC5* | NM_001130112 | chr12 | 116936945 | 116937457 | -1689 | 0.81 | 0.50 | 0.31 |
| *RGN* | NM_004683 | chrX | 46821152 | 46821440 | -1422 | 0.75 | 0.37 | 0.38 |
| *RGR* | NM_001012720 | chr10 | 85994661 | 85995183 | 134 | 0.76 | 0.41 | 0.35 |
| *RGS12* | NM_198227 | chr4 | 3341055 | 3341586 | -200 | 0.87 | 0.35 | 0.52 |
| *RGS14* | NM_006480 | chr5 | 176716736 | 176717250 | -456 | 0.69 | 0.35 | 0.34 |
| *RGS16* | NM_002928 | chr1 | 180842519 | 180842620 | -2398 | 0.80 | 0.50 | 0.30 |
| *RGS18* | NM_130782 | chr1 | 190393896 | 190394396 | -68 | 0.85 | 0.31 | 0.54 |
| *RGS20* | NM_170587 | chr8 | 54926104 | 54926640 | -548 | 0.57 | 0.16 | 0.40 |
| *RGS3* | NM_130795 | chr9 | 115301911 | 115302403 | -1370 | 0.63 | 0.20 | 0.43 |
| *RGS7* | NM_002924 | chr1 | 239588971 | 239589464 | -2116 | 0.91 | 0.31 | 0.60 |
| *RGS8* | NM_001102450 | chr1 | 180910638 | 180911132 | -2195 | 0.79 | 0.24 | 0.54 |
| *RGSL1* | NM_001137669 | chr1 | 180684432 | 180684948 | -1188 | 0.89 | 0.49 | 0.39 |
| *RHBDF2* | NM_001005498 | chr17 | 72009542 | 72010064 | -700 | 0.71 | 0.40 | 0.31 |
| *RHCE* | NM_020485 | chr1 | 25622308 | 25622413 | -2410 | 0.82 | 0.39 | 0.43 |
| *RHO* | NM_000539 | chr3 | 130728702 | 130729184 | -1228 | 0.84 | 0.34 | 0.51 |
| *RIC3* | NM_001135109 | chr11 | 8148847 | 8149365 | -1940 | 0.99 | 0.50 | 0.50 |
| *RIN1* | NM_004292 | chr11 | 65860927 | 65861426 | -600 | 0.56 | 0.24 | 0.32 |
| *RIN2* | NM_018993 | chr20 | 19817497 | 19817986 | -467 | 0.81 | 0.30 | 0.51 |
| *RINL* | NM_198445 | chr19 | 44060613 | 44061109 | -127 | 0.78 | 0.32 | 0.46 |
| *RLBP1* | NM_000326 | chr15 | 87565522 | 87566065 | 132 | 0.60 | 0.30 | 0.31 |
| *RLBP1L1* | NM_173519 | chr8 | 62361650 | 62362173 | -1166 | 0.79 | 0.48 | 0.31 |
| *RNASE6* | NM_005615 | chr14 | 20319049 | 20319537 | 244 | 0.78 | 0.39 | 0.39 |
| *RND3* | NM_005168 | chr2 | 151053508 | 151054019 | -1337 | 0.74 | 0.31 | 0.43 |
| *RNF112* | NM_007148 | chr17 | 19253549 | 19254049 | -1316 | 0.84 | 0.50 | 0.34 |
| *RNF144B* | NM_182757 | chr6 | 18494546 | 18495063 | -767 | 0.85 | 0.48 | 0.38 |
| *RNF180* | NM_001113561 | chr5 | 63495545 | 63496043 | -1632 | 0.94 | 0.61 | 0.33 |
| *RNF19A* | NM_015435 | chr8 | 101386739 | 101387137 | -2275 | 0.88 | 0.52 | 0.36 |
| *RORC* | NM_001001523 | chr1 | 150065039 | 150065539 | -112 | 0.66 | 0.18 | 0.48 |
| *RPE65* | NM_000329 | chr1 | 68688090 | 68688593 | -111 | 0.77 | 0.46 | 0.32 |
| *RPH3AL* | NM_006987 | chr17 | 204037 | 204539 | -1712 | 0.64 | 0.34 | 0.30 |
| *RPL13AP6* | NR_026715 | chr10 | 112687901 | 112688413 | -1154 | 0.70 | 0.35 | 0.36 |
| *RPL36A* | NM_021029 | chrX | 100530380 | 100530889 | -1968 | 0.61 | 0.26 | 0.35 |
| *RPLP0P2* | NR_002775 | chr11 | 61137833 | 61138317 | -1008 | 0.76 | 0.14 | 0.62 |
| *RPLP1* | NM_001003 | chr15 | 67530529 | 67531042 | -1426 | 0.57 | 0.26 | 0.30 |
| *RPS15* | NM_001018 | chr19 | 1387419 | 1387902 | -1701 | 0.64 | 0.32 | 0.32 |
| *RPS19BP1* | NM_194326 | chr22 | 38261170 | 38261280 | -2419 | 0.59 | 0.27 | 0.32 |
| *RPS23* | NM_001025 | chr5 | 81610953 | 81611448 | -1209 | 0.79 | 0.20 | 0.59 |
| *RPS27L* | NM_015920 | chr15 | 61238286 | 61238759 | -1728 | 0.75 | 0.43 | 0.32 |
| *RPS4X* | NM_001007 | chrX | 71414590 | 71414882 | -870 | 0.68 | 0.36 | 0.32 |
| *RPS6KA4* | NM_001006944 | chr11 | 63881820 | 63882326 | -1127 | 0.69 | 0.40 | 0.30 |
| *RRAGB* | NM_006064 | chrX | 55759957 | 55760493 | -609 | 0.73 | 0.39 | 0.34 |
| *RRH* | NM_006583 | chr4 | 110967555 | 110968056 | -792 | 0.83 | 0.26 | 0.57 |
| *RRM1* | NM_001033 | chr11 | 4070533 | 4071040 | -1712 | 0.77 | 0.38 | 0.39 |
| *RRP12* | NM_001145114 | chr10 | 99152635 | 99152839 | -1620 | 0.71 | 0.24 | 0.47 |
| *RSPH9* | NM_152732 | chr6 | 43719946 | 43720459 | -584 | 0.80 | 0.21 | 0.59 |
| *RSPO4* | NM_001029871 | chr20 | 931350 | 931852 | -697 | 0.69 | 0.25 | 0.44 |
| *RTN4* | NM_007008 | chr2 | 55092125 | 55092632 | -1404 | 0.67 | 0.16 | 0.51 |
| *RUFY4* | NM_198483 | chr2 | 218645916 | 218646394 | -129 | 0.55 | 0.20 | 0.35 |
| *RUSC2* | NM_001135999 | chr9 | 35527564 | 35528090 | -801 | 0.91 | 0.19 | 0.72 |
| *RXFP1* | NM_021634 | chr4 | 159660629 | 159661114 | -1624 | 0.87 | 0.55 | 0.32 |
| *RXFP4* | NM_181885 | chr1 | 154177418 | 154177936 | -426 | 0.88 | 0.48 | 0.40 |
| *S100A1* | NM_006271 | chr1 | 151867305 | 151867809 | 61 | 0.63 | 0.30 | 0.33 |
| *S100A13* | NM_001024211 | chr1 | 151868815 | 151869329 | -2331 | 0.85 | 0.43 | 0.42 |
| *S100A14* | NM_020672 | chr1 | 151855674 | 151856212 | -529 | 0.72 | 0.21 | 0.51 |
| *S100A16* | NM_080388 | chr1 | 151852578 | 151853082 | -692 | 0.91 | 0.41 | 0.50 |
| *S100A3* | NM_002960 | chr1 | 151789308 | 151789806 | -1199 | 0.61 | 0.19 | 0.41 |
| *S100A4* | NM_002961 | chr1 | 151787818 | 151788319 | -3162 | 0.77 | 0.43 | 0.34 |
| *S100A7* | NM_002963 | chr1 | 151701455 | 151702014 | -1973 | 0.84 | 0.20 | 0.64 |
| *S100P* | NM_005980 | chr4 | 6744805 | 6745254 | -1436 | 0.54 | 0.21 | 0.32 |
| *S1PR4* | NM_003775 | chr19 | 3127342 | 3127789 | -2199 | 0.71 | 0.39 | 0.32 |
| *SAA1* | NM_000331 | chr11 | 18242980 | 18243422 | -1146 | 0.94 | 0.65 | 0.30 |
| *SAMD3* | NM_152552 | chr6 | 130578145 | 130578636 | -203 | 0.64 | 0.15 | 0.50 |
| *SAMD7* | NM_182610 | chr3 | 171112123 | 171112624 | 198 | 0.65 | 0.10 | 0.55 |
| *SAMD8* | NM_144660 | chr10 | 76538374 | 76538917 | -2826 | 0.79 | 0.38 | 0.41 |
| *SAMSN1* | NM_022136 | chr21 | 14840800 | 14841353 | -541 | 0.66 | 0.32 | 0.34 |
| *SBK2* | NM_001101401 | chr19 | 60740684 | 60741212 | -1475 | 0.88 | 0.54 | 0.33 |
| *SCARF1* | NM_003693 | chr17 | 1496178 | 1496668 | -632 | 0.85 | 0.54 | 0.31 |
| *SCARNA22* | NR_003004 | chr4 | 1944723 | 1945217 | -1190 | 0.68 | 0.36 | 0.32 |
| *SCARNA7* | NR_003001 | chr3 | 161717408 | 161717945 | -1958 | 0.83 | 0.25 | 0.59 |
| *SCHIP1* | NM_014575 | chr3 | 160471804 | 160472347 | -2161 | 0.79 | 0.45 | 0.34 |
| *SCMH1* | NM_001031694 | chr1 | 41400492 | 41400984 | -1105 | 0.75 | 0.37 | 0.38 |
| *SCN4A* | NM_000334 | chr17 | 59404474 | 59404950 | -702 | 0.68 | 0.15 | 0.54 |
| *SCN7A* | NM_002976 | chr2 | 167051691 | 167052188 | -215 | 0.44 | 0.12 | 0.32 |
| *SCNN1A* | NM_001038 | chr12 | 6357014 | 6357521 | -2101 | 0.63 | 0.21 | 0.41 |
| *SCNN1B* | NM_000336 | chr16 | 23220350 | 23220849 | -491 | 0.47 | 0.17 | 0.30 |
| *SCRN2* | NM_001145023 | chr17 | 43275981 | 43276171 | -2378 | 0.81 | 0.31 | 0.51 |
| *SDCBP2* | NM_080489 | chr20 | 1259733 | 1260151 | -2104 | 0.97 | 0.61 | 0.36 |
| *SDK1* | NM_001079653 | chr7 | 4134966 | 4135404 | -657 | 0.73 | 0.20 | 0.53 |
| *SDR42E1* | NM_145168 | chr16 | 80603549 | 80604043 | -1202 | 0.82 | 0.41 | 0.41 |
| *SDSL* | NM_138432 | chr12 | 112343351 | 112343849 | -998 | 0.75 | 0.24 | 0.50 |
| *SELENBP1* | NM_003944 | chr1 | 149613458 | 149613991 | -1936 | 0.74 | 0.32 | 0.43 |
| *SEMA3B* | NM_001005914 | chr3 | 50279074 | 50279576 | -718 | 0.69 | 0.34 | 0.36 |
| *SEMA3D* | NM_152754 | chr7 | 84590582 | 84591094 | -1655 | 0.93 | 0.36 | 0.56 |
| *SEMA3G* | NM_020163 | chr3 | 52455544 | 52456036 | -1707 | 0.67 | 0.18 | 0.48 |
| *SEMA4A* | NM_022367 | chr1 | 154387595 | 154387951 | -2238 | 0.74 | 0.38 | 0.36 |
| *SEMA4G* | NM_017893 | chr10 | 102720604 | 102721138 | -1404 | 0.81 | 0.37 | 0.44 |
| *SEMA6B* | NM_032108 | chr19 | 4510950 | 4511156 | -1550 | 0.78 | 0.37 | 0.41 |
| *SEPN1* | NM_020451 | chr1 | 25997871 | 25998365 | -1135 | 0.80 | 0.32 | 0.48 |
| *SEPT12* | NM_001154458 | chr16 | 4778841 | 4778944 | -492 | 0.74 | 0.44 | 0.30 |
| *SEPT4* | NM_004574 | chr17 | 53961525 | 53962025 | -113 | 0.85 | 0.46 | 0.40 |
| *SEPT9* | NM_001113496 | chr17 | 72956820 | 72957315 | -1139 | 0.70 | 0.19 | 0.51 |
| *SERP2* | NM_001010897 | chr13 | 43845002 | 43845508 | -722 | 0.71 | 0.26 | 0.45 |
| *SERPINA1* | NM_000295 | chr14 | 93926245 | 93926753 | -1593 | 0.75 | 0.30 | 0.45 |
| *SERPINA13* | NR_015340 | chr14 | 94174392 | 94174869 | -2183 | 0.69 | 0.32 | 0.36 |
| *SERPINA3* | NM_001085 | chr14 | 94146851 | 94147050 | -1515 | 0.85 | 0.48 | 0.37 |
| *SERPINA4* | NM_006215 | chr14 | 94096653 | 94097148 | -634 | 0.53 | 0.11 | 0.42 |
| *SERPINF1* | NM_002615 | chr17 | 1611396 | 1611916 | -352 | 0.70 | 0.38 | 0.33 |
| *SERPINF2* | NM_000934 | chr17 | 1592554 | 1593057 | -73 | 0.83 | 0.38 | 0.45 |
| *SEZ6* | NM_001098635 | chr17 | 24357892 | 24358324 | -901 | 0.77 | 0.44 | 0.33 |
| *SFTA2* | NM_205854 | chr6 | 31010146 | 31010370 | -2327 | 0.82 | 0.52 | 0.30 |
| *SFTPB* | NM_000542 | chr2 | 85749260 | 85749764 | -692 | 0.88 | 0.38 | 0.50 |
| *SGCA* | NM_000023 | chr17 | 45598003 | 45598523 | -101 | 0.75 | 0.16 | 0.58 |
| *SGK2* | NM_170693 | chr20 | 41619683 | 41619985 | -1265 | 0.75 | 0.44 | 0.32 |
| *SGPL1* | NM_003901 | chr10 | 72244363 | 72244852 | -1101 | 0.80 | 0.39 | 0.42 |
| *SGPP2* | NM_152386 | chr2 | 222996613 | 222997124 | -696 | 0.76 | 0.34 | 0.42 |
| *SGSH* | NM_000199 | chr17 | 75810829 | 75811215 | -2228 | 0.74 | 0.41 | 0.32 |
| *SH2D2A* | NM_003975 | chr1 | 155053224 | 155053727 | -249 | 0.61 | 0.21 | 0.40 |
| *SH2D3C* | NM_001142531 | chr9 | 129565951 | 129566442 | -1656 | 0.51 | 0.19 | 0.33 |
| *SH2D4A* | NM_022071 | chr8 | 19213596 | 19214110 | -1633 | 0.88 | 0.56 | 0.32 |
| *SH2D6* | NM_198482 | chr2 | 85513997 | 85514487 | -1186 | 0.82 | 0.32 | 0.50 |
| *SH2D7* | NM_001101404 | chr15 | 76171937 | 76172415 | 195 | 0.74 | 0.37 | 0.38 |
| *SH3BGR* | NM_007341 | chr21 | 39744169 | 39744572 | -1278 | 0.87 | 0.48 | 0.40 |
| *SH3BGRL3* | NM_031286 | chr1 | 26477920 | 26478408 | -635 | 0.53 | 0.17 | 0.36 |
| *SH3BP2* | NM_001145855 | chr4 | 2782870 | 2783358 | -629 | 0.86 | 0.31 | 0.55 |
| *SH3D19* | NM_001009555 | chr4 | 152369060 | 152369458 | -2149 | 0.86 | 0.54 | 0.32 |
| *SH3PXD2B* | NM_001017995 | chr5 | 171815085 | 171815575 | -1198 | 0.65 | 0.34 | 0.32 |
| *SH3TC1* | NM_018986 | chr4 | 8250080 | 8250576 | -1631 | 0.80 | 0.37 | 0.43 |
| *SHISA5* | NM_016479 | chr3 | 48517455 | 48517967 | -1046 | 0.76 | 0.37 | 0.39 |
| *SHMT1* | NM_004169 | chr17 | 18207940 | 18208270 | -524 | 0.70 | 0.33 | 0.37 |
| *SHROOM1* | NM_133456 | chr5 | 132190390 | 132190871 | -729 | 0.74 | 0.26 | 0.48 |
| *SIGLEC1* | NM_023068 | chr20 | 3635728 | 3636238 | -208 | 0.70 | 0.31 | 0.40 |
| *SIGLEC10* | NM_033130 | chr19 | 56613106 | 56613652 | -116 | 0.72 | 0.36 | 0.36 |
| *SIGLEC9* | NM_014441 | chr19 | 56319190 | 56319706 | -528 | 0.52 | 0.15 | 0.37 |
| *SIGMAR1* | NM_005866 | chr9 | 34628228 | 34628735 | -713 | 0.64 | 0.25 | 0.39 |
| *SIPA1* | NM_006747 | chr11 | 65160749 | 65161233 | -1162 | 0.65 | 0.33 | 0.32 |
| *SLAMF8* | NM_020125 | chr1 | 158061240 | 158061719 | -1622 | 0.77 | 0.40 | 0.36 |
| *SLC10A1* | NM_003049 | chr14 | 69334712 | 69335220 | -1207 | 0.82 | 0.23 | 0.59 |
| *SLC12A5* | NM_001134771 | chr20 | 44081319 | 44081557 | -2297 | 0.70 | 0.23 | 0.47 |
| *SLC16A5* | NM_004695 | chr17 | 70594757 | 70595264 | -638 | 0.55 | 0.24 | 0.31 |
| *SLC17A8* | NM_001145288 | chr12 | 99274677 | 99275177 | -60 | 0.73 | 0.33 | 0.39 |
| *SLC1A7* | NM_006671 | chr1 | 53381883 | 53382367 | -1248 | 0.62 | 0.28 | 0.34 |
| *SLC22A1* | NM_003057 | chr6 | 160461479 | 160461988 | -1118 | 0.90 | 0.50 | 0.40 |
| *SLC22A10* | NM_001039752 | chr11 | 62812919 | 62813407 | -842 | 0.79 | 0.30 | 0.49 |
| *SLC22A12* | NM_144585 | chr11 | 64114392 | 64114892 | -215 | 0.67 | 0.17 | 0.50 |
| *SLC22A13* | NM_004256 | chr3 | 38281827 | 38282270 | -252 | 0.55 | 0.14 | 0.41 |
| *SLC22A18* | NM_183233 | chr11 | 2875640 | 2876150 | -1631 | 0.68 | 0.36 | 0.32 |
| *SLC22A18AS* | NM_007105 | chr11 | 2883195 | 2883705 | -1699 | 0.76 | 0.28 | 0.48 |
| *SLC22A20* | NM_001004326 | chr11 | 64737502 | 64737994 | -136 | 0.62 | 0.24 | 0.38 |
| *SLC22A23* | NM_021945 | chr6 | 3390158 | 3390368 | -46 | 0.74 | 0.39 | 0.35 |
| *SLC22A7* | NM_006672 | chr6 | 43373059 | 43373558 | -666 | 0.71 | 0.39 | 0.33 |
| *SLC22A8* | NM_004254 | chr11 | 62539738 | 62540250 | -107 | 0.67 | 0.22 | 0.45 |
| *SLC23A3* | NM_001144889 | chr2 | 219743010 | 219743406 | -147 | 0.74 | 0.36 | 0.38 |
| *SLC25A25* | NM_001006643 | chr9 | 129899822 | 129900346 | -572 | 0.66 | 0.36 | 0.30 |
| *SLC25A34* | NM_207348 | chr1 | 15934418 | 15934904 | -734 | 0.63 | 0.27 | 0.36 |
| *SLC25A4* | NM_001151 | chr4 | 186298975 | 186299522 | -2142 | 0.88 | 0.43 | 0.44 |
| *SLC25A45* | NM_001077241 | chr11 | 64908858 | 64909382 | -2402 | 0.78 | 0.45 | 0.33 |
| *SLC26A6* | NM_001040454 | chr3 | 48646227 | 48646723 | -192 | 0.82 | 0.47 | 0.35 |
| *SLC27A5* | NM_012254 | chr19 | 63715699 | 63716207 | -709 | 0.67 | 0.28 | 0.40 |
| *SLC28A1* | NM_004213 | chr15 | 83228472 | 83228952 | -204 | 0.82 | 0.34 | 0.48 |
| *SLC28A3* | NM_022127 | chr9 | 86173139 | 86173648 | -160 | 0.74 | 0.36 | 0.38 |
| *SLC29A1* | NM_001078174 | chr6 | 44294253 | 44294429 | -878 | 0.75 | 0.36 | 0.39 |
| *SLC29A3* | NM_018344 | chr10 | 72746592 | 72747125 | -2156 | 0.78 | 0.36 | 0.43 |
| *SLC2A2* | NM_000340 | chr3 | 172227402 | 172227921 | -199 | 0.58 | 0.16 | 0.42 |
| *SLC2A7* | NM_207420 | chr1 | 9010550 | 9010992 | -1780 | 0.66 | 0.33 | 0.32 |
| *SLC2A9* | NM_001001290 | chr4 | 9652366 | 9652662 | -1544 | 0.97 | 0.20 | 0.77 |
| *SLC34A3* | NM_080877 | chr9 | 139243715 | 139244230 | -1232 | 0.62 | 0.31 | 0.31 |
| *SLC35A2* | NM_001032289 | chrX | 48656326 | 48656650 | -2611 | 0.66 | 0.30 | 0.36 |
| *SLC38A11* | NM_173512 | chr2 | 165520146 | 165520665 | -124 | 0.49 | 0.17 | 0.33 |
| *SLC38A5* | NM_033518 | chrX | 48215728 | 48216041 | -2296 | 0.63 | 0.32 | 0.31 |
| *SLC39A4* | NM_017767 | chr8 | 145614182 | 145614664 | -1698 | 0.62 | 0.23 | 0.39 |
| *SLC44A4* | NM_025257 | chr6 | 31957078 | 31957284 | -2379 | 0.86 | 0.33 | 0.53 |
| *SLC45A3* | NM_033102 | chr1 | 203917568 | 203917856 | -1459 | 0.72 | 0.36 | 0.37 |
| *SLC45A4* | NM_001080431 | chr8 | 142308309 | 142308819 | -709 | 0.47 | 0.14 | 0.33 |
| *SLC47A1* | NM_018242 | chr17 | 19376291 | 19376485 | -1370 | 0.85 | 0.46 | 0.40 |
| *SLC47A2* | NM_001099646 | chr17 | 19560495 | 19560975 | -100 | 0.87 | 0.56 | 0.31 |
| *SLC4A1* | NM_000342 | chr17 | 39701378 | 39701892 | -607 | 0.89 | 0.52 | 0.37 |
| *SLC4A9* | NM_031467 | chr5 | 139719371 | 139719855 | -357 | 0.68 | 0.36 | 0.32 |
| *SLC5A1* | NM_000343 | chr22 | 30768094 | 30768570 | -704 | 0.93 | 0.31 | 0.63 |
| *SLC5A11* | NM_052944 | chr16 | 24763180 | 24763682 | -1621 | 0.83 | 0.23 | 0.59 |
| *SLC6A1* | NM_003042 | chr3 | 11007949 | 11008447 | -1221 | 0.57 | 0.26 | 0.30 |
| *SLC6A12* | NM_001122847 | chr12 | 194641 | 195127 | -1789 | 0.79 | 0.43 | 0.36 |
| *SLC6A14* | NM_007231 | chrX | 115480928 | 115481435 | -619 | 0.73 | 0.21 | 0.52 |
| *SLC6A7* | NM_014228 | chr5 | 149548299 | 149548810 | -1157 | 0.88 | 0.36 | 0.53 |
| *SLC7A7* | NM_001126106 | chr14 | 22359967 | 22360468 | -1363 | 0.69 | 0.22 | 0.47 |
| *SLC9A10* | NM_183061 | chr3 | 113496161 | 113496631 | -632 | 0.66 | 0.12 | 0.54 |
| *SLCO1A2* | NM_021094 | chr12 | 21380949 | 21381450 | -2100 | 0.66 | 0.30 | 0.36 |
| *SLCO1B1* | NM_006446 | chr12 | 21172959 | 21173413 | -2208 | 0.68 | 0.27 | 0.41 |
| *SLCO1B3* | NM_019844 | chr12 | 20854527 | 20855015 | -133 | 0.91 | 0.28 | 0.63 |
| *SLCO2B1* | NM_001145211 | chr11 | 74546068 | 74546594 | -2161 | 0.88 | 0.42 | 0.46 |
| *SLFNL1* | NM_144990 | chr1 | 41259913 | 41260421 | -193 | 0.67 | 0.28 | 0.38 |
| *SLITRK2* | NM_001144006 | chrX | 144709945 | 144710402 | -529 | 0.78 | 0.16 | 0.62 |
| *SLURP1* | NM_020427 | chr8 | 143820788 | 143821292 | -209 | 0.68 | 0.39 | 0.30 |
| *SMAD5OS* | NR_026763 | chr5 | 135500044 | 135500325 | -1706 | 0.93 | 0.49 | 0.44 |
| *SMAD6* | NM_001142861 | chr15 | 64786641 | 64787138 | -1117 | 0.79 | 0.27 | 0.52 |
| *SMARCB1* | NM_001007468 | chr22 | 22456720 | 22456862 | -2358 | 0.67 | 0.31 | 0.36 |
| *SMARCD1* | NM_003076 | chr12 | 48762824 | 48763359 | -2157 | 0.90 | 0.45 | 0.45 |
| *SMARCD3* | NM_001003801 | chr7 | 150577584 | 150578091 | -1155 | 0.70 | 0.32 | 0.38 |
| *SMCR7L* | NM_019008 | chr22 | 38227038 | 38227538 | -941 | 0.80 | 0.40 | 0.40 |
| *SMEK3P* | NR_002784 | chrX | 27392602 | 27393103 | -1473 | 0.87 | 0.57 | 0.30 |
| *SMOC1* | NM_001034852 | chr14 | 69414420 | 69414928 | -1221 | 0.83 | 0.39 | 0.44 |
| *SNAI1* | NM_005985 | chr20 | 48031449 | 48031957 | -1230 | 0.75 | 0.36 | 0.39 |
| *SNCG* | NM_003087 | chr10 | 88705828 | 88706301 | -2202 | 0.84 | 0.16 | 0.68 |
| *SNIP1* | NM_024700 | chr1 | 37792451 | 37792983 | -227 | 0.62 | 0.31 | 0.31 |
| *SNORA11B* | NR_003709 | chr14 | 90660312 | 90660767 | -1982 | 0.46 | 0.11 | 0.35 |
| *SNORA12* | NR_002954 | chr10 | 101986991 | 101987295 | -94 | 0.90 | 0.41 | 0.48 |
| *SNORA19* | NR_002917 | chr10 | 120810589 | 120811094 | -1201 | 0.87 | 0.22 | 0.65 |
| *SNORA46* | NR_002978 | chr16 | 57141869 | 57142402 | -2097 | 0.90 | 0.55 | 0.35 |
| *SNORA5B* | NR_002990 | chr7 | 45113968 | 45114445 | -1983 | 0.71 | 0.27 | 0.44 |
| *SNORA5C* | NR_002991 | chr7 | 45113968 | 45114445 | -3040 | 0.71 | 0.27 | 0.44 |
| *SNORA60* | NR_002986 | chr20 | 36510469 | 36510967 | -707 | 0.82 | 0.43 | 0.39 |
| *SNORA69* | NR_002584 | chrX | 118807213 | 118807726 | -1994 | 0.83 | 0.40 | 0.43 |
| *SNORA79* | NR_003021 | chr14 | 80739366 | 80739854 | -679 | 0.56 | 0.17 | 0.39 |
| *SNORD113-5* | NR_003233 | chr14 | 100473903 | 100474404 | -122 | 0.56 | 0.21 | 0.36 |
| *SNORD113-6* | NR_003234 | chr14 | 100473903 | 100474404 | -1491 | 0.56 | 0.21 | 0.36 |
| *SNORD113-7* | NR_003235 | chr14 | 100473903 | 100474404 | -3061 | 0.56 | 0.21 | 0.36 |
| *SNORD115-22* | NR_003314 | chr15 | 23005116 | 23005638 | -780 | 0.66 | 0.22 | 0.44 |
| *SNORD115-23* | NR_003315 | chr15 | 23005116 | 23005638 | -2658 | 0.66 | 0.22 | 0.44 |
| *SNORD115-25* | NR_003342 | chr15 | 23009074 | 23009566 | -2460 | 0.72 | 0.21 | 0.51 |
| *SNORD116-26* | NR_003340 | chr15 | 22894675 | 22895196 | -801 | 0.85 | 0.42 | 0.43 |
| *SNORD116-27* | NR_003341 | chr15 | 22896598 | 22897091 | -968 | 0.75 | 0.27 | 0.49 |
| *SNORD116-29* | NR_003360 | chr15 | 22901311 | 22901797 | -1205 | 0.94 | 0.60 | 0.35 |
| *SNORD117* | NR_003140 | chr6 | 31614419 | 31614643 | -2326 | 0.88 | 0.32 | 0.56 |
| *SNORD119* | NR_003684 | chr20 | 2392557 | 2393043 | -1107 | 0.85 | 0.40 | 0.46 |
| *SNORD124* | NR_003692 | chr17 | 35437363 | 35437870 | -192 | 0.68 | 0.37 | 0.31 |
| *SNORD125* | NR_003686 | chr22 | 28061219 | 28061711 | -2218 | 0.67 | 0.17 | 0.50 |
| *SNORD126* | NR_003693 | chr14 | 19864381 | 19864904 | -104 | 0.69 | 0.33 | 0.35 |
| *SNORD15B* | NR_000025 | chr11 | 74790696 | 74791254 | -2137 | 0.82 | 0.39 | 0.43 |
| *SNORD62A* | NR_002914 | chr9 | 133349000 | 133349506 | -1619 | 0.68 | 0.20 | 0.48 |
| *SNORD62B* | NR_003050 | chr9 | 133349000 | 133349506 | -1619 | 0.68 | 0.20 | 0.48 |
| *SNORD67* | NR_003056 | chr11 | 46742446 | 46742964 | -2080 | 0.76 | 0.46 | 0.31 |
| *SNORD71* | NR_003059 | chr16 | 70349841 | 70350340 | -199 | 0.78 | 0.42 | 0.36 |
| *SNORD85* | NR_003066 | chr1 | 31214935 | 31215445 | -1519 | 0.87 | 0.47 | 0.40 |
| *SNORD94* | NR_004378 | chr2 | 86215612 | 86216112 | -641 | 0.79 | 0.42 | 0.37 |
| *SNORD96B* | NR_004379 | chrX | 109355314 | 109355797 | -611 | 0.82 | 0.44 | 0.37 |
| *SNUPN* | NM_001042581 | chr15 | 73706973 | 73707443 | -1707 | 0.88 | 0.52 | 0.37 |
| *SNX22* | NM_024798 | chr15 | 62228752 | 62229203 | -1990 | 0.88 | 0.57 | 0.31 |
| *SNX32* | NM_152760 | chr11 | 65356611 | 65357101 | -1129 | 0.87 | 0.56 | 0.31 |
| *SNX7* | NM_015976 | chr1 | 98897716 | 98898163 | -1883 | 0.73 | 0.33 | 0.40 |
| *SOAT1* | NM_003101 | chr1 | 177528173 | 177528568 | -1268 | 0.78 | 0.48 | 0.30 |
| *SORBS2* | NM_021069 | chr4 | 187116204 | 187116707 | -1591 | 0.60 | 0.25 | 0.35 |
| *SOST* | NM_025237 | chr17 | 39192652 | 39193131 | -1209 | 0.59 | 0.18 | 0.41 |
| *SOX10* | NM_006941 | chr22 | 36711137 | 36711613 | -890 | 0.72 | 0.36 | 0.36 |
| *SOX15* | NM_006942 | chr17 | 7435473 | 7435974 | -1511 | 0.78 | 0.32 | 0.46 |
| *SOX5* | NM_006940 | chr12 | 23995352 | 23995851 | -1697 | 0.90 | 0.32 | 0.57 |
| *SP100* | NM_001080391 | chr2 | 230986699 | 230987228 | -2150 | 0.72 | 0.40 | 0.32 |
| *SP110* | NM_004509 | chr2 | 230793975 | 230794489 | -1161 | 0.84 | 0.44 | 0.40 |
| *SP140* | NM_001005176 | chr2 | 230797245 | 230797755 | -1188 | 0.88 | 0.53 | 0.35 |
| *SP140L* | NM_138402 | chr2 | 230897719 | 230898254 | -2150 | 0.83 | 0.52 | 0.31 |
| *SPAG1* | NM_003114 | chr8 | 101237473 | 101237978 | -2105 | 0.81 | 0.51 | 0.30 |
| *SPAG4L* | NM_080675 | chr20 | 31058816 | 31059322 | -3169 | 0.87 | 0.44 | 0.43 |
| *SPARCL1* | NM_001128310 | chr4 | 88671545 | 88672048 | -2117 | 0.73 | 0.40 | 0.34 |
| *SPATA12* | NM_181727 | chr3 | 57069287 | 57069718 | -5 | 0.79 | 0.47 | 0.33 |
| *SPATA13* | NM_153023 | chr13 | 23630919 | 23631423 | -1715 | 0.87 | 0.52 | 0.34 |
| *SPATA16* | NM_031955 | chr3 | 174343622 | 174344173 | -2171 | 0.81 | 0.51 | 0.30 |
| *SPATA18* | NM_145263 | chr4 | 52611091 | 52611619 | -994 | 0.78 | 0.44 | 0.35 |
| *SPATA3* | NM_139073 | chr2 | 231567719 | 231568187 | -1129 | 0.73 | 0.38 | 0.34 |
| *SPATA4* | NM_144644 | chr4 | 177354779 | 177355296 | -1221 | 0.84 | 0.39 | 0.45 |
| *SPATA8* | NM_173499 | chr15 | 95127316 | 95127800 | -124 | 0.77 | 0.30 | 0.47 |
| *SPATC1* | NM_001134374 | chr8 | 145156132 | 145156596 | -2205 | 0.47 | 0.18 | 0.30 |
| *SPDYA* | NM_001008779 | chr2 | 28890680 | 28891151 | -1450 | 0.84 | 0.51 | 0.33 |
| *SPEF1* | NM_015417 | chr20 | 3711575 | 3712085 | -1728 | 0.67 | 0.30 | 0.37 |
| *SPEM1* | NM_199339 | chr17 | 7264013 | 7264513 | -139 | 0.50 | 0.15 | 0.35 |
| *SPERT* | NM_152719 | chr13 | 45172007 | 45172514 | -2185 | 0.82 | 0.49 | 0.34 |
| *SPI1* | NM_001080547 | chr11 | 47356546 | 47357060 | -100 | 0.65 | 0.19 | 0.45 |
| *SPIN2B* | NM_001006681 | chrX | 57165650 | 57166094 | -1168 | 0.90 | 0.53 | 0.37 |
| *SPIN3* | NM_001010862 | chrX | 57039675 | 57040179 | -1214 | 0.83 | 0.47 | 0.35 |
| *SPIN4* | NM_001012968 | chrX | 62488386 | 62488903 | -701 | 0.80 | 0.38 | 0.42 |
| *SPINK9* | NM_001040433 | chr5 | 147693956 | 147694143 | -1264 | 0.82 | 0.49 | 0.33 |
| *SPINT4* | NM_178455 | chr20 | 43782733 | 43783115 | -1477 | 0.84 | 0.51 | 0.32 |
| *SPOPL* | NM_001001664 | chr2 | 138974615 | 138975099 | -962 | 0.59 | 0.24 | 0.35 |
| *SPRY3* | NM_005840 | chrX | 154650178 | 154650678 | -216 | 0.73 | 0.43 | 0.30 |
| *SPTBN5* | NM_016642 | chr15 | 39975008 | 39975411 | -1642 | 0.81 | 0.27 | 0.54 |
| *SREBF1* | NM_001005291 | chr17 | 17681736 | 17682248 | -942 | 0.71 | 0.32 | 0.39 |
| *SRI* | NM_198901 | chr7 | 87694090 | 87694588 | -95 | 0.84 | 0.35 | 0.49 |
| *SRPK1* | NM_003137 | chr6 | 35998448 | 35998934 | -1757 | 0.84 | 0.52 | 0.32 |
| *SRPK3* | NM_014370 | chrX | 152698429 | 152698937 | -1020 | 0.67 | 0.31 | 0.36 |
| *SRY* | NM_003140 | chrY | 2716740 | 2717253 | -1204 | 0.93 | 0.43 | 0.50 |
| *SSR1* | NM_003144 | chr6 | 7259239 | 7259718 | -938 | 0.83 | 0.44 | 0.39 |
| *ST6GALNAC4* | NM_175039 | chr9 | 129720510 | 129721008 | -1633 | 0.90 | 0.40 | 0.50 |
| *STAB1* | NM_015136 | chr3 | 52502506 | 52503028 | -1628 | 0.75 | 0.40 | 0.35 |
| *STARD3* | NM_006804 | chr17 | 35046151 | 35046671 | -526 | 0.61 | 0.29 | 0.32 |
| *STARD8* | NM_001142504 | chrX | 67828338 | 67828826 | -1635 | 0.89 | 0.57 | 0.32 |
| *STAT1* | NM_007315 | chr2 | 191588185 | 191588662 | -1202 | 0.86 | 0.56 | 0.30 |
| *STAT5A* | NM_003152 | chr17 | 37691947 | 37692453 | -890 | 0.81 | 0.31 | 0.51 |
| *STH* | NM_001007532 | chr17 | 41432119 | 41432618 | -83 | 0.64 | 0.27 | 0.37 |
| *STK24* | NM_003576 | chr13 | 97972281 | 97972789 | -193 | 0.78 | 0.13 | 0.66 |
| *STOML3* | NM_001144033 | chr13 | 38465111 | 38465465 | -2292 | 0.99 | 0.67 | 0.32 |
| *STON1-GTF2A1L* | NM_172311 | chr2 | 48648996 | 48649490 | -419 | 0.82 | 0.48 | 0.34 |
| *STRAP* | NM_007178 | chr12 | 15924639 | 15925140 | -1664 | 0.80 | 0.43 | 0.37 |
| *STX1A* | NM_004603 | chr7 | 72773422 | 72773969 | -1771 | 0.83 | 0.53 | 0.30 |
| *STX1B* | NM_052874 | chr16 | 30930171 | 30930575 | -1043 | 0.84 | 0.54 | 0.30 |
| *SUCNR1* | NM_033050 | chr3 | 153073846 | 153074337 | -34 | 0.86 | 0.55 | 0.31 |
| *SUGT1L1* | NR_003365 | chr13 | 40395208 | 40395716 | -1576 | 0.59 | 0.29 | 0.31 |
| *SUGT1P* | NR_003667 | chr9 | 33501909 | 33502091 | -953 | 0.74 | 0.41 | 0.33 |
| *SULT1A2* | NM_177528 | chr16 | 28515143 | 28515479 | -9 | 0.72 | 0.36 | 0.36 |
| *SULT1A3* | NM_177552 | chr16 | 29376234 | 29376722 | -2216 | 0.66 | 0.23 | 0.44 |
| *SULT1A4* | NM_001017390 | chr16 | 29376234 | 29376722 | -2216 | 0.66 | 0.23 | 0.44 |
| *SULT1C4* | NM_006588 | chr2 | 108360602 | 108361086 | -8 | 0.66 | 0.25 | 0.41 |
| *SUMO1P3* | NR_002190 | chr1 | 158551711 | 158552191 | -1727 | 0.60 | 0.30 | 0.30 |
| *SUMO4* | NM_001002255 | chr6 | 149760757 | 149761222 | -2197 | 0.94 | 0.13 | 0.81 |
| *SURF6* | NM_006753 | chr9 | 135194521 | 135194807 | -1796 | 0.73 | 0.41 | 0.32 |
| *SUSD2* | NM_019601 | chr22 | 22906029 | 22906531 | -1163 | 0.70 | 0.37 | 0.33 |
| *SUSD3* | NM_145006 | chr9 | 94858387 | 94858520 | -2355 | 0.96 | 0.50 | 0.46 |
| *SUV39H1* | NM_003173 | chrX | 48438183 | 48438701 | -1632 | 0.81 | 0.51 | 0.30 |
| *SYN3* | NM_003490 | chr22 | 31733645 | 31733886 | -959 | 0.44 | 0.12 | 0.32 |
| *SYNE1* | NM_015293 | chr6 | 152683149 | 152683631 | -2218 | 0.87 | 0.55 | 0.32 |
| *SYNGR1* | NM_145738 | chr22 | 38089740 | 38090230 | -135 | 0.80 | 0.28 | 0.52 |
| *SYNPO* | NM_001109974 | chr5 | 149997972 | 149998435 | -2208 | 0.88 | 0.28 | 0.59 |
| *SYTL1* | NM_032872 | chr1 | 27540184 | 27540733 | -640 | 0.59 | 0.17 | 0.42 |
| *TAAR1* | NM_138327 | chr6 | 133009280 | 133009800 | -705 | 0.94 | 0.64 | 0.30 |
| *TACC1* | NM_001146216 | chr8 | 38703362 | 38703852 | -1257 | 0.62 | 0.25 | 0.37 |
| *TAF2* | NM_003184 | chr8 | 120915231 | 120915698 | -1209 | 0.71 | 0.38 | 0.33 |
| *TAGLN* | NM_001001522 | chr11 | 116574859 | 116575369 | -135 | 0.49 | 0.17 | 0.33 |
| *TANK* | NM_004180 | chr2 | 161699292 | 161699769 | -2180 | 0.85 | 0.54 | 0.30 |
| *TAOK2* | NM_004783 | chr16 | 29891358 | 29891833 | -1126 | 0.90 | 0.42 | 0.48 |
| *TAPBPL* | NM_018009 | chr12 | 6431350 | 6431868 | 172 | 0.54 | 0.21 | 0.33 |
| *TAS1R2* | NM_152232 | chr1 | 19058600 | 19059114 | -115 | 0.88 | 0.32 | 0.56 |
| *TAS2R50* | NM_176890 | chr12 | 11031176 | 11031672 | -646 | 0.93 | 0.46 | 0.47 |
| *TASP1* | NM_017714 | chr20 | 13568226 | 13568683 | -871 | 0.78 | 0.46 | 0.32 |
| *TBC1D10A* | NM_031937 | chr22 | 29053696 | 29054235 | -1071 | 0.68 | 0.39 | 0.30 |
| *TBC1D10C* | NM_198517 | chr11 | 66927744 | 66928232 | 1 | 0.68 | 0.38 | 0.30 |
| *TBC1D26* | NM_178571 | chr17 | 15575939 | 15576433 | -129 | 0.72 | 0.41 | 0.31 |
| *TBC1D28* | NM_001039397 | chr17 | 18488320 | 18488804 | -97 | 0.79 | 0.43 | 0.36 |
| *TBC1D29* | NM_015594 | chr17 | 25910280 | 25910818 | -160 | 0.56 | 0.15 | 0.41 |
| *TBCE* | NM_001079515 | chr1 | 233596346 | 233596856 | -749 | 0.76 | 0.44 | 0.32 |
| *TBX15* | NM_152380 | chr1 | 119335496 | 119336004 | -2048 | 0.89 | 0.57 | 0.32 |
| *TBXA2R* | NM_001060 | chr19 | 3560022 | 3560116 | -2411 | 0.69 | 0.31 | 0.39 |
| *TC2N* | NM_001128596 | chr14 | 91404096 | 91404572 | -701 | 0.79 | 0.49 | 0.30 |
| *TCEA2* | NM_198723 | chr20 | 62157037 | 62157586 | -1570 | 0.71 | 0.20 | 0.52 |
| *TCEAL1* | NM_001006639 | chrX | 102769420 | 102769888 | -893 | 0.95 | 0.52 | 0.43 |
| *TCEAL2* | NM_080390 | chrX | 101265471 | 101265955 | -1602 | 0.79 | 0.47 | 0.32 |
| *TCF20* | NM_005650 | chr22 | 40942752 | 40943242 | -1608 | 0.76 | 0.25 | 0.51 |
| *TCIRG1* | NM_006053 | chr11 | 67565532 | 67566056 | -1228 | 0.71 | 0.25 | 0.46 |
| *TCL1B* | NM_004918 | chr14 | 95221149 | 95221627 | -1127 | 0.58 | 0.24 | 0.33 |
| *TCL6* | NM_020553 | chr14 | 95185792 | 95186320 | -1211 | 0.79 | 0.35 | 0.44 |
| *TCN2* | NM_000355 | chr22 | 29332575 | 29333071 | -337 | 0.61 | 0.29 | 0.31 |
| *TCTEX1D1* | NM_152665 | chr1 | 66989774 | 66990291 | -696 | 0.85 | 0.22 | 0.63 |
| *TCTEX1D4* | NM_001013632 | chr1 | 45046287 | 45046801 | -1000 | 0.56 | 0.18 | 0.38 |
| *TEKT2* | NM_014466 | chr1 | 36320652 | 36321077 | -1397 | 0.90 | 0.52 | 0.39 |
| *TEPP* | NM_199046 | chr16 | 56567419 | 56567909 | -175 | 0.79 | 0.37 | 0.42 |
| *TERC* | NR_001566 | chr3 | 170967663 | 170967965 | -2272 | 0.70 | 0.37 | 0.32 |
| *TET3* | NM_144993 | chr2 | 74125921 | 74126392 | -800 | 0.80 | 0.44 | 0.37 |
| *TEX264* | NM_001129884 | chr3 | 51677822 | 51678371 | -2164 | 0.83 | 0.23 | 0.59 |
| *TFDP3* | NM_016521 | chrX | 132180930 | 132181479 | -1162 | 0.93 | 0.55 | 0.39 |
| *TFR2* | NM_003227 | chr7 | 100078563 | 100079072 | -1708 | 0.68 | 0.37 | 0.31 |
| *TGFBI* | NM_000358 | chr5 | 135391022 | 135391538 | -1202 | 0.85 | 0.39 | 0.47 |
| *TGM1* | NM_000359 | chr14 | 23802196 | 23802702 | -193 | 0.81 | 0.42 | 0.39 |
| *TGM2* | NM_004613 | chr20 | 36227476 | 36227954 | -601 | 0.77 | 0.36 | 0.42 |
| *THBS2* | NM_003247 | chr6 | 169396516 | 169397002 | -697 | 0.63 | 0.32 | 0.31 |
| *THY1* | NM_006288 | chr11 | 118800644 | 118801150 | -1441 | 0.83 | 0.44 | 0.39 |
| *TIAF1* | NM_004740 | chr17 | 24428717 | 24429127 | -2169 | 0.74 | 0.29 | 0.45 |
| *TIAM2* | NM_012454 | chr6 | 155451355 | 155451835 | -1519 | 0.94 | 0.56 | 0.38 |
| *TICAM1* | NM_182919 | chr19 | 4784202 | 4784380 | -1554 | 0.96 | 0.53 | 0.43 |
| *TIE1* | NM_005424 | chr1 | 43538418 | 43538916 | -583 | 0.77 | 0.37 | 0.40 |
| *TIFAB* | NM_001099221 | chr5 | 134815836 | 134816327 | -93 | 0.85 | 0.42 | 0.44 |
| *TIGIT* | NM_173799 | chr3 | 115495202 | 115495744 | -91 | 0.78 | 0.32 | 0.45 |
| *TINAGL1* | NM_022164 | chr1 | 31813689 | 31814181 | -737 | 0.74 | 0.41 | 0.34 |
| *TJAP1* | NM_001146017 | chr6 | 43562880 | 43563331 | -2193 | 0.75 | 0.17 | 0.57 |
| *TJP3* | NM_014428 | chr19 | 3676933 | 3677303 | -2255 | 0.57 | 0.21 | 0.35 |
| *TLK1* | NM_001136554 | chr2 | 171795973 | 171796490 | -161 | 0.85 | 0.50 | 0.34 |
| *TLR5* | NM_003268 | chr1 | 221383696 | 221384186 | -694 | 0.69 | 0.24 | 0.45 |
| *TLR9* | NM_017442 | chr3 | 52236662 | 52237168 | -1696 | 0.62 | 0.24 | 0.39 |
| *TM4SF20* | NM_024795 | chr2 | 227953454 | 227953936 | -1429 | 0.81 | 0.34 | 0.46 |
| *TM6SF2* | NM_001001524 | chr19 | 19246035 | 19246537 | -1212 | 0.83 | 0.45 | 0.38 |
| *TM7SF3* | NM_016551 | chr12 | 27060475 | 27060907 | -2085 | 0.82 | 0.47 | 0.35 |
| *TMBIM1* | NM_022152 | chr2 | 218867463 | 218867963 | -2189 | 0.79 | 0.47 | 0.31 |
| *TMC3* | NM_001080532 | chr15 | 79455314 | 79455837 | -2102 | 0.88 | 0.56 | 0.33 |
| *TMC4* | NM_001145303 | chr19 | 59370406 | 59370919 | -1906 | 0.71 | 0.38 | 0.33 |
| *TMEM120A* | NM_031925 | chr7 | 75463278 | 75463769 | -1595 | 0.72 | 0.39 | 0.32 |
| *TMEM120B* | NM_001080825 | chr12 | 120633233 | 120633325 | -1761 | 0.63 | 0.30 | 0.32 |
| *TMEM125* | NM_144626 | chr1 | 43507977 | 43508497 | -756 | 0.86 | 0.31 | 0.55 |
| *TMEM126A* | NM_032273 | chr11 | 85035570 | 85036025 | -894 | 0.76 | 0.44 | 0.32 |
| *TMEM132A* | NM_017870 | chr11 | 60446522 | 60447011 | -1721 | 0.90 | 0.60 | 0.30 |
| *TMEM135* | NM_022918 | chr11 | 86424292 | 86424731 | -2200 | 0.81 | 0.42 | 0.38 |
| *TMEM145* | NM_173633 | chr19 | 47508650 | 47509138 | -422 | 0.69 | 0.35 | 0.34 |
| *TMEM177* | NM_001105198 | chr2 | 120150797 | 120151254 | -2186 | 0.80 | 0.36 | 0.44 |
| *TMEM185B* | NR_000034 | chr2 | 120698352 | 120698870 | -1157 | 0.70 | 0.40 | 0.30 |
| *TMEM188* | NM_153261 | chr16 | 48614272 | 48614503 | -2301 | 0.47 | 0.07 | 0.41 |
| *TMEM19* | NM_018279 | chr12 | 70365238 | 70365528 | -761 | 0.58 | 0.27 | 0.30 |
| *TMEM195* | NM_001004320 | chr7 | 15569131 | 15569639 | -1220 | 0.80 | 0.44 | 0.36 |
| *TMEM200A* | NM_052913 | chr6 | 130798418 | 130798888 | -1301 | 0.70 | 0.38 | 0.32 |
| *TMEM208* | NM_014187 | chr16 | 65817631 | 65818141 | -630 | 0.69 | 0.33 | 0.36 |
| *TMEM26* | NM_178505 | chr10 | 62883896 | 62884399 | -933 | 0.77 | 0.37 | 0.40 |
| *TMEM39A* | NM_018266 | chr3 | 120666010 | 120666534 | -1111 | 0.76 | 0.46 | 0.30 |
| *TMEM39B* | NM_018056 | chr1 | 32308743 | 32309084 | -2175 | 0.80 | 0.41 | 0.39 |
| *TMEM40* | NM_018306 | chr3 | 12778174 | 12778271 | -2414 | 0.78 | 0.29 | 0.49 |
| *TMEM42* | NM_144638 | chr3 | 44876170 | 44876679 | -1986 | 0.87 | 0.52 | 0.35 |
| *TMEM72* | NM_001123376 | chr10 | 44726756 | 44727278 | 248 | 0.71 | 0.36 | 0.35 |
| *TMEM86B* | NM_173804 | chr19 | 60433408 | 60433900 | -1210 | 0.51 | 0.19 | 0.32 |
| *TMEM89* | NM_001008269 | chr3 | 48634041 | 48634446 | -50 | 0.73 | 0.37 | 0.36 |
| *TMEM95* | NM_198154 | chr17 | 7197529 | 7198047 | -1432 | 0.70 | 0.30 | 0.40 |
| *TMPRSS11B* | NM_182502 | chr4 | 68795352 | 68795903 | -1620 | 0.99 | 0.38 | 0.61 |
| *TMPRSS11D* | NM_004262 | chr4 | 68433160 | 68433604 | -1071 | 0.80 | 0.30 | 0.50 |
| *TMPRSS4* | NM_001083947 | chr11 | 117450687 | 117451220 | -1982 | 0.61 | 0.18 | 0.43 |
| *TNFAIP8L2* | NM_024575 | chr1 | 149395390 | 149395887 | -89 | 0.65 | 0.09 | 0.56 |
| *TNFRSF10C* | NM_003841 | chr8 | 23015099 | 23015587 | -1035 | 0.81 | 0.40 | 0.42 |
| *TNFRSF10D* | NM_003840 | chr8 | 23079165 | 23079649 | -1922 | 0.72 | 0.38 | 0.34 |
| *TNFRSF19* | NM_148957 | chr13 | 23040839 | 23041374 | -1615 | 0.46 | 0.15 | 0.31 |
| *TNFRSF1A* | NM_001065 | chr12 | 6323004 | 6323488 | -1724 | 0.72 | 0.23 | 0.49 |
| *TNFRSF1B* | NM_001066 | chr1 | 12147242 | 12147681 | -2184 | 0.85 | 0.46 | 0.39 |
| *TNFRSF25* | NM_001039664 | chr1 | 6450584 | 6451088 | -1994 | 0.64 | 0.33 | 0.31 |
| *TNFRSF4* | NM_003327 | chr1 | 1140259 | 1140739 | -1124 | 0.73 | 0.34 | 0.39 |
| *TNFRSF6B* | NM_003823 | chr20 | 61795690 | 61796210 | -2514 | 0.69 | 0.37 | 0.32 |
| *TNFSF10* | NM_003810 | chr3 | 173724426 | 173724802 | -651 | 0.80 | 0.50 | 0.30 |
| *TNFSF13B* | NM_001145645 | chr13 | 107718626 | 107719123 | -1102 | 0.68 | 0.31 | 0.37 |
| *TNFSF14* | NM_003807 | chr19 | 6621540 | 6621992 | -167 | 0.71 | 0.28 | 0.43 |
| *TNFSF15* | NM_005118 | chr9 | 116610612 | 116610723 | -2438 | 0.65 | 0.18 | 0.48 |
| *TNFSF18* | NM_005092 | chr1 | 171287082 | 171287593 | -611 | 0.94 | 0.63 | 0.31 |
| *TNIP3* | NM_001128843 | chr4 | 122359018 | 122359499 | -2153 | 0.81 | 0.52 | 0.30 |
| *TNK2* | NM_001010938 | chr3 | 197107792 | 197108278 | -1206 | 0.80 | 0.24 | 0.56 |
| *TNMD* | NM_022144 | chrX | 99726057 | 99726596 | -118 | 0.63 | 0.33 | 0.30 |
| *TNNC1* | NM_003280 | chr3 | 52465042 | 52465540 | -2194 | 0.48 | 0.18 | 0.30 |
| *TNNC2* | NM_003279 | chr20 | 43890581 | 43890982 | -1421 | 0.65 | 0.23 | 0.42 |
| *TNNI3K* | NM_015978 | chr1 | 74472812 | 74473328 | -602 | 0.52 | 0.16 | 0.36 |
| *TNP1* | NM_003284 | chr2 | 217433475 | 217433991 | -706 | 0.74 | 0.38 | 0.36 |
| *TNPO1* | NM_002270 | chr5 | 72146306 | 72146799 | -1620 | 0.87 | 0.50 | 0.37 |
| *TNS1* | NM_022648 | chr2 | 218516945 | 218517443 | -153 | 0.64 | 0.34 | 0.30 |
| *TNS3* | NM_022748 | chr7 | 47546063 | 47546564 | -589 | 0.93 | 0.28 | 0.65 |
| *TNS4* | NM_032865 | chr17 | 35911791 | 35912291 | -661 | 0.85 | 0.54 | 0.31 |
| *TNXB* | NM_019105 | chr6 | 32185971 | 32186469 | -1091 | 0.64 | 0.35 | 0.30 |
| *TOMM20L* | NM_207377 | chr14 | 57930514 | 57930608 | -1835 | 0.97 | 0.64 | 0.32 |
| *TOMM22* | NM_020243 | chr22 | 37406511 | 37406697 | -1295 | 0.76 | 0.23 | 0.53 |
| *TOP1P1* | NR_002719 | chr1 | 169572219 | 169572730 | -2183 | 0.61 | 0.21 | 0.40 |
| *TP53* | NM_001126115 | chr17 | 7520361 | 7520851 | -1070 | 0.57 | 0.24 | 0.34 |
| *TP53TG5* | NM_014477 | chr20 | 43439981 | 43440434 | 163 | 0.84 | 0.43 | 0.41 |
| *TPM3* | NM_152263 | chr1 | 152432175 | 152432680 | -1194 | 0.72 | 0.27 | 0.45 |
| *TPP1* | NM_000391 | chr11 | 6599262 | 6599658 | -2192 | 0.83 | 0.49 | 0.34 |
| *TPRX1* | NM_198479 | chr19 | 52999539 | 53000043 | -1118 | 0.79 | 0.49 | 0.30 |
| *TPRXL* | NR_002223 | chr3 | 13951917 | 13952391 | -1653 | 0.62 | 0.30 | 0.32 |
| *TPSD1* | NM_012217 | chr16 | 1244397 | 1244883 | -1633 | 0.69 | 0.36 | 0.32 |
| *TR2IT1* | NM_001039783 | chr3 | 127810975 | 127811477 | -1138 | 0.78 | 0.36 | 0.42 |
| *TRAF4* | NM_004295 | chr17 | 24093178 | 24093458 | -1831 | 0.82 | 0.45 | 0.37 |
| *TRAF5* | NM_145759 | chr1 | 209584351 | 209584640 | -1832 | 0.73 | 0.39 | 0.34 |
| *TRAIP* | NM_005879 | chr3 | 49870341 | 49870845 | -1597 | 0.83 | 0.31 | 0.52 |
| *TREM2* | NM_018965 | chr6 | 41238864 | 41239340 | -202 | 0.91 | 0.55 | 0.37 |
| *TREML1* | NM_178174 | chr6 | 41231556 | 41231939 | -1699 | 0.93 | 0.54 | 0.39 |
| *TREX1* | NM_016381 | chr3 | 48480755 | 48481259 | -1225 | 0.81 | 0.40 | 0.41 |
| *TRIM15* | NM_033229 | chr6 | 30236085 | 30236552 | -2642 | 0.84 | 0.50 | 0.34 |
| *TRIM16L* | NM_001037330 | chr17 | 18565439 | 18565959 | -427 | 0.87 | 0.46 | 0.41 |
| *TRIM31* | NM_007028 | chr6 | 30190721 | 30191262 | -2145 | 0.84 | 0.53 | 0.32 |
| *TRIM40* | NM_138700 | chr6 | 30210048 | 30210521 | -2203 | 0.71 | 0.32 | 0.39 |
| *TRIM56* | NM_030961 | chr7 | 100514639 | 100515099 | -636 | 0.57 | 0.26 | 0.31 |
| *TRIM60* | NM_152620 | chr4 | 166170176 | 166170630 | -2197 | 0.84 | 0.54 | 0.31 |
| *TRIOBP* | NM_001039141 | chr22 | 36421738 | 36422224 | -959 | 0.74 | 0.34 | 0.40 |
| *TRMT12* | NM_017956 | chr8 | 125530800 | 125531295 | -1180 | 0.93 | 0.60 | 0.33 |
| *TRMT61A* | NM_152307 | chr14 | 103063332 | 103063842 | -1674 | 0.64 | 0.33 | 0.32 |
| *TRO* | NM_001039705 | chrX | 54963439 | 54963948 | -279 | 0.80 | 0.48 | 0.32 |
| *TRPM5* | NM_014555 | chr11 | 2400706 | 2401208 | -106 | 0.58 | 0.26 | 0.32 |
| *TRPT1* | NM_001033678 | chr11 | 63750971 | 63751471 | -964 | 0.63 | 0.26 | 0.37 |
| *TRPV2* | NM_016113 | chr17 | 16258891 | 16259385 | -474 | 0.73 | 0.23 | 0.50 |
| *TRPV4* | NM_021625 | chr12 | 108757969 | 108758077 | -2428 | 0.92 | 0.21 | 0.71 |
| *TSEPA* | NM_001010923 | chr6 | 128283361 | 128283859 | -2175 | 0.58 | 0.22 | 0.36 |
| *TSGA10IP* | NM_152762 | chr11 | 65468999 | 65469467 | -457 | 0.84 | 0.51 | 0.33 |
| *TSP50* | NM_013270 | chr3 | 46734727 | 46735146 | -559 | 0.85 | 0.40 | 0.45 |
| *TSPAN1* | NM_005727 | chr1 | 46418208 | 46418722 | -333 | 0.74 | 0.16 | 0.58 |
| *TSPAN10* | NM_031945 | chr17 | 77219236 | 77219762 | -254 | 0.73 | 0.38 | 0.35 |
| *TSPAN17* | NM_001006616 | chr5 | 176005603 | 176006125 | -1129 | 0.85 | 0.31 | 0.54 |
| *TSPAN2* | NM_005725 | chr1 | 115434584 | 115435106 | -1207 | 0.61 | 0.29 | 0.31 |
| *TSPAN31* | NM_005981 | chr12 | 56423127 | 56423420 | -1776 | 0.93 | 0.62 | 0.31 |
| *TSPAN32* | NM_139022 | chr11 | 2279429 | 2279927 | -140 | 0.59 | 0.28 | 0.31 |
| *TSPYL3* | NR_002781 | chr20 | 30242183 | 30242663 | -599 | 0.82 | 0.31 | 0.50 |
| *TSSK1B* | NM_032028 | chr5 | 112799923 | 112800361 | -1577 | 0.97 | 0.52 | 0.45 |
| *TSSK2* | NM_053006 | chr22 | 17497154 | 17497632 | -927 | 0.72 | 0.31 | 0.41 |
| *TTBK1* | NM_032538 | chr6 | 43316783 | 43317317 | -2149 | 0.67 | 0.29 | 0.38 |
| *TTC17* | NM_018259 | chr11 | 43334633 | 43335088 | -2205 | 0.74 | 0.38 | 0.36 |
| *TTC23* | NM_001040655 | chr15 | 97607700 | 97608207 | -615 | 0.75 | 0.40 | 0.35 |
| *TTC3* | NM_003316 | chr21 | 37376482 | 37376970 | -390 | 0.92 | 0.60 | 0.32 |
| *TTC36* | NM_001080441 | chr11 | 117900992 | 117901449 | -2198 | 0.89 | 0.51 | 0.39 |
| *TTC4* | NM_004623 | chr1 | 54953103 | 54953625 | -752 | 0.68 | 0.31 | 0.37 |
| *TTLL3* | NM_001025930 | chr3 | 9824812 | 9825307 | -1843 | 0.76 | 0.45 | 0.31 |
| *TTLL6* | NM_001130918 | chr17 | 44250218 | 44250750 | -1016 | 0.73 | 0.35 | 0.38 |
| *TTPAL* | NM_001039199 | chr20 | 42536785 | 42537327 | -904 | 0.83 | 0.50 | 0.34 |
| *TTTY1* | NR_001538 | chrY | 6317558 | 6318052 | -666 | 0.89 | 0.46 | 0.43 |
| *TTTY11* | NR_001548 | chrY | 8746386 | 8746887 | -1213 | 0.82 | 0.42 | 0.40 |
| *TTTY12* | NR_001551 | chrY | 7730533 | 7731088 | -2153 | 0.89 | 0.43 | 0.46 |
| *TTTY16* | NR_001552 | chrY | 7629279 | 7629802 | -252 | 0.80 | 0.48 | 0.32 |
| *TTTY3* | NR_001524 | chrY | 26283515 | 26284039 | -247 | 0.72 | 0.17 | 0.55 |
| *TTTY3B* | NR_002176 | chrY | 26283515 | 26284039 | -247 | 0.72 | 0.17 | 0.55 |
| *TTTY8* | NR_001533 | chrY | 6402132 | 6402642 | -716 | 0.84 | 0.46 | 0.38 |
| *TTYH2* | NM_052869 | chr17 | 69755955 | 69756453 | 105 | 0.82 | 0.52 | 0.30 |
| *TUB* | NM_003320 | chr11 | 8014766 | 8015267 | -1738 | 0.51 | 0.11 | 0.40 |
| *TWF2* | NM_007284 | chr3 | 52249148 | 52249638 | -1170 | 0.87 | 0.26 | 0.62 |
| *TXLNB* | NM_153235 | chr6 | 139656847 | 139657366 | -2205 | 0.57 | 0.26 | 0.31 |
| *TYROBP* | NM_003332 | chr19 | 41092244 | 41092755 | -1473 | 0.53 | 0.23 | 0.30 |
| *UBA5* | NM_198329 | chr3 | 133853542 | 133854098 | -2159 | 0.83 | 0.46 | 0.38 |
| *UBE2DNL* | NR_024062 | chrX | 84075015 | 84075504 | -552 | 0.90 | 0.60 | 0.30 |
| *UBE2J1* | NM_016021 | chr6 | 90121525 | 90121816 | -2332 | 0.85 | 0.51 | 0.34 |
| *UBE2NL* | NM_001012989 | chrX | 142793977 | 142794378 | -660 | 0.64 | 0.29 | 0.35 |
| *UBE2T* | NM_014176 | chr1 | 200578731 | 200579268 | -1282 | 0.78 | 0.46 | 0.31 |
| *UBL4B* | NM_203412 | chr1 | 110456052 | 110456558 | -279 | 0.67 | 0.12 | 0.55 |
| *UBQLN2* | NM_013444 | chrX | 56605323 | 56605837 | -1216 | 0.96 | 0.52 | 0.44 |
| *UBXN6* | NM_025241 | chr19 | 4411179 | 4411236 | -2417 | 0.74 | 0.44 | 0.30 |
| *UCN2* | NM_033199 | chr3 | 48576154 | 48576664 | -204 | 0.53 | 0.20 | 0.33 |
| *UCN3* | NM_053049 | chr10 | 5395389 | 5395703 | -1429 | 0.74 | 0.32 | 0.42 |
| *UCP1* | NM_021833 | chr4 | 141709871 | 141710369 | -711 | 0.66 | 0.32 | 0.34 |
| *UCP3* | NM_003356 | chr11 | 73398926 | 73399422 | -1244 | 0.84 | 0.08 | 0.76 |
| *UCRC* | NM_001003684 | chr22 | 28492009 | 28492478 | -1113 | 0.56 | 0.22 | 0.33 |
| *UGT1A1* | NM_000463 | chr2 | 234332280 | 234332796 | -1119 | 0.83 | 0.32 | 0.50 |
| *UGT1A3* | NM_019093 | chr2 | 234301120 | 234301632 | -1135 | 0.58 | 0.28 | 0.30 |
| *UGT1A4* | NM_007120 | chr2 | 234291810 | 234292307 | -117 | 0.42 | 0.07 | 0.35 |
| *UGT1A6* | NM_001072 | chr2 | 234265536 | 234266030 | -467 | 0.82 | 0.52 | 0.31 |
| *UGT1A7* | NM_019077 | chr2 | 234254362 | 234254580 | -851 | 0.62 | 0.29 | 0.32 |
| *UGT2B4* | NM_021139 | chr4 | 70397197 | 70397723 | -1245 | 0.89 | 0.54 | 0.35 |
| *UGT2B7* | NM_001074 | chr4 | 69995794 | 69996264 | -752 | 0.68 | 0.30 | 0.37 |
| *UGT3A1* | NM_152404 | chr5 | 36027620 | 36028103 | -605 | 0.84 | 0.48 | 0.35 |
| *ULBP3* | NM_024518 | chr6 | 150432735 | 150433247 | -1096 | 0.76 | 0.36 | 0.40 |
| *UNC119* | NM_005148 | chr17 | 23905148 | 23905682 | -1642 | 0.69 | 0.35 | 0.34 |
| *UNC45B* | NM_001033576 | chr17 | 30498360 | 30498859 | -338 | 0.86 | 0.20 | 0.65 |
| *UNC5CL* | NM_173561 | chr6 | 41116751 | 41117269 | -2104 | 0.90 | 0.20 | 0.70 |
| *UNC84B* | NM_015374 | chr22 | 37483334 | 37483763 | -1655 | 0.87 | 0.33 | 0.54 |
| *UNC93A* | NM_001143947 | chr6 | 167623358 | 167623839 | -1193 | 0.78 | 0.46 | 0.32 |
| *UNQ830* | NM_206895 | chr2 | 233442079 | 233442572 | -911 | 0.90 | 0.33 | 0.57 |
| *UPK1A* | NM_007000 | chr19 | 40848937 | 40849433 | -369 | 0.67 | 0.32 | 0.35 |
| *UPK2* | NM_006760 | chr11 | 118331744 | 118332232 | -247 | 0.74 | 0.27 | 0.47 |
| *UPK3B* | NM_030570 | chr7 | 75977582 | 75978066 | 144 | 0.79 | 0.39 | 0.40 |
| *UPLP* | NM_001114403 | chr7 | 102070374 | 102070786 | -106 | 0.82 | 0.45 | 0.37 |
| *UROC1* | NM_144639 | chr3 | 127719247 | 127719743 | -211 | 0.71 | 0.35 | 0.35 |
| *USE1* | NM_018467 | chr19 | 17184739 | 17185264 | -2152 | 0.61 | 0.24 | 0.37 |
| *USP18* | NM_017414 | chr22 | 17010909 | 17011395 | -1605 | 0.77 | 0.23 | 0.54 |
| *USP2* | NM_171997 | chr11 | 118741748 | 118742264 | -1904 | 0.77 | 0.40 | 0.37 |
| *USP26* | NM_031907 | chrX | 131990915 | 131991440 | -1211 | 0.68 | 0.25 | 0.43 |
| *USP47* | NM_017944 | chr11 | 11818590 | 11819109 | -695 | 0.84 | 0.48 | 0.36 |
| *USP54* | NM_152586 | chr10 | 75007575 | 75007858 | -2277 | 0.93 | 0.48 | 0.45 |
| *UTS2D* | NM_198152 | chr3 | 192533167 | 192533464 | -2296 | 0.63 | 0.33 | 0.31 |
| *VAMP2* | NM_014232 | chr17 | 8008421 | 8008931 | -1658 | 0.71 | 0.32 | 0.40 |
| *VAMP5* | NM_006634 | chr2 | 85664024 | 85664486 | -786 | 0.75 | 0.24 | 0.51 |
| *VARS* | NM_006295 | chr6 | 31873200 | 31873697 | -1757 | 0.66 | 0.26 | 0.40 |
| *VASN* | NM_138440 | chr16 | 4359958 | 4360470 | -1635 | 0.70 | 0.39 | 0.30 |
| *VCY* | NM_004679 | chrY | 14609182 | 14609685 | -1647 | 0.67 | 0.05 | 0.63 |
| *VGLL4* | NM_001128221 | chr3 | 11586706 | 11587217 | -1563 | 0.67 | 0.23 | 0.44 |
| *VIL1* | NM_007127 | chr2 | 218991340 | 218991640 | -591 | 0.89 | 0.57 | 0.32 |
| *VILL* | NM_015873 | chr3 | 38007656 | 38008096 | -2205 | 0.73 | 0.27 | 0.45 |
| *VNN1* | NM_004666 | chr6 | 133076829 | 133077357 | -206 | 0.66 | 0.27 | 0.39 |
| *VPREB1* | NM_007128 | chr22 | 20926806 | 20927345 | -2123 | 0.71 | 0.20 | 0.52 |
| *VPREB3* | NM_013378 | chr22 | 22428060 | 22428149 | -1474 | 0.58 | 0.25 | 0.33 |
| *VPS16* | NM_080414 | chr20 | 2790649 | 2791147 | -1694 | 0.69 | 0.33 | 0.35 |
| *VPS28* | NM_016208 | chr8 | 145626018 | 145626512 | -1530 | 0.80 | 0.50 | 0.30 |
| *VPS41* | NM_014396 | chr7 | 38916915 | 38917433 | -1849 | 0.88 | 0.57 | 0.31 |
| *VRK1* | NM_003384 | chr14 | 96331555 | 96332050 | -1633 | 0.77 | 0.32 | 0.45 |
| *VSIG2* | NM_014312 | chr11 | 124129684 | 124129798 | -2422 | 0.77 | 0.43 | 0.34 |
| *VSIG4* | NM_001100431 | chrX | 65176547 | 65177047 | -105 | 0.78 | 0.20 | 0.58 |
| *VSIG8* | NM_001013661 | chr1 | 158099535 | 158100011 | -702 | 0.88 | 0.37 | 0.51 |
| *VTCN1* | NM_024626 | chr1 | 117556035 | 117556413 | -1152 | 0.69 | 0.22 | 0.47 |
| *VTRNA1-3* | NR_026705 | chr5 | 140085510 | 140085990 | -177 | 0.54 | 0.20 | 0.33 |
| *VWA5A* | NM_001130142 | chr11 | 123490388 | 123490882 | -685 | 0.70 | 0.28 | 0.43 |
| *VWA5B1* | NM_001039500 | chr1 | 20489531 | 20490025 | -220 | 0.66 | 0.36 | 0.30 |
| *VWF* | NM_000552 | chr12 | 6103941 | 6104461 | -104 | 0.89 | 0.40 | 0.48 |
| *WAS* | NM_000377 | chrX | 48426290 | 48426756 | -606 | 0.69 | 0.28 | 0.42 |
| *WBP5* | NM_001006612 | chrX | 102496554 | 102497044 | -1236 | 0.94 | 0.42 | 0.52 |
| *WBSCR26* | NR_026690 | chr7 | 72786765 | 72787226 | -338 | 0.74 | 0.35 | 0.39 |
| *WDR40A* | NM_015397 | chr9 | 34117215 | 34117695 | -700 | 0.84 | 0.32 | 0.52 |
| *WDR41* | NM_018268 | chr5 | 76825545 | 76826042 | -1705 | 0.84 | 0.14 | 0.71 |
| *WDR59* | NM_030581 | chr16 | 73577837 | 73578280 | -1540 | 0.78 | 0.44 | 0.33 |
| *WDR66* | NM_144668 | chr12 | 120838874 | 120839395 | -1727 | 0.73 | 0.43 | 0.30 |
| *WDR73* | NM_032856 | chr15 | 82999882 | 83000372 | -1602 | 0.66 | 0.31 | 0.35 |
| *WDR88* | NM_173479 | chr19 | 38314205 | 38314411 | -529 | 0.86 | 0.44 | 0.42 |
| *WIPF1* | NM_003387 | chr2 | 175208000 | 175208496 | -695 | 0.70 | 0.16 | 0.54 |
| *WNT10A* | NM_025216 | chr2 | 219451630 | 219452114 | -1626 | 0.89 | 0.55 | 0.33 |
| *WNT16* | NM_016087 | chr7 | 120752369 | 120752899 | -22 | 0.68 | 0.31 | 0.37 |
| *WNT2B* | NM_004185 | chr1 | 112809572 | 112810084 | -1734 | 0.62 | 0.24 | 0.37 |
| *WNT3* | NM_030753 | chr17 | 42253020 | 42253512 | -2185 | 0.86 | 0.46 | 0.41 |
| *WNT8A* | NM_058244 | chr5 | 137445234 | 137445681 | -2214 | 0.77 | 0.14 | 0.63 |
| *XCR1* | NM_001024644 | chr3 | 46045671 | 46046207 | -1956 | 0.85 | 0.37 | 0.48 |
| *XIRP1* | NM_194293 | chr3 | 39209927 | 39210398 | -1081 | 0.85 | 0.52 | 0.33 |
| *XK* | NM_021083 | chrX | 37428087 | 37428566 | -1724 | 0.81 | 0.31 | 0.50 |
| *XPO7* | NM_001100162 | chr8 | 21877825 | 21878316 | -1623 | 0.75 | 0.22 | 0.52 |
| *XRCC1* | NM_006297 | chr19 | 48773034 | 48773534 | -1714 | 0.84 | 0.54 | 0.30 |
| *YIF1B* | NM_033557 | chr19 | 43494114 | 43494239 | -1827 | 0.94 | 0.40 | 0.54 |
| *YJEFN3* | NM_198537 | chr19 | 19499394 | 19499902 | -1071 | 0.67 | 0.30 | 0.37 |
| *ZAP70* | NM_207519 | chr2 | 97714861 | 97715314 | -2212 | 0.60 | 0.22 | 0.37 |
| *ZBED1* | NM_004729 | chrY | 2429782 | 2430276 | -1021 | 0.76 | 0.32 | 0.44 |
| *ZBTB47* | NM_145166 | chr3 | 42673437 | 42673904 | -2206 | 0.87 | 0.32 | 0.55 |
| *ZBTB7C* | NM_001039360 | chr18 | 43821856 | 43822366 | -619 | 0.81 | 0.39 | 0.42 |
| *ZC3H12D* | NM_207360 | chr6 | 149848846 | 149849307 | -1353 | 0.65 | 0.35 | 0.31 |
| *ZC3H14* | NM_207662 | chr14 | 88128515 | 88128992 | -1744 | 0.66 | 0.35 | 0.32 |
| *ZDHHC8P* | NR_003950 | chr22 | 22076654 | 22077156 | -2106 | 0.81 | 0.39 | 0.43 |
| *ZEB2* | NM_014795 | chr2 | 144995758 | 144996279 | -1632 | 0.58 | 0.20 | 0.38 |
| *ZFP14* | NM_020917 | chr19 | 41552937 | 41553128 | -2319 | 0.60 | 0.29 | 0.31 |
| *ZFP82* | NM_133466 | chr19 | 41602638 | 41603111 | -1484 | 0.87 | 0.57 | 0.30 |
| *ZFR2* | NM_001145640 | chr19 | 3820378 | 3820839 | -581 | 0.50 | 0.20 | 0.30 |
| *ZHX3* | NM_015035 | chr20 | 39364277 | 39364583 | -2277 | 0.88 | 0.43 | 0.45 |
| *ZIK1* | NM_001010879 | chr19 | 62785061 | 62785550 | -2133 | 0.81 | 0.45 | 0.35 |
| *ZMIZ2* | NM_174929 | chr7 | 44760941 | 44761439 | -1121 | 0.71 | 0.25 | 0.46 |
| *ZMYM4* | NM_005095 | chr1 | 35505272 | 35505781 | -1627 | 0.75 | 0.37 | 0.38 |
| *ZMYND8* | NM_012408 | chr20 | 45420745 | 45421247 | -2115 | 0.62 | 0.33 | 0.30 |
| *ZNF154* | NM_001085384 | chr19 | 62912855 | 62913324 | -698 | 0.85 | 0.54 | 0.32 |
| *ZNF160* | NM_001102603 | chr19 | 58299665 | 58299803 | -1235 | 0.80 | 0.46 | 0.34 |
| *ZNF18* | NM_144680 | chr17 | 11841771 | 11842277 | -610 | 0.77 | 0.39 | 0.38 |
| *ZNF211* | NM_006385 | chr19 | 62835212 | 62835680 | -900 | 0.84 | 0.44 | 0.40 |
| *ZNF275* | NM_001080485 | chrX | 152251729 | 152252193 | -845 | 0.81 | 0.20 | 0.60 |
| *ZNF323* | NM_001135216 | chr6 | 28411840 | 28412354 | -207 | 0.49 | 0.16 | 0.33 |
| *ZNF331* | NM_001079907 | chr19 | 58747941 | 58748448 | -2177 | 0.87 | 0.53 | 0.34 |
| *ZNF34* | NM_030580 | chr8 | 145985868 | 145985992 | -2401 | 0.82 | 0.47 | 0.35 |
| *ZNF384* | NM_001039916 | chr12 | 6671023 | 6671422 | -3136 | 0.78 | 0.45 | 0.33 |
| *ZNF385A* | NM_001130967 | chr12 | 53071298 | 53071799 | -198 | 0.61 | 0.27 | 0.34 |
| *ZNF414* | NM_001146175 | chr19 | 8486387 | 8486578 | -1434 | 0.77 | 0.25 | 0.52 |
| *ZNF48* | NM_152652 | chr16 | 30312982 | 30313476 | -1328 | 0.76 | 0.36 | 0.40 |
| *ZNF498* | NM_145115 | chr7 | 99051549 | 99052054 | -704 | 0.60 | 0.21 | 0.38 |
| *ZNF543* | NM_213598 | chr19 | 62522012 | 62522312 | -1526 | 0.72 | 0.32 | 0.41 |
| *ZNF563* | NM_145276 | chr19 | 12307383 | 12307892 | -2103 | 0.88 | 0.42 | 0.46 |
| *ZNF606* | NM_025027 | chr19 | 63208100 | 63208594 | -1821 | 0.80 | 0.45 | 0.36 |
| *ZNF608* | NM_020747 | chr5 | 124109072 | 124109576 | -620 | 0.65 | 0.26 | 0.39 |
| *ZNF616* | NM_178523 | chr19 | 57337162 | 57337460 | -2308 | 0.75 | 0.41 | 0.34 |
| *ZNF620* | NM_175888 | chr3 | 40521082 | 40521582 | -1201 | 0.85 | 0.55 | 0.30 |
| *ZNF629* | NM_001080417 | chr16 | 30706719 | 30706997 | -834 | 0.65 | 0.34 | 0.31 |
| *ZNF643* | NM_023070 | chr1 | 40685944 | 40686079 | -2353 | 0.81 | 0.51 | 0.30 |
| *ZNF648* | NM_001009992 | chr1 | 180297414 | 180297930 | -202 | 0.73 | 0.29 | 0.44 |
| *ZNF671* | NM_024833 | chr19 | 62931456 | 62931952 | -897 | 0.79 | 0.46 | 0.33 |
| *ZNF683* | NM_001114759 | chr1 | 26571698 | 26572192 | -92 | 0.75 | 0.44 | 0.31 |
| *ZNF70* | NM_021916 | chr22 | 22424913 | 22425413 | -1884 | 0.67 | 0.36 | 0.31 |
| *ZNF705D* | NM_001039615 | chr8 | 11981840 | 11982348 | -2161 | 0.87 | 0.40 | 0.46 |
| *ZNF709* | NM_001145647 | chr19 | 12485475 | 12485975 | -57 | 0.68 | 0.20 | 0.48 |
| *ZNF720* | NM_001130913 | chr16 | 31629627 | 31630168 | -2152 | 0.81 | 0.47 | 0.34 |
| *ZNF781* | NM_152605 | chr19 | 42877154 | 42877394 | -2218 | 0.93 | 0.63 | 0.30 |
| *ZNF788* | NR_027049 | chr19 | 12062562 | 12063102 | -1245 | 0.76 | 0.40 | 0.36 |
| *ZNF826* | NM_001039884 | chr19 | 20400116 | 20400651 | -781 | 0.81 | 0.44 | 0.37 |
| *ZNF831* | NM_178457 | chr20 | 57198892 | 57199378 | -334 | 0.63 | 0.32 | 0.31 |
| *ZNF835* | NM_001005850 | chr19 | 61876118 | 61876553 | -277 | 0.83 | 0.51 | 0.32 |
| *ZNF98* | NM_001098626 | chr19 | 22397254 | 22397666 | -472 | 0.65 | 0.34 | 0.31 |
| *ZP1* | NM_207341 | chr11 | 60389173 | 60389650 | -2178 | 0.47 | 0.16 | 0.31 |
| *ZP3* | NM_007155 | chr7 | 75863809 | 75864316 | -713 | 0.45 | 0.12 | 0.32 |
| *ZSCAN23* | NM_001012455 | chr6 | 28521500 | 28521726 | -2355 | 0.80 | 0.37 | 0.44 |
| *ZWINT* | NM_001005413 | chr10 | 57792514 | 57793021 | -1727 | 0.82 | 0.26 | 0.55 |
