# Supplementary material for: DNA methylation and gene expression profiles show novel regulatory pathways in hepatocellular carcinoma
Source: Clin Epigenetics. 2015 Apr 14;7(1):43. doi: 10.1186/s13148-015-0077-1 (PMC4419480; doi:10.1186/s13148-015-0077-1)
Supplement: Additional file 2: Table S2. — Hypomethylated genes in HCC tissue: 1,243 genes found hypomethylated in HCC as compared to cancer-free tissue. [file 13148_2015_77_MOESM2_ESM.doc]

| **Table S2** | |  |  |  |  |  |  |  |
| --- | --- | --- | --- | --- | --- | --- | --- | --- |
| **HYPOMETHYLATED GENES (n=1243) in HCC** | |  |  |  |  |  |  |  |
|  |  |  | **ROI coordinates** | | |  | **DNA methylation value** |  |
| **Gene symbol** | **RefSeq accession number** | **Chromosome** | **START** | **STOP** | **Transcription Start Site** | **HCC** | **cancer-free** | **difference** |
| *AADACL4* | NM_001013630 | chr1 | 12624723 | 12625062 | -2259 | 0.42 | 0.75 | -0.33 |
| *ABCA9* | NM_080283 | chr17 | 64569145 | 64569631 | -657 | 0.13 | 0.48 | -0.35 |
| *ABCB5* | NM_178559 | chr7 | 20653367 | 20653869 | -122 | 0.07 | 0.42 | -0.35 |
| *ABCC13* | NR_003087 | chr21 | 14567535 | 14568008 | -218 | 0.30 | 0.61 | -0.31 |
| *ABI3BP* | NM_015429 | chr3 | 102196911 | 102197415 | -2139 | 0.10 | 0.57 | -0.46 |
| *ABRA* | NM_139166 | chr8 | 107852121 | 107852440 | -632 | 0.30 | 0.63 | -0.33 |
| *ACCSL* | NM_001031854 | chr11 | 44025222 | 44025740 | -625 | 0.40 | 0.72 | -0.32 |
| *ACN9* | NM_020186 | chr7 | 96582352 | 96582883 | -1222 | 0.16 | 0.58 | -0.42 |
| *ACSM4* | NM_001080454 | chr12 | 7346314 | 7346810 | -1632 | 0.20 | 0.53 | -0.33 |
| *ADAD1* | NM_001159285 | chr4 | 123517149 | 123517694 | -2622 | 0.48 | 0.80 | -0.32 |
| *ADAM2* | NM_001464 | chr8 | 39814923 | 39815142 | -96 | 0.50 | 0.83 | -0.33 |
| *ADAM21* | NM_003813 | chr14 | 69992485 | 69992993 | -1230 | 0.25 | 0.56 | -0.31 |
| *ADAM29* | NM_001130703 | chr4 | 176074199 | 176074712 | -1627 | 0.51 | 0.85 | -0.34 |
| *ADAM6* | NR_002224 | chr14 | 105509243 | 105509767 | -102 | 0.18 | 0.55 | -0.37 |
| *ADAM7* | NM_003817 | chr8 | 24352394 | 24352942 | -1785 | 0.40 | 0.73 | -0.33 |
| *ADAMTS12* | NM_030955 | chr5 | 33929320 | 33929795 | -1676 | 0.22 | 0.79 | -0.57 |
| *ADH4* | NM_000670 | chr4 | 100284412 | 100284921 | -194 | 0.33 | 0.62 | -0.30 |
| *ADHFE1* | NM_144650 | chr8 | 67505586 | 67506091 | -1432 | 0.27 | 0.65 | -0.38 |
| *AFP* | NM_001134 | chr4 | 74519407 | 74519883 | -1151 | 0.17 | 0.52 | -0.35 |
| *AGBL4* | NM_032785 | chr1 | 50263160 | 50263667 | -1200 | 0.22 | 0.68 | -0.46 |
| *AGT* | NM_000029 | chr1 | 228918778 | 228919223 | -2041 | 0.52 | 0.84 | -0.32 |
| *AGXT2* | NM_031900 | chr5 | 35084372 | 35084874 | -790 | 0.38 | 0.84 | -0.46 |
| *AKAP6* | NM_004274 | chr14 | 31865812 | 31866356 | -2145 | 0.01 | 0.38 | -0.36 |
| *ALDH1A1* | NM_000689 | chr9 | 74759660 | 74760130 | -2106 | 0.25 | 0.70 | -0.45 |
| *AMPD3* | NM_001025389 | chr11 | 10432550 | 10433044 | -444 | 0.23 | 0.72 | -0.49 |
| *ANKRD20A2* | NM_001012421 | chr9 | 43125033 | 43125587 | -1770 | 0.28 | 0.60 | -0.32 |
| *ANKRD20A3* | NM_001012419 | chr9 | 43125033 | 43125587 | -1770 | 0.28 | 0.60 | -0.32 |
| *ANO3* | NM_031418 | chr11 | 26309367 | 26309847 | -646 | 0.09 | 0.47 | -0.39 |
| *ANXA3* | NM_005139 | chr4 | 79689880 | 79690418 | -1616 | 0.17 | 0.52 | -0.35 |
| *AOX2P* | NR_001557 | chr2 | 201343114 | 201343642 | -261 | 0.44 | 0.78 | -0.34 |
| *APCS* | NM_001639 | chr1 | 157823286 | 157823761 | -715 | 0.18 | 0.69 | -0.51 |
| *APEX2* | NM_014481 | chrX | 55041078 | 55041537 | -2196 | 0.40 | 0.78 | -0.38 |
| *APOB* | NM_000384 | chr2 | 21122838 | 21122945 | -2441 | 0.17 | 0.69 | -0.52 |
| *APOBEC4* | NM_203454 | chr1 | 181889016 | 181889510 | -192 | 0.35 | 0.76 | -0.41 |
| *APOH* | NM_000042 | chr17 | 61656749 | 61657289 | -1001 | 0.30 | 0.63 | -0.34 |
| *APOL3* | NM_145641 | chr22 | 34892634 | 34893140 | -716 | 0.22 | 0.52 | -0.31 |
| *AQP4* | NM_001650 | chr18 | 22700074 | 22700556 | -601 | 0.43 | 0.82 | -0.39 |
| *ARHGAP19* | NM_032900 | chr10 | 99043692 | 99044209 | -1547 | 0.28 | 0.69 | -0.41 |
| *ARHGAP25* | NM_001007231 | chr2 | 68815084 | 68815582 | -138 | 0.23 | 0.55 | -0.32 |
| *ARHGDIB* | NM_001175 | chr12 | 15005818 | 15006304 | -232 | 0.18 | 0.57 | -0.39 |
| *ARHGEF3* | NM_001128616 | chr3 | 56785731 | 56786253 | -1207 | 0.29 | 0.62 | -0.33 |
| *ARMC3* | NM_173081 | chr10 | 23254528 | 23254986 | -2202 | 0.12 | 0.54 | -0.43 |
| *ASAP1* | NM_018482 | chr8 | 131483922 | 131484421 | -772 | 0.12 | 0.54 | -0.42 |
| *ASB10* | NM_001142459 | chr7 | 150517378 | 150517868 | -2212 | 0.25 | 0.71 | -0.46 |
| *ASB18* | NM_212556 | chr2 | 236838191 | 236838683 | -710 | 0.31 | 0.63 | -0.32 |
| *ASCL4* | NM_203436 | chr12 | 106691682 | 106692193 | -353 | 0.47 | 0.87 | -0.40 |
| *ASNS* | NM_001673 | chr7 | 97340681 | 97341173 | -1137 | 0.43 | 0.79 | -0.35 |
| *ASTN1* | NM_004319 | chr1 | 175401446 | 175401947 | -1049 | 0.12 | 0.62 | -0.50 |
| *ATP10B* | NM_025153 | chr5 | 160211746 | 160212270 | -211 | 0.19 | 0.78 | -0.60 |
| *ATP12A* | NM_001676 | chr13 | 24151843 | 24152349 | -598 | 0.10 | 0.45 | -0.36 |
| *ATP13A4* | NM_032279 | chr3 | 194756326 | 194756806 | -1176 | 0.20 | 0.56 | -0.37 |
| *ATP13A5* | NM_198505 | chr3 | 194579049 | 194579576 | -104 | 0.33 | 0.69 | -0.36 |
| *ATP1A4* | NM_144699 | chr1 | 158386426 | 158386919 | -1302 | 0.42 | 0.78 | -0.37 |
| *ATP5L* | NM_006476 | chr11 | 117774874 | 117775346 | -2203 | 0.33 | 0.68 | -0.34 |
| *ATP6V1G3* | NM_133262 | chr1 | 196777576 | 196778074 | -1127 | 0.40 | 0.74 | -0.34 |
| *ATXN3L* | NM_001135995 | chrX | 13248813 | 13249304 | -619 | 0.51 | 0.90 | -0.39 |
| *AZGP1* | NM_001185 | chr7 | 99412485 | 99413015 | -1127 | 0.43 | 0.75 | -0.31 |
| *B3GALNT1* | NM_001038628 | chr3 | 162307740 | 162308253 | -2142 | 0.29 | 0.68 | -0.39 |
| *BAAT* | NM_001127610 | chr9 | 103186483 | 103186975 | -1107 | 0.32 | 0.70 | -0.38 |
| *BAZ2A* | NM_013449 | chr12 | 55317387 | 55317894 | -1210 | 0.16 | 0.62 | -0.46 |
| *BCAT1* | NM_005504 | chr12 | 24995856 | 24996032 | -2369 | 0.40 | 0.76 | -0.35 |
| *BCL2L15* | NM_001010922 | chr1 | 114231863 | 114232358 | -418 | 0.25 | 0.64 | -0.39 |
| *BEND2* | NM_153346 | chrX | 18150424 | 18150806 | -1670 | 0.27 | 0.72 | -0.44 |
| *BEST3* | NM_152439 | chr12 | 68370706 | 68371224 | -1642 | 0.24 | 0.72 | -0.48 |
| *BEYLA* | NR_027012 | chr8 | 47871285 | 47871800 | -129 | 0.16 | 0.51 | -0.36 |
| *BLK* | NM_001715 | chr8 | 11387564 | 11388082 | -1106 | 0.52 | 0.83 | -0.31 |
| *BLVRA* | NM_000712 | chr7 | 43763433 | 43763955 | -1102 | 0.10 | 0.56 | -0.46 |
| *BMP3* | NM_001201 | chr4 | 82168706 | 82169187 | -2195 | 0.30 | 0.62 | -0.32 |
| *BMP5* | NM_021073 | chr6 | 55850286 | 55850794 | -2206 | 0.16 | 0.51 | -0.35 |
| *BPI* | NM_001725 | chr20 | 36364588 | 36365088 | -1127 | 0.31 | 0.68 | -0.36 |
| *BPY2* | NM_004678 | chrY | 23539306 | 23539840 | -224 | 0.47 | 0.78 | -0.31 |
| *BPY2B* | NM_001002760 | chrY | 23539306 | 23539840 | -224 | 0.47 | 0.78 | -0.31 |
| *BPY2C* | NM_001002761 | chrY | 23539306 | 23539840 | -224 | 0.47 | 0.78 | -0.31 |
| *BTC* | NM_001729 | chr4 | 75939675 | 75940166 | -1014 | 0.17 | 0.72 | -0.54 |
| *BTLA* | NM_001085357 | chr3 | 113700962 | 113701500 | -133 | 0.43 | 0.83 | -0.41 |
| *BTN2A2* | NM_006995 | chr6 | 26488895 | 26489348 | -2210 | 0.00 | 0.41 | -0.41 |
| *C10orf128* | NM_001010863 | chr10 | 50066753 | 50067262 | -594 | 0.15 | 0.65 | -0.50 |
| *C10orf51* | NR_026795 | chr10 | 27271839 | 27272375 | -1171 | 0.51 | 0.81 | -0.31 |
| *C10orf93* | NM_173572 | chr10 | 134608309 | 134608527 | -2364 | 0.41 | 0.78 | -0.37 |
| *C11orf41* | NM_012194 | chr11 | 33518016 | 33518488 | -2200 | 0.36 | 0.67 | -0.31 |
| *C11orf53* | NM_198498 | chr11 | 110630957 | 110631479 | -698 | 0.24 | 0.59 | -0.35 |
| *C11orf70* | NM_032930 | chr11 | 101421750 | 101422249 | -1408 | 0.16 | 0.48 | -0.33 |
| *C12orf12* | NM_152638 | chr12 | 89874772 | 89875256 | -1930 | 0.21 | 0.56 | -0.36 |
| *C12orf54* | NM_152319 | chr12 | 47162353 | 47162842 | -691 | 0.51 | 0.92 | -0.41 |
| *C12orf69* | NM_001013698 | chr12 | 14859793 | 14860290 | -1658 | 0.22 | 0.64 | -0.42 |
| *C12orf74* | NM_001037671 | chr12 | 91620340 | 91620836 | -382 | 0.11 | 0.43 | -0.32 |
| *C13orf26* | NM_152325 | chr13 | 30402876 | 30403364 | -1713 | 0.46 | 0.80 | -0.34 |
| *C13orf30* | NM_182508 | chr13 | 42251802 | 42252325 | -1621 | 0.45 | 0.75 | -0.30 |
| *C13orf33* | NM_032849 | chr13 | 30376838 | 30377371 | -1206 | 0.29 | 0.66 | -0.38 |
| *C13orf39* | NM_001010977 | chr13 | 102146355 | 102146536 | -1590 | 0.44 | 0.76 | -0.33 |
| *C14orf105* | NM_018168 | chr14 | 57031899 | 57032372 | -1806 | 0.02 | 0.82 | -0.80 |
| *C14orf129* | NM_016472 | chr14 | 95913793 | 95914327 | -1714 | 0.24 | 0.57 | -0.32 |
| *C15orf42* | NM_152259 | chr15 | 87918005 | 87918330 | -1653 | 0.30 | 0.62 | -0.31 |
| *C17orf87* | NM_207103 | chr17 | 5080903 | 5081301 | -2295 | 0.18 | 0.52 | -0.34 |
| *C18orf26* | NM_173629 | chr18 | 50408879 | 50409401 | -247 | 0.13 | 0.61 | -0.48 |
| *C18orf62* | NM_001037331 | chr18 | 71268439 | 71268944 | -114 | 0.24 | 0.58 | -0.34 |
| *C1orf114* | NM_021179 | chr1 | 167663765 | 167664249 | -713 | 0.18 | 0.50 | -0.33 |
| *C1orf116* | NM_001083924 | chr1 | 205273618 | 205274118 | -1144 | 0.03 | 0.54 | -0.51 |
| *C1orf150* | NM_145278 | chr1 | 245778183 | 245778692 | -635 | 0.29 | 0.63 | -0.33 |
| *C1orf65* | NM_152610 | chr1 | 221631357 | 221631718 | -1799 | 0.18 | 0.48 | -0.30 |
| *C1orf87* | NM_152377 | chr1 | 60312386 | 60312891 | -624 | 0.44 | 0.74 | -0.30 |
| *C1orf94* | NM_001134734 | chr1 | 34413798 | 34414283 | -1098 | 0.18 | 0.54 | -0.36 |
| *C1QTNF3* | NM_030945 | chr5 | 34081003 | 34081497 | -2176 | 0.38 | 0.73 | -0.35 |
| *C1QTNF7* | NM_031911 | chr4 | 15038279 | 15038777 | -198 | 0.06 | 0.37 | -0.31 |
| *C20orf151* | NM_080833 | chr20 | 60437341 | 60437823 | -1598 | 0.33 | 0.79 | -0.46 |
| *C20orf30* | NM_001009923 | chr20 | 5044075 | 5044161 | -2385 | 0.07 | 0.58 | -0.51 |
| *C20orf79* | NM_178483 | chr20 | 18739944 | 18740479 | -2157 | 0.14 | 0.82 | -0.68 |
| *C21orf54* | NR_024102 | chr21 | 33466266 | 33466778 | -2111 | 0.16 | 0.46 | -0.30 |
| *C21orf82* | NR_027266 | chr21 | 34472432 | 34472966 | -2148 | 0.31 | 0.64 | -0.32 |
| *C21orf94* | NR_027246 | chr21 | 28306626 | 28307121 | -678 | 0.31 | 0.82 | -0.50 |
| *C2orf16* | NM_032266 | chr2 | 27651416 | 27651922 | -1223 | 0.15 | 0.53 | -0.38 |
| *C2orf71* | NM_001029883 | chr2 | 29150471 | 29150996 | -102 | 0.49 | 0.87 | -0.38 |
| *C3orf30* | NM_152539 | chr3 | 120346791 | 120347292 | -644 | 0.36 | 0.71 | -0.36 |
| *C3orf52* | NM_024616 | chr3 | 113285455 | 113286011 | -2138 | 0.05 | 0.36 | -0.31 |
| *C3orf55* | NM_001099777 | chr3 | 158742379 | 158742871 | -1227 | 0.26 | 0.58 | -0.32 |
| *C4BPA* | NM_000715 | chr1 | 205341805 | 205342343 | -2155 | 0.41 | 0.88 | -0.47 |
| *C4orf17* | NM_032149 | chr4 | 100649837 | 100650338 | -1134 | 0.24 | 0.62 | -0.38 |
| *C5orf46* | NM_206966 | chr5 | 147268277 | 147268667 | -2178 | 0.12 | 0.91 | -0.80 |
| *C5orf49* | NM_001089584 | chr5 | 7906506 | 7906728 | -2353 | 0.43 | 0.89 | -0.46 |
| *C6orf57* | NM_145267 | chr6 | 71330906 | 71331363 | -2210 | 0.27 | 0.65 | -0.39 |
| *C8orf22* | NM_001007176 | chr8 | 50146130 | 50146465 | -1157 | 0.27 | 0.62 | -0.35 |
| *C8orf34* | NM_052958 | chr8 | 69511669 | 69512186 | -773 | 0.40 | 0.92 | -0.52 |
| *C8orf85* | NM_001025357 | chr8 | 118017242 | 118017693 | -2176 | 0.20 | 0.59 | -0.40 |
| *C9orf144* | NR_024481 | chr9 | 34828458 | 34828949 | -120 | 0.34 | 0.68 | -0.34 |
| *C9orf71* | NM_153237 | chr9 | 70345963 | 70346476 | -616 | 0.49 | 0.82 | -0.33 |
| *C9orf84* | NM_001080551 | chr9 | 113561484 | 113561996 | -106 | 0.47 | 0.83 | -0.36 |
| *CACNA2D4* | NM_172364 | chr12 | 1900495 | 1900573 | -2403 | 0.39 | 0.87 | -0.48 |
| *CACNB2* | NM_201590 | chr10 | 18669057 | 18669566 | -307 | 0.38 | 0.70 | -0.32 |
| *CACNG2* | NM_006078 | chr22 | 35430249 | 35430793 | -1672 | 0.53 | 0.83 | -0.30 |
| *CALB1* | NM_004929 | chr8 | 91166083 | 91166583 | -2050 | 0.11 | 0.48 | -0.37 |
| *CALD1* | NM_004342 | chr7 | 134113225 | 134113749 | -1223 | 0.12 | 0.45 | -0.33 |
| *CAMK1G* | NM_020439 | chr1 | 207823198 | 207823701 | -217 | 0.15 | 0.48 | -0.33 |
| *CAPN13* | NM_144575 | chr2 | 30884684 | 30885173 | -1113 | 0.53 | 0.92 | -0.39 |
| *CAPN3* | NM_212464 | chr15 | 40425160 | 40425705 | -2159 | 0.33 | 0.63 | -0.30 |
| *CAPN6* | NM_014289 | chrX | 110401753 | 110402279 | -1609 | 0.20 | 0.59 | -0.39 |
| *CAPN9* | NM_006615 | chr1 | 228947335 | 228947772 | -2198 | 0.28 | 0.65 | -0.36 |
| *CAPSL* | NM_001042625 | chr5 | 35974937 | 35975458 | -703 | 0.60 | 0.92 | -0.32 |
| *CARD17* | NM_001007232 | chr11 | 104478396 | 104478890 | -1275 | 0.32 | 0.64 | -0.32 |
| *CARTPT* | NM_004291 | chr5 | 71049269 | 71049786 | -1221 | 0.42 | 0.82 | -0.40 |
| *CASP5* | NM_001136109 | chr11 | 104399476 | 104400022 | -644 | 0.50 | 0.83 | -0.32 |
| *CASQ1* | NM_001231 | chr1 | 158425033 | 158425541 | -1621 | 0.21 | 0.62 | -0.42 |
| *CCDC102B* | NM_024781 | chr18 | 64613867 | 64614331 | -2197 | 0.15 | 0.63 | -0.48 |
| *CCDC126* | NM_138771 | chr7 | 23601082 | 23601556 | -2203 | 0.24 | 0.71 | -0.48 |
| *CCDC53* | NM_016053 | chr12 | 100981827 | 100982318 | -2043 | 0.64 | 0.98 | -0.33 |
| *CCDC60* | NM_178499 | chr12 | 118254470 | 118255009 | -2159 | 0.18 | 0.66 | -0.49 |
| *CCL1* | NM_002981 | chr17 | 29714817 | 29715123 | -605 | 0.20 | 0.95 | -0.75 |
| *CCL5* | NM_002985 | chr17 | 31231433 | 31231958 | -205 | 0.39 | 0.75 | -0.35 |
| *CCL7* | NM_006273 | chr17 | 29619897 | 29620415 | -1196 | 0.62 | 0.96 | -0.34 |
| *CCNH* | NM_001239 | chr5 | 86745054 | 86745556 | -713 | 0.24 | 0.61 | -0.38 |
| *CCR5* | NM_000579 | chr3_random | 663752 | 664070 | -1003 | 0.32 | 0.62 | -0.30 |
| *CD1B* | NM_001764 | chr1 | 156568800 | 156569312 | -1111 | 0.04 | 0.34 | -0.30 |
| *CD1C* | NM_001765 | chr1 | 156525729 | 156526230 | -206 | 0.25 | 0.89 | -0.64 |
| *CD2* | NM_001767 | chr1 | 117098167 | 117098656 | -196 | 0.10 | 0.44 | -0.34 |
| *CD200* | NM_001004196 | chr3 | 113533414 | 113533766 | -1015 | 0.26 | 0.60 | -0.34 |
| *CD22* | NM_001771 | chr19 | 40511427 | 40511952 | -228 | 0.45 | 0.88 | -0.44 |
| *CD247* | NM_000734 | chr1 | 165755735 | 165756239 | -1516 | 0.45 | 0.88 | -0.43 |
| *CD300LB* | NM_174892 | chr17 | 70040597 | 70041073 | -1627 | 0.32 | 0.62 | -0.30 |
| *CD34* | NM_001025109 | chr1 | 206153077 | 206153579 | -2022 | 0.31 | 0.71 | -0.40 |
| *CD36* | NM_001001547 | chr7 | 80067003 | 80067561 | -2157 | 0.25 | 0.78 | -0.53 |
| *CD3D* | NM_000732 | chr11 | 117720497 | 117720983 | -2071 | 0.30 | 0.60 | -0.30 |
| *CD40LG* | NM_000074 | chrX | 135557130 | 135557618 | -627 | 0.53 | 0.93 | -0.39 |
| *CD44* | NM_000610 | chr11 | 35116562 | 35117052 | -185 | 0.23 | 0.59 | -0.36 |
| *CD48* | NM_001778 | chr1 | 158948171 | 158948657 | -205 | 0.27 | 0.75 | -0.48 |
| *CD53* | NM_000560 | chr1 | 111216939 | 111217437 | -56 | 0.05 | 0.50 | -0.45 |
| *CD69* | NM_001781 | chr12 | 9805637 | 9806115 | -1112 | 0.17 | 0.56 | -0.39 |
| *CD86* | NM_006889 | chr3 | 123277478 | 123277977 | -1711 | 0.24 | 0.60 | -0.36 |
| *CDC42SE1* | NM_001038707 | chr1 | 149299892 | 149300386 | -1390 | 0.04 | 0.43 | -0.39 |
| *CDH13* | NM_001257 | chr16 | 81217219 | 81217730 | -603 | 0.22 | 0.56 | -0.34 |
| *CDH17* | NM_004063 | chr8 | 95290831 | 95291387 | -1118 | 0.16 | 0.55 | -0.39 |
| *CDH26* | NM_021810 | chr20 | 58004350 | 58004836 | -230 | 0.29 | 0.65 | -0.37 |
| *CDX4* | NM_005193 | chrX | 72581394 | 72581835 | -2199 | 0.35 | 0.69 | -0.33 |
| *CEACAM18* | NM_001080405 | chr19 | 56669309 | 56669824 | -2082 | 0.32 | 0.86 | -0.54 |
| *CECR7* | NR_015352 | chr22 | 15896488 | 15896952 | -739 | 0.26 | 0.77 | -0.50 |
| *CECR8* | NR_003607 | chr22 | 15685948 | 15686495 | -2141 | 0.41 | 0.74 | -0.33 |
| *CER1* | NM_005454 | chr9 | 14713059 | 14713563 | -596 | 0.49 | 0.86 | -0.37 |
| *CES1* | NM_001025194 | chr16 | 54425418 | 54425928 | -1097 | 0.37 | 0.70 | -0.33 |
| *CGB* | NM_000737 | chr19 | 54219347 | 54219851 | -194 | 0.29 | 0.60 | -0.31 |
| *CGB1* | NM_033377 | chr19 | 54233082 | 54233577 | -1326 | 0.22 | 0.53 | -0.31 |
| *CGB2* | NM_033378 | chr19 | 54225501 | 54226014 | -1183 | 0.33 | 0.62 | -0.30 |
| *CHIA* | NM_021797 | chr1 | 111632566 | 111633017 | -2214 | 0.09 | 0.68 | -0.59 |
| *CHRNB2* | NM_000748 | chr1 | 152805432 | 152805960 | -1184 | 0.41 | 0.72 | -0.32 |
| *CHST6* | NM_021615 | chr16 | 74088724 | 74088840 | -2355 | 0.11 | 0.47 | -0.36 |
| *CIB2* | NM_006383 | chr15 | 76213186 | 76213400 | -2360 | 0.28 | 0.65 | -0.38 |
| *CKMT1A* | NM_001015001 | chr15 | 41771609 | 41772086 | -527 | 0.45 | 0.85 | -0.39 |
| *CLCA1* | NM_001285 | chr1 | 86705233 | 86705758 | -1617 | 0.05 | 0.58 | -0.53 |
| *CLDN8* | NM_199328 | chr21 | 30510567 | 30511068 | -628 | 0.30 | 0.82 | -0.51 |
| *CLEC1B* | NM_001099431 | chr12 | 10045181 | 10045581 | -2215 | 0.22 | 0.61 | -0.39 |
| *CLEC4D* | NM_080387 | chr12 | 8557211 | 8557701 | 54 | 0.32 | 0.65 | -0.33 |
| *CLEC4E* | NM_014358 | chr12 | 8585146 | 8585627 | -561 | 0.14 | 0.61 | -0.47 |
| *CLEC6A* | NM_001007033 | chr12 | 8499369 | 8499870 | -237 | 0.54 | 0.87 | -0.33 |
| *CLECL1* | NM_172004 | chr12 | 9778041 | 9778322 | -1054 | 0.42 | 0.72 | -0.30 |
| *CLIC5* | NM_001114086 | chr6 | 46156989 | 46157507 | -1204 | 0.37 | 0.78 | -0.41 |
| *CLRN1OS* | NR_024066 | chr3 | 152171264 | 152171793 | -1625 | 0.42 | 0.77 | -0.35 |
| *CMA1* | NM_001836 | chr14 | 24048255 | 24048773 | -1203 | 0.19 | 0.51 | -0.32 |
| *CMTM5* | NM_001037288 | chr14 | 22914964 | 22915447 | -650 | 0.30 | 0.62 | -0.33 |
| *CNBD1* | NM_173538 | chr8 | 87946875 | 87947378 | -664 | 0.31 | 0.72 | -0.41 |
| *CNGA2* | NM_005140 | chrX | 150653489 | 150654001 | -128 | 0.41 | 0.81 | -0.41 |
| *CNGB3* | NM_019098 | chr8 | 87826894 | 87827397 | -2128 | 0.16 | 0.49 | -0.33 |
| *CNTN1* | NM_001843 | chr12 | 39370758 | 39371258 | -1616 | 0.44 | 0.75 | -0.31 |
| *CNTN3* | NM_020872 | chr3 | 74653975 | 74654481 | -1195 | 0.47 | 0.90 | -0.43 |
| *CNTN6* | NM_014461 | chr3 | 1107771 | 1108245 | -1620 | 0.42 | 0.80 | -0.38 |
| *COL10A1* | NM_000493 | chr6 | 116556020 | 116556441 | -2241 | 0.06 | 0.39 | -0.33 |
| *COL29A1* | NM_153264 | chr3 | 131545564 | 131546064 | -1234 | 0.17 | 0.53 | -0.36 |
| *COL3A1* | NM_000090 | chr2 | 189545486 | 189545983 | -1608 | 0.48 | 0.86 | -0.38 |
| *COL5A2* | NM_000393 | chr2 | 189754775 | 189755275 | -2175 | 0.11 | 0.75 | -0.64 |
| *COL6A6* | NM_001102608 | chr3 | 131761498 | 131761979 | -128 | 0.27 | 0.68 | -0.41 |
| *COLEC10* | NM_006438 | chr8 | 120147967 | 120148462 | -411 | 0.32 | 0.62 | -0.30 |
| *COLQ* | NM_005677 | chr3 | 15540199 | 15540687 | -2181 | 0.18 | 0.53 | -0.35 |
| *COX7C* | NM_001867 | chr5 | 85949023 | 85949499 | -278 | 0.17 | 0.56 | -0.40 |
| *CPA2* | NM_001869 | chr7 | 129693762 | 129694274 | 80 | 0.08 | 0.38 | -0.30 |
| *CPA3* | NM_001870 | chr3 | 150063768 | 150064299 | -1698 | 0.10 | 0.43 | -0.32 |
| *CPA4* | NM_016352 | chr7 | 129719753 | 129720242 | -211 | 0.21 | 0.56 | -0.35 |
| *CPA5* | NM_001127441 | chr7 | 129769929 | 129770122 | -1839 | 0.25 | 0.56 | -0.31 |
| *CPA6* | NM_001127445 | chr8 | 68821131 | 68821621 | -202 | 0.33 | 0.69 | -0.36 |
| *CPEB1* | NM_001079533 | chr15 | 81039114 | 81039592 | -1725 | 0.12 | 0.74 | -0.62 |
| *CPO* | NM_173077 | chr2 | 207510104 | 207510657 | -2141 | 0.56 | 0.86 | -0.30 |
| *CPOX* | NM_000097 | chr3 | 99796560 | 99797056 | -1677 | 0.20 | 0.50 | -0.30 |
| *CR1* | NM_000573 | chr1 | 205734205 | 205734718 | -1633 | 0.30 | 0.80 | -0.51 |
| *CRB1* | NM_201253 | chr1 | 195502061 | 195502595 | -1702 | 0.45 | 0.78 | -0.33 |
| *CRCT1* | NM_019060 | chr1 | 150751182 | 150751724 | -2148 | 0.27 | 0.84 | -0.56 |
| *CRH* | NM_000756 | chr8 | 67253861 | 67254371 | -864 | 0.20 | 0.51 | -0.31 |
| *CRHBP* | NM_001882 | chr5 | 76282009 | 76282557 | -2152 | 0.49 | 0.80 | -0.31 |
| *CRISPLD1* | NM_031461 | chr8 | 76058032 | 76058524 | -1119 | 0.09 | 0.39 | -0.30 |
| *CRNN* | NM_016190 | chr1 | 150653236 | 150653614 | -51 | 0.38 | 0.76 | -0.37 |
| *CRYBA2* | NM_005209 | chr2 | 219567862 | 219568355 | -1743 | 0.19 | 0.56 | -0.37 |
| *CRYM* | NM_001014444 | chr16 | 21222401 | 21222884 | -724 | 0.33 | 0.65 | -0.32 |
| *CSNK2A1* | NM_001895 | chr20 | 474350 | 474842 | -2114 | 0.11 | 0.49 | -0.38 |
| *CST11* | NM_080830 | chr20 | 23383805 | 23383926 | -2383 | 0.28 | 0.64 | -0.36 |
| *CST8* | NM_005492 | chr20 | 23417327 | 23417874 | -2164 | 0.28 | 0.79 | -0.51 |
| *CST9L* | NM_080610 | chr20 | 23497328 | 23497842 | -199 | 0.60 | 0.92 | -0.32 |
| *CSTA* | NM_005213 | chr3 | 123524709 | 123525135 | -1778 | 0.32 | 0.62 | -0.30 |
| *CTAGE4* | NM_198495 | chr7 | 143599619 | 143599821 | -2395 | 0.12 | 0.43 | -0.31 |
| *CTAGE6* | NM_178561 | chr7 | 143086753 | 143087254 | -1227 | 0.17 | 0.50 | -0.33 |
| *CTBP2* | NM_022802 | chr10 | 126707546 | 126707719 | -1189 | 0.30 | 0.67 | -0.37 |
| *CTLA4* | NM_001037631 | chr2 | 204439851 | 204440366 | -644 | 0.21 | 0.58 | -0.38 |
| *CTXN3* | NM_001127385 | chr5 | 127015125 | 127015622 | -1231 | 0.33 | 0.64 | -0.31 |
| *CUGBP2* | NM_001025076 | chr10 | 11245141 | 11245643 | -1606 | 0.39 | 0.71 | -0.32 |
| *CXADRP2* | NR_024387 | chr15 | 19270108 | 19270606 | -102 | 0.50 | 0.92 | -0.41 |
| *CXCL10* | NM_001565 | chr4 | 77163623 | 77164137 | -206 | 0.19 | 0.55 | -0.36 |
| *CXorf22* | NM_152632 | chrX | 35846982 | 35847447 | -563 | 0.34 | 0.76 | -0.42 |
| *CXorf67* | NM_203407 | chrX | 51165375 | 51165860 | -888 | 0.35 | 0.65 | -0.30 |
| *CYP19A1* | NM_000103 | chr15 | 49418532 | 49419027 | -692 | 0.21 | 0.57 | -0.36 |
| *CYTIP* | NM_004288 | chr2 | 158008799 | 158009294 | -196 | 0.52 | 0.86 | -0.34 |
| *DAD1L* | NR_024064 | chr12 | 24964433 | 24964920 | -2143 | 0.18 | 0.59 | -0.41 |
| *DAPK2* | NM_014326 | chr15 | 62127938 | 62128038 | -2414 | 0.33 | 0.68 | -0.35 |
| *DAPP1* | NM_014395 | chr4 | 100955078 | 100955591 | -1668 | 0.30 | 0.60 | -0.30 |
| *DCHS2* | NM_017639 | chr4 | 155531749 | 155532263 | -107 | 0.48 | 0.77 | -0.30 |
| *DCLK1* | NM_004734 | chr13 | 35604837 | 35605355 | -1632 | 0.02 | 0.36 | -0.33 |
| *DCLK3* | NM_033403 | chr3 | 36757829 | 36758323 | -1720 | 0.59 | 0.91 | -0.31 |
| *DCN* | NM_001920 | chr12 | 90101920 | 90102413 | -1229 | 0.14 | 0.58 | -0.43 |
| *DDR2* | NM_001014796 | chr1 | 160866863 | 160867375 | -1732 | 0.30 | 0.82 | -0.52 |
| *DDX53* | NM_182699 | chrX | 22927966 | 22928463 | 207 | 0.52 | 0.83 | -0.31 |
| *DEF109P1B* | NR_003668 | chr8 | 7155351 | 7155890 | -2156 | 0.47 | 0.79 | -0.31 |
| *DEFB105A* | NM_152250 | chr8 | 7330670 | 7331182 | -1726 | 0.57 | 0.90 | -0.33 |
| *DEFB105B* | NM_001040703 | chr8 | 7330670 | 7331182 | -1726 | 0.57 | 0.90 | -0.33 |
| *DEFB106A* | NM_152251 | chr8 | 7333185 | 7333665 | -2106 | 0.25 | 0.64 | -0.39 |
| *DEFB106B* | NM_001040704 | chr8 | 7333185 | 7333665 | -2106 | 0.25 | 0.64 | -0.39 |
| *DEFB108B* | NM_001002035 | chr11 | 71221582 | 71222047 | -78 | 0.11 | 0.42 | -0.32 |
| *DEFB109P1* | NR_024044 | chr8 | 12304218 | 12304686 | -2207 | 0.33 | 0.74 | -0.42 |
| *DEFB113* | NM_001037729 | chr6 | 50045160 | 50045658 | -112 | 0.34 | 0.77 | -0.43 |
| *DEFB121* | NM_001011878 | chr20 | 29458976 | 29459189 | -1431 | 0.17 | 0.56 | -0.39 |
| *DEFB125* | NM_153325 | chr20 | 15280 | 15776 | -822 | 0.37 | 0.68 | -0.31 |
| *DEFB128* | NM_001037732 | chr20 | 118618 | 119117 | -603 | 0.14 | 0.51 | -0.37 |
| *DEFB129* | NM_080831 | chr20 | 154055 | 154447 | -1673 | 0.11 | 0.44 | -0.33 |
| *DEFB130* | NM_001037804 | chr8 | 11966606 | 11967134 | -205 | 0.40 | 0.78 | -0.38 |
| *DEFB131* | NM_001040448 | chr4 | 9052939 | 9053390 | -2192 | 0.21 | 0.55 | -0.34 |
| *DEFB134* | NM_001033019 | chr8 | 11892030 | 11892558 | -1125 | 0.41 | 0.71 | -0.30 |
| *DEFB4* | NM_004942 | chr8 | 7789489 | 7789998 | 135 | 0.43 | 0.74 | -0.32 |
| *DERA* | NM_015954 | chr12 | 15953037 | 15953577 | -2145 | 0.45 | 0.77 | -0.32 |
| *DHRS9* | NM_005771 | chr2 | 169629517 | 169630013 | 221 | 0.47 | 0.79 | -0.31 |
| *DISC2* | NR_002227 | chr1 | 230020751 | 230021241 | -110 | 0.53 | 0.87 | -0.34 |
| *DLG2* | NM_001142702 | chr11 | 83071972 | 83072468 | -1104 | 0.38 | 0.71 | -0.34 |
| *DLGAP3* | NM_001080418 | chr1 | 35145966 | 35146035 | -2429 | 0.37 | 0.78 | -0.42 |
| *DNAI2* | NM_023036 | chr17 | 69781134 | 69781638 | -605 | 0.45 | 0.81 | -0.36 |
| *DNAJC18* | NM_152686 | chr5 | 138804994 | 138805494 | -2206 | 0.28 | 0.79 | -0.50 |
| *DOCK8* | NM_203447 | chr9 | 260607 | 261056 | -2215 | 0.32 | 0.74 | -0.41 |
| *DPCR1* | NM_080870 | chr6 | 31026614 | 31027106 | -677 | 0.23 | 0.53 | -0.30 |
| *DPP4* | NM_001935 | chr2 | 162641531 | 162641716 | -2325 | 0.08 | 0.49 | -0.41 |
| *DRD3* | NM_000796 | chr3 | 115380443 | 115380948 | -106 | 0.24 | 0.54 | -0.30 |
| *DSC1* | NM_004948 | chr18 | 26999185 | 26999266 | -2408 | 0.33 | 0.73 | -0.40 |
| *DSG1* | NM_001942 | chr18 | 27150560 | 27151064 | -1237 | 0.20 | 0.60 | -0.40 |
| *DSPP* | NM_014208 | chr4 | 88747847 | 88748354 | -603 | 0.27 | 0.57 | -0.30 |
| *DST* | NM_183380 | chr6 | 56818319 | 56818818 | -2146 | 0.15 | 0.49 | -0.35 |
| *DTNA* | NM_032978 | chr18 | 30542730 | 30543265 | -1195 | 0.30 | 0.61 | -0.32 |
| *DTX4* | NM_015177 | chr11 | 58696035 | 58696521 | -109 | 0.25 | 0.55 | -0.31 |
| *DUSP27* | NM_001080426 | chr1 | 165330688 | 165331181 | 224 | 0.11 | 0.46 | -0.35 |
| *DYRK4* | NM_003845 | chr12 | 4568827 | 4569305 | -438 | 0.31 | 0.62 | -0.31 |
| *DYSF* | NM_001130976 | chr2 | 71532673 | 71533142 | -1352 | 0.10 | 0.49 | -0.39 |
| *EDN1* | NM_001955 | chr6 | 12397046 | 12397531 | -1225 | 0.48 | 0.82 | -0.34 |
| *EEF1DP3* | NR_027062 | chr13 | 31316931 | 31317448 | -1729 | 0.10 | 0.49 | -0.39 |
| *EGF* | NM_001963 | chr4 | 111052464 | 111052956 | -778 | 0.25 | 0.56 | -0.32 |
| *EHBP1* | NM_001142615 | chr2 | 62752626 | 62753153 | -1626 | 0.16 | 0.47 | -0.31 |
| *EHD3* | NM_014600 | chr2 | 31308824 | 31309293 | -1647 | 0.19 | 0.49 | -0.30 |
| *EHF* | NM_012153 | chr11 | 34598352 | 34598862 | -636 | 0.12 | 0.44 | -0.32 |
| *ELAVL4* | NM_001144777 | chr1 | 50285935 | 50286430 | -89 | 0.11 | 0.41 | -0.30 |
| *ELMO2* | NM_133171 | chr20 | 44469560 | 44470067 | -1135 | 0.13 | 0.46 | -0.33 |
| *EMR1* | NM_001974 | chr19 | 6838561 | 6839058 | 228 | 0.35 | 0.69 | -0.35 |
| *EMR3* | NM_032571 | chr19 | 14646873 | 14647172 | -292 | 0.18 | 0.49 | -0.31 |
| *EPHA3* | NM_005233 | chr3 | 89236947 | 89237472 | -2153 | 0.63 | 0.93 | -0.30 |
| *EPHA6* | NM_173655 | chr3 | 98639711 | 98640191 | -1228 | 0.20 | 0.61 | -0.41 |
| *ESM1* | NM_001135604 | chr5 | 54317110 | 54317510 | -139 | 0.25 | 0.56 | -0.32 |
| *ETS1* | NM_001143820 | chr11 | 127963573 | 127964077 | -1162 | 0.22 | 0.73 | -0.52 |
| *EXT2* | NM_000401 | chr11 | 44071798 | 44072291 | -1629 | 0.31 | 0.67 | -0.36 |
| *EYA1* | NM_172058 | chr8 | 72433004 | 72433529 | -1733 | 0.06 | 0.41 | -0.34 |
| *FABP12* | NM_001105281 | chr8 | 82606557 | 82607104 | -725 | 0.09 | 0.64 | -0.54 |
| *FAF1* | NM_007051 | chr1 | 51200815 | 51201006 | -2386 | 0.12 | 0.56 | -0.44 |
| *FAM107B* | NM_031453 | chr10 | 14857342 | 14857902 | -720 | 0.43 | 0.73 | -0.30 |
| *FAM10A4* | NR_002183 | chr13 | 49643765 | 49644253 | -145 | 0.46 | 0.76 | -0.30 |
| *FAM138A* | NR_026818 | chr19 | 29108 | 29571 | -649 | 0.26 | 0.56 | -0.30 |
| *FAM138C* | NR_026822 | chr19 | 29108 | 29571 | -649 | 0.26 | 0.56 | -0.30 |
| *FAM138D* | NR_026823 | chr12 | 20605 | 21070 | -1164 | 0.20 | 0.49 | -0.30 |
| *FAM138E* | NR_026819 | chr15 | 100311731 | 100312183 | -653 | 0.18 | 0.49 | -0.30 |
| *FAM138F* | NR_026820 | chr19 | 29108 | 29571 | -649 | 0.26 | 0.56 | -0.30 |
| *FAM164A* | NM_016010 | chr8 | 79738413 | 79738970 | -2144 | 0.35 | 0.73 | -0.37 |
| *FAM182A* | NR_026713 | chr20 | 25982470 | 25982980 | -524 | 0.26 | 0.59 | -0.32 |
| *FAM182B* | NR_027061 | chr20 | 25726323 | 25726816 | -1691 | 0.33 | 0.67 | -0.34 |
| *FAM183B* | NM_001105282 | chr7 | 38693661 | 38694160 | -696 | 0.32 | 0.64 | -0.32 |
| *FAM55A* | NM_152315 | chr11 | 113937143 | 113937650 | -1606 | 0.40 | 0.71 | -0.32 |
| *FAM5B* | NM_021165 | chr1 | 175405377 | 175405906 | -1613 | 0.40 | 0.73 | -0.32 |
| *FAM65B* | NM_014722 | chr6 | 25020645 | 25021134 | -1715 | 0.20 | 0.54 | -0.34 |
| *FAM66A* | NR_026789 | chr8 | 12262368 | 12262804 | -1312 | 0.41 | 0.73 | -0.32 |
| *FAM66C* | NR_026788 | chr12 | 8222007 | 8222539 | -1798 | 0.58 | 0.93 | -0.35 |
| *FAM81B* | NM_152548 | chr5 | 94751570 | 94752055 | -990 | 0.09 | 0.48 | -0.39 |
| *FAM92A3* | NR_003612 | chr4 | 184195422 | 184195944 | -128 | 0.48 | 0.85 | -0.37 |
| *FAT3* | NM_001008781 | chr11 | 91724481 | 91724954 | -191 | 0.49 | 0.82 | -0.33 |
| *FBXL5* | NM_012161 | chr4 | 15267534 | 15268052 | -1682 | 0.21 | 0.67 | -0.46 |
| *FBXL7* | NM_012304 | chr5 | 15551424 | 15551953 | -1615 | 0.10 | 0.51 | -0.42 |
| *FBXO32* | NM_148177 | chr8 | 124614433 | 124614912 | -1108 | 0.22 | 0.54 | -0.32 |
| *FBXW8* | NM_012174 | chr12 | 115831552 | 115832101 | -1316 | 0.38 | 0.75 | -0.36 |
| *FCAMR* | NM_001122979 | chr1 | 205212605 | 205212992 | -2205 | 0.20 | 0.65 | -0.44 |
| *FCER1A* | NM_002001 | chr1 | 157524558 | 157525049 | -1323 | 0.17 | 0.73 | -0.55 |
| *FCGR1A* | NM_000566 | chr1 | 148019539 | 148020061 | -1073 | 0.09 | 0.39 | -0.31 |
| *FCGR1C* | NM_001128589 | chr1 | 147634505 | 147635018 | -1155 | 0.07 | 0.45 | -0.38 |
| *FCGR3A* | NM_000569 | chr1 | 159788913 | 159789465 | -2747 | 0.18 | 0.57 | -0.39 |
| *FCRL1* | NM_001159397 | chr1 | 156056504 | 156057026 | -201 | 0.17 | 0.50 | -0.32 |
| *FCRL3* | NM_052939 | chr1 | 155937218 | 155937727 | -201 | 0.59 | 0.92 | -0.33 |
| *FCRL4* | NM_031282 | chr1 | 155834951 | 155835455 | -709 | 0.56 | 0.90 | -0.34 |
| *FCRL5* | NM_031281 | chr1 | 155788874 | 155789399 | -202 | 0.34 | 0.70 | -0.36 |
| *FCRL6* | NM_001004310 | chr1 | 158036906 | 158037229 | -1728 | 0.42 | 0.74 | -0.32 |
| *FER1L6* | NM_001039112 | chr8 | 124930987 | 124931521 | -2153 | 0.18 | 0.77 | -0.59 |
| *FGF12* | NM_004113 | chr3 | 193929951 | 193930431 | -2109 | 0.33 | 0.72 | -0.39 |
| *FGF20* | NM_019851 | chr8 | 16905775 | 16906284 | -1984 | 0.07 | 0.43 | -0.37 |
| *FGF23* | NM_020638 | chr12 | 4359095 | 4359603 | -194 | 0.47 | 0.78 | -0.30 |
| *FGF6* | NM_020996 | chr12 | 4426000 | 4426500 | -1209 | 0.20 | 0.54 | -0.34 |
| *FGFBP1* | NM_005130 | chr4 | 15549932 | 15550421 | -1107 | 0.27 | 0.77 | -0.49 |
| *FGG* | NM_000509 | chr4 | 155755223 | 155755702 | -2110 | 0.06 | 0.40 | -0.34 |
| *FHIT* | NM_002012 | chr3 | 61213643 | 61214114 | -1705 | 0.35 | 0.67 | -0.32 |
| *FKSG83* | NM_032030 | chr6 | 27399557 | 27400054 | -750 | 0.32 | 0.77 | -0.45 |
| *FKTN* | NM_001079802 | chr9 | 107358768 | 107359242 | -1226 | 0.21 | 0.54 | -0.33 |
| *FLG* | NM_002016 | chr1 | 150564779 | 150565285 | -729 | 0.21 | 0.69 | -0.48 |
| *FLG2* | NM_001014342 | chr1 | 150599980 | 150600482 | -1125 | 0.09 | 0.71 | -0.62 |
| *FLJ25758* | NR_024372 | chr19 | 6958291 | 6958773 | -1106 | 0.19 | 0.53 | -0.34 |
| *FLJ33360* | NM_001001702 | chr5 | 6392643 | 6392825 | -2329 | 0.33 | 0.65 | -0.32 |
| *FLJ36000* | NR_027084 | chr17 | 21826704 | 21827210 | -1231 | 0.27 | 0.59 | -0.32 |
| *FLJ37543* | NM_173667 | chr5 | 60968055 | 60968553 | -1088 | 0.25 | 0.63 | -0.38 |
| *FLJ41562* | NM_001129907 | chr4 | 68767363 | 68767843 | -1210 | 0.12 | 0.41 | -0.30 |
| *FLJ43080* | NM_001039763 | chr5 | 110090175 | 110090693 | -154 | 0.45 | 0.86 | -0.40 |
| *FLJ43859* | NM_001145197 | chr9 | 83731700 | 83732187 | -1218 | 0.36 | 0.79 | -0.43 |
| *FLJ43860* | NM_207414 | chr8 | 142588424 | 142588868 | -2134 | 0.45 | 0.79 | -0.34 |
| *FLJ43950* | NR_026851 | chr9 | 83716277 | 83716795 | -1635 | 0.21 | 0.62 | -0.41 |
| *FLJ44082* | NM_207416 | chr9 | 83746772 | 83747259 | -1218 | 0.32 | 0.81 | -0.49 |
| *FLJ45079* | NM_001001685 | chr17 | 73394141 | 73394241 | -2427 | 0.40 | 0.79 | -0.39 |
| *FMO6P* | NR_002601 | chr1 | 169372642 | 169373128 | -617 | 0.15 | 0.53 | -0.38 |
| *FMO9P* | NR_002925 | chr1 | 164838968 | 164839390 | -597 | 0.30 | 0.71 | -0.41 |
| *FNTA* | NM_001018676 | chr8 | 43028717 | 43029208 | -1635 | 0.08 | 0.53 | -0.45 |
| *FNTB* | NM_002028 | chr14 | 64521376 | 64521895 | -1623 | 0.20 | 0.57 | -0.37 |
| *FOLH1* | NM_001014986 | chr11 | 49189121 | 49189209 | -2367 | 0.31 | 0.88 | -0.57 |
| *FPR1* | NM_002029 | chr19 | 56946833 | 56947305 | -107 | 0.28 | 0.73 | -0.45 |
| *FPR3* | NM_002030 | chr19 | 56989032 | 56989560 | -926 | 0.35 | 0.86 | -0.51 |
| *FRG2* | NM_001005217 | chr4 | 191185245 | 191185760 | -96 | 0.11 | 0.55 | -0.45 |
| *FRG2B* | NM_001080998 | chr10 | 135290128 | 135290643 | -96 | 0.13 | 0.52 | -0.39 |
| *FRMD3* | NM_174938 | chr9 | 85344640 | 85345144 | -1724 | 0.36 | 0.78 | -0.42 |
| *FRMD4B* | NM_015123 | chr3 | 69520483 | 69520586 | -2414 | 0.13 | 0.48 | -0.35 |
| *FYN* | NM_153048 | chr6 | 112188322 | 112188842 | -1604 | 0.31 | 0.67 | -0.36 |
| *GABRB1* | NM_000812 | chr4 | 46727161 | 46727640 | -650 | 0.28 | 0.65 | -0.37 |
| *GALC* | NM_000153 | chr14 | 87531612 | 87532108 | -2200 | 0.05 | 0.36 | -0.30 |
| *GAPT* | NM_152687 | chr5 | 57821604 | 57822119 | -1224 | 0.17 | 0.51 | -0.34 |
| *GAS2L3* | NM_174942 | chr12 | 99489745 | 99490239 | -1627 | 0.60 | 0.93 | -0.33 |
| *GBP7* | NM_207398 | chr1 | 89414259 | 89414772 | -204 | 0.13 | 0.45 | -0.32 |
| *GCG* | NM_002054 | chr2 | 162718902 | 162719417 | -2156 | 0.28 | 0.66 | -0.37 |
| *GCNT2* | NM_145649 | chr6 | 10628961 | 10629454 | -345 | 0.17 | 0.50 | -0.33 |
| *GDAP1* | NM_001040875 | chr8 | 75423217 | 75423690 | -1753 | 0.04 | 0.37 | -0.34 |
| *GDF11* | NM_005811 | chr12 | 54420911 | 54421451 | -2149 | 0.27 | 0.60 | -0.32 |
| *GDPD4* | NM_182833 | chr11 | 76676058 | 76676552 | -194 | 0.42 | 0.90 | -0.48 |
| *GIMAP5* | NM_018384 | chr7 | 150064422 | 150064894 | -725 | 0.30 | 0.82 | -0.51 |
| *GIMAP7* | NM_153236 | chr7 | 149842520 | 149843012 | -111 | 0.15 | 0.50 | -0.35 |
| *GINS4* | NM_032336 | chr8 | 41503986 | 41504466 | -1655 | 0.28 | 0.67 | -0.38 |
| *GJA1* | NM_000165 | chr6 | 121797426 | 121797930 | -765 | 0.06 | 0.56 | -0.50 |
| *GJA5* | NM_181703 | chr1 | 145699277 | 145699794 | -197 | 0.34 | 0.64 | -0.30 |
| *GJC3* | NM_181538 | chr7 | 99365643 | 99366120 | -702 | 0.24 | 0.87 | -0.63 |
| *GLDN* | NM_181789 | chr15 | 49418532 | 49419027 | -2224 | 0.21 | 0.57 | -0.36 |
| *GLIPR1* | NM_006851 | chr12 | 74160457 | 74160963 | -69 | 0.40 | 0.73 | -0.34 |
| *GLRA2* | NM_001118885 | chrX | 14454905 | 14455358 | -2208 | 0.37 | 0.74 | -0.37 |
| *GLT8D2* | NM_031302 | chr12 | 102970204 | 102970484 | -2299 | 0.16 | 0.65 | -0.50 |
| *GLYATL1* | NM_080661 | chr11 | 58466653 | 58467165 | -388 | 0.54 | 0.91 | -0.37 |
| *GNG11* | NM_004126 | chr7 | 93388531 | 93389011 | -180 | 0.31 | 0.64 | -0.33 |
| *GNPDA1* | NM_005471 | chr5 | 141374843 | 141375219 | -2227 | 0.25 | 0.63 | -0.38 |
| *GPC5* | NM_004466 | chr13 | 90847064 | 90847579 | -1613 | 0.26 | 0.83 | -0.57 |
| *GPHB5* | NM_145171 | chr14 | 62856268 | 62856778 | -2207 | 0.13 | 0.50 | -0.38 |
| *GPM6B* | NM_001001995 | chrX | 13747124 | 13747634 | -2144 | 0.40 | 0.72 | -0.32 |
| *GPR115* | NM_153838 | chr6 | 47772403 | 47772888 | -1601 | 0.00 | 0.34 | -0.33 |
| *GPR116* | NM_015234 | chr6 | 47032562 | 47033084 | -2189 | 0.58 | 0.89 | -0.31 |
| *GPR128* | NM_032787 | chr3 | 101810747 | 101811275 | -123 | 0.35 | 0.71 | -0.37 |
| *GPR15* | NM_005290 | chr3 | 99732567 | 99733082 | -742 | 0.18 | 0.49 | -0.30 |
| *GPR85* | NM_001146266 | chr7 | 112514087 | 112514579 | -704 | 0.24 | 0.59 | -0.35 |
| *GPR89C* | NM_001097616 | chr1 | 145891830 | 145892318 | -116 | 0.12 | 0.65 | -0.54 |
| *GPX3* | NM_002084 | chr5 | 150378704 | 150379224 | -1227 | 0.15 | 0.49 | -0.34 |
| *GPX5* | NM_001509 | chr6 | 28601438 | 28601936 | -80 | 0.09 | 0.42 | -0.33 |
| *GPX8* | NM_001008397 | chr5 | 54490754 | 54491242 | -742 | 0.28 | 0.59 | -0.31 |
| *GRIK2* | NM_021956 | chr6 | 101951754 | 101952283 | -1606 | 0.14 | 0.49 | -0.35 |
| *GRM3* | NM_000840 | chr7 | 86109426 | 86109974 | -1465 | 0.51 | 0.81 | -0.30 |
| *GRM8* | NM_000845 | chr7 | 126671662 | 126672157 | -1104 | 0.37 | 0.73 | -0.36 |
| *GRPEL2* | NM_152407 | chr5 | 148702754 | 148703314 | -2135 | 0.29 | 0.66 | -0.37 |
| *GSTA3* | NM_000847 | chr6 | 52882426 | 52882902 | -209 | 0.24 | 0.61 | -0.37 |
| *GUSBL1* | NR_003504 | chr6 | 27032999 | 27033336 | -855 | 0.30 | 0.70 | -0.40 |
| *GYPA* | NM_002099 | chr4 | 145282210 | 145282724 | -1113 | 0.09 | 0.39 | -0.31 |
| *GYPB* | NM_002100 | chr4 | 145161298 | 145161817 | -1611 | 0.06 | 0.36 | -0.30 |
| *GYPE* | NM_002102 | chr4 | 145048021 | 145048531 | -2110 | 0.07 | 0.37 | -0.30 |
| *GZMA* | NM_006144 | chr5 | 54432890 | 54433438 | -1066 | 0.31 | 0.66 | -0.35 |
| *H1FNT* | NM_181788 | chr12 | 47006612 | 47007159 | -2143 | 0.28 | 0.59 | -0.30 |
| *HAO1* | NM_017545 | chr20 | 7868980 | 7869460 | -127 | 0.14 | 0.52 | -0.38 |
| *HAS1* | NM_001523 | chr19 | 56920671 | 56921123 | -1864 | 0.39 | 0.69 | -0.30 |
| *HBBP1* | NR_001589 | chr11 | 5223022 | 5223524 | -1875 | 0.08 | 0.64 | -0.57 |
| *HBEGF* | NM_001945 | chr5 | 139708689 | 139708803 | -2374 | 0.30 | 0.60 | -0.31 |
| *HBG1* | NM_000559 | chr11 | 5229600 | 5230095 | -2184 | 0.22 | 0.54 | -0.31 |
| *HCRTR2* | NM_001526 | chr6 | 55144605 | 55145141 | -2156 | 0.28 | 0.58 | -0.30 |
| *HDAC8* | NM_018486 | chrX | 71711502 | 71711777 | -2261 | 0.21 | 0.61 | -0.40 |
| *HDAC9* | NM_014707 | chr7 | 18501422 | 18501902 | -231 | 0.10 | 0.65 | -0.55 |
| *HDGFL1* | NM_138574 | chr6 | 22675772 | 22676288 | -1626 | 0.25 | 0.67 | -0.42 |
| *HEMGN* | NM_018437 | chr9 | 99746812 | 99747315 | -108 | 0.44 | 0.77 | -0.33 |
| *HEPACAM* | NM_152722 | chr11 | 124311481 | 124311969 | -207 | 0.09 | 0.46 | -0.37 |
| *HEPACAM2* | NM_198151 | chr7 | 92686649 | 92687150 | -93 | 0.08 | 0.42 | -0.34 |
| *HFM1* | NM_001017975 | chr1 | 91643425 | 91643948 | -672 | 0.12 | 0.47 | -0.35 |
| *HGF* | NM_000601 | chr7 | 81238846 | 81239337 | -1703 | 0.26 | 0.67 | -0.41 |
| *HHLA1* | NM_001145095 | chr8 | 133186636 | 133187168 | -208 | 0.32 | 0.79 | -0.47 |
| *HIBADH* | NM_152740 | chr7 | 27670633 | 27671123 | -1751 | 0.31 | 0.67 | -0.35 |
| *HIF1AN* | NM_017902 | chr10 | 102284994 | 102285481 | -392 | 0.17 | 0.49 | -0.32 |
| *HIPK1* | NM_198269 | chr1 | 114293358 | 114293661 | -1779 | 0.62 | 0.92 | -0.30 |
| *HIST1H1B* | NM_005322 | chr6 | 27945212 | 27945679 | -2107 | 0.06 | 0.37 | -0.31 |
| *HIST1H4F* | NM_003540 | chr6 | 26347179 | 26347580 | -1252 | 0.24 | 0.64 | -0.40 |
| *HIST2H2BF* | NM_001024599 | chr1 | 148051963 | 148052451 | -1672 | 0.15 | 0.47 | -0.33 |
| *HK1* | NM_033497 | chr10 | 70697345 | 70697888 | -2144 | 0.10 | 0.46 | -0.36 |
| *HLA-DPB2* | NR_001435 | chr6 | 33187836 | 33188305 | -199 | 0.11 | 0.44 | -0.32 |
| *HLA-DQA1* | NM_002122 | chr6 | 32713019 | 32713548 | 123 | 0.37 | 0.69 | -0.31 |
| *HMGCS2* | NM_005518 | chr1 | 120113987 | 120114506 | -1205 | 0.24 | 0.64 | -0.40 |
| *HMHB1* | NM_021182 | chr5 | 143170639 | 143171123 | -1037 | 0.42 | 0.79 | -0.37 |
| *HMP19* | NM_015980 | chr5 | 173404223 | 173404735 | -850 | 0.14 | 0.44 | -0.30 |
| *HNRNPA3P1* | NR_002726 | chr10 | 43605943 | 43606446 | -323 | 0.32 | 0.75 | -0.43 |
| *HOXA3* | NM_030661 | chr7 | 27127463 | 27127946 | -1965 | 0.12 | 0.59 | -0.47 |
| *HOXB1* | NM_002144 | chr17 | 43964135 | 43964610 | -1101 | 0.40 | 0.75 | -0.35 |
| *HPVC1* | NR_004422 | chr7 | 54237623 | 54238168 | -287 | 0.42 | 0.75 | -0.33 |
| *HRC* | NM_002152 | chr19 | 54351814 | 54352362 | -1595 | 0.17 | 0.60 | -0.43 |
| *HRNR* | NM_001009931 | chr1 | 150463245 | 150463764 | -211 | 0.04 | 0.58 | -0.54 |
| *HSD11B1* | NM_181755 | chr1 | 207925303 | 207925806 | -617 | 0.47 | 0.91 | -0.44 |
| *HTR3E* | NM_182589 | chr3 | 185298236 | 185298707 | -2188 | 0.22 | 0.52 | -0.30 |
| *HYDIN* | NM_017558 | chr16 | 69823026 | 69823533 | -1209 | 0.10 | 0.56 | -0.47 |
| *IDI2* | NM_033261 | chr10 | 1063970 | 1064075 | -2223 | 0.51 | 0.85 | -0.34 |
| *IFI16* | NM_005531 | chr1 | 157244937 | 157245417 | -1128 | 0.11 | 0.60 | -0.49 |
| *IFNA10* | NM_002171 | chr9 | 21197048 | 21197560 | -162 | 0.22 | 0.66 | -0.44 |
| *IFNA21* | NM_002175 | chr9 | 21158102 | 21158574 | -1679 | 0.27 | 0.65 | -0.39 |
| *IFNA5* | NM_002169 | chr9 | 21295108 | 21295619 | -108 | 0.37 | 0.76 | -0.39 |
| *IFNA8* | NM_002170 | chr9 | 21398743 | 21399237 | -155 | 0.54 | 0.84 | -0.30 |
| *IFNB1* | NM_002176 | chr9 | 21068797 | 21069296 | -1103 | 0.35 | 0.68 | -0.33 |
| *IGF1* | NM_001111284 | chr12 | 101396942 | 101397431 | -627 | 0.22 | 0.53 | -0.31 |
| *IGFBP1* | NM_000596 | chr7 | 45892871 | 45893359 | -1368 | 0.34 | 0.76 | -0.43 |
| *IGFL2* | NM_001135113 | chr19 | 51342935 | 51343437 | -153 | 0.28 | 0.60 | -0.32 |
| *IGFL3* | NM_207393 | chr19 | 51319710 | 51320227 | -197 | 0.46 | 0.76 | -0.30 |
| *IGJ* | NM_144646 | chr4 | 71751104 | 71751578 | -129 | 0.06 | 0.52 | -0.45 |
| *IGSF6* | NM_005849 | chr16 | 21572824 | 21573311 | -1594 | 0.44 | 0.75 | -0.31 |
| *IL12A* | NM_000882 | chr3 | 161186901 | 161187389 | -2177 | 0.26 | 0.56 | -0.30 |
| *IL1F10* | NM_173161 | chr2 | 113539578 | 113540041 | -2207 | 0.19 | 0.49 | -0.30 |
| *IL1F9* | NM_019618 | chr2 | 113450218 | 113450698 | -1618 | 0.48 | 0.84 | -0.36 |
| *IL1RL1* | NM_003856 | chr2 | 102319272 | 102319758 | -633 | 0.23 | 0.59 | -0.36 |
| *IL21* | NM_021803 | chr4 | 123763029 | 123763522 | -1614 | 0.45 | 0.90 | -0.44 |
| *IL22RA2* | NM_052962 | chr6 | 137537700 | 137538208 | -1476 | 0.17 | 0.60 | -0.43 |
| *IL26* | NM_018402 | chr12 | 66906820 | 66907311 | -1227 | 0.41 | 0.72 | -0.30 |
| *IL2RG* | NM_000206 | chrX | 70248569 | 70249069 | -691 | 0.45 | 0.80 | -0.35 |
| *IL7R* | NM_002185 | chr5 | 35890859 | 35891414 | -1610 | 0.57 | 0.91 | -0.34 |
| *IL8* | NM_000584 | chr4 | 74822707 | 74823256 | -2156 | 0.11 | 0.47 | -0.36 |
| *INE2* | NR_002725 | chrX | 15716481 | 15716980 | -1097 | 0.17 | 0.68 | -0.52 |
| *IQCC* | NM_018134 | chr1 | 32443692 | 32444212 | 94 | 0.22 | 0.54 | -0.32 |
| *IQCF2* | NM_203424 | chr3 | 51869680 | 51870173 | -757 | 0.49 | 0.84 | -0.35 |
| *ITGA10* | NM_003637 | chr1 | 144234873 | 144235356 | -1231 | 0.28 | 0.61 | -0.33 |
| *ITLN1* | NM_017625 | chr1 | 159123411 | 159123830 | -2036 | 0.49 | 0.86 | -0.37 |
| *ITM2A* | NM_004867 | chrX | 78511672 | 78512189 | -2227 | 0.06 | 0.90 | -0.84 |
| *KCNAB1* | NM_172160 | chr3 | 157319671 | 157320152 | -1118 | 0.36 | 0.81 | -0.45 |
| *KCNE4* | NM_080671 | chr2 | 223623165 | 223623689 | -1678 | 0.48 | 0.81 | -0.33 |
| *KCNH1* | NM_002238 | chr1 | 209376274 | 209376567 | -2340 | 0.13 | 0.43 | -0.30 |
| *KCNIP1* | NM_001034837 | chr5 | 169862278 | 169862663 | -1154 | 0.22 | 0.57 | -0.35 |
| *KCNIP4* | NM_147183 | chr4 | 20916114 | 20916596 | -1728 | 0.55 | 0.91 | -0.36 |
| *KCNJ1* | NM_000220 | chr11 | 128218432 | 128218950 | -1118 | 0.17 | 0.47 | -0.30 |
| *KCNJ16* | NM_018658 | chr17 | 65580583 | 65581142 | -2157 | 0.40 | 0.72 | -0.32 |
| *KCNJ6* | NM_002240 | chr21 | 38211397 | 38211895 | -1080 | 0.13 | 0.49 | -0.36 |
| *KCNJ9* | NM_004983 | chr1 | 158317098 | 158317626 | -621 | 0.34 | 0.68 | -0.33 |
| *KCNK10* | NM_021161 | chr14 | 87862949 | 87863473 | -202 | 0.18 | 0.56 | -0.38 |
| *KCNK2* | NM_001017424 | chr1 | 213244625 | 213245097 | -646 | 0.28 | 0.78 | -0.50 |
| *KCNMB1* | NM_004137 | chr5 | 169749556 | 169749769 | -446 | 0.23 | 0.53 | -0.30 |
| *KCNMB3* | NM_171828 | chr3 | 180468734 | 180469245 | -1457 | 0.13 | 0.57 | -0.44 |
| *KCTD1* | NM_198991 | chr18 | 22476577 | 22476786 | -2375 | 0.48 | 0.78 | -0.30 |
| *KERA* | NM_007035 | chr12 | 89976207 | 89976730 | -206 | 0.14 | 0.55 | -0.41 |
| *KHDRBS2* | NM_152688 | chr6 | 63056007 | 63056517 | -2203 | 0.23 | 0.58 | -0.35 |
| *KIAA0748* | NM_001098815 | chr12 | 53662285 | 53662811 | -659 | 0.32 | 0.63 | -0.31 |
| *KIAA1210* | NM_020721 | chrX | 118170422 | 118170918 | -2100 | 0.32 | 0.77 | -0.45 |
| *KIAA1305* | NM_025081 | chr14 | 23935401 | 23935848 | -2206 | 0.16 | 0.46 | -0.31 |
| *KIAA1486* | NM_020864 | chr2 | 225973489 | 225973983 | -109 | 0.18 | 0.73 | -0.55 |
| *KIAA1688* | NM_025251 | chr8 | 145809647 | 145810145 | -200 | 0.36 | 0.73 | -0.37 |
| *KIF2B* | NM_032559 | chr17 | 49254891 | 49255391 | -96 | 0.46 | 0.85 | -0.39 |
| *KIF4B* | NM_001099293 | chr5 | 154371085 | 154371615 | -2102 | 0.43 | 0.85 | -0.43 |
| *KIFAP3* | NM_014970 | chr1 | 168310852 | 168311369 | -607 | 0.14 | 0.44 | -0.30 |
| *KIR2DL2* | NM_014219 | chr19_random | 163633 | 163989 | -788 | 0.32 | 0.80 | -0.48 |
| *KIR2DL3* | NM_014511 | chr19 | 59940357 | 59940831 | -1191 | 0.48 | 0.79 | -0.30 |
| *KIR2DL4* | NM_001080770 | chr19 | 60006506 | 60007011 | -119 | 0.47 | 0.79 | -0.31 |
| *KIR2DL5A* | NM_020535 | chr19_random | 228075 | 228424 | -1129 | 0.34 | 0.64 | -0.30 |
| *KIR2DL5B* | NM_001018081 | chr19_random | 228075 | 228424 | -1184 | 0.34 | 0.64 | -0.30 |
| *KIR3DL3* | NM_153443 | chr19 | 59926362 | 59926848 | -1190 | 0.40 | 0.79 | -0.39 |
| *KIR3DP1* | NM_001015070 | chr19 | 59926362 | 59926848 | -1207 | 0.40 | 0.79 | -0.39 |
| *KIR3DS1* | NM_001083539 | chr19_random | 210896 | 211394 | -1640 | 0.40 | 0.72 | -0.32 |
| *KLF17* | NM_173484 | chr1 | 44354668 | 44355122 | -2213 | 0.47 | 0.80 | -0.33 |
| *KLHDC9* | NM_001007255 | chr1 | 159332361 | 159332908 | -2142 | 0.30 | 0.62 | -0.31 |
| *KLHL14* | NM_020805 | chr18 | 28606637 | 28607146 | 80 | 0.43 | 0.74 | -0.31 |
| *KLHL3* | NM_017415 | chr5 | 137100027 | 137100518 | -594 | 0.11 | 0.57 | -0.46 |
| *KLHL31* | NM_001003760 | chr6 | 53639200 | 53639703 | -986 | 0.52 | 0.83 | -0.31 |
| *KLHL5* | NM_015990 | chr4 | 38738023 | 38738582 | -2151 | 0.08 | 0.47 | -0.39 |
| *KLK7* | NM_005046 | chr19 | 56180222 | 56180518 | -1408 | 0.44 | 0.85 | -0.41 |
| *KLKP1* | NR_002948 | chr19 | 56092774 | 56093289 | -1565 | 0.11 | 0.46 | -0.34 |
| *KLRC2* | NM_002260 | chr12 | 10481209 | 10481719 | -1605 | 0.31 | 0.75 | -0.44 |
| *KLRG1* | NM_005810 | chr12 | 9033426 | 9033903 | 177 | 0.42 | 0.82 | -0.39 |
| *KRT1* | NM_006121 | chr12 | 51361313 | 51361809 | -1103 | 0.19 | 0.56 | -0.37 |
| *KRT13* | NM_002274 | chr17 | 36915331 | 36915857 | -203 | 0.26 | 0.57 | -0.31 |
| *KRT2* | NM_000423 | chr12 | 51333115 | 51333596 | -1129 | 0.07 | 0.69 | -0.62 |
| *KRT26* | NM_181539 | chr17 | 36182300 | 36182782 | -604 | 0.21 | 0.63 | -0.43 |
| *KRT3* | NM_057088 | chr12 | 51478034 | 51478403 | -2059 | 0.20 | 0.77 | -0.56 |
| *KRT31* | NM_002277 | chr17 | 36807212 | 36807720 | -96 | 0.50 | 0.83 | -0.32 |
| *KRT33A* | NM_004138 | chr17 | 36762956 | 36763046 | -2419 | 0.53 | 0.85 | -0.33 |
| *KRT34* | NM_021013 | chr17 | 36793227 | 36793705 | -1304 | 0.31 | 0.90 | -0.59 |
| *KRT37* | NM_003770 | chr17 | 36835196 | 36835619 | -1059 | 0.12 | 0.43 | -0.30 |
| *KRT38* | NM_006771 | chr17 | 36851065 | 36851574 | -197 | 0.11 | 0.47 | -0.36 |
| *KRT4* | NM_002272 | chr12 | 51494958 | 51495446 | -600 | 0.55 | 0.87 | -0.32 |
| *KRT6A* | NM_005554 | chr12 | 51174412 | 51174912 | -1214 | 0.45 | 0.80 | -0.35 |
| *KRT6B* | NM_005555 | chr12 | 51134320 | 51134640 | -2303 | 0.31 | 0.89 | -0.58 |
| *KRT72* | NM_001146225 | chr12 | 51282923 | 51283432 | -1588 | 0.45 | 0.75 | -0.30 |
| *KRT75* | NM_004693 | chr12 | 51114334 | 51114818 | -199 | 0.43 | 0.75 | -0.32 |
| *KRT80* | NM_001081492 | chr12 | 50872415 | 50872922 | -617 | 0.32 | 0.63 | -0.31 |
| *KRT84* | NM_033045 | chr12 | 51066617 | 51067120 | -1184 | 0.52 | 0.89 | -0.37 |
| *KRTAP1-1* | NM_030967 | chr17 | 36452156 | 36452684 | -1181 | 0.16 | 0.47 | -0.31 |
| *KRTAP12-3* | NM_198697 | chr21 | 44901890 | 44902374 | -144 | 0.49 | 0.88 | -0.39 |
| *KRTAP12-4* | NM_198698 | chr21 | 44901890 | 44902374 | -3128 | 0.49 | 0.88 | -0.39 |
| *KRTAP1-3* | NM_030966 | chr17 | 36446817 | 36447129 | -2340 | 0.10 | 0.51 | -0.41 |
| *KRTAP13-2* | NM_181621 | chr21 | 30666291 | 30666816 | -125 | 0.15 | 0.71 | -0.56 |
| *KRTAP19-4* | NM_181610 | chr21 | 30791158 | 30791716 | -138 | 0.52 | 0.84 | -0.32 |
| *KRTAP19-6* | NM_181612 | chr21 | 30836004 | 30836502 | -201 | 0.22 | 0.54 | -0.32 |
| *KRTAP19-7* | NM_181614 | chr21 | 30855852 | 30856353 | -623 | 0.30 | 0.65 | -0.34 |
| *KRTAP20-3* | NM_001128077 | chr21 | 30935412 | 30935946 | -1374 | 0.15 | 0.51 | -0.36 |
| *KRTAP2-1* | NM_001123387 | chr17 | 36456674 | 36457154 | 180 | 0.18 | 0.53 | -0.35 |
| *KRTAP2-2* | NM_033032 | chr17_random | 297141 | 297696 | -591 | 0.09 | 0.61 | -0.52 |
| *KRTAP2-4* | NM_033184 | chr17_random | 286207 | 286754 | -861 | 0.28 | 0.62 | -0.34 |
| *KRTAP24-1* | NM_001085455 | chr21 | 30578493 | 30579007 | -1603 | 0.14 | 0.66 | -0.51 |
| *KRTAP3-2* | NM_031959 | chr17_random | 352119 | 352604 | -1076 | 0.40 | 0.84 | -0.44 |
| *KRTAP3-3* | NM_033185 | chr17 | 36403866 | 36404353 | -198 | 0.18 | 0.47 | -0.30 |
| *KRTAP4-11* | NM_033059 | chr17 | 36530067 | 36530550 | -2176 | 0.08 | 0.74 | -0.66 |
| *KRTAP4-8* | NM_031960 | chr17 | 36509551 | 36510073 | -1950 | 0.05 | 0.39 | -0.34 |
| *KRTAP8-1* | NM_175857 | chr21 | 31107383 | 31107908 | -204 | 0.26 | 0.77 | -0.51 |
| *KRTAP9-3* | NM_031962 | chr17 | 36640331 | 36640851 | -1649 | 0.44 | 0.76 | -0.32 |
| *KRTAP9-4* | NM_033191 | chr17 | 36657573 | 36658062 | -1646 | 0.33 | 0.66 | -0.33 |
| *KRTAP9-8* | NM_031963 | chr17 | 36646912 | 36647380 | -649 | 0.59 | 0.93 | -0.34 |
| *KRTAP9-9* | NM_030975 | chr17 | 36640331 | 36640851 | -1686 | 0.44 | 0.76 | -0.32 |
| *L3MBTL2* | NM_031488 | chr22 | 39929114 | 39929351 | -2025 | 0.47 | 0.84 | -0.37 |
| *LALBA* | NM_002289 | chr12 | 47251845 | 47252346 | -1999 | 0.19 | 0.64 | -0.45 |
| *LAMA2* | NM_000426 | chr6 | 129244545 | 129245026 | -1192 | 0.32 | 0.63 | -0.31 |
| *LANCL3* | NM_198511 | chrX | 37314255 | 37314772 | -1226 | 0.03 | 0.33 | -0.30 |
| *LARP1* | NM_015315 | chr5 | 154071321 | 154071842 | -1072 | 0.40 | 0.72 | -0.32 |
| *LAX1* | NM_001136190 | chr1 | 202000465 | 202000985 | -181 | 0.21 | 0.51 | -0.30 |
| *LCE1A* | NM_178348 | chr1 | 151065331 | 151065869 | -972 | 0.12 | 0.75 | -0.63 |
| *LCE1E* | NM_178353 | chr1 | 151024458 | 151024989 | -652 | 0.32 | 0.62 | -0.30 |
| *LCE1F* | NM_178354 | chr1 | 151014550 | 151015038 | -677 | 0.07 | 0.57 | -0.49 |
| *LCE2A* | NM_178428 | chr1 | 150935576 | 150936109 | -1620 | 0.04 | 0.35 | -0.31 |
| *LCE2B* | NM_014357 | chr1 | 150924731 | 150925255 | -229 | 0.28 | 0.60 | -0.33 |
| *LCE2C* | NM_178429 | chr1 | 150913414 | 150913917 | -728 | 0.16 | 0.48 | -0.32 |
| *LCE5A* | NM_178438 | chr1 | 150749249 | 150749751 | -443 | 0.27 | 0.71 | -0.44 |
| *LCLAT1* | NM_001002257 | chr2 | 30522436 | 30522951 | -946 | 0.25 | 0.57 | -0.32 |
| *LCN1* | NM_002297 | chr9 | 137551795 | 137552324 | -1046 | 0.25 | 0.75 | -0.50 |
| *LDLR* | NM_000527 | chr19 | 11059705 | 11060191 | -1108 | 0.18 | 0.67 | -0.49 |
| *LELP1* | NM_001010857 | chr1 | 151440117 | 151440583 | -2192 | 0.51 | 0.81 | -0.30 |
| *LEMD1* | NM_001001552 | chr1 | 203658172 | 203658676 | -620 | 0.46 | 0.80 | -0.34 |
| *LGALS3* | NM_002306 | chr14 | 54663859 | 54664336 | -1526 | 0.19 | 0.50 | -0.31 |
| *LGALS9C* | NM_001040078 | chr17 | 18318839 | 18319162 | -1822 | 0.31 | 0.63 | -0.32 |
| *LHFPL1* | NM_178175 | chrX | 111811017 | 111811507 | -1231 | 0.13 | 0.50 | -0.37 |
| *LILRA5* | NM_021250 | chr19 | 59516141 | 59516528 | -113 | 0.46 | 0.85 | -0.40 |
| *LMO2* | NM_005574 | chr11 | 33872272 | 33872763 | -2105 | 0.54 | 0.88 | -0.35 |
| *LNX1* | NM_032622 | chr4 | 54121109 | 54121629 | -2176 | 0.16 | 0.57 | -0.40 |
| *LOC100101120* | NR_003592 | chrY | 10163718 | 10164215 | -1095 | 0.41 | 0.87 | -0.46 |
| *LOC100101266* | NR_003603 | chr19 | 24139943 | 24140457 | -2111 | 0.28 | 0.80 | -0.52 |
| *LOC100128675* | NR_024561 | chr19 | 40289395 | 40289884 | -624 | 0.22 | 0.55 | -0.33 |
| *LOC100129055* | NR_024524 | chr10 | 38502184 | 38502631 | -2196 | 0.10 | 0.53 | -0.43 |
| *LOC100129935* | NR_026870 | chr19 | 44818728 | 44819220 | -2190 | 0.20 | 0.51 | -0.32 |
| *LOC100131726* | NR_024479 | chr8 | 124284041 | 124284523 | -118 | 0.29 | 0.60 | -0.31 |
| *LOC100131897* | NM_001129891 | chr5 | 169342139 | 169342675 | -2085 | 0.17 | 0.48 | -0.31 |
| *LOC100192426* | NR_024419 | chr18 | 8358497 | 8358982 | -1707 | 0.17 | 0.60 | -0.43 |
| *LOC100216001* | NR_024475 | chr10 | 4710739 | 4711252 | -733 | 0.39 | 0.76 | -0.36 |
| *LOC100240734* | NR_026657 | chr12 | 52780575 | 52781015 | -1464 | 0.24 | 0.67 | -0.44 |
| *LOC126661* | NM_001102601 | chr1 | 45739688 | 45740187 | -1704 | 0.18 | 0.52 | -0.34 |
| *LOC144742* | NR_024246 | chr12 | 118227399 | 118227883 | -2073 | 0.10 | 0.45 | -0.35 |
| *LOC148824* | NR_027309 | chr1 | 245762593 | 245763087 | -2111 | 0.22 | 0.72 | -0.50 |
| *LOC152225* | NR_026934 | chr3 | 103139966 | 103140508 | -2155 | 0.31 | 0.89 | -0.57 |
| *LOC154822* | NR_024394 | chr7 | 158492401 | 158492903 | -1153 | 0.40 | 0.81 | -0.41 |
| *LOC157381* | NR_027321 | chr8 | 126021010 | 126021552 | -2149 | 0.35 | 0.68 | -0.33 |
| *LOC196415* | NM_001101339 | chr12 | 25042163 | 25042642 | -762 | 0.17 | 0.82 | -0.66 |
| *LOC220115* | NR_002793 | chr13 | 51959244 | 51959712 | -1650 | 0.50 | 0.83 | -0.33 |
| *LOC254312* | NR_015413 | chr10 | 11035012 | 11035507 | -1127 | 0.16 | 0.47 | -0.31 |
| *LOC257358* | NR_026945 | chr5 | 169690046 | 169690570 | -704 | 0.22 | 0.61 | -0.38 |
| *LOC26080* | NR_027278 | chr13_random | 22762 | 22864 | -1235 | 0.16 | 0.85 | -0.69 |
| *LOC284276* | NR_015417 | chr18 | 72367177 | 72367616 | -2202 | 0.42 | 0.75 | -0.33 |
| *LOC284379* | NR_002938 | chr19 | 58798131 | 58798622 | 186 | 0.44 | 0.80 | -0.36 |
| *LOC284661* | NR_027088 | chr1 | 4369532 | 4370001 | -2203 | 0.11 | 0.55 | -0.44 |
| *LOC284688* | NR_026957 | chr1 | 168519981 | 168520484 | -259 | 0.42 | 0.83 | -0.41 |
| *LOC285194* | NR_015391 | chr3 | 117910454 | 117910957 | -618 | 0.32 | 0.63 | -0.31 |
| *LOC285370* | NR_027102 | chr3 | 10782717 | 10783221 | -2092 | 0.14 | 0.57 | -0.43 |
| *LOC285401* | NR_027104 | chr3 | 63061515 | 63062040 | -1625 | 0.20 | 0.55 | -0.35 |
| *LOC285692* | NR_027112 | chr5 | 9958683 | 9959179 | -1995 | 0.05 | 0.38 | -0.33 |
| *LOC285768* | NR_027115 | chr6 | 1046432 | 1046907 | -102 | 0.54 | 0.91 | -0.36 |
| *LOC285780* | NR_026970 | chr6 | 6569002 | 6569507 | -1196 | 0.18 | 0.68 | -0.51 |
| *LOC29034* | NR_002763 | chr2 | 211190173 | 211190684 | -110 | 0.08 | 0.48 | -0.39 |
| *LOC339535* | NR_015407 | chr1 | 236716798 | 236717310 | -1114 | 0.51 | 0.91 | -0.40 |
| *LOC374491* | NR_002815 | chr13 | 24051894 | 24052439 | -178 | 0.52 | 0.83 | -0.31 |
| *LOC389493* | NM_001145712 | chr7 | 56152847 | 56153363 | -1521 | 0.48 | 0.84 | -0.36 |
| *LOC389634* | NR_024420 | chr12 | 8436645 | 8437036 | -2225 | 0.23 | 0.54 | -0.31 |
| *LOC400043* | NR_026656 | chr12 | 52804670 | 52805204 | -1184 | 0.11 | 0.46 | -0.35 |
| *LOC440354* | NR_002473 | chr16 | 29485517 | 29485983 | -709 | 0.54 | 0.87 | -0.32 |
| *LOC441294* | NM_001008747 | chr7 | 142977547 | 142978109 | -1183 | 0.16 | 0.46 | -0.30 |
| *LOC441601* | NR_003034 | chr11 | 50216034 | 50216472 | -2054 | 0.19 | 0.56 | -0.37 |
| *LOC550643* | NR_015367 | chrX | 56770558 | 56771058 | -1634 | 0.42 | 0.79 | -0.37 |
| *LOC642425* | NM_001146208 | chr11 | 89212433 | 89212895 | -2209 | 0.22 | 0.74 | -0.52 |
| *LOC642587* | NM_001104548 | chr1 | 207667960 | 207668493 | -563 | 0.54 | 0.86 | -0.32 |
| *LOC642929* | NR_027472 | chr9 | 43135269 | 43135668 | 11 | 0.50 | 0.85 | -0.34 |
| *LOC643854* | NM_001145659 | chr6 | 132074234 | 132074779 | -656 | 0.19 | 0.83 | -0.64 |
| *LOC643896* | NM_001101386 | chr2 | 100490985 | 100491475 | -940 | 0.16 | 0.51 | -0.34 |
| *LOC644145* | NR_003935 | chr4 | 56379511 | 56380052 | -1211 | 0.30 | 0.69 | -0.39 |
| *LOC644669* | NR_027417 | chr18 | 15316508 | 15317058 | -865 | 0.34 | 0.65 | -0.31 |
| *LOC646214* | NR_027053 | chr15 | 19195587 | 19196074 | -1714 | 0.45 | 0.76 | -0.30 |
| *LOC646405* | NR_026730 | chr13 | 24440963 | 24441506 | -627 | 0.36 | 0.72 | -0.36 |
| *LOC651503* | NM_001080842 | chr6 | 29339781 | 29340329 | -220 | 0.08 | 0.39 | -0.31 |
| *LOC654254* | NM_001137608 | chr4 | 280293 | 280784 | -594 | 0.42 | 0.73 | -0.31 |
| *LOC727797* | NM_001105536 | chr3_random | 663752 | 664070 | -1807 | 0.32 | 0.62 | -0.30 |
| *LOC727924* | NR_015416 | chr15 | 19777405 | 19777920 | -1732 | 0.06 | 0.57 | -0.51 |
| *LOC728989* | NR_024442 | chr1 | 144983163 | 144983538 | -2127 | 0.45 | 0.75 | -0.30 |
| *LOC729384* | NM_001105522 | chr11 | 89296520 | 89296989 | -124 | 0.45 | 0.76 | -0.31 |
| *LOC729668* | NR_003524 | chr10 | 30705057 | 30705556 | -1923 | 0.23 | 0.59 | -0.36 |
| *LOC730811* | NR_024468 | chr2 | 2301377 | 2301870 | -386 | 0.17 | 0.61 | -0.45 |
| *LOC84856* | NR_026827 | chr10 | 42289463 | 42289962 | -1231 | 0.34 | 0.72 | -0.39 |
| *LOC91431* | NM_001099776 | chr4 | 113729233 | 113729731 | -1226 | 0.27 | 0.67 | -0.40 |
| *LOXHD1* | NM_144612 | chr18 | 42435586 | 42436078 | -208 | 0.40 | 0.82 | -0.42 |
| *LPO* | NM_006151 | chr17 | 53670534 | 53671065 | -46 | 0.17 | 0.47 | -0.30 |
| *LRP1B* | NM_018557 | chr2 | 142606748 | 142607266 | -1267 | 0.29 | 0.73 | -0.44 |
| *LRRC69* | NM_001129890 | chr8 | 92181588 | 92182147 | -2154 | 0.10 | 0.45 | -0.35 |
| *LRRC7* | NM_020794 | chr1 | 69996026 | 69996557 | -2153 | 0.11 | 0.60 | -0.49 |
| *LSM11* | NM_173491 | chr5 | 157102402 | 157102921 | -670 | 0.29 | 0.59 | -0.30 |
| *LST-3TM12* | NM_001009562 | chr12 | 21057944 | 21058476 | -1686 | 0.18 | 0.53 | -0.36 |
| *LTA* | NM_000595 | chr6 | 31646551 | 31646738 | -1426 | 0.27 | 0.57 | -0.30 |
| *LY9* | NM_001033667 | chr1 | 159030579 | 159031070 | -1726 | 0.35 | 0.75 | -0.40 |
| *LYVE1* | NM_006691 | chr11 | 10547430 | 10547925 | -736 | 0.13 | 0.54 | -0.41 |
| *MAB21L1* | NM_005584 | chr13 | 34948544 | 34949045 | 37 | 0.30 | 0.78 | -0.48 |
| *MACC1* | NM_182762 | chr7 | 20223922 | 20224440 | -643 | 0.11 | 0.41 | -0.30 |
| *MACF1* | NM_033044 | chr1 | 39567914 | 39568419 | -1229 | 0.12 | 0.85 | -0.74 |
| *MAP2* | NM_001039538 | chr2 | 209994588 | 209995035 | -2203 | 0.11 | 0.57 | -0.46 |
| *MAPK10* | NM_138982 | chr4 | 87496696 | 87497183 | -2172 | 0.08 | 0.61 | -0.53 |
| *MARCO* | NM_006770 | chr2 | 119414731 | 119415232 | -1232 | 0.45 | 0.86 | -0.41 |
| *MBIP* | NM_001144891 | chr14 | 35861916 | 35862105 | -2377 | 0.05 | 0.38 | -0.33 |
| *MBNL2* | NM_144778 | chr13 | 96670145 | 96670596 | -2203 | 0.13 | 0.68 | -0.56 |
| *MBOAT4* | NM_001100916 | chr8 | 30124009 | 30124083 | -2304 | 0.23 | 0.65 | -0.42 |
| *MC3R* | NM_019888 | chr20 | 54256753 | 54257253 | -191 | 0.16 | 0.46 | -0.30 |
| *MC5R* | NM_005913 | chr18 | 13814900 | 13815398 | -615 | 0.44 | 0.75 | -0.30 |
| *MCFD2* | NM_139279 | chr2 | 46998674 | 46998885 | -2326 | 0.17 | 0.57 | -0.40 |
| *MCHR2* | NM_001040179 | chr6 | 100551224 | 100551312 | -2448 | 0.19 | 0.56 | -0.37 |
| *MGC34034* | NR_027029 | chr6 | 134182092 | 134182602 | -1630 | 0.43 | 0.80 | -0.37 |
| *MGC4473* | NR_024160 | chr1 | 167020367 | 167020845 | -2196 | 0.24 | 0.59 | -0.35 |
| *MGC52282* | NR_026864 | chr16 | 2833931 | 2834352 | -1388 | 0.49 | 0.86 | -0.36 |
| *MGP* | NM_000900 | chr12 | 14930958 | 14931448 | -1108 | 0.41 | 0.85 | -0.44 |
| *MIRHG2* | NR_001458 | chr21 | 25854971 | 25855450 | -1116 | 0.34 | 0.69 | -0.34 |
| *MMP20* | NM_004771 | chr11 | 102003160 | 102003642 | -2128 | 0.07 | 0.38 | -0.31 |
| *MMP3* | NM_002422 | chr11 | 102219491 | 102220007 | -197 | 0.36 | 0.66 | -0.30 |
| *MMP7* | NM_002423 | chr11 | 101907054 | 101907536 | -607 | 0.14 | 0.46 | -0.31 |
| *MOCS1* | NM_001075098 | chr6 | 40011572 | 40012095 | -1601 | 0.07 | 0.43 | -0.36 |
| *MOCS2* | NM_004531 | chr5 | 52441945 | 52442452 | -1116 | 0.27 | 0.63 | -0.36 |
| *MR1* | NM_001531 | chr1 | 179268446 | 179268948 | -1064 | 0.24 | 0.61 | -0.37 |
| *MRGPRX1* | NM_147199 | chr11 | 18914064 | 18914585 | -1199 | 0.45 | 0.91 | -0.46 |
| *MRPS18A* | NM_018135 | chr6 | 43765336 | 43765753 | -2038 | 0.21 | 0.52 | -0.31 |
| *MS4A13* | NM_001012417 | chr11 | 60038021 | 60038573 | -1164 | 0.23 | 0.55 | -0.32 |
| *MS4A3* | NM_001031666 | chr11 | 59580088 | 59580560 | -352 | 0.22 | 0.59 | -0.38 |
| *MS4A4A* | NM_024021 | chr11 | 59805623 | 59806159 | -770 | 0.14 | 0.46 | -0.32 |
| *MS4A7* | NM_021201 | chr11 | 59902199 | 59902717 | -75 | 0.12 | 0.48 | -0.37 |
| *MS4A8B* | NM_031457 | chr11 | 60223194 | 60223682 | -184 | 0.36 | 0.68 | -0.32 |
| *MT1B* | NM_005947 | chr16 | 55241870 | 55242377 | -1187 | 0.14 | 0.44 | -0.30 |
| *MTMR8* | NM_017677 | chrX | 63533932 | 63534452 | -2156 | 0.30 | 0.62 | -0.33 |
| *MUC4* | NM_004532 | chr3 | 197023506 | 197023990 | -203 | 0.13 | 0.62 | -0.49 |
| *MUCL1* | NM_058173 | chr12 | 53533075 | 53533604 | -1225 | 0.28 | 0.58 | -0.31 |
| *MYBPC1* | NM_002465 | chr12 | 100512769 | 100513292 | 153 | 0.36 | 0.72 | -0.36 |
| *MYH15* | NM_014981 | chr3 | 109731832 | 109732349 | -1231 | 0.37 | 0.89 | -0.51 |
| *MYH4* | NM_017533 | chr17 | 10313557 | 10314071 | -213 | 0.17 | 0.54 | -0.37 |
| *MYLK4* | NM_001012418 | chr6 | 2697517 | 2698052 | -1631 | 0.44 | 0.85 | -0.41 |
| *MYO1G* | NM_033054 | chr7 | 44987140 | 44987656 | -2205 | 0.27 | 0.57 | -0.30 |
| *MYT1* | NM_004535 | chr20 | 62264802 | 62265295 | -1221 | 0.49 | 0.79 | -0.30 |
| *NACAP1* | NR_002182 | chr8 | 102447857 | 102448406 | -2164 | 0.42 | 0.76 | -0.33 |
| *NAV3* | NM_014903 | chr12 | 76748738 | 76749210 | -225 | 0.15 | 0.56 | -0.41 |
| *NBN* | NM_002485 | chr8 | 91066432 | 91066732 | -507 | 0.30 | 0.62 | -0.32 |
| *NBPF22P* | NR_003719 | chr5 | 85612064 | 85612561 | -1704 | 0.22 | 0.88 | -0.66 |
| *NBPF7* | NM_001047980 | chr1 | 120190225 | 120190725 | -1173 | 0.16 | 0.64 | -0.48 |
| *NCRNA00158* | NR_024027 | chr21 | 25727747 | 25728182 | -2080 | 0.44 | 0.77 | -0.33 |
| *NDST4* | NM_022569 | chr4 | 116254335 | 116254832 | -102 | 0.43 | 0.80 | -0.37 |
| *NDUFS4* | NM_002495 | chr5 | 52890747 | 52891256 | -1219 | 0.24 | 0.61 | -0.37 |
| *NEBL* | NM_006393 | chr10 | 21226410 | 21226927 | -131 | 0.63 | 0.98 | -0.35 |
| *NECAB1* | NM_022351 | chr8 | 91871666 | 91872146 | -1190 | 0.33 | 0.73 | -0.40 |
| *NEUROD6* | NM_022728 | chr7 | 31346910 | 31347431 | -107 | 0.52 | 0.86 | -0.34 |
| *NIACR1* | NM_177551 | chr12 | 121753796 | 121754319 | -200 | 0.53 | 0.90 | -0.37 |
| *NIACR2* | NM_006018 | chr12 | 121767250 | 121767769 | -117 | 0.55 | 0.88 | -0.33 |
| *NIN* | NM_016350 | chr14 | 50361478 | 50362028 | -1647 | 0.15 | 0.51 | -0.36 |
| *NLRP12* | NM_144687 | chr19 | 59019438 | 59019658 | -88 | 0.27 | 0.60 | -0.33 |
| *NLRP13* | NM_176810 | chr19 | 61135672 | 61136115 | -379 | 0.42 | 0.76 | -0.34 |
| *NLRP3* | NM_001079821 | chr1 | 245645865 | 245646385 | 45 | 0.15 | 0.64 | -0.49 |
| *NME5* | NM_003551 | chr5 | 137503882 | 137504388 | -1104 | 0.47 | 0.88 | -0.40 |
| *NMUR2* | NM_020167 | chr5 | 151764889 | 151765385 | -104 | 0.22 | 0.61 | -0.40 |
| *NOV* | NM_002514 | chr8 | 120495298 | 120495847 | -2159 | 0.15 | 0.49 | -0.33 |
| *NOX4* | NM_001143837 | chr11 | 88964348 | 88964854 | -2174 | 0.24 | 0.66 | -0.42 |
| *NPBWR2* | NM_005286 | chr20 | 62209772 | 62210176 | -1346 | 0.25 | 0.65 | -0.40 |
| *NPFFR2* | NM_004885 | chr4 | 73115280 | 73115791 | -848 | 0.57 | 0.87 | -0.30 |
| *NPS* | NM_001030013 | chr10 | 129236018 | 129236487 | -1349 | 0.20 | 0.58 | -0.37 |
| *NPY6R* | NR_002713 | chr5 | 137170950 | 137171474 | -148 | 0.34 | 0.71 | -0.37 |
| *NRSN1* | NM_080723 | chr6 | 24233533 | 24234051 | -600 | 0.62 | 0.94 | -0.31 |
| *NUDT17* | NM_001012758 | chr1 | 144302399 | 144302865 | -1840 | 0.44 | 0.74 | -0.30 |
| *NXF2* | NM_022053 | chrX | 101387875 | 101388177 | -799 | 0.48 | 0.81 | -0.34 |
| *OC90* | NM_001080399 | chr8 | 133141209 | 133141748 | -669 | 0.16 | 0.61 | -0.45 |
| *ODAM* | NM_017855 | chr4 | 71095850 | 71096371 | -721 | 0.20 | 0.55 | -0.36 |
| *OPHN1* | NM_002547 | chrX | 67570863 | 67571379 | -1097 | 0.24 | 0.56 | -0.32 |
| *OPN5* | NM_001030051 | chr6 | 47862429 | 47862950 | -130 | 0.14 | 0.50 | -0.36 |
| *OPTC* | NM_014359 | chr1 | 201729046 | 201729529 | -605 | 0.38 | 0.78 | -0.40 |
| *OR10A2* | NM_001004460 | chr11 | 6846100 | 6846594 | -1214 | 0.19 | 0.51 | -0.33 |
| *OR10A3* | NM_001003745 | chr11 | 7918988 | 7919512 | -1607 | 0.15 | 0.48 | -0.33 |
| *OR10A6* | NM_001004461 | chr11 | 7906649 | 7907131 | -105 | 0.17 | 0.58 | -0.41 |
| *OR10A7* | NM_001005280 | chr12 | 53899121 | 53899600 | -1714 | 0.14 | 0.52 | -0.38 |
| *OR10AD1* | NM_001004134 | chr12 | 46883686 | 46884191 | -596 | 0.32 | 0.78 | -0.46 |
| *OR10G2* | NM_001005466 | chr14 | 21172799 | 21173312 | -217 | 0.43 | 0.94 | -0.51 |
| *OR10G8* | NM_001004464 | chr11 | 123403105 | 123403605 | -2184 | 0.35 | 0.66 | -0.31 |
| *OR10G9* | NM_001001953 | chr11 | 123396976 | 123397465 | -1708 | 0.22 | 0.56 | -0.34 |
| *OR10H1* | NM_013940 | chr19 | 15779763 | 15780187 | -39 | 0.39 | 0.81 | -0.41 |
| *OR10H2* | NM_013939 | chr19 | 15699367 | 15699845 | -227 | 0.38 | 0.81 | -0.43 |
| *OR10J5* | NM_001004469 | chr1 | 157772914 | 157773422 | -747 | 0.45 | 0.95 | -0.50 |
| *OR10P1* | NM_206899 | chr12 | 54314960 | 54315083 | -1920 | 0.49 | 0.82 | -0.33 |
| *OR10S1* | NM_001004474 | chr11 | 123354069 | 123354554 | -703 | 0.49 | 0.79 | -0.30 |
| *OR10T2* | NM_001004475 | chr1 | 156637843 | 156638332 | -2207 | 0.06 | 0.78 | -0.71 |
| *OR10X1* | NM_001004477 | chr1 | 156816163 | 156816657 | -97 | 0.08 | 0.43 | -0.34 |
| *OR11A1* | NM_013937 | chr6 | 29505688 | 29505894 | -2303 | 0.15 | 0.45 | -0.30 |
| *OR11H12* | NM_001013354 | chr14 | 18447234 | 18447726 | -113 | 0.07 | 0.39 | -0.32 |
| *OR11H6* | NM_001004480 | chr14 | 19759989 | 19760425 | -1501 | 0.12 | 0.49 | -0.38 |
| *OR11L1* | NM_001001959 | chr1 | 246072225 | 246072777 | -680 | 0.41 | 0.75 | -0.34 |
| *OR12D2* | NM_013936 | chr6 | 29470406 | 29470929 | -1726 | 0.00 | 0.53 | -0.53 |
| *OR13C4* | NM_001001919 | chr9 | 106329174 | 106329666 | -109 | 0.06 | 0.64 | -0.58 |
| *OR13C5* | NM_001004482 | chr9 | 106401912 | 106402397 | -639 | 0.15 | 0.54 | -0.39 |
| *OR13C9* | NM_001001956 | chr9 | 106422325 | 106422749 | -2231 | 0.08 | 0.55 | -0.47 |
| *OR13F1* | NM_001004485 | chr9 | 106304121 | 106304657 | -1975 | 0.58 | 0.93 | -0.35 |
| *OR14C36* | NM_001001918 | chr1 | 246576722 | 246577222 | -1727 | 0.17 | 0.49 | -0.32 |
| *OR14I1* | NM_001004734 | chr1 | 246912167 | 246912697 | -204 | 0.14 | 0.53 | -0.39 |
| *OR1J2* | NM_054107 | chr9 | 124311924 | 124312383 | -747 | 0.46 | 0.76 | -0.31 |
| *OR1N2* | NM_001004457 | chr9 | 124353367 | 124353848 | -1661 | 0.21 | 0.76 | -0.55 |
| *OR1Q1* | NM_012364 | chr9 | 124414397 | 124414870 | -2203 | 0.41 | 0.72 | -0.31 |
| *OR1S1* | NM_001004458 | chr11 | 57737414 | 57737914 | -1128 | 0.33 | 0.77 | -0.44 |
| *OR1S2* | NM_001004459 | chr11 | 57729196 | 57729669 | -1203 | 0.10 | 0.42 | -0.31 |
| *OR2A1* | NM_001005287 | chr7 | 143645542 | 143646021 | -368 | 0.29 | 0.69 | -0.40 |
| *OR2A12* | NM_001004135 | chr7 | 143422566 | 143423059 | -320 | 0.12 | 0.47 | -0.35 |
| *OR2A42* | NM_001001802 | chr7 | 143645542 | 143646021 | -386 | 0.29 | 0.69 | -0.40 |
| *OR2A7* | NM_001005328 | chr7 | 143589795 | 143590097 | -2292 | 0.20 | 0.57 | -0.36 |
| *OR2AT4* | NM_001005285 | chr11 | 74479757 | 74480282 | -1613 | 0.36 | 0.69 | -0.33 |
| *OR2B11* | NM_001004492 | chr1 | 245683168 | 245683675 | -1514 | 0.43 | 0.78 | -0.36 |
| *OR2C3* | NM_198074 | chr1 | 245764968 | 245765472 | -1456 | 0.45 | 0.76 | -0.31 |
| *OR2D2* | NM_003700 | chr11 | 6870253 | 6870783 | -211 | 0.25 | 0.66 | -0.41 |
| *OR2D3* | NM_001004684 | chr11 | 6897378 | 6897888 | -1175 | 0.31 | 0.74 | -0.43 |
| *OR2F1* | NM_012369 | chr7 | 143287071 | 143287575 | -629 | 0.26 | 0.68 | -0.42 |
| *OR2G3* | NM_001001914 | chr1 | 245834754 | 245835199 | -533 | 0.32 | 0.76 | -0.44 |
| *OR2H1* | NM_030883 | chr6 | 29533839 | 29534336 | -120 | 0.15 | 0.78 | -0.63 |
| *OR2L3* | NM_001004687 | chr1 | 246290170 | 246290684 | -179 | 0.25 | 0.84 | -0.59 |
| *OR2M2* | NM_001004688 | chr1 | 246408523 | 246409043 | -1127 | 0.46 | 0.84 | -0.38 |
| *OR2M3* | NM_001004689 | chr1 | 246431871 | 246432384 | -864 | 0.44 | 0.83 | -0.39 |
| *OR2M5* | NM_001004690 | chr1 | 246373082 | 246373559 | -1751 | 0.45 | 0.78 | -0.33 |
| *OR2M7* | NM_001004691 | chr1 | 246555858 | 246556353 | -1612 | 0.16 | 0.48 | -0.32 |
| *OR2S2* | NM_019897 | chr9 | 35948101 | 35948625 | -212 | 0.33 | 0.63 | -0.31 |
| *OR2T10* | NM_001004693 | chr1 | 246824638 | 246825146 | -1200 | 0.43 | 0.86 | -0.43 |
| *OR2T2* | NM_001004136 | chr1 | 246680283 | 246680832 | -2163 | 0.20 | 0.63 | -0.42 |
| *OR2T27* | NM_001001824 | chr1 | 246880671 | 246881159 | -107 | 0.25 | 0.63 | -0.37 |
| *OR2T3* | NM_001005495 | chr1 | 246701809 | 246702266 | -1236 | 0.31 | 0.66 | -0.35 |
| *OR2T34* | NM_001001821 | chr1 | 246807056 | 246807154 | -2424 | 0.33 | 0.87 | -0.54 |
| *OR2T35* | NM_001001827 | chr1 | 246870654 | 246871136 | -1713 | 0.16 | 0.47 | -0.31 |
| *OR2T6* | NM_001005471 | chr1 | 246616519 | 246617023 | -761 | 0.19 | 0.63 | -0.45 |
| *OR2V2* | NM_206880 | chr5 | 180513855 | 180514354 | -443 | 0.48 | 0.78 | -0.30 |
| *OR2W5* | NM_001004698 | chr1 | 245719564 | 245720061 | -1239 | 0.35 | 0.73 | -0.38 |
| *OR3A2* | NM_002551 | chr17 | 3130754 | 3131253 | -1985 | 0.37 | 0.70 | -0.33 |
| *OR3A3* | NM_012373 | chr17 | 3270235 | 3270722 | -132 | 0.29 | 0.60 | -0.31 |
| *OR4A15* | NM_001005275 | chr11 | 54891483 | 54891945 | -221 | 0.19 | 0.49 | -0.30 |
| *OR4A16* | NM_001005274 | chr11 | 54866857 | 54867346 | -150 | 0.13 | 0.69 | -0.57 |
| *OR4A47* | NM_001005512 | chr11 | 48466583 | 48467066 | -95 | 0.23 | 0.70 | -0.47 |
| *OR4C12* | NM_001005270 | chr11 | 49961470 | 49961958 | -1101 | 0.18 | 0.52 | -0.34 |
| *OR4C13* | NM_001001955 | chr11 | 49928196 | 49928650 | -2127 | 0.25 | 0.55 | -0.30 |
| *OR4D2* | NM_001004707 | chr17 | 53600052 | 53600502 | -1738 | 0.35 | 0.68 | -0.33 |
| *OR4D6* | NM_001004708 | chr11 | 58980113 | 58980619 | -643 | 0.20 | 0.53 | -0.33 |
| *OR4F17* | NM_001005240 | chr19 | 60746 | 61284 | -663 | 0.32 | 0.75 | -0.43 |
| *OR4F4* | NM_001004195 | chr15 | 100280744 | 100281233 | -203 | 0.07 | 0.38 | -0.31 |
| *OR4F5* | NM_001005484 | chr1 | 57972 | 58465 | -734 | 0.26 | 0.87 | -0.62 |
| *OR4F6* | NM_001005326 | chr15 | 100163060 | 100163582 | -124 | 0.58 | 0.92 | -0.34 |
| *OR4K15* | NM_001005486 | chr14 | 19511082 | 19511562 | -2195 | 0.36 | 0.74 | -0.38 |
| *OR4K2* | NM_001005501 | chr14 | 19412882 | 19413380 | -1135 | 0.08 | 0.52 | -0.45 |
| *OR4L1* | NM_001004717 | chr14 | 19596138 | 19596677 | -1635 | 0.18 | 0.53 | -0.35 |
| *OR4N4* | NM_001005241 | chr15 | 19883804 | 19884310 | 221 | 0.33 | 0.91 | -0.59 |
| *OR4X1* | NM_001004726 | chr11 | 48241594 | 48242080 | -151 | 0.08 | 0.71 | -0.63 |
| *OR51A7* | NM_001004749 | chr11 | 4885081 | 4885582 | 156 | 0.35 | 0.84 | -0.49 |
| *OR51B4* | NM_033179 | chr11 | 5281734 | 5282243 | -2236 | 0.19 | 0.56 | -0.37 |
| *OR51B5* | NM_001005567 | chr11 | 5323351 | 5323733 | -2212 | 0.17 | 0.75 | -0.58 |
| *OR51E2* | NM_030774 | chr11 | 4676590 | 4677097 | -1191 | 0.08 | 0.73 | -0.66 |
| *OR51F1* | NM_001004752 | chr11 | 4747587 | 4748076 | -108 | 0.31 | 0.74 | -0.43 |
| *OR51F2* | NM_001004753 | chr11 | 4798704 | 4799233 | -222 | 0.24 | 0.84 | -0.60 |
| *OR51I2* | NM_001004754 | chr11 | 5428878 | 5429405 | -2152 | 0.12 | 0.50 | -0.38 |
| *OR51L1* | NM_001004755 | chr11 | 4976429 | 4976928 | -109 | 0.19 | 0.56 | -0.37 |
| *OR51M1* | NM_001004756 | chr11 | 5366803 | 5367291 | -157 | 0.08 | 0.89 | -0.81 |
| *OR51Q1* | NM_001004757 | chr11 | 5398674 | 5399163 | -1087 | 0.33 | 0.71 | -0.39 |
| *OR52A4* | NM_001005222 | chr11 | 5101169 | 5101668 | -2034 | 0.11 | 0.60 | -0.49 |
| *OR52B2* | NM_001004052 | chr11 | 6148073 | 6148594 | -201 | 0.02 | 0.44 | -0.42 |
| *OR52B4* | NM_001005161 | chr11 | 4347548 | 4348066 | -1706 | 0.16 | 0.73 | -0.57 |
| *OR52D1* | NM_001005163 | chr11 | 5464096 | 5464647 | -2140 | 0.15 | 0.50 | -0.35 |
| *OR52I2* | NM_001005170 | chr11 | 4562179 | 4562628 | -2214 | 0.34 | 0.85 | -0.51 |
| *OR52K2* | NM_001005172 | chr11 | 4424723 | 4425278 | -2144 | 0.27 | 0.69 | -0.42 |
| *OR52L1* | NM_001005173 | chr11 | 5964651 | 5965176 | -122 | 0.14 | 0.74 | -0.59 |
| *OR52N4* | NM_001005175 | chr11 | 5730058 | 5730542 | -2198 | 0.02 | 0.44 | -0.42 |
| *OR56A1* | NM_001001917 | chr11 | 6005449 | 6005990 | -209 | 0.17 | 0.51 | -0.34 |
| *OR56A5* | NM_001146033 | chr11 | 5948149 | 5948647 | -2098 | 0.22 | 0.57 | -0.35 |
| *OR56B1* | NM_001005180 | chr11 | 5713964 | 5714456 | -112 | 0.06 | 0.54 | -0.48 |
| *OR5AP2* | NM_001002925 | chr11 | 56166363 | 56166868 | -124 | 0.18 | 0.48 | -0.30 |
| *OR5B12* | NM_001004733 | chr11 | 57964159 | 57964655 | -207 | 0.17 | 0.64 | -0.47 |
| *OR5B21* | NM_001005218 | chr11 | 58033502 | 58034006 | -1600 | 0.04 | 0.66 | -0.62 |
| *OR5C1* | NM_001001923 | chr9 | 124589628 | 124590040 | -1198 | 0.27 | 0.70 | -0.43 |
| *OR5D14* | NM_001004735 | chr11 | 55317167 | 55317626 | -2210 | 0.14 | 0.54 | -0.40 |
| *OR5D16* | NM_001005496 | chr11 | 55362425 | 55362937 | -122 | 0.19 | 0.64 | -0.45 |
| *OR5H14* | NM_001005514 | chr3 | 99350549 | 99351090 | -99 | 0.27 | 0.59 | -0.32 |
| *OR5H15* | NM_001005515 | chr3 | 99369877 | 99370418 | -85 | 0.08 | 0.39 | -0.31 |
| *OR5M10* | NM_001004741 | chr11 | 56101620 | 56102173 | -123 | 0.34 | 0.65 | -0.32 |
| *OR5P3* | NM_153445 | chr11 | 7805576 | 7806084 | -1735 | 0.48 | 0.84 | -0.37 |
| *OR5T2* | NM_001004746 | chr11 | 55757201 | 55757681 | -204 | 0.51 | 0.82 | -0.31 |
| *OR5W2* | NM_001001960 | chr11 | 55440013 | 55440527 | -1636 | 0.17 | 0.71 | -0.54 |
| *OR6A2* | NM_003696 | chr11 | 6773663 | 6774156 | -194 | 0.23 | 0.54 | -0.31 |
| *OR6B1* | NM_001005281 | chr7 | 143330067 | 143330569 | -1704 | 0.49 | 0.79 | -0.30 |
| *OR6C2* | NM_054105 | chr12 | 54132209 | 54132698 | 189 | 0.18 | 0.52 | -0.34 |
| *OR6C65* | NM_001005518 | chr12 | 54078139 | 54078629 | -2195 | 0.43 | 0.96 | -0.54 |
| *OR6C76* | NM_001005183 | chr12 | 54103870 | 54104393 | -2172 | 0.56 | 0.87 | -0.30 |
| *OR6K2* | NM_001005279 | chr1 | 156936931 | 156937406 | -102 | 0.29 | 0.68 | -0.39 |
| *OR6K6* | NM_001005184 | chr1 | 156989350 | 156989845 | -1631 | 0.28 | 0.63 | -0.36 |
| *OR6N2* | NM_001005278 | chr1 | 157015027 | 157015585 | -1257 | 0.39 | 0.81 | -0.41 |
| *OR6S1* | NM_001001968 | chr14 | 20179650 | 20180148 | -209 | 0.19 | 0.55 | -0.35 |
| *OR7A17* | NM_030901 | chr19 | 14854043 | 14854511 | -1110 | 0.46 | 0.89 | -0.43 |
| *OR7C1* | NM_198944 | chr19 | 14773655 | 14774044 | -1901 | 0.47 | 0.79 | -0.32 |
| *OR8B8* | NM_012378 | chr11 | 123817561 | 123818060 | -1619 | 0.33 | 0.69 | -0.35 |
| *OR8D4* | NM_001005197 | chr11 | 123279932 | 123280459 | -2152 | 0.21 | 0.56 | -0.35 |
| *OR8J3* | NM_001004064 | chr11 | 55663392 | 55663916 | -1884 | 0.27 | 0.57 | -0.30 |
| *OR8K1* | NM_001002907 | chr11 | 55869626 | 55870149 | -202 | 0.14 | 0.82 | -0.69 |
| *OR9G1* | NM_001005213 | chr11 | 56222010 | 56222566 | -2151 | 0.28 | 0.65 | -0.38 |
| *OR9G9* | NM_001013358 | chr11 | 56222010 | 56222566 | -2151 | 0.28 | 0.65 | -0.38 |
| *OR9K2* | NM_001005243 | chr12 | 53809510 | 53810053 | -37 | 0.31 | 0.64 | -0.33 |
| *OTOA* | NM_144672 | chr16 | 21597228 | 21597777 | 167 | 0.15 | 0.54 | -0.39 |
| *OTOL1* | NM_001080440 | chr3 | 162695991 | 162696546 | -1020 | 0.15 | 0.53 | -0.38 |
| *OXCT1* | NM_000436 | chr5 | 41907501 | 41908003 | -1204 | 0.27 | 0.57 | -0.31 |
| *P2RY12* | NM_176876 | chr3 | 152542708 | 152543156 | -1657 | 0.27 | 0.61 | -0.34 |
| *P2RY14* | NM_001081455 | chr3 | 152451097 | 152451605 | -1663 | 0.06 | 0.70 | -0.64 |
| *PADI3* | NM_016233 | chr1 | 17445838 | 17446395 | -2062 | 0.20 | 0.56 | -0.36 |
| *PADI6* | NM_207421 | chr1 | 17569444 | 17569946 | -1632 | 0.39 | 0.72 | -0.33 |
| *PAK7* | NM_020341 | chr20 | 9769631 | 9770149 | -2203 | 0.06 | 0.41 | -0.34 |
| *PAMR1* | NM_001001991 | chr11 | 35505135 | 35505666 | -1648 | 0.42 | 0.88 | -0.46 |
| *PAPOLB* | NM_020144 | chr7 | 4869147 | 4869635 | -1240 | 0.47 | 0.78 | -0.31 |
| *PAQR3* | NM_001040202 | chr4 | 80081730 | 80082034 | -2276 | 0.03 | 0.48 | -0.45 |
| *PATE1* | NM_138294 | chr11 | 125120422 | 125120916 | -728 | 0.36 | 0.75 | -0.39 |
| *PATE2* | NM_212555 | chr11 | 125154880 | 125155369 | -1200 | 0.28 | 0.75 | -0.47 |
| *PBX1* | NM_002585 | chr1 | 162793460 | 162793970 | -1710 | 0.25 | 0.66 | -0.41 |
| *PCDH20* | NM_022843 | chr13 | 60888651 | 60889149 | -1244 | 0.46 | 0.89 | -0.43 |
| *PCDHA13* | NM_018904 | chr5 | 140240154 | 140240663 | -1628 | 0.42 | 0.84 | -0.42 |
| *PCDHA4* | NM_018907 | chr5 | 140165370 | 140165919 | -1210 | 0.14 | 0.47 | -0.33 |
| *PCDHAC2* | NM_018899 | chr5 | 140323864 | 140324305 | -2210 | 0.35 | 0.90 | -0.55 |
| *PCDHB12* | NM_018932 | chr5 | 140566503 | 140567019 | -1713 | 0.23 | 0.79 | -0.55 |
| *PCDHB18* | NR_001281 | chr5 | 140592243 | 140592740 | -1629 | 0.13 | 0.49 | -0.36 |
| *PCDHB6* | NM_018939 | chr5 | 140507584 | 140508038 | -2211 | 0.17 | 0.51 | -0.34 |
| *PCDHB7* | NM_018940 | chr5 | 140531543 | 140532046 | -631 | 0.13 | 0.45 | -0.32 |
| *PCDHB8* | NM_019120 | chr5 | 140536839 | 140537346 | -520 | 0.15 | 0.57 | -0.42 |
| *PCDHB9* | NM_019119 | chr5 | 140547062 | 140547591 | 250 | 0.14 | 0.59 | -0.44 |
| *PCDHGA10* | NM_018913 | chr5 | 140772263 | 140772768 | -410 | 0.10 | 0.41 | -0.32 |
| *PCNXL2* | NM_014801 | chr1 | 231499440 | 231499949 | -1612 | 0.46 | 0.89 | -0.43 |
| *PCTP* | NM_001102402 | chr17 | 51180938 | 51181464 | -2153 | 0.13 | 0.55 | -0.41 |
| *PDC* | NM_002597 | chr1 | 184698859 | 184699341 | -2238 | 0.55 | 0.88 | -0.33 |
| *PDE1A* | NM_001003683 | chr2 | 183095957 | 183096418 | -477 | 0.34 | 0.70 | -0.36 |
| *PDE4B* | NM_001037340 | chr1 | 66229778 | 66230273 | -951 | 0.24 | 0.54 | -0.30 |
| *PDE4DIP* | NM_001002810 | chr1 | 143707242 | 143707732 | -1108 | 0.13 | 0.43 | -0.30 |
| *PDLIM3* | NM_001114107 | chr4 | 186695048 | 186695229 | -1432 | 0.39 | 0.73 | -0.34 |
| *PDZK1* | NM_002614 | chr1 | 144439067 | 144439569 | 236 | 0.46 | 0.81 | -0.35 |
| *PEG3AS* | NR_023847 | chr19 | 62014668 | 62015172 | -1619 | 0.27 | 0.71 | -0.44 |
| *PER4* | NR_002790 | chr7 | 9640016 | 9640512 | -160 | 0.30 | 0.60 | -0.30 |
| *PGBD5* | NM_024554 | chr1 | 228580450 | 228580930 | -700 | 0.26 | 0.67 | -0.41 |
| *PGR* | NM_000926 | chr11 | 100507234 | 100507691 | -1708 | 0.27 | 0.62 | -0.35 |
| *PHACTR3* | NM_183244 | chr20 | 57727240 | 57727587 | -2245 | 0.22 | 0.86 | -0.64 |
| *PI15* | NM_015886 | chr8 | 75897449 | 75897954 | -1624 | 0.10 | 0.78 | -0.69 |
| *PIGC* | NM_002642 | chr1 | 170680724 | 170681220 | -1119 | 0.57 | 0.88 | -0.31 |
| *PIGT* | NM_015937 | chr20 | 43476713 | 43477166 | -1197 | 0.11 | 0.55 | -0.44 |
| *PIK3CG* | NM_002649 | chr7 | 106292300 | 106292789 | -614 | 0.03 | 0.63 | -0.60 |
| *PIWIL3* | NM_001008496 | chr22 | 23502357 | 23502863 | -1927 | 0.27 | 0.82 | -0.55 |
| *PIWIL4* | NM_152431 | chr11 | 93937682 | 93938240 | -2160 | 0.50 | 0.84 | -0.35 |
| *PLA1A* | NM_015900 | chr3 | 120798938 | 120799447 | -218 | 0.18 | 0.51 | -0.33 |
| *PLA2G7* | NM_005084 | chr6 | 46811617 | 46812122 | -759 | 0.32 | 0.73 | -0.41 |
| *PLAC1L* | NM_173801 | chr11 | 59562446 | 59562988 | -1606 | 0.38 | 0.71 | -0.34 |
| *PLAC8L1* | NM_001029869 | chr5 | 145466438 | 145466617 | -2388 | 0.16 | 0.48 | -0.33 |
| *PLCH1* | NM_001130960 | chr3 | 156877169 | 156877687 | -629 | 0.20 | 0.60 | -0.40 |
| *PLCXD3* | NM_001005473 | chr5 | 41546930 | 41547452 | -704 | 0.05 | 0.35 | -0.30 |
| *PLP1* | NM_000533 | chrX | 102917124 | 102917636 | -1029 | 0.32 | 0.70 | -0.38 |
| *PLSCR5* | NM_001085420 | chr3 | 147807882 | 147808429 | -1462 | 0.32 | 0.73 | -0.41 |
| *PMP2* | NM_002677 | chr8 | 82524258 | 82524747 | -2228 | 0.03 | 0.36 | -0.32 |
| *PNLIP* | NM_000936 | chr10 | 118294041 | 118294538 | -1127 | 0.16 | 0.75 | -0.59 |
| *PNLIPRP3* | NM_001011709 | chr10 | 118175441 | 118175931 | -1727 | 0.12 | 0.69 | -0.57 |
| *POLR2J* | NM_006234 | chr7 | 101907672 | 101908155 | -1527 | 0.15 | 0.47 | -0.31 |
| *POP1* | NM_001145860 | chr8 | 99196306 | 99196438 | -2324 | 0.49 | 0.86 | -0.37 |
| *POSTN* | NM_001135934 | chr13 | 37071367 | 37071869 | -637 | 0.19 | 0.52 | -0.33 |
| *POTEA* | NM_001002920 | chr8 | 43264851 | 43265382 | -1624 | 0.50 | 0.86 | -0.36 |
| *POTEC* | NM_001137671 | chr18 | 14534538 | 14535073 | -1206 | 0.40 | 0.78 | -0.38 |
| *POU5F1B* | NM_001159542 | chr8 | 128495047 | 128495554 | -1737 | 0.50 | 0.81 | -0.31 |
| *POU5F1P1* | NR_002304 | chr8 | 128495047 | 128495554 | -1737 | 0.50 | 0.81 | -0.31 |
| *POU6F2* | NM_007252 | chr7 | 39010492 | 39010953 | -2209 | 0.14 | 0.48 | -0.35 |
| *PPM1J* | NM_005167 | chr1 | 113061969 | 113062496 | -2759 | 0.51 | 0.81 | -0.30 |
| *PPP1R3A* | NM_002711 | chr7 | 113346173 | 113346569 | -53 | 0.16 | 0.50 | -0.35 |
| *PPP1R8* | NM_002713 | chr1 | 28027464 | 28028027 | -2133 | 0.30 | 0.63 | -0.32 |
| *PPP2R2B* | NM_181674 | chr5 | 146417794 | 146418313 | -2160 | 0.43 | 0.81 | -0.38 |
| *PPP3CB* | NM_001142353 | chr10 | 74926594 | 74927103 | -1060 | 0.18 | 0.58 | -0.40 |
| *PPP3R2* | NM_147180 | chr9 | 103398532 | 103399003 | -1663 | 0.12 | 0.43 | -0.32 |
| *PPP5C* | NM_006247 | chr19 | 51539715 | 51539966 | -2292 | 0.37 | 0.69 | -0.32 |
| *PRAMEF13* | NM_001024661 | chr1 | 13546887 | 13547374 | -1032 | 0.28 | 0.62 | -0.33 |
| *PRAMEF14* | NM_001099854 | chr1 | 13546887 | 13547374 | -1032 | 0.28 | 0.62 | -0.33 |
| *PRAMEF15* | NM_001098376 | chr1 | 13292810 | 13293137 | -788 | 0.30 | 0.63 | -0.32 |
| *PRAMEF2* | NM_023014 | chr1 | 12838410 | 12838868 | -888 | 0.44 | 0.76 | -0.33 |
| *PRAMEF20* | NM_001099852 | chr1 | 13607536 | 13607994 | -1728 | 0.05 | 0.35 | -0.30 |
| *PRAMEF6* | NM_001010889 | chr1 | 12931812 | 12932194 | -2010 | 0.27 | 0.57 | -0.30 |
| *PRAMEF9* | NM_001010890 | chr1 | 13292810 | 13293137 | -788 | 0.30 | 0.63 | -0.32 |
| *PRB1* | NM_005039 | chr12 | 11400759 | 11401255 | -1216 | 0.40 | 0.79 | -0.40 |
| *PRB3* | NM_006249 | chr12 | 11314863 | 11315373 | -1210 | 0.22 | 0.91 | -0.70 |
| *PRDM1* | NM_182907 | chr6 | 106653026 | 106653531 | -228 | 0.41 | 0.79 | -0.38 |
| *PRDM10* | NM_199438 | chr11 | 129323095 | 129323567 | -604 | 0.63 | 0.95 | -0.32 |
| *PRDM11* | NM_020229 | chr11 | 45071252 | 45071772 | -627 | 0.50 | 0.86 | -0.36 |
| *PRDX4* | NM_006406 | chrX | 23594624 | 23595111 | -697 | 0.33 | 0.71 | -0.38 |
| *PRG1* | NR_026881 | chr19 | 48543256 | 48543714 | -1562 | 0.26 | 0.76 | -0.49 |
| *PRG2* | NM_002728 | chr11 | 56916691 | 56917187 | -2233 | 0.19 | 0.66 | -0.48 |
| *PRICKLE2* | NM_198859 | chr3 | 64187168 | 64187658 | -1242 | 0.34 | 0.67 | -0.32 |
| *PRKAB2* | NM_005399 | chr1 | 145112042 | 145112528 | -1532 | 0.17 | 0.52 | -0.34 |
| *PRKACG* | NM_002732 | chr9 | 70820209 | 70820701 | -1596 | 0.21 | 0.54 | -0.33 |
| *PRKCG* | NM_002739 | chr19 | 59076254 | 59076787 | -757 | 0.11 | 0.43 | -0.32 |
| *PRKG1* | NM_001098512 | chr10 | 52419577 | 52420076 | -1123 | 0.34 | 0.69 | -0.34 |
| *PRKG2* | NM_006259 | chr4 | 82345686 | 82346201 | -704 | 0.49 | 0.80 | -0.31 |
| *PRL* | NM_000948 | chr6 | 22407182 | 22407674 | -1719 | 0.37 | 0.75 | -0.38 |
| *PRNT* | NR_024267 | chr20 | 4670834 | 4671327 | -1766 | 0.20 | 0.50 | -0.30 |
| *PROL1* | NM_021225 | chr4 | 71295769 | 71296313 | -2146 | 0.31 | 0.77 | -0.45 |
| *PRR21* | NM_001080835 | chr2 | 240632305 | 240632811 | -1486 | 0.42 | 0.73 | -0.31 |
| *PRSS3* | NM_002771 | chr9 | 33783617 | 33784123 | -1688 | 0.21 | 0.73 | -0.52 |
| *PSAPL1* | NM_001085382 | chr4 | 7489439 | 7489950 | -2094 | 0.33 | 0.70 | -0.37 |
| *PTPN13* | NM_006264 | chr4 | 87732057 | 87732504 | -2210 | 0.34 | 0.69 | -0.35 |
| *PTX3* | NM_002852 | chr3 | 158637026 | 158637529 | 4 | 0.32 | 0.73 | -0.40 |
| *PWRN2* | NR_026647 | chr15 | 21967468 | 21967998 | -1587 | 0.10 | 0.42 | -0.32 |
| *PXT1* | NM_152990 | chr6 | 36476154 | 36476452 | -14 | 0.16 | 0.48 | -0.32 |
| *PYDC2* | NM_001083308 | chr3 | 192660687 | 192661087 | -758 | 0.15 | 0.59 | -0.43 |
| *PYHIN1* | NM_152501 | chr1 | 157166650 | 157167148 | -1066 | 0.31 | 0.74 | -0.42 |
| *RAB37* | NM_175738 | chr17 | 70176982 | 70177459 | -1643 | 0.47 | 0.82 | -0.35 |
| *RAB3GAP2* | NM_012414 | chr1 | 218513449 | 218513951 | -1234 | 0.39 | 0.74 | -0.34 |
| *RAD54B* | NM_012415 | chr8 | 95557713 | 95558114 | -1427 | 0.39 | 0.80 | -0.41 |
| *RAG1* | NM_000448 | chr11 | 36543736 | 36544210 | -2165 | 0.04 | 0.69 | -0.65 |
| *RAG2* | NM_000536 | chr11 | 36576241 | 36576759 | -112 | 0.42 | 0.88 | -0.45 |
| *RAI14* | NM_001145521 | chr5 | 34719040 | 34719557 | -1069 | 0.45 | 0.79 | -0.34 |
| *RASGRP1* | NM_001128602 | chr15 | 36645018 | 36645517 | -968 | 0.21 | 0.57 | -0.36 |
| *RBM19* | NM_001146698 | chr12 | 112890348 | 112890834 | -2032 | 0.43 | 0.84 | -0.41 |
| *RBM22* | NM_018047 | chr5 | 150061832 | 150062321 | -1214 | 0.41 | 0.71 | -0.30 |
| *RBMS2* | NM_002898 | chr12 | 55200740 | 55201146 | -932 | 0.04 | 0.54 | -0.50 |
| *RCAN2* | NM_005822 | chr6 | 46401438 | 46401954 | -206 | 0.58 | 0.92 | -0.33 |
| *RDH14* | NM_020905 | chr2 | 18606890 | 18607390 | -1700 | 0.20 | 0.69 | -0.49 |
| *REG1P* | NR_002714 | chr2 | 79219950 | 79220474 | -1151 | 0.01 | 0.31 | -0.30 |
| *REXO1L2P* | NR_003594 | chr8 | 86886089 | 86886382 | -874 | 0.15 | 0.50 | -0.35 |
| *RFESD* | NM_001131065 | chr5 | 95006300 | 95006803 | -1786 | 0.08 | 0.48 | -0.41 |
| *RGS4* | NM_001102445 | chr1 | 161304746 | 161305236 | -28 | 0.21 | 0.58 | -0.38 |
| *RGS5* | NM_003617 | chr1 | 161440381 | 161440696 | -1042 | 0.22 | 0.57 | -0.35 |
| *RGS9* | NM_001081955 | chr17 | 60561570 | 60562043 | -2203 | 0.34 | 0.68 | -0.34 |
| *RHOG* | NM_001665 | chr11 | 3820944 | 3821249 | -2307 | 0.15 | 0.50 | -0.35 |
| *RIMBP2* | NM_015347 | chr12 | 129568203 | 129568726 | -101 | 0.08 | 0.40 | -0.32 |
| *RIMS2* | NM_014677 | chr8 | 104900389 | 104900876 | 41 | 0.49 | 0.79 | -0.30 |
| *RNASE11* | NM_145250 | chr14 | 20129937 | 20130441 | -1932 | 0.56 | 0.90 | -0.33 |
| *RNASE12* | NM_001024822 | chr14 | 20129937 | 20130441 | -1467 | 0.56 | 0.90 | -0.33 |
| *RNASE2* | NM_002934 | chr14 | 20491540 | 20492046 | -1676 | 0.52 | 0.87 | -0.35 |
| *RNASE3* | NM_002935 | chr14 | 20426963 | 20427505 | -2167 | 0.39 | 0.72 | -0.33 |
| *RNF17* | NM_031277 | chr13 | 24233864 | 24234226 | -2255 | 0.34 | 0.74 | -0.40 |
| *RNF175* | NM_173662 | chr4 | 154902662 | 154903174 | -2081 | 0.11 | 0.47 | -0.36 |
| *RNF182* | NM_152737 | chr6 | 14030741 | 14031216 | -2202 | 0.17 | 0.49 | -0.33 |
| *RNF217* | NM_152553 | chr6 | 125345555 | 125346050 | -409 | 0.50 | 0.87 | -0.37 |
| *RNF24* | NM_001134337 | chr20 | 3945782 | 3946288 | -1819 | 0.09 | 0.49 | -0.40 |
| *RNF43* | NM_017763 | chr17 | 53850402 | 53850901 | -721 | 0.21 | 0.80 | -0.59 |
| *RNF5P1* | NR_003129 | chr8 | 38578299 | 38578797 | -616 | 0.31 | 0.61 | -0.31 |
| *ROPN1B* | NM_001012337 | chr3 | 127168742 | 127169242 | -1725 | 0.31 | 0.64 | -0.33 |
| *RP1* | NM_006269 | chr8 | 55688742 | 55689290 | -2163 | 0.30 | 0.59 | -0.30 |
| *RPRM* | NM_019845 | chr2 | 154045769 | 154045948 | -2290 | 0.14 | 0.52 | -0.38 |
| *RPTN* | NM_001122965 | chr1 | 150398782 | 150399282 | -704 | 0.10 | 0.73 | -0.62 |
| *RUNX1T1* | NM_175636 | chr8 | 93100434 | 93100947 | -1606 | 0.13 | 0.44 | -0.31 |
| *S100A11* | NM_005620 | chr1 | 150277654 | 150278150 | -1767 | 0.29 | 0.79 | -0.49 |
| *S100A5* | NM_002962 | chr1 | 151782712 | 151783224 | -2103 | 0.31 | 0.67 | -0.36 |
| *S100A7A* | NM_176823 | chr1 | 151653709 | 151654107 | -1715 | 0.35 | 0.78 | -0.42 |
| *S100B* | NM_006272 | chr21 | 46851821 | 46851918 | -2406 | 0.38 | 0.75 | -0.36 |
| *SALL2* | NM_005407 | chr14 | 21076757 | 21077241 | -1822 | 0.09 | 0.52 | -0.43 |
| *SCGN* | NM_006998 | chr6 | 25757968 | 25758531 | -2157 | 0.18 | 0.57 | -0.39 |
| *SCN11A* | NM_014139 | chr3 | 38969255 | 38969458 | -2300 | 0.43 | 0.77 | -0.34 |
| *SDR16C6* | NM_001145251 | chr8 | 57472433 | 57472941 | -2209 | 0.58 | 0.89 | -0.31 |
| *SDR39U1* | NM_020195 | chr14 | 23982310 | 23982789 | -702 | 0.25 | 0.75 | -0.50 |
| *SEL1L* | NM_005065 | chr14 | 81071862 | 81072358 | -2152 | 0.31 | 0.70 | -0.39 |
| *SELL* | NM_000655 | chr1 | 167947486 | 167948014 | -289 | 0.32 | 0.79 | -0.48 |
| *SEMG1* | NM_003007 | chr20 | 43266656 | 43267200 | -2159 | 0.13 | 0.75 | -0.62 |
| *SERPINA7* | NM_000354 | chrX | 105168593 | 105169085 | -1118 | 0.13 | 0.69 | -0.56 |
| *SERPINB11* | NM_080475 | chr18 | 59518752 | 59519288 | -2153 | 0.33 | 0.78 | -0.46 |
| *SERPINB12* | NM_080474 | chr18 | 59373915 | 59374433 | -198 | 0.41 | 0.75 | -0.34 |
| *SERPINB3* | NM_006919 | chr18 | 59482543 | 59482619 | -2404 | 0.28 | 0.89 | -0.61 |
| *SERPINB4* | NM_002974 | chr18 | 59464332 | 59464882 | -2125 | 0.46 | 0.91 | -0.44 |
| *SERPINB7* | NM_001040147 | chr18 | 59568835 | 59569374 | -2151 | 0.24 | 0.63 | -0.40 |
| *SFXN1* | NM_022754 | chr5 | 174836148 | 174836537 | -1776 | 0.07 | 0.60 | -0.53 |
| *SGCD* | NM_000337 | chr5 | 155683905 | 155684403 | -2190 | 0.26 | 0.67 | -0.41 |
| *SGK269* | NM_024776 | chr15 | 75366607 | 75366807 | -2319 | 0.16 | 0.47 | -0.30 |
| *SH3GLP3* | NR_026577 | chr17_random | 1621924 | 1622435 | -48 | 0.39 | 0.83 | -0.45 |
| *SI* | NM_001041 | chr3 | 166279848 | 166280334 | -1115 | 0.19 | 0.52 | -0.32 |
| *SIDT1* | NM_017699 | chr3 | 114732071 | 114732554 | -1594 | 0.21 | 0.64 | -0.43 |
| *SIRPB1* | NM_001083910 | chr20 | 1550060 | 1550553 | -1617 | 0.21 | 0.58 | -0.37 |
| *SIRPB2* | NM_001122962 | chr20 | 1420188 | 1420682 | -202 | 0.14 | 0.49 | -0.35 |
| *SIRPG* | NM_001039508 | chr20 | 1587776 | 1588314 | -1620 | 0.12 | 0.44 | -0.32 |
| *SLA* | NM_001045556 | chr8 | 134184338 | 134184856 | -105 | 0.21 | 0.51 | -0.30 |
| *SLAMF7* | NM_021181 | chr1 | 158975296 | 158975776 | -164 | 0.23 | 0.65 | -0.43 |
| *SLC10A2* | NM_000452 | chr13 | 102518040 | 102518537 | -1091 | 0.47 | 0.79 | -0.32 |
| *SLC10A5* | NM_001010893 | chr8 | 82769629 | 82770125 | -115 | 0.25 | 0.57 | -0.32 |
| *SLC10A6* | NM_197965 | chr4 | 87990679 | 87991126 | -1462 | 0.44 | 0.76 | -0.32 |
| *SLC16A9* | NM_194298 | chr10 | 61141509 | 61141994 | -2096 | 0.13 | 0.46 | -0.32 |
| *SLC18A1* | NM_001135691 | chr8 | 20087214 | 20087411 | -2315 | 0.20 | 0.57 | -0.36 |
| *SLC1A6* | NM_005071 | chr19 | 14945545 | 14945857 | -971 | 0.45 | 0.75 | -0.30 |
| *SLC24A5* | NM_205850 | chr15 | 46199754 | 46200266 | -450 | 0.25 | 0.65 | -0.39 |
| *SLC25A21* | NM_030631 | chr14 | 36713031 | 36713534 | -1666 | 0.25 | 0.57 | -0.32 |
| *SLC25A24* | NM_213651 | chr1 | 108538877 | 108539363 | -2234 | 0.48 | 0.80 | -0.31 |
| *SLC26A4* | NM_000441 | chr7 | 107086959 | 107087475 | -1098 | 0.42 | 0.71 | -0.30 |
| *SLC30A8* | NM_173851 | chr8 | 118215587 | 118216082 | -682 | 0.22 | 0.66 | -0.44 |
| *SLC35F4* | NM_001080455 | chr14 | 57133214 | 57133736 | -107 | 0.13 | 0.43 | -0.31 |
| *SLC36A4* | NM_152313 | chr11 | 92572140 | 92572667 | -1660 | 0.14 | 0.69 | -0.55 |
| *SLC38A4* | NM_001143824 | chr12 | 45506901 | 45507371 | -1089 | 0.25 | 0.58 | -0.33 |
| *SLC39A12* | NM_001145195 | chr10 | 18280315 | 18280808 | -211 | 0.49 | 0.84 | -0.35 |
| *SLC44A5* | NM_001130058 | chr1 | 75850283 | 75850778 | -1143 | 0.13 | 0.57 | -0.44 |
| *SLC4A10* | NM_022058 | chr2 | 162186657 | 162187110 | -2206 | 0.21 | 0.61 | -0.40 |
| *SLC5A12* | NM_178498 | chr11 | 26701025 | 26701499 | -1112 | 0.18 | 0.74 | -0.56 |
| *SLC5A7* | NM_021815 | chr2 | 107967938 | 107968469 | -1222 | 0.20 | 0.51 | -0.31 |
| *SLC6A11* | NM_014229 | chr3 | 10831977 | 10832474 | -690 | 0.42 | 0.87 | -0.46 |
| *SLC6A19* | NM_001003841 | chr5 | 1252575 | 1253068 | -1887 | 0.35 | 0.78 | -0.43 |
| *SLC7A11* | NM_014331 | chr4 | 139384443 | 139384963 | -1750 | 0.15 | 0.50 | -0.36 |
| *SLC7A13* | NM_138817 | chr8 | 87312227 | 87312673 | -730 | 0.46 | 0.88 | -0.42 |
| *SLC8A3* | NM_001130417 | chr14 | 69617029 | 69617516 | -596 | 0.27 | 0.76 | -0.49 |
| *SLMO2* | NM_016045 | chr20 | 57052645 | 57053147 | -1600 | 0.15 | 0.50 | -0.36 |
| *SMCP* | NM_030663 | chr1 | 151116963 | 151117481 | -199 | 0.22 | 0.57 | -0.35 |
| *SNAI2* | NM_003068 | chr8 | 49997532 | 49998013 | -1231 | 0.25 | 0.71 | -0.46 |
| *SNAR-C1* | NR_024220 | chr19 | 53105800 | 53105849 | -2294 | 0.29 | 0.66 | -0.37 |
| *SNAR-C2* | NR_024217 | chr19 | 53105800 | 53105849 | -2294 | 0.29 | 0.66 | -0.37 |
| *SNAR-C4* | NR_024218 | chr19 | 53133158 | 53133217 | -1421 | 0.46 | 0.78 | -0.32 |
| *SNAR-C5* | NR_024219 | chr19 | 53105800 | 53105849 | -2294 | 0.29 | 0.66 | -0.37 |
| *SNORA42* | NR_002974 | chr1 | 154156928 | 154157445 | -729 | 0.10 | 0.52 | -0.42 |
| *SNORA70C* | NR_003708 | chr9 | 118985321 | 118985740 | -2231 | 0.23 | 0.88 | -0.65 |
| *SNORA74A* | NR_002915 | chr5 | 138641049 | 138641464 | -1110 | 0.33 | 0.76 | -0.43 |
| *SNORA75* | NR_002921 | chr2 | 232029331 | 232029852 | -700 | 0.34 | 0.67 | -0.33 |
| *SNORD109A* | NR_001295 | chr15 | 23073157 | 23073664 | -1171 | 0.10 | 0.47 | -0.38 |
| *SNORD109B* | NR_001289 | chr15 | 23073157 | 23073664 | -1171 | 0.10 | 0.47 | -0.38 |
| *SNORD113-3* | NR_003231 | chr14 | 100463132 | 100463608 | -2638 | 0.33 | 0.72 | -0.39 |
| *SNORD113-8* | NR_003236 | chr14 | 100478896 | 100479403 | -390 | 0.25 | 0.60 | -0.35 |
| *SNORD113-9* | NR_003237 | chr14 | 100478896 | 100479403 | -2588 | 0.25 | 0.60 | -0.35 |
| *SNORD17* | NR_003045 | chr20 | 17893141 | 17893638 | -1800 | 0.13 | 0.65 | -0.52 |
| *SNORD20* | NR_002908 | chr2 | 232029331 | 232029852 | -113 | 0.34 | 0.67 | -0.33 |
| *SNORD8* | NR_002916 | chr14 | 20935914 | 20936098 | -606 | 0.38 | 0.89 | -0.52 |
| *SNRNP48* | NM_152551 | chr6 | 7534092 | 7534597 | -1085 | 0.19 | 0.52 | -0.32 |
| *SNX16* | NM_022133 | chr8 | 82918391 | 82918922 | -1580 | 0.38 | 0.75 | -0.37 |
| *SNX2* | NM_003100 | chr5 | 122136673 | 122137157 | -1733 | 0.27 | 0.66 | -0.39 |
| *SPACA5* | NM_205856 | chrX | 47870797 | 47871224 | -535 | 0.50 | 0.80 | -0.30 |
| *SPAG11B* | NM_016512 | chr8 | 7310950 | 7311057 | -2401 | 0.19 | 0.51 | -0.32 |
| *SPANXB1* | NM_032461 | chrX | 139922461 | 139923001 | -1695 | 0.10 | 0.57 | -0.47 |
| *SPANXB2* | NM_145664 | chrX | 139922461 | 139923001 | -1695 | 0.10 | 0.57 | -0.47 |
| *SPANXC* | NM_022661 | chrX | 140164147 | 140164378 | 49 | 0.53 | 0.86 | -0.32 |
| *SPANXD* | NM_032417 | chrX | 140615951 | 140616488 | -1955 | 0.27 | 0.64 | -0.37 |
| *SPANXE* | NM_145665 | chrX | 140615951 | 140616488 | -1898 | 0.27 | 0.64 | -0.37 |
| *SPANXF1* | NM_139019 | chrX | 139922461 | 139923001 | -1695 | 0.10 | 0.57 | -0.47 |
| *SPANXN1* | NM_001009614 | chrX | 144134865 | 144135364 | -1683 | 0.30 | 0.84 | -0.54 |
| *SPANXN5* | NM_001009616 | chrX | 52844003 | 52844484 | -1130 | 0.44 | 0.74 | -0.30 |
| *SPC25* | NM_020675 | chr2 | 169456056 | 169456531 | -1103 | 0.28 | 0.65 | -0.37 |
| *SPG20* | NM_001142294 | chr13 | 35843825 | 35844309 | -1750 | 0.13 | 0.88 | -0.74 |
| *SPINK1* | NM_003122 | chr5 | 147192406 | 147192927 | -1213 | 0.35 | 0.97 | -0.62 |
| *SPINK5* | NM_001127698 | chr5 | 147422546 | 147423044 | -932 | 0.33 | 0.64 | -0.31 |
| *SPINK8* | NM_001080525 | chr3 | 48345104 | 48345577 | -505 | 0.18 | 0.48 | -0.30 |
| *SPINLW1* | NM_020398 | chr20 | 43611144 | 43611646 | -1953 | 0.26 | 0.70 | -0.44 |
| *SPINT3* | NM_006652 | chr20 | 43579669 | 43580158 | -2235 | 0.10 | 0.78 | -0.68 |
| *SPRR1A* | NM_005987 | chr1 | 151221292 | 151221814 | -1627 | 0.22 | 0.78 | -0.56 |
| *SPRR1B* | NM_003125 | chr1 | 151267884 | 151268312 | -2204 | 0.14 | 0.51 | -0.38 |
| *SPRR2D* | NM_006945 | chr1 | 151280608 | 151281110 | -641 | 0.30 | 0.87 | -0.57 |
| *SPRR2E* | NM_001024209 | chr1 | 151334998 | 151335486 | -1617 | 0.05 | 0.47 | -0.42 |
| *SPRR2F* | NM_001014450 | chr1 | 151353459 | 151353966 | -1099 | 0.52 | 0.82 | -0.30 |
| *SPRR2G* | NM_001014291 | chr1 | 151391001 | 151391508 | -1203 | 0.14 | 0.54 | -0.41 |
| *SPRR3* | NM_001097589 | chr1 | 151239479 | 151239956 | -1128 | 0.54 | 0.87 | -0.32 |
| *SPRR4* | NM_173080 | chr1 | 151207313 | 151207792 | -2198 | 0.29 | 0.64 | -0.35 |
| *SPRYD5* | NM_032681 | chr11 | 55405496 | 55406012 | -1594 | 0.09 | 0.41 | -0.32 |
| *SPTB* | NM_000347 | chr14 | 64360477 | 64360990 | -1114 | 0.30 | 0.62 | -0.32 |
| *SPTLC3* | NM_018327 | chr20 | 12936749 | 12937243 | -630 | 0.35 | 0.74 | -0.38 |
| *SSX6* | NM_173357 | chrX | 47849593 | 47850137 | -2166 | 0.51 | 0.82 | -0.31 |
| *SSX8* | NR_027250 | chrX | 52666271 | 52666712 | -2217 | 0.43 | 0.83 | -0.40 |
| *ST18* | NM_014682 | chr8 | 53485940 | 53486444 | -1200 | 0.24 | 0.56 | -0.32 |
| *STAC* | NM_003149 | chr3 | 36394666 | 36395117 | -2208 | 0.19 | 0.49 | -0.30 |
| *STK33* | NM_030906 | chr11 | 8573983 | 8574500 | -2162 | 0.15 | 0.69 | -0.54 |
| *STRC* | NM_153700 | chr15 | 41698754 | 41699030 | -602 | 0.26 | 0.72 | -0.45 |
| *STX7* | NM_003569 | chr6 | 132877881 | 132878398 | -2109 | 0.11 | 0.57 | -0.46 |
| *SULF1* | NM_001128204 | chr8 | 70538997 | 70539546 | -2140 | 0.52 | 0.83 | -0.30 |
| *SV2A* | NM_014849 | chr1 | 148156411 | 148156899 | -597 | 0.25 | 0.57 | -0.31 |
| *SVOP* | NM_018711 | chr12 | 107944973 | 107945472 | -1994 | 0.18 | 0.58 | -0.41 |
| *SYCP2L* | NM_001040274 | chr6 | 10994399 | 10994624 | -537 | 0.14 | 0.48 | -0.34 |
| *SYK* | NR_024155 | chr9 | 92629095 | 92629616 | -169 | 0.47 | 0.79 | -0.32 |
| *SYNPR* | NM_144642 | chr3 | 63402821 | 63403369 | -697 | 0.45 | 0.79 | -0.34 |
| *SYTL2* | NM_032943 | chr11 | 85146652 | 85147168 | -218 | 0.39 | 0.87 | -0.47 |
| *SYTL3* | NM_001009991 | chr6 | 158989639 | 158990178 | -1124 | 0.59 | 0.89 | -0.31 |
| *TAAR5* | NM_003967 | chr6 | 132953981 | 132954462 | -1651 | 0.25 | 0.63 | -0.38 |
| *TAAR8* | NM_053278 | chr6 | 132913543 | 132914050 | -1727 | 0.22 | 0.65 | -0.43 |
| *TAAR9* | NM_175057 | chr6 | 132899183 | 132899685 | -1685 | 0.06 | 0.74 | -0.68 |
| *TAS2R16* | NM_016945 | chr7 | 122423454 | 122423987 | -730 | 0.10 | 0.41 | -0.31 |
| *TAS2R38* | NM_176817 | chr7 | 141320985 | 141321511 | -1206 | 0.41 | 0.80 | -0.38 |
| *TAS2R40* | NM_176882 | chr7 | 142626857 | 142627233 | -2248 | 0.24 | 0.56 | -0.32 |
| *TAS2R60* | NM_177437 | chr7 | 142849288 | 142849804 | -1121 | 0.63 | 0.96 | -0.33 |
| *TBRG1* | NM_032811 | chr11 | 123995560 | 123996096 | -2123 | 0.15 | 0.66 | -0.50 |
| *TBXAS1* | NM_001061 | chr7 | 139173062 | 139173511 | -2204 | 0.17 | 0.52 | -0.35 |
| *TCP11* | NM_001093728 | chr6 | 35219310 | 35219491 | -2235 | 0.47 | 0.80 | -0.33 |
| *TEKT3* | NM_031898 | chr17 | 15187073 | 15187570 | -1638 | 0.20 | 0.77 | -0.57 |
| *TEX10* | NM_017746 | chr9 | 102156851 | 102157264 | -2062 | 0.10 | 0.44 | -0.33 |
| *TEX101* | NM_031451 | chr19 | 48583229 | 48583726 | -1124 | 0.42 | 0.85 | -0.43 |
| *TG* | NM_003235 | chr8 | 133946874 | 133947344 | -1277 | 0.08 | 0.71 | -0.63 |
| *TGIF2LX* | NM_138960 | chrX | 89061161 | 89061628 | -2200 | 0.16 | 0.46 | -0.30 |
| *TGM6* | NM_198994 | chr20 | 2308224 | 2308741 | -1070 | 0.33 | 0.68 | -0.35 |
| *THSD7B* | NM_001080427 | chr2 | 137463487 | 137464035 | -1170 | 0.30 | 0.78 | -0.48 |
| *TM7SF4* | NM_030788 | chr8 | 105420809 | 105421294 | -177 | 0.47 | 0.77 | -0.30 |
| *TMCO5A* | NM_152453 | chr15 | 36014441 | 36014912 | -71 | 0.13 | 0.52 | -0.38 |
| *TMEM100* | NM_018286 | chr17 | 51156564 | 51157063 | -1589 | 0.15 | 0.59 | -0.44 |
| *TMEM110* | NM_198563 | chr3 | 52907934 | 52908438 | -1599 | 0.20 | 0.59 | -0.40 |
| *TMEM128* | NM_032927 | chr4 | 4301698 | 4302086 | -1057 | 0.36 | 0.67 | -0.32 |
| *TMEM132C* | NM_001136103 | chr12 | 127464448 | 127465003 | -517 | 0.10 | 0.45 | -0.34 |
| *TMEM133* | NM_032021 | chr11 | 100366043 | 100366574 | -1711 | 0.18 | 0.64 | -0.46 |
| *TMEM136* | NM_174926 | chr11 | 119699309 | 119699808 | -1666 | 0.24 | 0.69 | -0.45 |
| *TMEM14E* | NM_001123228 | chr3 | 153542343 | 153542849 | -1127 | 0.32 | 0.77 | -0.45 |
| *TMEM174* | NM_153217 | chr5 | 72502888 | 72503402 | -1633 | 0.34 | 0.77 | -0.42 |
| *TMEM5* | NM_014254 | chr12 | 62458021 | 62458527 | -1629 | 0.27 | 0.64 | -0.37 |
| *TMEM71* | NM_001145153 | chr8 | 133842962 | 133843460 | -1115 | 0.23 | 0.55 | -0.32 |
| *TMEM74* | NM_153015 | chr8 | 109871019 | 109871411 | -2269 | 0.20 | 0.56 | -0.36 |
| *TMPRSS5* | NM_030770 | chr11 | 113083597 | 113084133 | -1587 | 0.30 | 0.74 | -0.43 |
| *TMSB15A* | NM_021992 | chrX | 101659815 | 101660195 | -1650 | 0.47 | 0.83 | -0.36 |
| *TNC* | NM_002160 | chr9 | 116922664 | 116922782 | -2416 | 0.10 | 0.43 | -0.33 |
| *TNFSF9* | NM_003811 | chr19 | 6480293 | 6480743 | -1491 | 0.38 | 0.77 | -0.39 |
| *TNR* | NM_003285 | chr1 | 173979881 | 173980373 | -752 | 0.53 | 0.84 | -0.31 |
| *TPO* | NM_000547 | chr2 | 1395762 | 1396264 | -228 | 0.33 | 0.75 | -0.42 |
| *TRAT1* | NM_016388 | chr3 | 110022433 | 110022979 | -1614 | 0.52 | 0.82 | -0.30 |
| *TREML2* | NM_024807 | chr6 | 41278104 | 41278627 | -1462 | 0.19 | 0.74 | -0.54 |
| *TREML4* | NM_198153 | chr6 | 41301602 | 41302079 | -2198 | 0.24 | 0.54 | -0.30 |
| *TRHR* | NM_003301 | chr8 | 110167735 | 110168262 | -902 | 0.43 | 0.75 | -0.33 |
| *TRIM43* | NM_138800 | chr2 | 95619077 | 95619603 | -2152 | 0.55 | 0.86 | -0.31 |
| *TRIM48* | NM_024114 | chr11 | 54784299 | 54784796 | -1685 | 0.24 | 0.58 | -0.35 |
| *TRIM53* | NM_001145126 | chr11 | 89212433 | 89212895 | -2209 | 0.22 | 0.74 | -0.52 |
| *TRIM55* | NM_033058 | chr8 | 67201496 | 67201976 | -95 | 0.28 | 0.66 | -0.38 |
| *TRIM64* | NM_001136486 | chr11 | 89340922 | 89341429 | -143 | 0.20 | 0.61 | -0.41 |
| *TRIM77* | NM_001146162 | chr11 | 89082192 | 89082705 | -665 | 0.10 | 0.49 | -0.39 |
| *TRIML1* | NM_178556 | chr4 | 189296601 | 189297107 | -737 | 0.29 | 0.78 | -0.49 |
| *TRIMP1* | NR_002777 | chr11 | 5620532 | 5621007 | -217 | 0.20 | 0.51 | -0.31 |
| *TRPC7* | NM_020389 | chr5 | 135720728 | 135721218 | -1 | 0.37 | 0.85 | -0.48 |
| *TRPM3* | NM_001007470 | chr9 | 72674647 | 72675181 | -1120 | 0.21 | 0.51 | -0.30 |
| *TRPM4* | NM_017636 | chr19 | 54351814 | 54352362 | -775 | 0.17 | 0.60 | -0.43 |
| *TSGA13* | NM_052933 | chr7 | 130022695 | 130023197 | -1000 | 0.08 | 0.44 | -0.36 |
| *TSHB* | NM_000549 | chr1 | 115371992 | 115372497 | -1692 | 0.45 | 0.86 | -0.40 |
| *TSPAN19* | NM_001100917 | chr12 | 83955496 | 83955966 | -1545 | 0.49 | 0.90 | -0.41 |
| *TTC18* | NM_145170 | chr10 | 74790089 | 74790612 | -1732 | 0.25 | 0.61 | -0.35 |
| *TTC30B* | NM_152517 | chr2 | 178127214 | 178127737 | -1705 | 0.26 | 0.61 | -0.35 |
| *TTLL8* | NM_001080447 | chr22 | 48835661 | 48835860 | -578 | 0.27 | 0.62 | -0.35 |
| *TTPA* | NM_000370 | chr8 | 64162630 | 64163151 | -1724 | 0.26 | 0.60 | -0.35 |
| *TTTY5* | NR_001541 | chrY | 22855138 | 22855581 | -948 | 0.21 | 0.76 | -0.54 |
| *TTTY7* | NR_001534 | chrY | 10163718 | 10164215 | -1095 | 0.41 | 0.87 | -0.46 |
| *TUBB4Q* | NM_020040 | chr4 | 191143655 | 191144145 | -882 | 0.13 | 0.43 | -0.30 |
| *TUBB8* | NM_177987 | chr10 | 86474 | 86894 | -1506 | 0.13 | 0.49 | -0.36 |
| *TXNDC8* | NM_001003936 | chr9 | 112140297 | 112140814 | -609 | 0.35 | 0.69 | -0.35 |
| *UBE2MP1* | NR_002837 | chr16 | 34262614 | 34263112 | -600 | 0.29 | 0.64 | -0.35 |
| *UBTFL2* | NM_001101321 | chr11 | 89126702 | 89127213 | -166 | 0.16 | 0.48 | -0.32 |
| *UGT2A2* | NM_001105677 | chr4 | 70539876 | 70540389 | -209 | 0.24 | 0.58 | -0.34 |
| *UNC13C* | NM_001080534 | chr15 | 52090502 | 52091032 | -1625 | 0.16 | 0.56 | -0.41 |
| *UPK1B* | NM_006952 | chr3 | 120374667 | 120375196 | -182 | 0.56 | 0.89 | -0.34 |
| *USH2A* | NM_007123 | chr1 | 214665358 | 214665836 | -2236 | 0.14 | 0.50 | -0.36 |
| *USP40* | NM_018218 | chr2 | 234136751 | 234137019 | -2279 | 0.33 | 0.65 | -0.32 |
| *USP6NL* | NM_001080491 | chr10 | 11615140 | 11615623 | -1101 | 0.28 | 0.60 | -0.32 |
| *VAC14* | NM_018052 | chr16 | 69393413 | 69393918 | -1103 | 0.42 | 0.74 | -0.32 |
| *VCX2* | NM_016378 | chrX | 8100679 | 8101169 | -1616 | 0.44 | 0.75 | -0.31 |
| *VCX3A* | NM_016379 | chrX | 6464521 | 6465028 | -1615 | 0.17 | 0.48 | -0.30 |
| *VEPH1* | NM_024621 | chr3 | 158700901 | 158701376 | -1101 | 0.06 | 0.38 | -0.32 |
| *VPS13C* | NM_001018088 | chr15 | 60141933 | 60142335 | -2195 | 0.24 | 0.69 | -0.45 |
| *VPS8* | NM_001009921 | chr3 | 186010195 | 186010663 | -2195 | 0.15 | 0.49 | -0.34 |
| *VSTM1* | NM_198481 | chr19 | 59260159 | 59260678 | -1399 | 0.57 | 0.87 | -0.30 |
| *VSTM2A* | NM_182546 | chr7 | 54575072 | 54575543 | -2204 | 0.43 | 0.74 | -0.31 |
| *WBP11P1* | NR_003558 | chr18 | 28343648 | 28344148 | -1725 | 0.18 | 0.48 | -0.30 |
| *WDR42B* | NM_001017930 | chrX | 27911206 | 27911704 | -1968 | 0.29 | 0.70 | -0.41 |
| *WEE2* | NM_001105558 | chr7 | 141052203 | 141052632 | -2203 | 0.38 | 0.71 | -0.34 |
| *WFDC10B* | NM_172006 | chr20 | 43767435 | 43767954 | -622 | 0.10 | 0.41 | -0.31 |
| *WFDC12* | NM_080869 | chr20 | 43188493 | 43188994 | -2223 | 0.26 | 0.74 | -0.48 |
| *WFDC6* | NM_080827 | chr20 | 43601900 | 43602412 | -608 | 0.25 | 0.74 | -0.49 |
| *WIF1* | NM_007191 | chr12 | 63803301 | 63803799 | -2167 | 0.16 | 0.55 | -0.39 |
| *WIPF3* | NM_001080529 | chr7 | 29838438 | 29838904 | -2194 | 0.16 | 0.53 | -0.37 |
| *WNT8B* | NM_003393 | chr10 | 102210859 | 102211356 | -1693 | 0.42 | 0.74 | -0.31 |
| *WSCD2* | NM_014653 | chr12 | 107045749 | 107046261 | -1635 | 0.34 | 0.70 | -0.36 |
| *XCL1* | NM_002995 | chr1 | 166811122 | 166811637 | -1099 | 0.31 | 0.77 | -0.45 |
| *XCL2* | NM_003175 | chr1 | 166780324 | 166780852 | -729 | 0.45 | 0.82 | -0.37 |
| *XIRP2* | NM_001079810 | chr2 | 167466860 | 167467358 | -1111 | 0.29 | 0.66 | -0.36 |
| *XKR3* | NM_175878 | chr22 | 15683770 | 15684310 | -1456 | 0.42 | 0.76 | -0.35 |
| *XKR9* | NM_001011720 | chr8 | 71742273 | 71742773 | -1630 | 0.36 | 0.69 | -0.33 |
| *XPA* | NM_000380 | chr9 | 99499959 | 99500491 | -713 | 0.16 | 0.51 | -0.35 |
| *XRCC6BP1* | NM_033276 | chr12 | 56620279 | 56620777 | -1183 | 0.11 | 0.52 | -0.42 |
| *ZCCHC5* | NM_152694 | chrX | 77803399 | 77803873 | -2155 | 0.10 | 0.44 | -0.34 |
| *ZCWPW2* | NM_001040432 | chr3 | 28404550 | 28405036 | -2197 | 0.32 | 0.62 | -0.30 |
| *ZFP112* | NM_001083335 | chr19 | 49554216 | 49554699 | -1761 | 0.40 | 0.73 | -0.33 |
| *ZNF197* | NM_001024855 | chr3 | 44640889 | 44641405 | -367 | 0.11 | 0.43 | -0.32 |
| *ZNF219* | NM_001101672 | chr14 | 20639019 | 20639421 | -2207 | 0.22 | 0.58 | -0.35 |
| *ZNF221* | NM_013359 | chr19 | 49145368 | 49145562 | -1771 | 0.05 | 0.37 | -0.32 |
| *ZNF227* | NM_182490 | chr19 | 49406865 | 49407398 | -1398 | 0.45 | 0.76 | -0.31 |
| *ZNF239* | NM_005674 | chr10 | 43386208 | 43386332 | -2357 | 0.09 | 0.65 | -0.55 |
| *ZNF322B* | NM_199005 | chr9 | 99003601 | 99003824 | -1981 | 0.21 | 0.67 | -0.46 |
| *ZNF354A* | NM_005649 | chr5 | 178092666 | 178092783 | -2415 | 0.11 | 0.48 | -0.38 |
| *ZNF385B* | NM_152520 | chr2 | 180436419 | 180436922 | -2193 | 0.26 | 0.64 | -0.38 |
| *ZNF397* | NM_001135178 | chr18 | 31072580 | 31073121 | -2144 | 0.33 | 0.67 | -0.33 |
| *ZNF479* | NM_033273 | chr7 | 57213860 | 57213981 | -2407 | 0.14 | 0.46 | -0.33 |
| *ZNF485* | NM_145312 | chr10 | 43420894 | 43421396 | -715 | 0.48 | 0.83 | -0.35 |
| *ZNF501* | NM_145044 | chr3 | 44744310 | 44744506 | -1719 | 0.27 | 0.59 | -0.33 |
| *ZNF548* | NM_152909 | chr19 | 62590596 | 62591138 | -2162 | 0.07 | 0.45 | -0.37 |
| *ZNF556* | NM_024967 | chr19 | 2816372 | 2816863 | -1714 | 0.36 | 0.72 | -0.36 |
| *ZNF610* | NM_173530 | chr19 | 57540310 | 57540703 | 13 | 0.30 | 0.70 | -0.40 |
| *ZNF614* | NM_025040 | chr19 | 57225341 | 57225844 | -2100 | 0.47 | 0.80 | -0.33 |
| *ZNF662* | NM_001134656 | chr3 | 42919965 | 42920436 | -2460 | 0.32 | 0.73 | -0.42 |
| *ZNF705A* | NM_001004328 | chr12 | 8216022 | 8216472 | -169 | 0.34 | 0.66 | -0.31 |
| *ZNF782* | NM_001001662 | chr9 | 98658529 | 98658637 | -2373 | 0.07 | 0.40 | -0.34 |
| *ZNF805* | NM_001023563 | chr19 | 62441993 | 62442177 | -1779 | 0.24 | 0.64 | -0.40 |
| *ZNF81* | NM_007137 | chrX | 47578805 | 47579277 | -2203 | 0.16 | 0.46 | -0.30 |
| *ZPBP* | NM_007009 | chr7 | 50104827 | 50105316 | -1699 | 0.35 | 0.66 | -0.31 |
| *ZSCAN4* | NM_152677 | chr19 | 62870160 | 62870655 | -1706 | 0.32 | 0.84 | -0.52 |
| *ZSCAN5B* | NM_001080456 | chr19 | 61396097 | 61396595 | -113 | 0.44 | 0.75 | -0.32 |
| *ZSWIM2* | NM_182521 | chr2 | 187422481 | 187422953 | -575 | 0.29 | 0.61 | -0.33 |
