# Supplementary material for: DNA methylation and gene expression profiles show novel regulatory pathways in hepatocellular carcinoma
Source: Clin Epigenetics. 2015 Apr 14;7(1):43. doi: 10.1186/s13148-015-0077-1 (PMC4419480; doi:10.1186/s13148-015-0077-1)
Supplement: Additional file 3: Table S3. — Repressed genes in HCC tissue: 1,524 transcripts corresponding to 1,004 repressed genes in HCC as compared to cancer-free tissue (p.value.adj < 0.05). [file 13148_2015_77_MOESM3_ESM.doc]

| **Table S3 - REPRESSED GENES IN HCC TISSUE**  1524 transcripts corresponding to 1004 repressed genes in HCC tissue (p.value.adj<0.05) | | | |
| --- | --- | --- | --- |
| **Gene symbol** | **Gene ID** | **GenBank accession number** | **log2 fold change** |
| *AADAT* | 51166 | NM_016228 | -2.493 |
| *AASS* | 10157 | NM_005763 | -1.945 |
| *ABAT* | 18 | BC015628 | -1.332 |
| *ABAT* | 18 | NM_000663 | -1.068 |
| *ABCA8* | 10351 | NM_007168 | -1.839 |
| *ABCB11* | 8647 | NM_003742 | -1.275 |
| *ABCC6* | 368 | NM_001171 | -1.172 |
| *ABCC9* | 10060 | NM_005691 | -2.229 |
| *ABCC9* | 10060 | NM_020297 | -2.124 |
| *ABCG2* | 9429 | BC021281 | -1.78 |
| *ABCG2* | 9429 | NM_004827 | -1.472 |
| *ABCG2* | 9429 | AK002040 | -1.717 |
| *ABHD2* | 11057 | BC019248 | -1.06 |
| *ABHD2* | 11057 | BC017905 | -1.539 |
| *ABI3BP* | 25890 | NM_015429 | -1.354 |
| *ABP1* | 26 | BC014093 | -2.102 |
| *ABP1* | 26 | U11863 | -2.158 |
| *ABP1* | 26 | NM_001091 | -2.213 |
| *ACAD11* | 84129 | AJ608287 | -1.118 |
| *ACAD11* | 84129 | NM_032169 | -1.054 |
| *ACADL* | 33 | BC064549 | -1.974 |
| *ACADL* | 33 | NM_001608 | -1.679 |
| *ACADM* | 34 | AF251043 | -1.323 |
| *ACADM* | 34 | NM_000016 | -1.089 |
| *ACADS* | 35 | NM_000017 | -1.338 |
| *ACADS* | 35 | BC025963 | -1.068 |
| *ACADSB* | 36 | BC013756 | -1.972 |
| *ACADSB* | 36 | NM_001609 | -1.165 |
| *ACF* | 29974 | BC054873 | -1.073 |
| *ACMSD* | 130013 | NM_138326 | -1.011 |
| *ACMSD* | 130013 | BC107420 | -1.037 |
| *ACOT11* | 26027 | NM_147161 | -1.067 |
| *ACOX1* | 51 | BC010425 | -1.033 |
| *ACOX1* | 51 | NM_004035 | -1.654 |
| *ACSL1* | 2180 | BC050073 | -1.918 |
| *ACSL5* | 51703 | BC007985 | -1.031 |
| *ACSL5* | 51703 | NM_016234 | -1.03 |
| *ACSM3* | 6296 | NM_005622 | -2.078 |
| *ACSM3* | 6296 | AK223139 | -1.887 |
| *ACSM3* | 6296 | NM_202000 | -1.867 |
| *ADAMTS13* | 11093 | NM_139028 | -2.044 |
| *ADAMTS13* | 11093 | NM_139026 | -2.111 |
| *ADAMTS13* | 11093 | NM_139025 | -2.099 |
| *ADAMTS2* | 9509 | NM_021599 | -1.639 |
| *ADAMTSL2* | 9719 | NM_014694 | -2.129 |
| *ADAMTSL3* | 57188 | NM_207517 | -1.868 |
| *ADH1A* | 125 | NM_000667 | -1.358 |
| *ADH1B* | 125 | NM_000668 | -1.358 |
| *ADH6* | 130 | BC039065 | -2.018 |
| *ADH6* | 130 | NM_000672 | -1.865 |
| *ADK* | 132 | BC003568 | -1.046 |
| *ADMR* | 11318 | NM_007264 | -2.498 |
| *ADORA3* | 140 | BC064411 | -1.918 |
| *ADORA3* | 140 | NM_020683 | -1.882 |
| *ADRA1A* | 148 | NM_033303 | -3.847 |
| *ADRA1A* | 148 | NM_000680 | -3.595 |
| *ADRA1A* | 148 | BC095512 | -3.346 |
| *ADRA1A* | 148 | NM_033302 | -3.325 |
| *ADRA1A* | 148 | NM_033304 | -3.825 |
| *ADRA1B* | 147 | NM_000679 | -1.618 |
| *ADRA2B* | 151 | NM_000682 | -1.548 |
| *ADRB2* | 154 | M15169 | -1.378 |
| *AGL* | 178 | NM_000028 | -1.534 |
| *AGL* | 178 | BC078663 | -1.702 |
| *AGTR1* | 185 | NM_000685 | -1.545 |
| *AGXT2L1* | 64850 | NM_031279 | -1.158 |
| *AK3* | 50808 | BC013771 | -1.072 |
| *AKR1D1* | 6718 | NM_005989 | -3.48 |
| *AKR1D1* | 6718 | AK058142 | -2.714 |
| *ALAD* | 210 | NM_000031 | -1.068 |
| *ALDH1A3* | 220 | NM_000693 | -1.722 |
| *ALDH3A2* | 224 | BC002430 | -1.171 |
| *ALDH8A1* | 64577 | BC113862 | -1.491 |
| *ALDH8A1* | 64577 | NM_022568 | -1.474 |
| *ALLC* | 55821 | NM_018436 | -1.847 |
| *ALLC* | 55821 | NM_199232 | -1.113 |
| *ALPL* | 249 | AB209814 | -1.559 |
| *ALPL* | 249 | NM_000478 | -1.403 |
| *ALS2* | 57679 | BC071576 | -1.425 |
| *ALS2* | 57679 | NM_020919 | -1.028 |
| *AMDHD1* | 144193 | BC029146 | -1.092 |
| *AMHR2* | 269 | NM_020547 | -1.393 |
| *AMOTL1* | 154810 | BC037539 | -1.8 |
| *AMOTL1* | 154810 | AK093699 | -1.24 |
| *AMOTL1* | 154810 | NM_130847 | -1.57 |
| *AMOTL2* | 51421 | AK223598 | -1.324 |
| *ANGPTL1* | 9068 | BC050640 | -3.071 |
| *ANGPTL4* | 51129 | NM_001039667 | -1.054 |
| *ANGPTL6* | 83854 | NM_031917 | -2.377 |
| *ANK2* | 287 | NM_001148 | -1.048 |
| *ANK3* | 288 | AK126851 | -1.925 |
| *ANK3* | 288 | NM_001149 | -1.815 |
| *ANKRD38* | 163782 | AK091941 | -1.424 |
| *ANTXR2* | 118429 | BC107876 | -1.358 |
| *ANTXR2* | 118429 | AK055636 | -1.172 |
| *ANTXR2* | 118429 | NM_058172 | -1.351 |
| *ANUBL1* | 93550 | BC017591 | -1.171 |
| *ANXA10* | 11199 | NM_007193 | -2.176 |
| *ANXA10* | 11199 | AY626137 | -1.788 |
| *APBA1* | 320 | NM_001163 | -2.126 |
| *APBB2* | 323 | NM_173075 | -1.192 |
| *APOA5* | 116519 | BC101787 | -1.718 |
| *APOF* | 319 | NM_001638 | -1.269 |
| *APOL6* | 80830 | AK097266 | -1.611 |
| *APOL6* | 80830 | NM_030641 | -1.53 |
| *APOL6* | 80830 | BC038950 | -1.288 |
| *AQP3* | 360 | NM_004925 | -1.401 |
| *AQP4* | 361 | NM_001650 | -1.125 |
| *AQP7* | 364 | BC062701 | -1.017 |
| *AQP9* | 366 | BC026258 | -1.666 |
| *AQP9* | 366 | NM_020980 | -1.418 |
| *AR* | 367 | NM_000044 | -1.38 |
| *AR* | 367 | M34233 | -1.324 |
| *AR* | 367 | M21748 | -1.29 |
| *ARHGAP10* | 79658 | NM_024605 | -1.06 |
| *ARHGAP10* | 79658 | AK024549 | -1.027 |
| *ARHGAP20* | 57569 | AY496266 | -1.647 |
| *ARHGAP20* | 57569 | NM_020809 | -1.338 |
| *ARHGAP29* | 9411 | BC093741 | -1.029 |
| *ARHGEF12* | 23365 | NM_015313 | -1.126 |
| *ARL6IP* | 23204 | NM_015161 | -1.092 |
| *ARMETL1* | 441549 | NM_001029954 | -1.054 |
| *ARSD* | 414 | BC020229 | -1.355 |
| *ARSF* | 416 | NM_004042 | -1.52 |
| *ASAHL* | 27163 | NM_014435 | -1.414 |
| *ASPA* | 443 | NM_000049 | -2.252 |
| *ATF5* | 22809 | NM_012068 | -1.643 |
| *ATF5* | 22809 | BC005174 | -1.789 |
| *ATOH7* | 220202 | NM_145178 | -1.968 |
| *ATOH8* | 84913 | BC094832 | -2.286 |
| *ATOH8* | 84913 | NM_032827 | -2.311 |
| *ATP11C* | 286410 | NM_001010986 | -1.023 |
| *ATP13A3* | 79572 | XM_927225 | -1.122 |
| *ATRNL1* | 26033 | NM_207303 | -1.178 |
| *AXL* | 558 | NM_001699 | -1.518 |
| *AXUD1* | 64651 | NM_033027 | -2.035 |
| *B3GAT1* | 27087 | NM_018644 | -4.384 |
| *bA16L21.2.1* | 548645 | BC048318 | -1.171 |
| *bA16L21.2.1* | 548645 | NM_001015882 | -1.205 |
| *BACH2* | 60468 | NM_021813 | -1.523 |
| *BBOX1* | 8424 | BC011034 | -2.735 |
| *BBOX1* | 8424 | NM_003986 | -2.749 |
| *BCDO2* | 83875 | BC041656 | -3.612 |
| *BCDO2* | 83875 | AK091783 | -2.944 |
| *BCDO2* | 83875 | AJ290393 | -3.061 |
| *BCHE* | 590 | NM_000055 | -1.795 |
| *BCKDHB* | 594 | NM_000056 | -1.438 |
| *BCKDHB* | 594 | NM_183050 | -1.571 |
| *BCKDHB* | 594 | BC034481 | -1.365 |
| *BDH2* | 56898 | BC037277 | -1.737 |
| *BDH2* | 56898 | BC001953 | -1.219 |
| *BHMT* | 635 | NM_001713 | -1.11 |
| *BLNK* | 29760 | AM180330 | -1.038 |
| *BMP6* | 654 | NM_001718 | -1.69 |
| *BMPER* | 168667 | BC060868 | -4.407 |
| *BRP44L* | 51660 | CR621401 | -1.226 |
| *BRP44L* | 51660 | BC000810 | -1.193 |
| *BRP44L* | 51660 | NM_016098 | -1.156 |
| *BRWD1* | 54014 | NM_001007246 | -1.365 |
| *BST1* | 683 | NM_004334 | -1.177 |
| *BZRAP1* | 9256 | NM_004758 | -1.041 |
| *BZW1* | 9689 | NM_014670 | -1.045 |
| *BZW1* | 9689 | BC026303 | -1.234 |
| *C10orf104* | 119504 | NM_173473 | -1.106 |
| *C10orf26* | 54838 | AK000374 | -1.329 |
| *C10orf33* | 84795 | NM_032709 | -2.132 |
| *C10orf38* | 221061 | BC109302 | -2.158 |
| *C10orf57* | 80195 | NM_025125 | -1.15 |
| *C10orf65* | 112817 | NM_138413 | -1.825 |
| *C10orf65* | 112817 | BC057821 | -1.751 |
| *C10orf65* | 112817 | AK094791 | -1.112 |
| *C10orf83* | 118812 | NM_178832 | -1.181 |
| *C10orf97* | 80013 | BC020605 | -1.032 |
| *C11orf43* | 492304 | BC042127 | -4.416 |
| *C11orf43* | 492304 | BC011786 | -4.36 |
| *C11orf43* | 492304 | NM_001007139 | -3.839 |
| *C11orf54* | 28970 | AL136605 | -1.248 |
| *C11orf54* | 28970 | BC046477 | -1.368 |
| *C11orf54* | 28970 | NM_014039 | -1.34 |
| *C14orf105* | 55195 | NM_018168 | -1.286 |
| *C14orf140* | 79696 | BC004259 | -1.477 |
| *C14orf37* | 145407 | BX161448 | -1.648 |
| *C14orf68* | 283600 | AB073385 | -2.376 |
| *C14orf68* | 283600 | NM_207117 | -2.029 |
| *C14orf8* | 122664 | NM_173846 | -2.309 |
| *C16orf45* | 89927 | NM_033201 | -1.32 |
| *C17orf51* | 339263 | XM_378661 | -1.042 |
| *C1orf126* | 200197 | NM_182534 | -2.409 |
| *C1orf168* | 199920 | AK125198 | -1.221 |
| *C1orf168* | 199920 | NM_001004303 | -1.526 |
| *C1orf179* | 338094 | NM_176782 | -2.847 |
| *C1orf179* | 338094 | BC015993 | -2.123 |
| *C1orf71* | 163882 | NM_152609 | -1.186 |
| *C1QTNF1* | 114897 | NM_030968 | -2.29 |
| *C1R* | 715 | XM_930681 | -1.181 |
| *C1RL* | 51279 | AK024084 | -1.491 |
| *C1RL* | 51279 | NM_016546 | -1.023 |
| *C20orf127* | 140851 | BC103840 | -1.896 |
| *C20orf127* | 140851 | NM_080757 | -1.682 |
| *C20orf132* | 140699 | NM_152503 | -2.264 |
| *C20orf132* | 140699 | BC030006 | -1.985 |
| *C20orf175* | 140876 | NM_080829 | -1.385 |
| *C20orf74* | 57186 | XM_046600 | -1.131 |
| *C21orf84* | 114038 | BC107109 | -1.559 |
| *C21orf91* | 54149 | CR749496 | -1.405 |
| *C4orf16* | 55435 | BC009485 | -1.236 |
| *C5AR1* | 728 | BC008982 | -1.13 |
| *C5orf23* | 79614 | NM_024563 | -1.211 |
| *C6orf208* | 80069 | BC101251 | -1.14 |
| *C6orf71* | 389434 | NM_203395 | -1.75 |
| *C6orf71* | 389434 | BC056253 | -1.345 |
| *C6orf71* | 389434 | AY957660 | -1.742 |
| *C8A* | 731 | NM_000562 | -1.511 |
| *C8B* | 732 | NM_000066 | -1.276 |
| *C8orf4* | 56892 | BC020623 | -2.598 |
| *C8orf4* | 56892 | NM_020130 | -2.229 |
| *C9orf103* | 414328 | NM_001001551 | -1.162 |
| *C9orf103* | 414328 | AY615889 | -1.082 |
| *C9orf111* | 375775 | NM_152286 | -1.423 |
| *C9orf150* | 286343 | NM_203403 | -1.258 |
| *C9orf26* | 90865 | NM_033439 | -2.015 |
| *C9orf52* | 158219 | BC038592 | -1.174 |
| *C9orf52* | 158219 | NM_152574 | -1.258 |
| *C9orf68* | 55064 | NM_001039395 | -1.889 |
| *C9orf72* | 203228 | BC068445 | -1.656 |
| *C9orf72* | 203228 | NM_018325 | -1.536 |
| *C9orf95* | 54981 | NM_017881 | -1.046 |
| *C9orf95* | 54981 | BC036804 | -1.033 |
| *CA13* | 377677 | NM_198584 | -1.239 |
| *CAMK2B* | 816 | NM_172084 | -1.884 |
| *CAMK2B* | 816 | NM_001220 | -2.206 |
| *CAMK2B* | 816 | NM_172078 | -2.204 |
| *CAMK2B* | 816 | NM_172080 | -2.403 |
| *CAMK2B* | 816 | NM_172081 | -2.346 |
| *CAMK2B* | 816 | NM_172082 | -2.514 |
| *CAMK2D* | 817 | NM_001221 | -1.812 |
| *CAND2* | 23066 | XM_371617 | -1.072 |
| *CARF* | 55602 | NM_017632 | -1.018 |
| *CARKL* | 23729 | BC020543 | -1.024 |
| *CASC4* | 113201 | NM_138423 | -1.117 |
| *CASP10* | 843 | NM_032974 | -1.062 |
| *CAT* | 847 | BC112217 | -1.069 |
| *CBR4* | 84869 | NM_032783 | -1.239 |
| *CBR4* | 84869 | BC021973 | -1.01 |
| *CBS* | 875 | BC007257 | -1.024 |
| *CCBE1* | 147372 | BC046645 | -3.381 |
| *CCBE1* | 147372 | NM_133459 | -3.132 |
| *CCDC68* | 80323 | NM_025214 | -1.633 |
| *CCL14* | 6358 | BC038289 | -1.458 |
| *CCNC* | 892 | NM_001013399 | -1.02 |
| *CCNC* | 892 | AK222768 | -1.114 |
| *CCNC* | 892 | BC010135 | -1.066 |
| *CCPG1* | 9236 | AK022459 | -1.342 |
| *CCR1* | 1230 | BC051306 | -1.721 |
| *CCR1* | 1230 | NM_001295 | -1.69 |
| *CCRN4L* | 25819 | NM_012118 | -1.03 |
| *CD14* | 929 | NM_000591 | -1.391 |
| *CD14* | 929 | NM_001040021 | -1.153 |
| *CD14* | 929 | BC010507 | -1.142 |
| *CD163* | 9332 | Z22968 | -1.253 |
| *CD163* | 9332 | NM_203416 | -1.244 |
| *CD1A* | 909 | NM_001763 | -1.259 |
| *CD1D* | 912 | NM_001766 | -1.704 |
| *CD1E* | 913 | NM_030893 | -1.254 |
| *CD209* | 30835 | AY042225 | -4.54 |
| *CD209* | 30835 | AY042222 | -2.157 |
| *CD244* | 51744 | AJ245377 | -1.744 |
| *CD244* | 51744 | AF242540 | -1.491 |
| *CD244* | 51744 | BC028073 | -1.121 |
| *CD244* | 51744 | NM_016382 | -1.123 |
| *CD300A* | 11314 | NM_007261 | -1.17 |
| *CD302* | 9936 | NM_014880 | -1.216 |
| *CD4* | 920 | BC025782 | -1.145 |
| *CD4* | 920 | NM_000616 | -1.23 |
| *CD5L* | 922 | NM_005894 | -2.203 |
| *CD5L* | 922 | BC033586 | -2.514 |
| *CD5L* | 922 | AY358494 | -2.145 |
| *CD82* | 3732 | NM_001024844 | -1.066 |
| *CDC14B* | 8555 | NM_033331 | -1.394 |
| *CDC14B* | 8555 | NM_003671 | -1.297 |
| *CDC14C* | 168448 | XM_498195 | -1.042 |
| *CDC37L1* | 55664 | NM_017913 | -1.144 |
| *CDH1* | 999 | AB025105 | -1.494 |
| *CDH1* | 999 | AB025106 | -1.407 |
| *CDH19* | 28513 | BC015877 | -1.226 |
| *CDH19* | 28513 | NM_021153 | -2.558 |
| *CDH23* | 64072 | NM_022124 | -2.805 |
| *CDH23* | 64072 | AY563165 | -2.919 |
| *CDH23* | 64072 | AB053445 | -2.49 |
| *CDH23* | 64072 | NM_052836 | -1.618 |
| *CDH23* | 64072 | BC065284 | -1.395 |
| *CDH23* | 64072 | AK093818 | -1.486 |
| *CDRT4* | 284040 | BC029542 | -1.326 |
| *CETP* | 1071 | BC025739 | -3.036 |
| *CETP* | 1071 | NM_000078 | -3.215 |
| *CFHR3* | 10878 | BC070259 | -1.76 |
| *CFHR4* | 10877 | AJ640130 | -1.017 |
| *CFI* | 3426 | BC020718 | -1.014 |
| *CFP* | 5199 | NM_002621 | -3.267 |
| *CG018* | 90634 | AL049786 | -1.942 |
| *CG018* | 90634 | NM_052818 | -2.13 |
| *CGN* | 57530 | NM_020770 | -1.048 |
| *CHN2* | 1124 | BC112155 | -1.451 |
| *CHPF* | 79586 | NM_024536 | -1.06 |
| *CHST4* | 10164 | NM_005769 | -2.72 |
| *CHST7* | 56548 | BC045537 | -1.131 |
| *CHST7* | 56548 | NM_019886 | -1.133 |
| *CHST9* | 83539 | NM_031422 | -1.504 |
| *CHST9* | 83539 | AY358488 | -1.492 |
| *CILP* | 8483 | NM_003613 | -2.122 |
| *CILP* | 8483 | BC035776 | -1.424 |
| *CISH* | 1154 | NM_145071 | -1.18 |
| *CISH* | 1154 | AF035947 | -1.142 |
| *CLDN1* | 9076 | BC012471 | -1 |
| *CLDN10* | 9071 | NM_006984 | -1.115 |
| *CLDN10* | 9071 | BC010920 | -2.065 |
| *CLEC1B* | 51266 | BC029554 | -5.174 |
| *CLEC1B* | 51266 | NM_016509 | -2.564 |
| *CLEC4G* | 339390 | BC093691 | -4.398 |
| *CLEC4G* | 339390 | NM_198492 | -3.435 |
| *CLEC4M* | 10332 | AY042235 | -4.162 |
| *CLEC4M* | 10332 | AF290887 | -4.792 |
| *CLEC4M* | 10332 | NM_214677 | -4.518 |
| *CLEC4M* | 10332 | AY343913 | -3.01 |
| *CLEC4M* | 10332 | NM_214675 | -5.216 |
| *CLEC4M* | 10332 | NM_014257 | -5.343 |
| *CLEC4M* | 10332 | BC110614 | -5.571 |
| *CLN5* | 1203 | NM_006493 | -1.13 |
| *CLN8* | 2055 | NM_018941 | -1.128 |
| *CLPX* | 10845 | AL136922 | -1.043 |
| *CMYA5* | 202333 | NM_153610 | -1.043 |
| *CMYA5* | 202333 | BC111529 | -1.079 |
| *CNDP1* | 84735 | NM_032649 | -5.463 |
| *CNDP1* | 84735 | BC110295 | -5.104 |
| *CNGA1* | 1259 | NM_000087 | -1.876 |
| *CNKSR1* | 10256 | NM_006314 | -2.465 |
| *CNTFR* | 1271 | NM_001842 | -2.097 |
| *CNTN3* | 5067 | NM_020872 | -1.604 |
| *CNTN4* | 152330 | NM_175607 | -1.248 |
| *COBLL1* | 22837 | NM_014900 | -1.717 |
| *COLEC10* | 10584 | NM_006438 | -3.455 |
| *COLEC10* | 10584 | BC103815 | -3.429 |
| *COQ10B* | 80219 | NM_025147 | -1.664 |
| *CPEB2* | 132864 | BC103940 | -1.145 |
| *CPEB3* | 22849 | BC036444 | -2.733 |
| *CPEB3* | 22849 | NM_014912 | -1.994 |
| *CPN1* | 1369 | NM_001308 | -1.055 |
| *CRHBP* | 1393 | NM_001882 | -4.992 |
| *CRYL1* | 51084 | NM_015974 | -1.045 |
| *CSF3R* | 1441 | NM_000760 | -1.238 |
| *CTBS* | 1486 | BC024007 | -1.589 |
| *CTH* | 1491 | NM_153742 | -2.453 |
| *CTH* | 1491 | NM_001902 | -2.629 |
| *CTNNA3* | 29119 | NM_013266 | -1.878 |
| *CTNNA3* | 29119 | BC065819 | -1.663 |
| *CTNNA3* | 29119 | BC022004 | -1.219 |
| *CX3CR1* | 1524 | NM_001337 | -1.599 |
| *CXCL12* | 6387 | DQ345517 | -2.743 |
| *CXCL12* | 6387 | NM_001033886 | -2.833 |
| *CXCL12* | 6387 | NM_199168 | -2.778 |
| *CXCL12* | 6387 | NM_000609 | -2.705 |
| *CXCL14* | 9547 | AF106911 | -3.366 |
| *CXCL14* | 9547 | NM_004887 | -4.125 |
| *CXCL14* | 9547 | BC003513 | -3.404 |
| *CXorf6* | 10046 | NM_005491 | -1.378 |
| *CYB5D1* | 124637 | AK057061 | -1.2 |
| *CYB5D2* | 124936 | BC020263 | -1.246 |
| *CYorf14* | 55410 | AF119903 | -1.694 |
| *CYP1A1* | 1543 | NM_000499 | -2.267 |
| *CYP1A1* | 1543 | AY310359 | -2.311 |
| *CYP1A2* | 1544 | BC067424 | -4.952 |
| *CYP1A2* | 1544 | NM_000761 | -3.408 |
| *CYP26A1* | 1592 | NM_000783 | -4.044 |
| *CYP2B6* | 1555 | BC067430 | -1.659 |
| *CYP2C8* | 1558 | NM_030878 | -1.302 |
| *CYP2C9* | 1559 | NM_000771 | -1.352 |
| *CYP39A1* | 51302 | NM_016593 | -3.167 |
| *CYP39A1* | 51302 | BC010358 | -3.1 |
| *CYP3A4* | 1576 | NM_017460 | -1.199 |
| *CYP3A43* | 64816 | AF280113 | -2.385 |
| *CYP3A43* | 64816 | NM_022820 | -2.175 |
| *CYP4A11* | 1579 | BC041158 | -1.709 |
| *CYP4A22* | 284541 | NM_001010969 | -1.067 |
| *CYP4F2* | 8529 | BC067437 | -1.452 |
| *CYP8B1* | 1582 | BC067441 | -2.713 |
| *DAK* | 26007 | NM_015533 | -1.396 |
| *DBH* | 1621 | NM_000787 | -2.685 |
| *DBT* | 1629 | BC016675 | -1.515 |
| *DBT* | 1629 | J03208 | -1.593 |
| *DBT* | 1629 | NM_001918 | -1.02 |
| *DCHS1* | 8642 | NM_003737 | -1.282 |
| *DCN* | 1634 | NM_133505 | -2.837 |
| *DCN* | 1634 | BC005322 | -2.982 |
| *DCN* | 1634 | NM_133506 | -2.986 |
| *DDX3Y* | 8653 | BC034942 | -1.423 |
| *DDX3Y* | 8653 | NM_004660 | -2.22 |
| *DEPDC7* | 91614 | NM_139160 | -1.574 |
| *DEPDC7* | 91614 | BC030970 | -1.443 |
| *DERA* | 51071 | NM_015954 | -1.096 |
| *DHODH* | 1723 | NM_001361 | -1.122 |
| *DHODH* | 1723 | NM_001025193 | -1.05 |
| *DHRS1* | 115817 | NM_138452 | -1.054 |
| *DHRS1* | 115817 | BC014057 | -1.055 |
| *DIRAS3* | 9077 | NM_004675 | -1.756 |
| *DIRAS3* | 9077 | BC005362 | -1.581 |
| *DKFZP564J102* | 25854 | NM_001006655 | -1.784 |
| *DKFZP586H2123* | 25891 | BC089434 | -2.165 |
| *DKFZP586H2123* | 25891 | NM_001001991 | -2.115 |
| *DLG2* | 1740 | NM_001364 | -1.222 |
| *DMD* | 1756 | X14298 | -1.425 |
| *DMD* | 1756 | NM_004019 | -1.225 |
| *DMD* | 1756 | NM_000109 | -1.116 |
| *DNAJB9* | 4189 | BC028912 | -1.009 |
| *DNAJC12* | 56521 | NM_201262 | -1.759 |
| *DNAJC12* | 56521 | BC017018 | -2.001 |
| *DNAJC12* | 56521 | NM_021800 | -1.935 |
| *DNALI1* | 7802 | NM_003462 | -1.772 |
| *DNALI1* | 7802 | BC046117 | -1.559 |
| *DNASE1L3* | 1776 | BC015831 | -2.949 |
| *DNMBP* | 23268 | NM_015221 | -1.441 |
| *DOCK8* | 81704 | NM_203447 | -1.427 |
| *DOPEY1* | 23033 | NM_015018 | -1.392 |
| *DPF3* | 8110 | AK126933 | -1.89 |
| *DPT* | 1805 | NM_001937 | -2.328 |
| *DSCR1* | 1827 | NM_004414 | -2.055 |
| *DUSP1* | 1843 | NM_004417 | -1.032 |
| *DUSP16* | 80824 | BC109234 | -1.403 |
| *DUSP16* | 80824 | AY038927 | -1.486 |
| *ECM1* | 1893 | NM_004425 | -2.555 |
| *ECM1* | 1893 | NM_022664 | -2.528 |
| *ECM2* | 1842 | BC105958 | -1.595 |
| *ECM2* | 1842 | BC036806 | -1.779 |
| *EDEM1* | 9695 | NM_014674 | -1.058 |
| *EDNRB* | 1910 | NM_003991 | -1.131 |
| *EDNRB* | 1910 | BC014472 | -1.284 |
| *EDNRB* | 1910 | AF114165 | -1.142 |
| *EFHA2* | 286097 | NM_181723 | -1.552 |
| *EFNB3* | 1949 | BC042944 | -1.969 |
| *EFNB3* | 1949 | NM_001406 | -1.975 |
| *EHD3* | 30845 | BC033100 | -2.652 |
| *EHD3* | 30845 | NM_014600 | -2.41 |
| *EHHADH* | 1962 | BC110460 | -1.449 |
| *EHHADH* | 1962 | NM_001966 | -1.403 |
| *EIF4E3* | 317649 | NM_173359 | -1.782 |
| *EIF5* | 1983 | NM_001969 | -1.919 |
| *EML1* | 2009 | NM_001008707 | -1.352 |
| *EML1* | 2009 | BC033043 | -1.318 |
| *EML1* | 2009 | BC032541 | -1.494 |
| *EMR1* | 2015 | AK131562 | -1.695 |
| *EMR1* | 2015 | NM_001974 | -1.535 |
| *EMR1* | 2015 | BC059395 | -1.314 |
| *ENO3* | 2027 | NM_053013 | -1.373 |
| *ENPP1* | 5167 | NM_006208 | -1.109 |
| *ENPP1* | 5167 | BX537580 | -1.177 |
| *ENTPD8* | 377841 | NM_198585 | -1.098 |
| *EPB41L3* | 23136 | BC008377 | -1.023 |
| *EPB41L4A* | 64097 | AB030240 | -1.582 |
| *EPB41L4A* | 64097 | NM_022140 | -1.574 |
| *EPB41L4B* | 54566 | AK000388 | -1.651 |
| *EPB41L4B* | 54566 | AB032179 | -1.844 |
| *EPB41L4B* | 54566 | NM_018424 | -1.649 |
| *EPB41L4B* | 54566 | AK025249 | -1.547 |
| *EPB41L5* | 57669 | BC032822 | -1.553 |
| *EPHA2* | 1969 | NM_004431 | -1.677 |
| *EPHB1* | 2047 | NM_004441 | -2.103 |
| *EPHB6* | 2051 | NM_004445 | -1.194 |
| *EPHX2* | 2053 | NM_001979 | -1.094 |
| *EPM2A* | 7957 | NM_001018041 | -1.419 |
| *ERG* | 2078 | BC040168 | -1.502 |
| *ERN1* | 2081 | NM_001433 | -1.228 |
| *ESR1* | 2099 | AF258450 | -2.612 |
| *ESR1* | 2099 | AF258449 | -2.299 |
| *ESR1* | 2099 | M69297 | -1.493 |
| *ESR1* | 2099 | NM_000125 | -2.969 |
| *ESRRG* | 2104 | NM_001438 | -1.315 |
| *ETFDH* | 2110 | NM_004453 | -1.839 |
| *ETFDH* | 2110 | BC011890 | -1.272 |
| *ETS2* | 2114 | BC017040 | -1.609 |
| *ETS2* | 2114 | NM_005239 | -1.342 |
| *EVC* | 2121 | NM_153717 | -1.635 |
| *EVC* | 2121 | NM_014556 | -1.447 |
| *EXOD1* | 112479 | AB040937 | -1.545 |
| *EXPH5* | 23086 | BC113119 | -2.438 |
| *EXPH5* | 23086 | NM_015065 | -2.277 |
| *EYA2* | 2139 | NM_005244 | -2.529 |
| *EYA2* | 2139 | AY705349 | -2.085 |
| *F3* | 2152 | BC011029 | -1.346 |
| *F3* | 2152 | NM_001993 | -1.296 |
| *F8* | 2157 | NM_000132 | -1.418 |
| *F9* | 2158 | BC109214 | -1.764 |
| *F9* | 2158 | NM_000133 | -1.024 |
| *FAM107A* | 11170 | AK055443 | -1.27 |
| *FAM107A* | 11170 | BC010561 | -1.161 |
| *FAM13A1* | 10144 | BC086875 | -1.684 |
| *FAM13A1* | 10144 | AB020721 | -1.784 |
| *FAM13A1* | 10144 | NM_001015045 | -1.577 |
| *FAM46C* | 54855 | NM_017709 | -1.27 |
| *FAM59A* | 64762 | AL834491 | -1.653 |
| *FAM59A* | 64762 | NM_022751 | -1.306 |
| *FAM82A* | 151393 | NM_144713 | -1.697 |
| *FAM82A* | 151393 | AK095462 | -1.588 |
| *FAM82A* | 151393 | AF435956 | -1.457 |
| *FAM83F* | 113828 | NM_138435 | -1.406 |
| *FAM83F* | 113828 | BC031099 | -1.205 |
| *FAM8A1* | 51439 | BC047881 | -1.6 |
| *FAM92A1* | 137392 | XM_931181 | -1.236 |
| *FAM99A* | 387742 | NM_001014374 | -1.752 |
| *FANCC* | 2176 | NM_000136 | -1.342 |
| *FARP2* | 9855 | BC021301 | -1.078 |
| *FAT4* | 79633 | NM_024582 | -1.69 |
| *FBLN5* | 10516 | BX248290 | -1.39 |
| *FBXL5* | 26234 | AF199420 | -1.135 |
| *FBXO21* | 23014 | AL136899 | -1.206 |
| *FBXO28* | 23219 | NM_015176 | -1.164 |
| *FBXO3* | 26273 | NM_033406 | -1.255 |
| *FBXO3* | 26273 | BC039291 | -1.02 |
| *FBXO34* | 55030 | BC109120 | -1.095 |
| *FCGR2B* | 2213 | M31933 | -1.436 |
| *FCGR2B* | 2213 | BC031992 | -1.172 |
| *FCGR2C* | 9103 | NM_001005411 | -1.154 |
| *FCN1* | 2219 | NM_002003 | -1.084 |
| *FCN1* | 2219 | BC020635 | -1.193 |
| *FCN2* | 2220 | NM_004108 | -3.733 |
| *FCN3* | 8547 | NM_003665 | -3.601 |
| *FCRL6* | 343413 | AK131201 | -1.094 |
| *FCRL6* | 343413 | AY212514 | -1.102 |
| *FES* | 2242 | NM_002005 | -1.459 |
| *FES* | 2242 | AY513657 | -1.349 |
| *FES* | 2242 | AY513656 | -1.574 |
| *FES* | 2242 | AY513655 | -1.674 |
| *FEZ1* | 9638 | NM_005103 | -1.604 |
| *FGD4* | 121512 | BC045552 | -1.141 |
| *FGD4* | 121512 | NM_139241 | -1.135 |
| *FGFR1OP2* | 26127 | NM_015633 | -1.297 |
| *FGFR2* | 2263 | NM_022973 | -2.002 |
| *FGFR2* | 2263 | NM_022971 | -1.668 |
| *FGFR2* | 2263 | BC039243 | -1.966 |
| *FHL1* | 2273 | AF063002 | -1.323 |
| *FLJ10847* | 55244 | BC058882 | -1.039 |
| *FLJ10847* | 55244 | AK222625 | -1.02 |
| *FLJ10847* | 55244 | NM_018242 | -1.239 |
| *FLJ10986* | 55277 | CR623798 | -1.68 |
| *FLJ11795* | 79722 | NM_001039935 | -1.638 |
| *FLJ12688* | 79613 | XM_928238 | -1.05 |
| *FLJ13391* | 84141 | BC016157 | -1.054 |
| *FLJ13391* | 84141 | NM_032181 | -1.044 |
| *FLJ14001* | 79730 | NM_024677 | -1.173 |
| *FLJ16237* | 392636 | BC108676 | -1.286 |
| *FLJ20152* | 54463 | NM_001034850 | -2.922 |
| *FLJ20152* | 54463 | BC073132 | -2.607 |
| *FLJ20581* | 54988 | NM_017888 | -1.01 |
| *FLJ20701* | 55022 | NM_017933 | -1.175 |
| *FLJ20701* | 55022 | AK096636 | -1.129 |
| *FLJ21106* | 80167 | NM_001039717 | -1.052 |
| *FLJ21106* | 80167 | NM_025097 | -1.106 |
| *FLJ21986* | 79974 | NM_024913 | -2.739 |
| *FLJ21986* | 79974 | BC030538 | -1.674 |
| *FLJ21986* | 79974 | AY358220 | -1.521 |
| *FLJ25422* | 202151 | NM_145000 | -2.518 |
| *FLJ25422* | 202151 | AK058151 | -1.576 |
| *FLJ25530* | 220296 | NM_152722 | -2.056 |
| *FLJ25530* | 220296 | AK122595 | -2.189 |
| *FLJ25996* | 401109 | NM_001001699 | -2.667 |
| *FLJ30046* | 122060 | AL834203 | -1.587 |
| *FLJ32028* | 201799 | BC060775 | -1.158 |
| *FLJ32312* | 150962 | BC101680 | -1.171 |
| *FLJ35740* | 253650 | NM_147195 | -1.029 |
| *FLJ36031* | 168455 | NM_175884 | -1.424 |
| *FLJ37464* | 283848 | NM_173815 | -1.269 |
| *FLJ37464* | 283848 | AK094783 | -1.243 |
| *FLJ38668* | 644903 | NM_001039776 | -1.384 |
| *FLJ42393* | 401105 | NM_207488 | -1.338 |
| *FLJ43806* | 399563 | NM_201628 | -1.977 |
| *FLJ90013* | 202018 | BC066899 | -1.381 |
| *FLJ90013* | 202018 | NM_153365 | -1.046 |
| *FMO3* | 2328 | NM_001002294 | -1.01 |
| *FMO5* | 2330 | NM_001461 | -1.279 |
| *FNDC3A* | 22862 | NM_014923 | -1.379 |
| *FOLH1* | 2346 | NM_004476 | -1.533 |
| *FOSB* | 2354 | NM_006732 | -2.547 |
| *FOSL1* | 8061 | NM_005438 | -1.246 |
| *FOXO1A* | 2308 | BC021981 | -1.007 |
| *FOXO1A* | 2308 | NM_002015 | -1.342 |
| *FOXP2* | 93986 | NM_014491 | -1.071 |
| *FPR1* | 2357 | BC005315 | -1.77 |
| *FPR1* | 2357 | NM_002029 | -1.768 |
| *FPRL1* | 2358 | BC029125 | -2.544 |
| *FPRL1* | 2358 | NM_001005738 | -2.198 |
| *FREM2* | 341640 | XM_929884 | -1.679 |
| *FREM2* | 341640 | NM_207361 | -1.101 |
| *FRMD4B* | 23150 | AB023230 | -1.248 |
| *FRMD6* | 122786 | AK055545 | -1.464 |
| *FTCD* | 10841 | NM_006657 | -1.105 |
| *FTCD* | 10841 | NM_206965 | -1.149 |
| *FXYD6* | 53826 | NM_022003 | -1.369 |
| *FXYD7* | 53822 | BC018619 | -1.075 |
| *FZD5* | 7855 | NM_003468 | -1.048 |
| *GABARAPL1* | 23710 | NM_031412 | -1.106 |
| *GADD45A* | 1647 | NM_001924 | -1.093 |
| *GADD45B* | 4616 | NM_015675 | -1.707 |
| *GADD45B* | 4616 | AF087853 | -1.962 |
| *GADD45G* | 10912 | NM_006705 | -1.377 |
| *GADD45G* | 10912 | BC000465 | -1.386 |
| *GALC* | 2581 | NM_001037525 | -1.331 |
| *GALNT14* | 79623 | BC010659 | -1.26 |
| *GBA3* | 57733 | BC101829 | -2.634 |
| *GBA3* | 57733 | NM_020973 | -2.32 |
| *GBA3* | 57733 | BC109377 | -2.088 |
| *GCAT* | 23464 | NM_014291 | -1.159 |
| *GCDH* | 2639 | NM_000159 | -1.034 |
| *GCDH* | 2639 | NM_013976 | -1.038 |
| *GCDH* | 2639 | BC002579 | -1.02 |
| *GCGR* | 2642 | NM_000160 | -2.082 |
| *GCGR* | 2642 | BC104854 | -2.306 |
| *GCH1* | 2643 | NM_001024024 | -1.802 |
| *GCH1* | 2643 | NM_000161 | -1.327 |
| *GCH1* | 2643 | NM_001024070 | -1.299 |
| *GCKR* | 2646 | NM_001486 | -1.137 |
| *GCNT2* | 2651 | BC074802 | -1.347 |
| *Gcom1* | 145781 | NM_001018096 | -1.196 |
| *Gcom1* | 145781 | BC101645 | -1.014 |
| *Gcom1* | 145781 | NM_001018100 | -1.086 |
| *GDF2* | 2658 | BC074921 | -4.225 |
| *GDF2* | 2658 | BC069643 | -3.028 |
| *GDF2* | 2658 | NM_016204 | -2.748 |
| *GFRA1* | 2674 | BC014962 | -2.517 |
| *GFRA1* | 2674 | NM_145793 | -2.72 |
| *GFRA1* | 2674 | AF042080 | -2.662 |
| *GHR* | 2690 | NM_000163 | -2.614 |
| *GIPC2* | 54810 | NM_017655 | -1.363 |
| *GK* | 2710 | BC037549 | -1.07 |
| *GLS2* | 27165 | NM_013267 | -2.671 |
| *GLUD2* | 2747 | NM_012084 | -1.254 |
| *GLULD1* | 51557 | NM_016571 | -2.272 |
| *GLYAT* | 10249 | NM_005838 | -2.161 |
| *GLYAT* | 10249 | NM_201648 | -1.955 |
| *GLYATL1* | 92292 | BC008353 | -1.233 |
| *GLYATL1* | 92292 | NM_080661 | -1.322 |
| *GNA14* | 9630 | NM_004297 | -1.321 |
| *GNAO1* | 2775 | NM_138736 | -1.247 |
| *GNAO1* | 2775 | NM_020988 | -2.782 |
| *GNAO1* | 2775 | BC030027 | -2.1 |
| *GNAQ* | 2776 | NM_002072 | -1.579 |
| *GNAQ* | 2776 | BC069520 | -1.762 |
| *GNAQ* | 2776 | BC075096 | -1.562 |
| *GNE* | 10020 | AF051852 | -1.778 |
| *GNE* | 10020 | NM_005476 | -2.066 |
| *GNMT* | 27232 | NM_018960 | -1.67 |
| *GP6* | 51206 | BC069485 | -1.365 |
| *GPM6A* | 2823 | NM_005277 | -3.397 |
| *GPM6A* | 2823 | BC044612 | -2.695 |
| *GPR125* | 166647 | NM_145290 | -1.207 |
| *GPR126* | 57211 | NM_001032394 | -1.266 |
| *GPR128* | 84873 | NM_032787 | -2.946 |
| *GPR180* | 160897 | BC033906 | -1.791 |
| *GPR180* | 160897 | BC052243 | -1.513 |
| *GPRASP2* | 114928 | NM_001004051 | -1.69 |
| *GPRASP2* | 114928 | BC013576 | -1.549 |
| *GPT* | 2875 | NM_005309 | -1.031 |
| *GPT2* | 84706 | BC062555 | -1.166 |
| *GRAMD1C* | 54762 | NM_017577 | -3.188 |
| *GREM2* | 64388 | BC046632 | -2.795 |
| *GREM2* | 64388 | NM_022469 | -2.699 |
| *GRM8* | 2918 | AJ236921 | -2.678 |
| *GRM8* | 2918 | NM_000845 | -1.955 |
| *GRM8* | 2918 | AJ236922 | -2.631 |
| *GRM8* | 2918 | BC093725 | -2.012 |
| *GSTZ1* | 2954 | NM_145870 | -1.519 |
| *GSTZ1* | 2954 | BC001453 | -1.334 |
| *GSTZ1* | 2954 | NM_001513 | -1.398 |
| *GYG2* | 8908 | U94363 | -1.678 |
| *GYS2* | 2998 | NM_021957 | -2.985 |
| *H6PD* | 9563 | BC081559 | -1.085 |
| *HAMP* | 57817 | NM_021175 | -3.412 |
| *HAO2* | 51179 | NM_001005783 | -2.587 |
| *HAO2* | 51179 | BC020863 | -2.331 |
| *HAO2* | 51179 | NM_016527 | -1.769 |
| *HAPLN4* | 404037 | NM_023002 | -1.563 |
| *HBA1* | 3039 | NM_000558 | -1.416 |
| *HBA2* | 3040 | NM_000517 | -1.529 |
| *HBD* | 3045 | BC069307 | -1.542 |
| *HEPN1* | 641654 | NM_001037558 | -3.198 |
| *HERC3* | 8916 | NM_014606 | -1.086 |
| *HGFAC* | 3083 | NM_001528 | -3.087 |
| *HHIP* | 64399 | BC025311 | -2.205 |
| *HHIP* | 64399 | NM_022475 | -1.334 |
| *HIBCH* | 26275 | NM_198047 | -1.24 |
| *HIBCH* | 26275 | U66669 | -1.191 |
| *HIBCH* | 26275 | NM_014362 | -1.214 |
| *HIBCH* | 26275 | BC067822 | -1.127 |
| *HIPK3* | 10114 | AF305239 | -1.379 |
| *HIVEP1* | 3096 | NM_002114 | -1.057 |
| *HK3* | 3101 | NM_002115 | -1.326 |
| *HMFN0672* | 389643 | AK129923 | -1.828 |
| *HMFN0672* | 389643 | XM_372035 | -1.905 |
| *HMFN0839* | 84803 | NM_032717 | -1.694 |
| *HOMER2* | 9455 | NM_004839 | -1.537 |
| *HOMER2* | 9455 | NM_199331 | -1.416 |
| *HOOK1* | 51361 | NM_015888 | -1.19 |
| *HRASLS3* | 11145 | BC103807 | -1.253 |
| *HS3ST3A1* | 9955 | BC044647 | -1.625 |
| *HS3ST3A1* | 9955 | NM_006042 | -1.436 |
| *HS3ST3B1* | 9953 | NM_006041 | -1.819 |
| *HS3ST3B1* | 9953 | BC063301 | -2.184 |
| *HSD11B1* | 3290 | NM_005525 | -1.123 |
| *HSD11B1* | 3290 | NM_181755 | -1.152 |
| *HSD11B1* | 3290 | BC012593 | -1.085 |
| *HSD17B12* | 51144 | AK222881 | -1.089 |
| *HSD17B13* | 345275 | BC112303 | -2.753 |
| *HSD17B13* | 345275 | NM_178135 | -3.11 |
| *IBRDC1* | 154214 | NM_152553 | -1.14 |
| *ID1* | 3397 | NM_181353 | -2.017 |
| *ID1* | 3397 | NM_002165 | -1.629 |
| *IGF1* | 3479 | NM_000618 | -1.861 |
| *IGF2* | 3481 | BC053318 | -4.183 |
| *IGF2* | 3481 | NM_000612 | -5.133 |
| *IGFALS* | 3483 | NM_004970 | -3.162 |
| *IGFBP3* | 3486 | NM_000598 | -1.663 |
| *IGFBP3* | 3486 | BC000013 | -1.606 |
| *IL13RA1* | 3597 | U81380 | -1.163 |
| *IL13RA2* | 3598 | NM_000640 | -3.76 |
| *IL17RC* | 84818 | NM_153463 | -1.052 |
| *IL18R1* | 8809 | BC069575 | -1.532 |
| *IL18R1* | 8809 | NM_003855 | -1.622 |
| *IL18R1* | 8809 | BC093975 | -1.029 |
| *IL18R1* | 8809 | BC093977 | -1.038 |
| *IL1B* | 3553 | NM_000576 | -1.84 |
| *IL1R1* | 3554 | BC067507 | -1.306 |
| *IL1R1* | 3554 | BC075062 | -1.614 |
| *IL1RAP* | 3556 | NM_002182 | -1.76 |
| *IL1RAP* | 3556 | BC053621 | -2.097 |
| *IL1RAP* | 3556 | NM_134470 | -2.051 |
| *IL1RAPL2* | 26280 | BC104784 | -1.043 |
| *IL1RL1* | 9173 | NM_016232 | -1.588 |
| *IL1RN* | 3557 | NM_000577 | -1.093 |
| *IL8RA* | 3577 | BC028221 | -1.467 |
| *IL8RA* | 3577 | NM_000634 | -1.037 |
| *IL8RB* | 3579 | NM_001557 | -1.678 |
| *IL8RB* | 3579 | BC037961 | -1.634 |
| *INDOL1* | 169355 | NM_194294 | -3.723 |
| *INHBC* | 3626 | NM_005538 | -1.422 |
| *INMT* | 11185 | BC106902 | -1.218 |
| *INTS6* | 26512 | BC039829 | -1.08 |
| *IQGAP2* | 10788 | NM_006633 | -1.423 |
| *IRF8* | 3394 | NM_002163 | -1.534 |
| *ISOC1* | 51015 | BC008367 | -1.008 |
| *ITLN1* | 55600 | NM_017625 | -1.668 |
| *IVD* | 3712 | BC017202 | -1.3 |
| *IVD* | 3712 | NM_002225 | -1.314 |
| *JDP2* | 122953 | NM_130469 | -1.136 |
| *JMJD3* | 23135 | XM_043272 | -1.21 |
| *JMJD5* | 79831 | BC027911 | -2.38 |
| *JMJD5* | 79831 | NM_024773 | -1.99 |
| *KBTBD11* | 9920 | NM_014867 | -3.984 |
| *KCNK17* | 89822 | NM_031460 | -2.207 |
| *KCNK6* | 9424 | NM_004823 | -1.023 |
| *KCNMA1* | 3778 | AB113382 | -1.559 |
| *KCNN2* | 3781 | NM_170775 | -4.12 |
| *KCNN2* | 3781 | NM_021614 | -2.382 |
| *KDELC2* | 143888 | AK125519 | -1.447 |
| *KIAA0040* | 9674 | BC111699 | -1.251 |
| *KIAA0562* | 9731 | NM_014704 | -1.184 |
| *KIAA0774* | 23281 | NM_001033602 | -1.112 |
| *KIAA0774* | 23281 | NM_015233 | -1.297 |
| *KIAA0894* | 22833 | AB020701 | -1.622 |
| *KIAA1026* | 23254 | NM_015209 | -1.739 |
| *KIAA1026* | 23254 | NM_001017999 | -1.502 |
| *KIAA1026* | 23254 | NM_001018001 | -1.453 |
| *KIAA1026* | 23254 | AB028949 | -1.756 |
| *KIAA1026* | 23254 | NM_001018000 | -1.459 |
| *KIAA1109* | 84162 | XM_934076 | -1.131 |
| *KIAA1155* | 400961 | XM_376062 | -2.056 |
| *KIAA1217* | 56243 | NM_019590 | -1.332 |
| *KIAA1217* | 56243 | BX640796 | -1.437 |
| *KIAA1370* | 56204 | NM_019600 | -1.19 |
| *KIAA1450* | 57600 | AB040883 | -1.191 |
| *KIAA1571* | 57683 | XM_371590 | -1.142 |
| *KIAA1713* | 80816 | AB051500 | -1.827 |
| *KIF25* | 3834 | NM_005355 | -1.224 |
| *KLC4* | 89953 | NM_138343 | -1.268 |
| *KLF10* | 7071 | NM_001032282 | -1.354 |
| *KLF11* | 8462 | BC069383 | -1.54 |
| *KLF11* | 8462 | NM_003597 | -1.434 |
| *KLF11* | 8462 | BC074922 | -1.472 |
| *KLF4* | 9314 | BC030811 | -1.261 |
| *KLF4* | 9314 | NM_004235 | -1.289 |
| *KLF9* | 687 | NM_001206 | -1.296 |
| *KLHL15* | 80311 | BC109058 | -1.48 |
| *KLHL15* | 80311 | NM_030624 | -1.27 |
| *KLHL2* | 11275 | BC036468 | -1.448 |
| *KLHL2* | 11275 | NM_007246 | -1.381 |
| *KLHL3* | 26249 | NM_017415 | -1.924 |
| *KLKB1* | 3818 | NM_000892 | -1.779 |
| *KLRD1* | 3824 | AJ000001 | -1.391 |
| *KLRD1* | 3824 | NM_007334 | -1.145 |
| *KLRD1* | 3824 | AF498040 | -1.36 |
| *KLRD1* | 3824 | AF498041 | -1.023 |
| *KLRF1* | 51348 | BC098354 | -1.692 |
| *KLRF1* | 51348 | NM_016523 | -1.466 |
| *KMO* | 8564 | BC005297 | -1.82 |
| *L3MBTL3* | 84456 | BC060845 | -1.148 |
| *LARP2* | 55132 | NM_178043 | -1.493 |
| *LARP2* | 55132 | AK001240 | -1.458 |
| *LARP2* | 55132 | BC110300 | -1.404 |
| *LARP2* | 55132 | NM_032239 | -1.343 |
| *LARP2* | 55132 | NM_018078 | -1.054 |
| *LARP2* | 55132 | BX537937 | -1.354 |
| *LATS2* | 26524 | NM_014572 | -1.031 |
| *LCAT* | 3931 | NM_000229 | -1.764 |
| *LDB2* | 9079 | AF064493 | -1.334 |
| *LDB2* | 9079 | NM_001290 | -1.234 |
| *LDHD* | 197257 | BC047902 | -1.203 |
| *LDHD* | 197257 | NM_153486 | -1.121 |
| *LEAP-2* | 116842 | NM_052971 | -1.054 |
| *LEPR* | 3953 | NM_001003679 | -2.098 |
| *LEPREL1* | 55214 | NM_018192 | -1.953 |
| *LHX2* | 9355 | BC093662 | -2.176 |
| *LHX2* | 9355 | NM_004789 | -2.185 |
| *LHX2* | 9355 | AK094511 | -1.732 |
| *LIFR* | 3977 | NM_002310 | -3.471 |
| *LILRA1* | 11024 | BC093708 | -1.151 |
| *LILRA1* | 11024 | NM_006863 | -1.259 |
| *LILRA2* | 11027 | NM_006866 | -1.232 |
| *LILRA3* | 11026 | AF014924 | -1.022 |
| *LILRA5* | 353514 | NM_181985 | -1.478 |
| *LILRB2* | 10288 | BC036827 | -1.078 |
| *LILRB5* | 10990 | AK223296 | -1.3 |
| *LILRB5* | 10990 | NM_006840 | -1.065 |
| *LIPG* | 9388 | NM_006033 | -1.192 |
| *LMAN1* | 3998 | NM_005570 | -2.224 |
| *LOC131873* | 131873 | XM_067585 | -1.001 |
| *LOC133308* | 133308 | BC047447 | -1.675 |
| *LOC133308* | 133308 | NM_178833 | -1.855 |
| *LOC143941* | 143941 | XM_084672 | -1.777 |
| *LOC144481* | 144481 | XM_931702 | -1.811 |
| *LOC161247* | 161247 | NM_203402 | -2.048 |
| *LOC201895* | 201895 | NM_174921 | -1.035 |
| *LOC280665* | 280665 | AF547222 | -1.925 |
| *LOC283398* | 283398 | XM_931770 | -1.037 |
| *LOC283874* | 283874 | NM_001012731 | -1.124 |
| *LOC285498* | 285498 | NM_194439 | -1.11 |
| *LOC339105* | 339105 | NM_001039503 | -1.112 |
| *LOC339240* | 339240 | BC040601 | -1.93 |
| *LOC339240* | 339240 | AK127974 | -1.758 |
| *LOC342979* | 342979 | XM_292820 | -1.088 |
| *LOC374569* | 374569 | XM_930559 | -2.209 |
| *LOC374569* | 374569 | XM_370777 | -2.271 |
| *LOC374569* | 374569 | XM_935431 | -2.203 |
| *LOC388503* | 388503 | NM_001013640 | -2.019 |
| *LOC400553* | 400553 | AK126852 | -1.197 |
| *LOC402560* | 402560 | XM_379506 | -1.166 |
| *LOC440508* | 440508 | BC047593 | -1.536 |
| *LOC54103* | 54103 | NM_017439 | -1.009 |
| *LOC554235* | 554235 | NM_001024656 | -1.03 |
| *LOC619208* | 619208 | NM_001033564 | -1.085 |
| *LOC642262* | 642262 | XM_925810 | -1.346 |
| *LOC642561* | 642561 | XM_926047 | -1.545 |
| *LOC643554* | 643554 | XM_926857 | -1.665 |
| *LOC643637* | 643637 | XM_926941 | -1.376 |
| *LOC644462* | 644462 | XM_930312 | -1.012 |
| *LOC644578* | 644578 | XM_927694 | -1.637 |
| *LOC645039* | 645039 | XM_928095 | -2.063 |
| *LOC645650* | 645650 | XM_928664 | -1.432 |
| *LOC645745* | 645745 | NM_001039954 | -1.836 |
| *LOC646037* | 646037 | XM_928998 | -1.066 |
| *LOC653107* | 653107 | XM_931361 | -2.548 |
| *LOC653107* | 653107 | XM_931342 | -1.099 |
| *LOC653107* | 653107 | NM_001040084 | -2.427 |
| *LOC653107* | 653107 | XM_931353 | -2.665 |
| *LOC653107* | 653107 | XM_926508 | -2.756 |
| *LOC653107* | 653107 | XM_931388 | -2.31 |
| *LOC653197* | 653197 | XM_932726 | -2.202 |
| *LOC653299* | 653299 | XM_932790 | -2.586 |
| *LOC653299* | 653299 | XM_932796 | -1.742 |
| *LOC653326* | 653326 | XM_928507 | -1.512 |
| *LOC653330* | 653330 | XM_933390 | -1.51 |
| *LOC653480* | 653480 | XM_932187 | -1.016 |
| *LOC653489* | 653489 | XM_934115 | -1.469 |
| *LOC653513* | 653513 | XM_927826 | -1.472 |
| *LOC653521* | 653521 | XM_929861 | -2.521 |
| *LOC653589* | 653589 | XM_928274 | -1.039 |
| *LOC653701* | 653701 | XM_929047 | -1.43 |
| *LOC653762* | 653762 | XM_929519 | -1.391 |
| *LOC92689* | 92689 | BC001096 | -1.079 |
| *LOC92689* | 92689 | NM_138389 | -1.092 |
| *LONPL* | 83752 | BC093910 | -1.138 |
| *LONRF1* | 91694 | NM_152271 | -1.747 |
| *LONRF3* | 79836 | NM_001031855 | -1.334 |
| *LPA* | 4018 | XM_926329 | -2.155 |
| *LPAL2* | 80350 | NM_145727 | -1.441 |
| *LPAL2* | 80350 | XM_935128 | -1.819 |
| *LPAL2* | 80350 | BC110570 | -1.437 |
| *LPAL2* | 80350 | NM_024492 | -1.522 |
| *LRAT* | 9227 | NM_004744 | -1.752 |
| *LRRC25* | 126364 | BC093842 | -1.287 |
| *LRRC4* | 64101 | AJ297858 | -1.749 |
| *LRRK2* | 120892 | XM_930820 | -1.566 |
| *LRRN3* | 54674 | NM_018334 | -1.516 |
| *LST-3TM12* | 338821 | NM_001009562 | -2.3 |
| *LTBP1* | 4052 | NM_000627 | -1.471 |
| *LTK* | 4058 | BC045607 | -1.899 |
| *LY6E* | 4061 | BC003392 | -1.713 |
| *LY6E* | 4061 | NM_002346 | -1.553 |
| *LZTFL1* | 54585 | BC042483 | -1.032 |
| *LZTFL1* | 54585 | NM_020347 | -1.367 |
| *MAB21L2* | 10586 | NM_006439 | -1.05 |
| *MACF1* | 23499 | AK127519 | -1.14 |
| *MAN1A1* | 4121 | NM_005907 | -1.525 |
| *MAN1A1* | 4121 | BC065827 | -2.255 |
| *MAN1C1* | 57134 | NM_020379 | -2.033 |
| *MAP2K1* | 5604 | NM_002755 | -1.061 |
| *MAP2K3* | 5606 | NM_002756 | -1.112 |
| *MAP2K3* | 5606 | BC032478 | -1.099 |
| *MAP3K7IP2* | 23118 | NM_015093 | -1.41 |
| *MAP3K7IP2* | 23118 | BC035910 | -1.174 |
| *MAPRE3* | 22924 | NM_012326 | -1.019 |
| *MARCO* | 8685 | NM_006770 | -3.152 |
| *MASP1* | 5648 | NM_139125 | -2.307 |
| *MASP1* | 5648 | BC106945 | -1.93 |
| *MASP1* | 5648 | NM_001879 | -2.21 |
| *MASP1* | 5648 | NM_001031849 | -1.223 |
| *MASP2* | 10747 | NM_006610 | -1.291 |
| *MAWBP* | 64081 | BC009738 | -2.026 |
| *MAWBP* | 64081 | AK027673 | -2.146 |
| *MAWBP* | 64081 | NM_022129 | -1.63 |
| *MAWBP* | 64081 | NM_001033083 | -1.291 |
| *MAWBP* | 64081 | AK092826 | -1.453 |
| *MBL2* | 4153 | BC069338 | -2.235 |
| *MBL2* | 4153 | NM_000242 | -1.884 |
| *MBNL2* | 10150 | BC104038 | -2.7 |
| *MBNL2* | 10150 | AF061261 | -2.057 |
| *MBNL2* | 10150 | NM_144778 | -1.941 |
| *MBTPS1* | 8720 | NM_201268 | -1.241 |
| *MCC* | 4163 | BC009279 | -1.867 |
| *MCC* | 4163 | NM_002387 | -2.97 |
| *MCEE* | 84693 | NM_032601 | -1.069 |
| *MCL1* | 4170 | BC107735 | -1.467 |
| *MCTP2* | 55784 | NM_018349 | -1.412 |
| *MDN1* | 23195 | NM_014611 | -1.484 |
| *MEFV* | 4210 | BC101537 | -1.24 |
| *MEFV* | 4210 | BC101511 | -1.255 |
| *MEFV* | 4210 | NM_000243 | -1.391 |
| *MEST* | 4232 | NM_002402 | -1.622 |
| *MFAP3L* | 9848 | NM_021647 | -1.271 |
| *MFAP3L* | 9848 | NM_001009554 | -1.793 |
| *MFAP3L* | 9848 | BC066912 | -1.497 |
| *MFSD2* | 84879 | NM_032793 | -3.332 |
| *MFSD2* | 84879 | AK027396 | -1.669 |
| *MGC10701* | 84744 | BC004487 | -1.073 |
| *MGC14376* | 84981 | NM_001001870 | -1.228 |
| *MGC14376* | 84981 | NM_032895 | -1.155 |
| *MGC16121* | 84848 | BC007360 | -1.649 |
| *MGC35308* | 285800 | BC034775 | -1.231 |
| *MGC35308* | 285800 | NM_175922 | -1.726 |
| *MGC39518* | 285172 | NM_173822 | -1.167 |
| *MGC70870* | 403340 | NM_203481 | -1.411 |
| *MGLL* | 11343 | AK125135 | -1.762 |
| *MICALCL* | 84953 | NM_032867 | -1.09 |
| *MIP* | 4284 | NM_012064 | -1.038 |
| *MMAA* | 166785 | NM_172250 | -1.017 |
| *MME* | 4311 | BC101632 | -2.617 |
| *MME* | 4311 | X07166 | -2.821 |
| *MMRN1* | 22915 | BC063848 | -2.101 |
| *MMRN1* | 22915 | NM_007351 | -1.399 |
| *MOGAT2* | 80168 | NM_025098 | -3.234 |
| *MOGAT2* | 80168 | AK128620 | -2.952 |
| *MOGAT2* | 80168 | BC103878 | -2.4 |
| *MORC3* | 23515 | NM_015358 | -1.329 |
| *MOSC1* | 64757 | NM_022746 | -1.257 |
| *MOSC2* | 54996 | NM_017898 | -1.267 |
| *MPDZ* | 8777 | NM_003829 | -1.882 |
| *MPEG1* | 219972 | BC104997 | -1.121 |
| *MPEG1* | 219972 | XM_166227 | -1.408 |
| *MPPED1* | 758 | NM_001585 | -1.861 |
| *MRC1L1* | 414308 | NM_001009567 | -2.725 |
| *MRGPRF* | 219928 | NM_145015 | -1.451 |
| *MRGPRF* | 219928 | BC016964 | -1.487 |
| *MRGPRF* | 219928 | AK075492 | -1.158 |
| *MRO* | 83876 | BC029860 | -2.341 |
| *MRO* | 83876 | NM_031939 | -1.899 |
| *MSRA* | 4482 | AY958431 | -1.289 |
| *MSRA* | 4482 | BC054033 | -1.173 |
| *MSRA* | 4482 | NM_012331 | -1.111 |
| *MT1A* | 4489 | BC029475 | -1.545 |
| *MT1A* | 4489 | NM_005946 | -1.471 |
| *MT1E* | 4493 | AF495759 | -2.414 |
| *MT1F* | 4494 | NM_005949 | -2.79 |
| *MT1G* | 4495 | NM_005950 | -2.768 |
| *MT1G* | 4495 | BC020757 | -2.481 |
| *MT1H* | 4496 | NM_005951 | -1.574 |
| *MT1H* | 4496 | BC008408 | -1.677 |
| *MT1JP* | 4498 | NM_175622 | -1.654 |
| *MT1M* | 4499 | BC103841 | -2.167 |
| *MT1M* | 4499 | NM_176870 | -2.164 |
| *MT1X* | 4501 | BC032131 | -2.071 |
| *MT1X* | 4501 | NM_005952 | -1.728 |
| *MT1X* | 4501 | BC018190 | -1.727 |
| *MT2A* | 4502 | BC007034 | -1.506 |
| *MT2A* | 4502 | NM_005953 | -1.428 |
| *MTHFD2L* | 441024 | BC032771 | -1.299 |
| *MTMR9* | 66036 | BC022003 | -1.349 |
| *MUCDHL* | 53841 | NM_017717 | -1.485 |
| *MUM1L1* | 139221 | BC031229 | -1.02 |
| *MUT* | 4594 | NM_000255 | -1.316 |
| *MUT* | 4594 | BC016282 | -1.27 |
| *MYO10* | 4651 | NM_012334 | -2.021 |
| *MYO15A* | 51168 | NM_016239 | -1.207 |
| *MYO1B* | 4430 | AK022489 | -1.745 |
| *MYOM2* | 9172 | NM_003970 | -2.309 |
| *MYOM2* | 9172 | BC052969 | -2.064 |
| *NAB1* | 4664 | U47007 | -1.406 |
| *NALP12* | 91662 | NM_144687 | -1.128 |
| *NALP12* | 91662 | AK095460 | -1.401 |
| *NALP12* | 91662 | AY116204 | -1.37 |
| *NAP1L5* | 266812 | BC104883 | -1.098 |
| *NAT1* | 9 | NM_000662 | -1.916 |
| *NAT2* | 10 | BC015878 | -2.99 |
| *NAT2* | 10 | NM_000015 | -2.715 |
| *NAV2* | 89797 | NM_145117 | -1.267 |
| *NBLA00301* | 79804 | XM_496724 | -1.586 |
| *NBLA00301* | 79804 | AB075501 | -1.514 |
| *NDEL1* | 81565 | NM_001025579 | -1.002 |
| *NDFIP2* | 54602 | BC021988 | -1.384 |
| *NDRG2* | 57447 | AF087872 | -1.765 |
| *NDRG2* | 57447 | NM_016250 | -1.869 |
| *NDRG2* | 57447 | BC011240 | -1.745 |
| *NDRG2* | 57447 | BC010458 | -1.284 |
| *NDST3* | 9348 | NM_004784 | -1.016 |
| *NEBL* | 10529 | NM_006393 | -1.095 |
| *NFATC1* | 4772 | NM_006162 | -1.057 |
| *NFE2* | 4778 | NM_006163 | -1.409 |
| *NFE2* | 4778 | BC005044 | -1.255 |
| *NGFR* | 4804 | NM_002507 | -2.085 |
| *NGFR* | 4804 | BC050309 | -2.021 |
| *NHLRC2* | 374354 | AK126751 | -1.213 |
| *NHLRC2* | 374354 | BX647641 | -1.235 |
| *NHSL1* | 57224 | XM_933674 | -1.563 |
| *NHSL1* | 57224 | XM_496826 | -1.622 |
| *NID1* | 4811 | NM_002508 | -1.011 |
| *NME5* | 8382 | NM_003551 | -1.255 |
| *NOL4* | 8715 | BC000313 | -1.53 |
| *NOL4* | 8715 | NM_003787 | -1.264 |
| *NOTCH2* | 4853 | AF308601 | -1.5 |
| *NPAL1* | 152519 | NM_207330 | -1.602 |
| *NPY1R* | 4886 | NM_000909 | -3.336 |
| *NPY1R* | 4886 | BC071720 | -3.411 |
| *NR3C2* | 4306 | BC111758 | -1.709 |
| *NR3C2* | 4306 | AJ315514 | -1.105 |
| *NR4A1* | 3164 | NM_002135 | -1.321 |
| *NR4A3* | 8013 | D78579 | -1.135 |
| *NRBF2* | 29982 | BC011707 | -1.133 |
| *NRBF2* | 29982 | NM_030759 | -1.043 |
| *NRG1* | 3084 | NM_013959 | -1.223 |
| *NRG1* | 3084 | NM_013956 | -1.603 |
| *NRG1* | 3084 | NM_013958 | -2.607 |
| *NRG1* | 3084 | NM_013962 | -2.125 |
| *NRG1* | 3084 | NM_013961 | -2.54 |
| *NT5C1B* | 93034 | NM_001002006 | -1.317 |
| *NT5DC1* | 221294 | NM_152729 | -1.263 |
| *NTF3* | 4908 | BC069773 | -2.442 |
| *NTF3* | 4908 | NM_002527 | -2.567 |
| *NTN4* | 59277 | AF297711 | -2.29 |
| *NTN4* | 59277 | NM_021229 | -2.297 |
| *NUDT10* | 170685 | BC049383 | -1.21 |
| *NXF3* | 56000 | BC031616 | -1.53 |
| *NXF3* | 56000 | NM_022052 | -1.262 |
| *OAT* | 4942 | NM_000274 | -1.985 |
| *OBSL1* | 23363 | AK023854 | -1.437 |
| *OIT3* | 170392 | NM_152635 | -2.681 |
| *OLFML3* | 56944 | AK092766 | -2.47 |
| *OLFML3* | 56944 | NM_020190 | -2.315 |
| *OSBPL11* | 114885 | NM_022776 | -1.565 |
| *OTUD4* | 54726 | NM_199324 | -1.114 |
| *P2RY13* | 53829 | NM_023914 | -2.053 |
| *PALM2* | 114299 | AK095003 | -1.442 |
| *PALM2* | 114299 | BC039306 | -1.534 |
| *PALM2-AKAP2* | 445815 | NM_147150 | -1.225 |
| *PANK1* | 53354 | NM_138316 | -1.747 |
| *PAPSS2* | 9060 | BC009894 | -1.076 |
| *PBX1* | 5087 | BC101578 | -1.762 |
| *PBX1* | 5087 | NM_002585 | -1.071 |
| *PCDH7* | 5099 | NM_032456 | -1.317 |
| *PCDHGB4* | 8641 | NM_032098 | -1.224 |
| *PCK2* | 5106 | NM_001018073 | -1.204 |
| *PCSK6* | 5046 | NM_138325 | -1.401 |
| *PCSK6* | 5046 | NM_138322 | -1.093 |
| *PCSK6* | 5046 | NM_138320 | -1.197 |
| *PCYOX1* | 51449 | NM_016297 | -1.357 |
| *PCYOX1* | 51449 | BC051891 | -1.316 |
| *PDE11A* | 50940 | NM_016953 | -1.163 |
| *PDE2A* | 5138 | AY495087 | -1.813 |
| *PDE2A* | 5138 | NM_002599 | -1.669 |
| *PDE2A* | 5138 | BC040974 | -1.352 |
| *PDE3B* | 5140 | NM_000922 | -1.375 |
| *PDE4DIP* | 9659 | NM_022359 | -1.4 |
| *PDE4DIP* | 9659 | NM_001002810 | -1.535 |
| *PDE7B* | 27115 | BC075082 | -1.332 |
| *PDE7B* | 27115 | NM_018945 | -1.821 |
| *PDGFC* | 56034 | NM_016205 | -1.325 |
| *PDGFRA* | 5156 | NM_006206 | -2.772 |
| *PDLIM5* | 10611 | NM_001011515 | -1.182 |
| *PDLIM5* | 10611 | BC008741 | -1.116 |
| *PECR* | 55825 | AF212234 | -1.49 |
| *PECR* | 55825 | NM_018441 | -1.313 |
| *PGA5* | 5222 | NM_014224 | -1.08 |
| *PGLYRP2* | 114770 | AF384856 | -1.875 |
| *PHGDH* | 26227 | NM_006623 | -1.473 |
| *PHLDA1* | 22822 | NM_007350 | -1.469 |
| *PHLDA1* | 22822 | BC018929 | -1.378 |
| *PHLDB2* | 90102 | NM_145753 | -1.125 |
| *PIK3R1* | 5295 | BC030815 | -1.276 |
| *PIK3R1* | 5295 | BC094795 | -1.597 |
| *PKHD1* | 5314 | NM_138694 | -2.534 |
| *PLAC8* | 51316 | NM_016619 | -2.571 |
| *PLAC8* | 51316 | BC012205 | -2.258 |
| *PLCXD3* | 345557 | NM_001005473 | -1.723 |
| *PLD1* | 5337 | NM_002662 | -2.076 |
| *PLD1* | 5337 | AB209907 | -1.448 |
| *PLEKHC1* | 10979 | BX161467 | -1.239 |
| *PLEKHC1* | 10979 | BC017327 | -1.219 |
| *PLEKHC1* | 10979 | NM_006832 | -1.084 |
| *PLGLA1* | 285189 | XM_934125 | -1.416 |
| *PLGLA1* | 285189 | XM_934126 | -1.285 |
| *PLGLA1* | 285189 | XM_376111 | -1.707 |
| *PLGLB1* | 5343 | BC109248 | -1.197 |
| *PLGLB1* | 5343 | NM_001032392 | -1.273 |
| *PLGLB2* | 5342 | BC022294 | -1.28 |
| *PLGLB2* | 5342 | BC005379 | -1.159 |
| *PLIN* | 5346 | BC031084 | -2.105 |
| *PLIN* | 5346 | NM_002666 | -1.836 |
| *PLSCR1* | 5359 | BC017901 | -1.066 |
| *PLSCR4* | 57088 | NM_020353 | -2.147 |
| *PNRC1* | 10957 | BC018112 | -1.228 |
| *PNRC1* | 10957 | AY303779 | -1.241 |
| *PNRC1* | 10957 | NM_006813 | -1.001 |
| *PNRC1* | 10957 | BC044919 | -1.14 |
| *PON3* | 5446 | NM_000940 | -1.212 |
| *POU6F2* | 11281 | NM_007252 | -1.196 |
| *PPAP2B* | 8613 | NM_003713 | -1.748 |
| *PPAP2B* | 8613 | BC009196 | -1.338 |
| *PPAPDC1A* | 196051 | NM_001030059 | -1.753 |
| *PPARGC1A* | 10891 | AB061325 | -1.855 |
| *PPFIBP1* | 8496 | NM_003622 | -1.592 |
| *PPL* | 5493 | NM_002705 | -1.551 |
| *PPM1K* | 152926 | AF351614 | -1.339 |
| *PPM1K* | 152926 | NM_152542 | -1.06 |
| *PPP1R3B* | 79660 | NM_024607 | -2.222 |
| *PPP1R3B* | 79660 | BC043388 | -1.642 |
| *PPP1R3G* | 389364 | XM_371796 | -1.709 |
| *PPP2CB* | 5516 | NM_001009552 | -1.069 |
| *PPP2R1B* | 5519 | CR617223 | -1.003 |
| *PRAM1* | 84106 | BC028012 | -1.178 |
| *PRKAG2* | 51422 | BC068598 | -1.331 |
| *PRKAG2* | 51422 | NM_016203 | -1.067 |
| *PROM1* | 8842 | NM_006017 | -2.625 |
| *PROS1* | 5627 | BC015801 | -1.885 |
| *PROZ* | 8858 | M55671 | -1.922 |
| *PRRG2* | 5639 | NM_000951 | -1.01 |
| *PRRG4* | 79056 | BC010052 | -1.256 |
| *PRRG4* | 79056 | NM_024081 | -1.204 |
| *PRSS8* | 5652 | NM_002773 | -3.363 |
| *PSAT1* | 29968 | NM_021154 | -1.62 |
| *PSD3* | 23362 | BC011238 | -1.667 |
| *PSD4* | 23550 | BC035307 | -1.232 |
| *PTCH* | 5727 | U43148 | -1.244 |
| *PTGER2* | 5732 | BC093927 | -1.009 |
| *PTHR1* | 5745 | BC112221 | -2.639 |
| *PTHR1* | 5745 | NM_000316 | -2.518 |
| *PTN* | 5764 | BC005916 | -1.071 |
| *PTPN13* | 5783 | NM_006264 | -1.917 |
| *PTPN3* | 5774 | DQ104439 | -1.173 |
| *PTPRB* | 5787 | BC101679 | -1.404 |
| *PTPRB* | 5787 | NM_002837 | -1.407 |
| *PTPRD* | 5789 | BC106713 | -1.303 |
| *PTPRD* | 5789 | NM_002839 | -1.328 |
| *PTPRT* | 11122 | NM_007050 | -1.184 |
| *PVALB* | 5816 | BC069300 | -1.26 |
| *PVRL3* | 25945 | BC017572 | -1.022 |
| *RAB14* | 51552 | NM_016322 | -1.167 |
| *RAB33B* | 83452 | BC111977 | -1.118 |
| *RAB33B* | 83452 | NM_031296 | -1.378 |
| *RAG1* | 5896 | NM_000448 | -1.248 |
| *RAPGEF4* | 11069 | NM_007023 | -1.036 |
| *RASGEF1B* | 153020 | NM_152545 | -2.114 |
| *RBL2* | 5934 | BC034490 | -1.164 |
| *RBMS3* | 27303 | AF023259 | -1.194 |
| *RCBTB2* | 1102 | NM_001268 | -1.279 |
| *RCHY1* | 25898 | NM_001008925 | -1.209 |
| *RCL1* | 10171 | BC001025 | -1.5 |
| *RCL1* | 10171 | NM_005772 | -1.375 |
| *RDH16* | 8608 | NM_003708 | -1.39 |
| *RDH5* | 5959 | BC028298 | -1.026 |
| *RDH5* | 5959 | NM_002905 | -1.048 |
| *REL* | 5966 | NM_002908 | -1.179 |
| *RET* | 5979 | BC004257 | -2.356 |
| *RET* | 5979 | NM_020630 | -1.416 |
| *RET* | 5979 | BC003072 | -1.151 |
| *REV3L* | 5980 | AF071798 | -1.014 |
| *REV3L* | 5980 | NM_002912 | -1.012 |
| *RGAG4* | 340526 | NM_001024455 | -1.829 |
| *RGN* | 9104 | NM_152869 | -1.729 |
| *RGPD2* | 440872 | NM_001024457 | -1.082 |
| *RGPD4* | 285190 | XM_496581 | -1.597 |
| *RIC3* | 79608 | NM_024557 | -1.793 |
| *RIC3* | 79608 | AK021670 | -1.491 |
| *RIPK4* | 54101 | BC110617 | -1.679 |
| *RIPK4* | 54101 | NM_020639 | -1.426 |
| *RMND5A* | 64795 | BC047668 | -1.077 |
| *RND1* | 27289 | NM_014470 | -1.554 |
| *RND3* | 390 | NM_005168 | -2.265 |
| *RNF125* | 54941 | BC012021 | -1.388 |
| *RNF125* | 54941 | NM_017831 | -1.599 |
| *RNF128* | 79589 | BC063404 | -1.43 |
| *RNF128* | 79589 | NM_024539 | -1.174 |
| *RNF135* | 84282 | NM_197939 | -1.083 |
| *RNF135* | 84282 | NM_032322 | -1.122 |
| *RNF152* | 220441 | BC094004 | -1.319 |
| *RNF152* | 220441 | NM_173557 | -1.344 |
| *RNF165* | 494470 | NM_152470 | -1.675 |
| *RNF180* | 285671 | BC101277 | -1.27 |
| *RNF180* | 285671 | NM_178532 | -1.17 |
| *RP11-169K16.3* | 388595 | NM_001013641 | -1.521 |
| *RP11-321G1.1* | 54885 | AK000305 | -1.094 |
| *RP11-38L15.1* | 439965 | XM_931975 | -1.105 |
| *RP11-54H7.1* | 23026 | AL834447 | -1.075 |
| *RP2* | 6102 | NM_006915 | -1.099 |
| *RP5-860F19.3* | 57593 | XM_044921 | -1.236 |
| *RP5-860F19.3* | 57593 | BC054347 | -1.098 |
| *RSPO3* | 84870 | NM_032784 | -2.67 |
| *SALL1* | 6299 | NM_002968 | -1.122 |
| *SARDH* | 1757 | AF095736 | -1.688 |
| *SARDH* | 1757 | AF095737 | -1.533 |
| *SARDH* | 1757 | NM_007101 | -1.043 |
| *SART2* | 29940 | NM_013352 | -1.285 |
| *SART2* | 29940 | BC039245 | -1.245 |
| *SAV1* | 60485 | BC020537 | -1.049 |
| *SC5DL* | 6309 | BC050427 | -1.778 |
| *SCP2* | 6342 | NM_001007099 | -1.009 |
| *SDPR* | 8436 | BC016475 | -2.336 |
| *SDPR* | 8436 | NM_004657 | -1.495 |
| *SDS* | 10993 | BC020750 | -1.522 |
| *SEC23A* | 10484 | NM_006364 | -1.389 |
| *SEC24B* | 10427 | NM_006323 | -1.043 |
| *SEC24D* | 9871 | BC037736 | -1.255 |
| *SELE* | 6401 | NM_000450 | -1.278 |
| *SERP1* | 27230 | NM_014445 | -1.151 |
| *SERPINE1* | 5054 | NM_000602 | -1.981 |
| *SERPINE1* | 5054 | BC010860 | -1.948 |
| *SESN1* | 27244 | BC112036 | -1.554 |
| *SESN1* | 27244 | NM_014454 | -1.199 |
| *SFRP1* | 6422 | NM_003012 | -2.408 |
| *SFRP5* | 6425 | NM_003015 | -1.751 |
| *SFXN2* | 118980 | NM_178858 | -1.364 |
| *SGEF* | 26084 | BC078655 | -1.647 |
| *SGEF* | 26084 | NM_015595 | -1.314 |
| *SGK* | 6446 | NM_005627 | -1.078 |
| *SH3D19* | 152503 | BC108890 | -1.19 |
| *SH3YL1* | 26751 | BC008374 | -1.251 |
| *SHMT1* | 6470 | BC022874 | -1.064 |
| *SHPRH* | 257218 | NM_173082 | -1.164 |
| *SIAH1* | 6477 | BC035562 | -1 |
| *SIGIRR* | 59307 | AY358342 | -1.066 |
| *SIGLEC11* | 114132 | NM_052884 | -1.076 |
| *SIGLEC7* | 27036 | NM_014385 | -1.144 |
| *SIGLEC7* | 27036 | NM_016543 | -1.237 |
| *SLA/LP* | 51091 | NM_153825 | -1.397 |
| *SLC10A1* | 6554 | NM_003049 | -2.205 |
| *SLC10A1* | 6554 | BC074724 | -1.829 |
| *SLC10A1* | 6554 | BC069799 | -1.771 |
| *SLC16A1* | 6566 | BC045664 | -1.421 |
| *SLC16A10* | 117247 | NM_018593 | -1.72 |
| *SLC16A10* | 117247 | AK172789 | -1.074 |
| *SLC16A4* | 9122 | NM_004696 | -1.852 |
| *SLC16A4* | 9122 | BC021664 | -1.732 |
| *SLC17A2* | 10246 | BC104822 | -1.216 |
| *SLC17A2* | 10246 | NM_005835 | -1.091 |
| *SLC19A2* | 10560 | BC018514 | -1.174 |
| *SLC19A3* | 80704 | BC032014 | -1.647 |
| *SLC1A1* | 6505 | BC033040 | -2.358 |
| *SLC22A1* | 6580 | NM_153187 | -2.064 |
| *SLC22A1* | 6580 | NM_003057 | -1.986 |
| *SLC22A10* | 387775 | XM_933429 | -2.478 |
| *SLC22A10* | 387775 | XM_933441 | -2.608 |
| *SLC22A10* | 387775 | XM_929102 | -2.6 |
| *SLC22A10* | 387775 | NM_001039752 | -2.521 |
| *SLC22A10* | 387775 | XM_370629 | -2.451 |
| *SLC23A2* | 9962 | NM_005116 | -1.844 |
| *SLC23A2* | 9962 | BC013112 | -1.739 |
| *SLC23A2* | 9962 | AF092511 | -1.819 |
| *SLC25A15* | 10166 | NM_014252 | -1.602 |
| *SLC25A15* | 10166 | BC002702 | -1.392 |
| *SLC25A25* | 114789 | BC103930 | -1.343 |
| *SLC2A10* | 81031 | BC101657 | -1.149 |
| *SLC35A1* | 10559 | AJ851889 | -1.122 |
| *SLC35A1* | 10559 | CR626359 | -1.3 |
| *SLC35A1* | 10559 | AJ851890 | -1.027 |
| *SLC35A1* | 10559 | NM_006416 | -1.277 |
| *SLC35A3* | 23443 | CR749816 | -1.11 |
| *SLC35A3* | 23443 | NM_012243 | -1.093 |
| *SLC35A5* | 55032 | BC005207 | -1.234 |
| *SLC37A4* | 2542 | NM_001467 | -1.006 |
| *SLC38A2* | 54407 | NM_018976 | -1.105 |
| *SLC38A4* | 55089 | BC069819 | -2.036 |
| *SLC38A4* | 55089 | AK001053 | -1.864 |
| *SLC39A14* | 23516 | BC015770 | -1.951 |
| *SLC3A1* | 6519 | DQ023514 | -1.406 |
| *SLC3A1* | 6519 | DQ023515 | -2.059 |
| *SLC41A2* | 84102 | BC106871 | -1.517 |
| *SLC41A2* | 84102 | NM_032148 | -1.944 |
| *SLC45A3* | 85414 | NM_033102 | -1.211 |
| *SLC4A4* | 8671 | NM_003759 | -1.383 |
| *SLC4A4* | 8671 | BC030977 | -1.443 |
| *SLC4A4* | 8671 | AF011390 | -1.316 |
| *SLC5A1* | 6523 | NM_000343 | -3.176 |
| *SLC6A12* | 6539 | NM_003044 | -1.675 |
| *SLC6A19* | 340024 | AK096054 | -1.172 |
| *SLC7A2* | 6542 | AL832016 | -2.804 |
| *SLC7A2* | 6542 | NM_001008539 | -1.239 |
| *SLC7A2* | 6542 | BC069648 | -1.971 |
| *SLC7A8* | 23428 | NM_012244 | -1.806 |
| *SLC9A9* | 285195 | NM_173653 | -1.198 |
| *SLCO1B3* | 28234 | AY442326 | -1.441 |
| *SLCO1B3* | 28234 | NM_019844 | -3.684 |
| *SLCO2B1* | 11309 | AF205073 | -1.368 |
| *SLCO4C1* | 353189 | NM_180991 | -2.01 |
| *SMAD6* | 4091 | NM_005585 | -1.338 |
| *SMAD6* | 4091 | BC012986 | -1.355 |
| *SMAD7* | 4092 | BC074818 | -1.073 |
| *SMCY* | 8284 | NM_004653 | -1.482 |
| *SMOC1* | 64093 | BC008608 | -1.193 |
| *SMOC1* | 64093 | BC011548 | -1.557 |
| *SMOC1* | 64093 | NM_001034852 | -1.191 |
| *SNAP29* | 9342 | NM_004782 | -1.155 |
| *SNED1* | 25992 | AF439717 | -1.143 |
| *SNX10* | 29887 | BC034992 | -1.424 |
| *SNX25* | 83891 | NM_031953 | -1.088 |
| *SOCS2* | 8835 | NM_003877 | -2.197 |
| *SOCS6* | 9306 | NM_004232 | -1.23 |
| *SORBS1* | 10580 | BC042612 | -1.658 |
| *SORBS2* | 8470 | AF090937 | -1.725 |
| *SORBS2* | 8470 | NM_003603 | -1.133 |
| *SORL1* | 6653 | NM_003105 | -1.231 |
| *SOS2* | 6655 | NM_006939 | -1.011 |
| *SPAG9* | 9043 | NM_172345 | -1.102 |
| *SPATA18* | 132671 | NM_145263 | -2.61 |
| *SPFH1* | 10613 | NM_006459 | -1.703 |
| *SPFH1* | 10613 | AK127017 | -1.509 |
| *SPG20* | 23111 | NM_015087 | -1.115 |
| *SPG20* | 23111 | BC047083 | -1.695 |
| *SPIRE1* | 56907 | BC016825 | -1.98 |
| *SPIRE1* | 56907 | AL833817 | -1.486 |
| *SPIRE1* | 56907 | NM_020148 | -1.912 |
| *SPP2* | 6694 | BC069401 | -2.948 |
| *SPP2* | 6694 | NM_006944 | -2.712 |
| *SPRED2* | 200734 | NM_181784 | -1.053 |
| *SPRY2* | 10253 | NM_005842 | -1.883 |
| *SPRY2* | 10253 | BC004205 | -1.599 |
| *SRD5A2* | 6716 | BC112252 | -4.203 |
| *SRD5A2* | 6716 | NM_000348 | -2.916 |
| *SRPX* | 8406 | NM_006307 | -2.765 |
| *SSFA2* | 6744 | NM_006751 | -1.134 |
| *ST3GAL6* | 10402 | NM_006100 | -2.105 |
| *ST6GAL2* | 84620 | AB058780 | -2.073 |
| *STAB2* | 55576 | NM_017564 | -3.332 |
| *STARD5* | 80765 | BC004365 | -1.052 |
| *STEAP3* | 55240 | BC042150 | -1.726 |
| *STEAP3* | 55240 | AF262322 | -1.67 |
| *STEAP4* | 79689 | NM_024636 | -1.904 |
| *STEAP4* | 79689 | BC020600 | -1.525 |
| *STK40* | 83931 | NM_032017 | -1.004 |
| *STX11* | 8676 | NM_003764 | -1.209 |
| *STYX* | 6815 | NM_145251 | -1.427 |
| *SUCNR1* | 56670 | NM_033050 | -1.761 |
| *SULF2* | 55959 | BC110539 | -1.835 |
| *SULF2* | 55959 | AY358461 | -1.519 |
| *SYNE1* | 23345 | NM_015293 | -1.101 |
| *SYT9* | 143425 | NM_175733 | -2.981 |
| *SYT9* | 143425 | BC029605 | -2.095 |
| *SYTL3* | 94120 | BC100828 | -1.225 |
| *SYTL3* | 94120 | BC100829 | -1.247 |
| *SYTL5* | 94122 | BX647688 | -1.719 |
| *TACSTD1* | 4072 | BC014785 | -2.473 |
| *TAL1* | 6886 | NM_003189 | -1.077 |
| *TAT* | 6898 | NM_000353 | -1.608 |
| *TBC1D23* | 55773 | BX648032 | -1.003 |
| *TBRG1* | 84897 | NM_032811 | -1.478 |
| *TBRG1* | 84897 | BC109269 | -1.221 |
| *TBX15* | 6913 | AK127536 | -2.065 |
| *TBX15* | 6913 | NM_152380 | -1.705 |
| *TBXA2R* | 6915 | NM_001060 | -1.274 |
| *TBXA2R* | 6915 | NM_201636 | -1.266 |
| *TBXA2R* | 6915 | U11271 | -1.374 |
| *TBXA2R* | 6915 | BC074750 | -1.464 |
| *TBXAS1* | 6916 | NM_001061 | -1.489 |
| *TCF21* | 6943 | NM_003206 | -1.368 |
| *TCF21* | 6943 | NM_198392 | -1.073 |
| *TCTEX1D1* | 200132 | NM_152665 | -1.274 |
| *TCTEX1D1* | 200132 | BC106885 | -1.522 |
| *TDO2* | 6999 | NM_005651 | -1.264 |
| *TECTA* | 7007 | NM_005422 | -1.432 |
| *TFPI2* | 7980 | NM_006528 | -2.918 |
| *TGFA* | 7039 | NM_003236 | -1.274 |
| *TGFBR3* | 7049 | L07594 | -1.002 |
| *THBD* | 7056 | BC035602 | -1.852 |
| *TIAM1* | 7074 | NM_003253 | -2.147 |
| *TIMD4* | 91937 | NM_138379 | -2.241 |
| *TINAGL1* | 64129 | BC064633 | -1.292 |
| *TJP2* | 9414 | BC027592 | -1.115 |
| *TJP2* | 9414 | NM_004817 | -1.059 |
| *TLOC1* | 7095 | BC012035 | -1.232 |
| *TLOC1* | 7095 | AK057365 | -1.347 |
| *TLR3* | 7098 | NM_003265 | -1.66 |
| *TLR4* | 7099 | NM_138557 | -1.48 |
| *TLR4* | 7099 | NM_003266 | -1.802 |
| *TLR4* | 7099 | NM_138554 | -1.332 |
| *TMEM100* | 55273 | NM_018286 | -1.486 |
| *TMEM100* | 55273 | BC010128 | -1.216 |
| *TMEM12* | 119467 | NM_152311 | -3.243 |
| *TMEM125* | 128218 | NM_144626 | -1.259 |
| *TMEM133* | 83935 | BC093843 | -1.243 |
| *TMEM16A* | 55107 | BC033036 | -1.782 |
| *TMEM16A* | 55107 | BC027590 | -1.366 |
| *TMEM25* | 84866 | AK027305 | -1.717 |
| *TMEM26* | 219623 | NM_178505 | -1.242 |
| *TMEM27* | 57393 | BC015099 | -2.466 |
| *TMEM30A* | 55754 | BC009006 | -1.181 |
| *TMEM30B* | 161291 | BC101726 | -1.555 |
| *TMEM30B* | 161291 | NM_001017970 | -1.282 |
| *TMEM45A* | 55076 | BC040355 | -1.996 |
| *TMEM45A* | 55076 | NM_018004 | -2.089 |
| *TMEM56* | 148534 | NM_152487 | -2.34 |
| *TMEM56* | 148534 | BC108263 | -1.469 |
| *TMEM57* | 55219 | AY845035 | -1.343 |
| *TMPRSS2* | 7113 | NM_005656 | -1.462 |
| *TMPRSS6* | 164656 | BC039082 | -1.382 |
| *TMPRSS6* | 164656 | CR456446 | -1.141 |
| *TNFRSF10B* | 8795 | BC001281 | -1 |
| *TNFRSF10D* | 8793 | BC052270 | -1.656 |
| *TNFRSF10D* | 8793 | NM_003840 | -1.226 |
| *TNFRSF11B* | 4982 | BC030155 | -1.475 |
| *TNFSF11* | 8600 | NM_003701 | -1.498 |
| *TNFSF11* | 8600 | BC074890 | -1.244 |
| *TOR1AIP1* | 26092 | BC023247 | -1.261 |
| *TPD52L1* | 7164 | NM_001003396 | -1.464 |
| *TPD52L1* | 7164 | NM_001003395 | -1.036 |
| *TPD52L1* | 7164 | NM_003287 | -1.07 |
| *TPMT* | 7172 | BC009596 | -1.193 |
| *TPMT* | 7172 | BC005339 | -1.151 |
| *TPPP* | 11076 | NM_007030 | -1.474 |
| *TRAM2* | 9697 | NM_012288 | -1.14 |
| *TREH* | 11181 | BC109206 | -2.483 |
| *TREH* | 11181 | NM_007180 | -2.302 |
| *TRIB1* | 10221 | NM_025195 | -1.115 |
| *TRIM10* | 10107 | NM_006778 | -1.102 |
| *TRPV4* | 59341 | DQ059644 | -2.349 |
| *TRPV4* | 59341 | AF263523 | -1.952 |
| *TRPV4* | 59341 | DQ059646 | -2.364 |
| *TRPV4* | 59341 | NM_021625 | -2.083 |
| *TSHR* | 7253 | NM_000369 | -1.254 |
| *TSPAN12* | 23554 | NM_012338 | -1.422 |
| *TSPAN12* | 23554 | BC031265 | -1.383 |
| *TSPAN7* | 7102 | D10653 | -1.594 |
| *TSPAN7* | 7102 | NM_004615 | -1.488 |
| *TSPAN9* | 10867 | BC071881 | -1.085 |
| *TSPYL5* | 85453 | BC045630 | -1.818 |
| *TSPYL5* | 85453 | NM_033512 | -1.818 |
| *TUBE1* | 51175 | NM_016262 | -2.475 |
| *TUBE1* | 51175 | BC031101 | -2.597 |
| *TXK* | 7294 | NM_003328 | -1.224 |
| *UGP2* | 7360 | NM_001001521 | -1.264 |
| *ULK2* | 9706 | BC034988 | -1.77 |
| *UNC93A* | 54346 | BC098248 | -1.564 |
| *UNC93A* | 54346 | NM_018974 | -1.235 |
| *UNQ1940* | 389558 | NM_205855 | -3.461 |
| *UROC1* | 131669 | AB075869 | -2.922 |
| *UROC1* | 131669 | NM_144639 | -2.471 |
| *USP38* | 84640 | BC039115 | -1.385 |
| *USP43* | 124739 | AK090821 | -1.32 |
| *USP9Y* | 8287 | NM_004654 | -1.766 |
| *UTY* | 7404 | NM_182659 | -1.596 |
| *UTY* | 7404 | NM_182660 | -1.243 |
| *UTY* | 7404 | NM_007125 | -1.703 |
| *VIPR1* | 7433 | BC064424 | -3.064 |
| *VIPR1* | 7433 | NM_004624 | -3.207 |
| *VIPR1* | 7433 | X75299 | -3.29 |
| *VMO1* | 284013 | BC104194 | -1.325 |
| *VMO1* | 284013 | NM_182566 | -1.08 |
| *VMO1* | 284013 | BC038467 | -1.386 |
| *VNN1* | 8876 | BC096265 | -1.848 |
| *VNN1* | 8876 | NM_004666 | -1.475 |
| *VPS37B* | 79720 | NM_024667 | -1.217 |
| *VSIG4* | 11326 | NM_007268 | -2.426 |
| *VSIG4* | 11326 | AY358341 | -2.08 |
| *WASF3* | 10810 | NM_006646 | -2.069 |
| *WDFY1* | 57590 | NM_020830 | -1.297 |
| *WDR23* | 80344 | AF130070 | -1.154 |
| *WDR23* | 80344 | BX247970 | -1.126 |
| *WDR66* | 144406 | NM_144668 | -2.112 |
| *WDR66* | 144406 | BC036233 | -1.956 |
| *WDR72* | 256764 | BC101614 | -1.692 |
| *WNT11* | 7481 | NM_004626 | -2.427 |
| *WNT11* | 7481 | BC074791 | -2.014 |
| *WNT11* | 7481 | AK075540 | -2.314 |
| *WWC2* | 80014 | AK126057 | -1.093 |
| *WWC2* | 80014 | BX647704 | -1.033 |
| *WWC2* | 80014 | BC053873 | -1.053 |
| *XLKD1* | 10894 | NM_006691 | -3.729 |
| *YPEL2* | 388403 | NM_001005404 | -1.028 |
| *ZA20D2* | 7763 | BC027707 | -1.57 |
| *ZADH2* | 284273 | NM_175907 | -1.193 |
| *ZBED1* | 9189 | BC015030 | -1.053 |
| *ZFP1* | 162239 | NM_153688 | -1.931 |
| *ZFP3* | 124961 | BX647638 | -1.651 |
| *ZFP3* | 124961 | NM_153018 | -1.82 |
| *ZFP36L2* | 678 | NM_006887 | -1.182 |
| *ZFPM2* | 23414 | BC109222 | -1.263 |
| *ZG16* | 123887 | NM_152338 | -2.05 |
| *ZNF295* | 49854 | NM_020727 | -1.327 |
| *ZNF295* | 49854 | BC063290 | -1.106 |
| *ZNF334* | 55713 | NM_018102 | -1.395 |
| *ZNF533* | 151126 | BC048123 | -1.938 |
| *ZNF533* | 151126 | BC038422 | -2.227 |
| *ZNF533* | 151126 | NM_152520 | -1.849 |
| *ZRANB1* | 54764 | NM_017580 | -1.317 |
