# Supplementary material for: DNA methylation and gene expression profiles show novel regulatory pathways in hepatocellular carcinoma
Source: Clin Epigenetics. 2015 Apr 14;7(1):43. doi: 10.1186/s13148-015-0077-1 (PMC4419480; doi:10.1186/s13148-015-0077-1)
Supplement: Additional file 4: Table S4. — Induced genes in HCC tissue: 1,027 transcripts corresponding to 668 induced genes in HCC as compared to cancer-free tissue (p.value.adj < 0.05). [file 13148_2015_77_MOESM4_ESM.doc]

| **Table S4 - INDUCED GENES IN HCC TISSUE**  1027 transcripts corresponding to 668 induced genes in HCC tissue (p.value.adj<0.05) | | | |
| --- | --- | --- | --- |
| **Gene symbol** | **Gene ID** | **GenBank accession number** | **log2 fold change** |
| *ABCC4* | 10257 | NM_005845 | 1.809 |
| *ABCC4* | 10257 | BC041560 | 1.272 |
| *ABCC5* | 10057 | NM_005688 | 1.524 |
| *ABCC5* | 10057 | NM_001023587 | 1.103 |
| *ACSL4* | 2182 | NM_004458 | 2.058 |
| *ACTBL1* | 23784 | NM_001004053 | 1.692 |
| *ACTL8* | 81569 | NM_030812 | 2.826 |
| *ADAMDEC1* | 27299 | NM_014479 | 2.083 |
| *ADM2* | 79924 | NM_024866 | 1.082 |
| *ANKRD13B* | 124930 | BC062597 | 1.206 |
| *ANKRD13B* | 124930 | NM_152345 | 1.083 |
| *ANKRD21* | 317754 | NM_174981 | 1.509 |
| *ANKRD27* | 84079 | BC050529 | 1.058 |
| *ANKRD27* | 84079 | BC045605 | 1.236 |
| *ANKRD27* | 84079 | NM_032139 | 1.05 |
| *ANKRD29* | 147463 | BC030622 | 1.23 |
| *ANKRD29* | 147463 | AK057782 | 1.261 |
| *ANLN* | 54443 | NM_018685 | 2.817 |
| *APIN* | 54959 | NM_017855 | 1.673 |
| *ARHGAP11A* | 9824 | NM_014783 | 1.96 |
| *ARHGAP11A* | 9824 | NM_199357 | 1.867 |
| *ARHGAP11A* | 9824 | BC039563 | 1.566 |
| *ARL15* | 54622 | NM_019087 | 1.244 |
| *ARMC1* | 55156 | NM_018120 | 1.107 |
| *ASF1B* | 55723 | NM_018154 | 1.573 |
| *ASPM* | 259266 | NM_018136 | 2.606 |
| *ASPM* | 259266 | AY971957 | 2.581 |
| *ASPM* | 259266 | BC034607 | 2.543 |
| *ASRGL1* | 80150 | BC006267 | 1.669 |
| *ASRGL1* | 80150 | NM_025080 | 1.801 |
| *ASRGL1* | 80150 | BC093070 | 1.794 |
| *ASRGL1* | 80150 | BC064963 | 1.802 |
| *ATP6V1C1* | 528 | NM_001007254 | 1.493 |
| *AURKA* | 6790 | NM_003600 | 1.634 |
| *AURKA* | 6790 | BC002499 | 1.584 |
| *AURKB* | 9212 | NM_004217 | 2.387 |
| *AURKB* | 9212 | BC013300 | 2.251 |
| *AZI1* | 22994 | NM_001009811 | 1.349 |
| *B4GALNT1* | 2583 | AK127448 | 2.295 |
| *BAIAP2L2* | 80115 | NM_025045 | 1.864 |
| *BAIAP2L2* | 80115 | AY358164 | 1.782 |
| *BARD1* | 580 | NM_000465 | 1.241 |
| *BAX* | 581 | NM_004324 | 1.143 |
| *BCAS4* | 55653 | NM_017843 | 1.276 |
| *BCL2L12* | 83596 | NM_052842 | 1.032 |
| *BIRC5* | 332 | NM_001012270 | 3.009 |
| *BIRC5* | 332 | BC008718 | 2.617 |
| *BIRC5* | 332 | AY927772 | 1.603 |
| *BIRC5* | 332 | AB154416 | 1.38 |
| *BLM* | 641 | NM_000057 | 1.573 |
| *BOLA2* | 552900 | NM_001031827 | 1.552 |
| *BOLA2* | 552900 | NM_001031833 | 1.135 |
| *BOLA3* | 388962 | NM_212552 | 1.156 |
| *BRRN1* | 23397 | BC024211 | 2.017 |
| *BRUNOL6* | 60677 | NM_052840 | 1.025 |
| *BUB1* | 699 | NM_004336 | 2.807 |
| *BUB1B* | 701 | NM_001211 | 2.224 |
| *C12orf24* | 29902 | BC020967 | 1.202 |
| *C12orf27* | 283460 | AK096009 | 1.17 |
| *C12orf48* | 55010 | NM_017915 | 1.756 |
| *C12orf48* | 55010 | BC098313 | 1.322 |
| *C12orf48* | 55010 | BC099844 | 1.487 |
| *C12orf48* | 55010 | BC099734 | 1.351 |
| *C13orf3* | 221150 | BC048988 | 1.628 |
| *C13orf3* | 221150 | NM_145061 | 2.089 |
| *C15orf20* | 80119 | NM_025049 | 1.82 |
| *C15orf20* | 80119 | AB185927 | 1.063 |
| *C15orf42* | 90381 | NM_152259 | 1.927 |
| *C16orf59* | 80178 | NM_025108 | 1.363 |
| *C16orf59* | 80178 | AK128408 | 1.328 |
| *C18orf24* | 220134 | BC015706 | 3.125 |
| *C18orf24* | 220134 | NM_001039535 | 2.99 |
| *C18orf54* | 162681 | NM_173529 | 1.02 |
| *C19orf40* | 91442 | BC010170 | 1.167 |
| *C19orf40* | 91442 | BC003535 | 1.132 |
| *C19orf40* | 91442 | NM_152266 | 1.128 |
| *C1orf104* | 284618 | NM_173639 | 1.113 |
| *C1orf104* | 284618 | AK125510 | 1.022 |
| *C1orf155* | 91687 | NM_033319 | 1.13 |
| *C1orf155* | 91687 | BC007071 | 1.368 |
| *C1orf2* | 10712 | BC010471 | 1.042 |
| *C1orf2* | 10712 | BC017262 | 1.028 |
| *C1orf31* | 388753 | NM_001012985 | 1.188 |
| *C1orf34* | 22996 | AF007170 | 2.066 |
| *C1orf34* | 22996 | BC028374 | 1.801 |
| *C1orf79* | 85028 | BC022205 | 1.083 |
| *C1orf79* | 85028 | XM_934809 | 1.007 |
| *C20orf42* | 55612 | NM_017671 | 1.921 |
| *C22orf18* | 79019 | BC007495 | 2.838 |
| *C22orf18* | 79019 | BC000705 | 3.121 |
| *C22orf18* | 79019 | NM_001002876 | 3.045 |
| *C22orf18* | 79019 | AK123479 | 1.699 |
| *C2orf27* | 29798 | NM_013310 | 1.178 |
| *C2orf27* | 29798 | BC093716 | 1.009 |
| *C3orf24* | 115795 | NM_173472 | 1.097 |
| *C3orf32* | 51066 | NM_015931 | 2.382 |
| *C3orf32* | 51066 | BC052614 | 2.613 |
| *C3orf50* | 93556 | XM_376284 | 1.495 |
| *C3orf50* | 93556 | XM_934886 | 1.054 |
| *C5orf16* | 285613 | NM_173828 | 1.276 |
| *C6orf117* | 112609 | NM_138409 | 1.739 |
| *C6orf173* | 387103 | NM_001012507 | 2.72 |
| *C6orf26* | 401251 | NM_001039651 | 1.175 |
| *C6orf88* | 441123 | XM_496791 | 1.023 |
| *C7orf13* | 129790 | NM_032625 | 1.239 |
| *C7orf13* | 129790 | BC070228 | 1.03 |
| *C8orf32* | 55093 | BC008781 | 1.291 |
| *C8orf32* | 55093 | NM_018024 | 1.161 |
| *C8orf36* | 286053 | NM_173685 | 1.288 |
| *C8orf39* | 55472 | AF116672 | 1.304 |
| *C8orf59* | 401466 | XM_933998 | 1.106 |
| *C8orf59* | 401466 | XM_929832 | 1.485 |
| *C8orf59* | 401466 | BC032347 | 1.208 |
| *C8orf76* | 84933 | BC012379 | 2.034 |
| *C8orf76* | 84933 | NM_032847 | 1.146 |
| *C9orf100* | 84904 | NM_032818 | 1.135 |
| *C9orf100* | 84904 | NM_001031728 | 1.909 |
| *C9orf100* | 84904 | BC038673 | 1.718 |
| *C9orf140* | 89958 | NM_178448 | 1.808 |
| *CA12* | 771 | NM_001218 | 3.804 |
| *CA12* | 771 | BC011691 | 3.182 |
| *CA12* | 771 | BC001012 | 3.598 |
| *CA12* | 771 | BC000278 | 2.908 |
| *CACNB1* | 782 | NM_000723 | 1.075 |
| *CACNB3* | 784 | NM_000725 | 1.141 |
| *CACYBP* | 27101 | NM_001007214 | 1.357 |
| *CAP2* | 10486 | BC008481 | 1.667 |
| *CAP2* | 10486 | NM_006366 | 2.155 |
| *CAPG* | 822 | NM_001747 | 1.524 |
| *CAPN10* | 11132 | NM_023089 | 1.013 |
| *CBX8* | 57332 | NM_020649 | 1.533 |
| *CCDC11* | 220136 | NM_145020 | 1.542 |
| *CCDC34* | 91057 | NM_030771 | 1.232 |
| *CCDC64* | 92558 | NM_207311 | 1.999 |
| *CCDC99* | 54908 | NM_017785 | 1.256 |
| *CCL20* | 6364 | NM_004591 | 2.713 |
| *CCNA2* | 890 | NM_001237 | 2.132 |
| *CCNB1* | 891 | NM_031966 | 3.137 |
| *CCNB1* | 891 | BC006510 | 3.102 |
| *CCNB2* | 9133 | BC105086 | 3 |
| *CCNB2* | 9133 | NM_004701 | 3.214 |
| *CCNE1* | 898 | NM_001238 | 2.085 |
| *CCNF* | 899 | NM_001761 | 1.608 |
| *CD109* | 135228 | NM_133493 | 1.636 |
| *CD19* | 930 | AK130657 | 1.221 |
| *CD200* | 4345 | NM_001004196 | 2.104 |
| *CD34* | 947 | BX538076 | 1.394 |
| *CD34* | 947 | NM_001773 | 1.496 |
| *CDC2* | 983 | NM_001786 | 2.468 |
| *CDC2* | 983 | NM_033379 | 2.704 |
| *CDC2* | 983 | BC107750 | 2.597 |
| *CDC20* | 991 | NM_001255 | 3.24 |
| *CDC25A* | 993 | NM_001789 | 1.846 |
| *CDC25C* | 995 | NM_001790 | 3.867 |
| *CDC45L* | 8318 | NM_003504 | 1.419 |
| *CDC6* | 990 | BC025232 | 2.163 |
| *CDC6* | 990 | NM_001254 | 2.058 |
| *CDC7* | 8317 | NM_003503 | 1.073 |
| *CDC7* | 8317 | BC110526 | 1.117 |
| *CDCA1* | 83540 | NM_031423 | 1.645 |
| *CDCA2* | 157313 | BC104450 | 1.743 |
| *CDCA3* | 83461 | NM_031299 | 2.169 |
| *CDCA4* | 55038 | NM_145701 | 1.422 |
| *CDCA4* | 55038 | NM_017955 | 1.217 |
| *CDCA5* | 113130 | NM_080668 | 2.779 |
| *CDCA8* | 55143 | NM_018101 | 2.38 |
| *CDCA8* | 55143 | BC001651 | 2.262 |
| *CDH24* | 64403 | AL137477 | 1.301 |
| *CDH24* | 64403 | AK025342 | 1.295 |
| *CDKN2A* | 1029 | L27211 | 2.609 |
| *CDKN2A* | 1029 | NM_058195 | 2.769 |
| *CDKN2A* | 1029 | U26727 | 2.565 |
| *CDKN2A* | 1029 | BC021998 | 2.883 |
| *CDKN2A* | 1029 | NM_058197 | 1.732 |
| *CDKN2A* | 1029 | NM_000077 | 2.808 |
| *CDKN2C* | 1031 | NM_001262 | 1.486 |
| *CDKN2C* | 1031 | NM_078626 | 1.035 |
| *CDKN3* | 1033 | BC064965 | 3.253 |
| *CDKN3* | 1033 | NM_005192 | 2.957 |
| *CDT1* | 81620 | BC000137 | 1.457 |
| *CDT1* | 81620 | NM_030928 | 1.415 |
| *CDT1* | 81620 | BC009410 | 1.637 |
| *CELSR3* | 1951 | NM_001407 | 2.029 |
| *CENPA* | 1058 | NM_001809 | 3.042 |
| *CENPA* | 1058 | BC002703 | 2.72 |
| *CENPE* | 1062 | NM_001813 | 1.728 |
| *CENPE* | 1062 | Z15005 | 1.55 |
| *CENPF* | 1063 | NM_016343 | 3.656 |
| *CENPJ* | 55835 | BC113110 | 1.202 |
| *CENPJ* | 55835 | BC113111 | 1.025 |
| *CEP152* | 22995 | NM_014985 | 1.431 |
| *CEP55* | 55165 | NM_018131 | 1.535 |
| *CGREF1* | 10669 | NM_006569 | 1.924 |
| *CHAF1A* | 10036 | BC067093 | 1.162 |
| *CHAF1A* | 10036 | NM_005483 | 1.116 |
| *CHEK1* | 1111 | NM_001274 | 1.162 |
| *CHEK2* | 11200 | AY551298 | 1.03 |
| *CHEK2* | 11200 | NM_145862 | 1.079 |
| *CHEK2* | 11200 | NM_001005735 | 1.155 |
| *CHEK2* | 11200 | AY551303 | 1.105 |
| *CHRM3* | 1131 | NM_000740 | 1.98 |
| *CKS2* | 1164 | NM_001827 | 1.581 |
| *CKS2* | 1164 | BC006458 | 1.485 |
| *CLCC1* | 23155 | AB018304 | 1.436 |
| *CLSPN* | 63967 | BC113116 | 1.325 |
| *CNTNAP1* | 8506 | NM_003632 | 1.213 |
| *COL15A1* | 1306 | NM_001855 | 2.181 |
| *COL5A3* | 50509 | NM_015719 | 1.451 |
| *COLEC12* | 81035 | NM_130386 | 1.273 |
| *COLEC12* | 81035 | BC060789 | 1.602 |
| *COMP* | 1311 | NM_000095 | 1.438 |
| *COX4I2* | 84701 | NM_032609 | 1.404 |
| *COX7B2* | 170712 | BC107855 | 2.236 |
| *COX7B2* | 170712 | NM_130902 | 2.361 |
| *COX7B2* | 170712 | BC035923 | 2.449 |
| *CPNE5* | 57699 | NM_020939 | 1.042 |
| *CSAG1* | 158511 | BC059947 | 2.683 |
| *CSAG1* | 158511 | NM_153479 | 2.597 |
| *CSAG2* | 9598 | NM_004909 | 2.955 |
| *CSAG3A* | 389903 | NM_203311 | 2.965 |
| *CSE1L* | 1434 | NM_177436 | 1.269 |
| *CSMD1* | 64478 | NM_033225 | 2.029 |
| *CTHRC1* | 115908 | NM_138455 | 3.184 |
| *CTLA4* | 1493 | BC074893 | 1.372 |
| *CTLA4* | 1493 | BC074842 | 1.147 |
| *CTLA4* | 1493 | BC069566 | 1.257 |
| *CTLA4* | 1493 | NM_001037631 | 2.215 |
| *CTLA4* | 1493 | NM_005214 | 1.816 |
| *CXCL10* | 3627 | NM_001565 | 1.576 |
| *CYP17A1* | 1586 | NM_000102 | 3.246 |
| *CYP17A1* | 1586 | BC063388 | 2.966 |
| *DAPK2* | 23604 | BC114506 | 1.025 |
| *DBF4* | 10926 | AB028070 | 1.004 |
| *DBNDD1* | 79007 | AK022644 | 1.434 |
| *DCC1* | 79075 | BC001531 | 1.855 |
| *DCC1* | 79075 | NM_024094 | 2.044 |
| *DDEF1* | 50807 | BC020631 | 1.276 |
| *DDEF1* | 50807 | NM_018482 | 1.258 |
| *DDX39* | 10212 | NM_005804 | 1.12 |
| *DDX39* | 10212 | BC032128 | 1.129 |
| *DDX39* | 10212 | NM_138998 | 1.008 |
| *DEPDC1* | 55635 | NM_017779 | 3.003 |
| *DEPDC1* | 55635 | AK000490 | 2.355 |
| *DEPDC1B* | 55789 | NM_018369 | 1.554 |
| *DKFZp686J0529* | 388468 | XM_932259 | 1.185 |
| *DKFZp686J0529* | 388468 | XM_496269 | 1.299 |
| *DKFZp762E1312* | 55355 | NM_018410 | 2.773 |
| *DLG7* | 9787 | NM_014750 | 1.997 |
| *DMP1* | 1758 | NM_004407 | 1.433 |
| *DNAJC6* | 9829 | NM_014787 | 1.979 |
| *DNM3* | 26052 | NM_015569 | 1.418 |
| *DNMT3B* | 1789 | DQ321787 | 1.11 |
| *DTL* | 51514 | BC033297 | 2.314 |
| *DTL* | 51514 | NM_016448 | 2.519 |
| *DTL* | 51514 | BC033540 | 2.159 |
| *DTL* | 51514 | AK027651 | 1.677 |
| *DTNA* | 1837 | NM_001391 | 1.763 |
| *DTNA* | 1837 | NM_001390 | 2.001 |
| *DTNA* | 1837 | NM_001392 | 1.342 |
| *DUT* | 1854 | NM_001025248 | 1.052 |
| *DUXA* | 503835 | NM_001012729 | 2.939 |
| *E2F1* | 1869 | BC050369 | 1.742 |
| *E2F1* | 1869 | NM_005225 | 1.694 |
| *E2F2* | 1870 | NM_004091 | 2.078 |
| *E2F2* | 1870 | BC053676 | 1.839 |
| *E2F8* | 79733 | BC028244 | 2.459 |
| *E2F8* | 79733 | NM_024680 | 2.05 |
| *EBF* | 1879 | BC038805 | 1.423 |
| *ECT2* | 1894 | BC112086 | 1.894 |
| *ECT2* | 1894 | NM_018098 | 2.006 |
| *EDIL3* | 10085 | BX648583 | 2.158 |
| *EFNA4* | 1945 | NM_182689 | 1.148 |
| *EFNA4* | 1945 | BC107483 | 1.21 |
| *EFNA4* | 1945 | NM_005227 | 1.129 |
| *EHMT2* | 10919 | AK056936 | 1.162 |
| *EIF5A2* | 56648 | BC036072 | 1.689 |
| *EME1* | 146956 | NM_152463 | 3.469 |
| *ENAH* | 55740 | NM_001008493 | 1.312 |
| *ENAH* | 55740 | AF519769 | 1.062 |
| *ENAH* | 55740 | BC095481 | 1.102 |
| *ENPP6* | 133121 | NM_153343 | 1.249 |
| *EPS8L3* | 79574 | NM_133181 | 3.083 |
| *EPS8L3* | 79574 | NM_024526 | 3.054 |
| *ESM1* | 11082 | NM_007036 | 2.047 |
| *ESM1* | 11082 | AJ973643 | 1.719 |
| *ESPL1* | 9700 | NM_012291 | 1.881 |
| *EXO1* | 9156 | NM_003686 | 2.725 |
| *EXOSC4* | 54512 | NM_019037 | 1.013 |
| *EZH2* | 2146 | NM_004456 | 1.651 |
| *FABP5* | 2171 | NM_001444 | 1.058 |
| *FABP5L3* | 220832 | XM_165511 | 1.162 |
| *FAM112B* | 121355 | NM_144594 | 3.002 |
| *FAM24B* | 196792 | NM_152644 | 1.092 |
| *FAM54A* | 113115 | NM_138419 | 1.639 |
| *FAM64A* | 54478 | NM_019013 | 1.44 |
| *FAM72A* | 389835 | NM_207418 | 2.066 |
| *FAM72A* | 389835 | BC046199 | 2.028 |
| *FAM72A* | 389835 | BC035696 | 1.53 |
| *FAM7A2* | 89839 | XM_931039 | 1.892 |
| *FAM7A2* | 89839 | XM_926156 | 1.383 |
| *FAM7A2* | 89839 | XM_931028 | 1.705 |
| *FAM7A2* | 89839 | NM_001039841 | 1.458 |
| *FAM7A2* | 89839 | XM_931023 | 1.578 |
| *FAM7A2* | 89839 | XM_931017 | 1.567 |
| *FAM7A2* | 89839 | XM_931045 | 1.51 |
| *FAM7A2* | 89839 | XM_931050 | 1.478 |
| *FAM83D* | 81610 | NM_030919 | 1.861 |
| *FANCA* | 2175 | NM_000135 | 1.054 |
| *FANCA* | 2175 | NM_001018112 | 1.17 |
| *FANCD2* | 2177 | NM_001018115 | 1.787 |
| *FANCD2* | 2177 | NM_033084 | 1.693 |
| *FANCG* | 2189 | BC011623 | 1.078 |
| *FANCG* | 2189 | NM_004629 | 1.077 |
| *FBN3* | 84467 | AK131282 | 1.604 |
| *FBXL18* | 80028 | BC024256 | 1.195 |
| *FBXO32* | 114907 | NM_058229 | 2.086 |
| *FHAD1* | 114827 | XM_934890 | 1.382 |
| *FKSG14* | 64105 | NM_022145 | 1.812 |
| *FLJ12684* | 79584 | NM_024534 | 1.299 |
| *FLJ13744* | 80078 | AK023806 | 2.965 |
| *FLJ20097* | 55610 | AK000104 | 1.269 |
| *FLJ20105* | 54821 | NM_001009954 | 1.484 |
| *FLJ20280* | 54876 | NM_017741 | 1.014 |
| *FLJ20433* | 54932 | BC065002 | 1.388 |
| *FLJ22531* | 79703 | NM_024650 | 1.228 |
| *FLJ22624* | 79866 | BC056876 | 1.018 |
| *FLJ22795* | 80154 | NM_025084 | 1.574 |
| *FLJ22795* | 80154 | AF316855 | 1.539 |
| *FLJ22795* | 80154 | BC007811 | 1.501 |
| *FLJ35767* | 400629 | NM_207459 | 1.088 |
| *FLJ35767* | 400629 | BC036191 | 1.09 |
| *FLJ36070* | 284358 | AK131427 | 1.218 |
| *FLJ38020* | 644849 | NM_001039775 | 2.038 |
| *FLJ38894* | 646029 | XM_928992 | 2.078 |
| *FLJ39632* | 642477 | XM_925855 | 3.146 |
| *FLJ42291* | 346547 | NM_207367 | 1.837 |
| *FLJ42562* | 400954 | XM_934190 | 1.608 |
| *FLVCR* | 28982 | NM_014053 | 1.906 |
| *FOXD2* | 2306 | NM_004474 | 1.474 |
| *FOXM1* | 2305 | NM_202003 | 1.617 |
| *FOXM1* | 2305 | BC006192 | 1.932 |
| *FOXM1* | 2305 | NM_021953 | 1.799 |
| *FSHPRH1* | 2491 | NM_006733 | 1.129 |
| *FSTL4* | 23105 | BX640845 | 1.47 |
| *FUT2* | 2524 | AY751739 | 1.319 |
| *G6PD* | 2539 | NM_000402 | 1.535 |
| *GABRD* | 2563 | NM_000815 | 2.711 |
| *GAGE5* | 2577 | NM_001475 | 3.696 |
| *GAGE6* | 2578 | NM_001476 | 3.549 |
| *GAGE8* | 26749 | BC018052 | 3.108 |
| *GALIG* | 81625 | NM_194327 | 1.041 |
| *GATS* | 352954 | BC044886 | 1.69 |
| *GLDN* | 342035 | NM_181789 | 1.2 |
| *GLS* | 2744 | NM_014905 | 1.001 |
| *GMNN* | 51053 | BC005389 | 1.479 |
| *GMNN* | 51053 | NM_015895 | 1.624 |
| *GMNN* | 51053 | BC005185 | 1.4 |
| *GNAZ* | 2781 | BC026342 | 2.111 |
| *GNAZ* | 2781 | NM_002073 | 2.412 |
| *GNAZ* | 2781 | BC096828 | 1.441 |
| *GNG4* | 2786 | NM_004485 | 1.423 |
| *GPC3* | 2719 | BC035972 | 3.59 |
| *GPC3* | 2719 | NM_004484 | 3.677 |
| *GPR158* | 57512 | NM_020752 | 2.043 |
| *GPR172A* | 79581 | NM_024531 | 1.061 |
| *GTF2IRD1* | 9569 | NM_005685 | 1.089 |
| *GTSE1* | 51512 | BC006325 | 2.789 |
| *GTSE1* | 51512 | NM_016426 | 2.697 |
| *GTSE1* | 51512 | BC075828 | 2.919 |
| *H2AFX* | 3014 | NM_002105 | 1.378 |
| *H4/o* | 554313 | NM_001034077 | 1.955 |
| *HCAP-G* | 64151 | BC068467 | 3.4 |
| *HDAC11* | 79885 | AL834223 | 1.139 |
| *HELLS* | 3070 | AB102716 | 1.364 |
| *HELLS* | 3070 | AB102720 | 1.288 |
| *HELLS* | 3070 | AB113249 | 1.308 |
| *HELLS* | 3070 | NM_018063 | 1.265 |
| *HEY1* | 23462 | NM_012258 | 1.062 |
| *HEY1* | 23462 | BC001873 | 1.022 |
| *HIST1H1C* | 3006 | NM_005319 | 1.03 |
| *HIST1H1C* | 3006 | BC002649 | 1.037 |
| *HIST1H2AD* | 3013 | NM_021065 | 1.19 |
| *HIST1H2BG* | 8339 | BC082232 | 2.277 |
| *HIST1H2BJ* | 8970 | BC014312 | 1.861 |
| *HIST1H3F* | 8968 | BC096131 | 1.042 |
| *HIST1H3H* | 8357 | BC007518 | 2.379 |
| *HIST1H3H* | 8357 | BC096128 | 1.084 |
| *HIST1H4A* | 8359 | NM_003538 | 1.282 |
| *HIST1H4B* | 8366 | NM_003544 | 1.042 |
| *HIST1H4F* | 8361 | NM_003540 | 1.733 |
| *HIST1H4H* | 8365 | BC010926 | 2.691 |
| *HIST1H4I* | 8294 | BC075806 | 1.46 |
| *HIST1H4I* | 8294 | NM_003495 | 1.047 |
| *HIST1H4K* | 8362 | NM_003541 | 1.036 |
| *HIST2H4* | 8370 | NM_003548 | 1.487 |
| *HKDC1* | 80201 | BC110504 | 2.507 |
| *HKDC1* | 80201 | AK026414 | 2.517 |
| *HKDC1* | 80201 | NM_025130 | 2.531 |
| *HMGA1* | 3159 | BC063434 | 1.031 |
| *HMGA1* | 3159 | NM_002131 | 1.093 |
| *HMGA1* | 3159 | BC071863 | 1.025 |
| *HMGB2* | 3148 | BC100019 | 1.151 |
| *HMGB2* | 3148 | NM_002129 | 1.272 |
| *HMGB2* | 3148 | BC000903 | 1.013 |
| *HMMR* | 3161 | NM_012484 | 2.184 |
| *HMMR* | 3161 | BC108904 | 1.894 |
| *HN1* | 51155 | BC001420 | 1.286 |
| *HN1* | 51155 | BC039343 | 1.111 |
| *HN1* | 51155 | NM_001002032 | 1.246 |
| *HN1* | 51155 | NM_001002033 | 1.224 |
| *HOXD9* | 3235 | NM_014213 | 1.859 |
| *HSF2BP* | 11077 | NM_007031 | 1.395 |
| *HSPA12B* | 116835 | BC110881 | 1.397 |
| *IGF2BP3* | 10643 | NM_006547 | 2.245 |
| *IGSF3* | 3321 | NM_001007237 | 2.252 |
| *ILF3* | 3609 | NM_004516 | 1.014 |
| *INCENP* | 3619 | BC098576 | 1.378 |
| *INPP5F* | 22876 | NM_014937 | 1.109 |
| *IQGAP3* | 128239 | NM_178229 | 3.401 |
| *ITGB3BP* | 23421 | U37139 | 1.084 |
| *KCNJ5* | 3762 | BC069386 | 1.101 |
| *KCNN3* | 3782 | NM_002249 | 1.336 |
| *KHDRBS3* | 10656 | BC032606 | 1.015 |
| *KHDRBS3* | 10656 | NM_006558 | 1.074 |
| *KIAA0101* | 9768 | NM_001029989 | 1.917 |
| *KIAA0101* | 9768 | NM_014736 | 1.524 |
| *KIAA0841* | 23354 | XM_932182 | 1.076 |
| *KIAA1598* | 57698 | NM_018330 | 1.098 |
| *KIAA1666* | 85376 | AL133030 | 1.326 |
| *KIAA1688* | 80728 | NM_025251 | 1.57 |
| *KIAA1794* | 55215 | NM_018193 | 1.848 |
| *KIAA1822L* | 79802 | BC007638 | 1.589 |
| *KIAA1822L* | 79802 | NM_024746 | 1.147 |
| *KIAA1893* | 114787 | NM_052899 | 1.639 |
| *KIF11* | 3832 | NM_004523 | 1.484 |
| *KIF14* | 9928 | NM_014875 | 2.322 |
| *KIF15* | 56992 | NM_020242 | 2.277 |
| *KIF18A* | 81930 | NM_031217 | 1.83 |
| *KIF18A* | 81930 | BC048347 | 1.673 |
| *KIF20A* | 10112 | BC012999 | 2.553 |
| *KIF20A* | 10112 | NM_005733 | 2.654 |
| *KIF23* | 9493 | NM_004856 | 2.087 |
| *KIF23* | 9493 | NM_138555 | 1.501 |
| *KIF2C* | 11004 | NM_006845 | 3.284 |
| *KIF2C* | 11004 | AY026505 | 2.405 |
| *KIF3A* | 11127 | AB209780 | 1.087 |
| *KIF4A* | 24137 | NM_012310 | 2.222 |
| *KIF4A* | 24137 | BC050548 | 2.094 |
| *KIF7* | 374654 | NM_198525 | 1.374 |
| *KIFC1* | 3833 | NM_002263 | 3.673 |
| *KIFC2* | 90990 | NM_145754 | 1.193 |
| *KNTC1* | 9735 | NM_014708 | 1.334 |
| *KNTC2* | 10403 | NM_006101 | 2.737 |
| *KNTC2* | 10403 | BC035617 | 2.231 |
| *KPNA2* | 3838 | BC067848 | 1.027 |
| *KPNA2* | 3838 | BC005978 | 1.037 |
| *LAMA3* | 3909 | NM_000227 | 1.616 |
| *LAMP3* | 27074 | BC032940 | 1.429 |
| *LARS* | 51520 | BC110084 | 1.143 |
| *LDLRAD1* | 388633 | NM_001010978 | 1.293 |
| *LEF1* | 51176 | AF294627 | 1.512 |
| *LEF1* | 51176 | NM_016269 | 2.22 |
| *LHX9* | 56956 | NM_001014434 | 1.246 |
| *LIG1* | 3978 | BC110622 | 1.05 |
| *LMNB1* | 4001 | NM_005573 | 1.025 |
| *LOC120376* | 120376 | XM_071712 | 1.402 |
| *LOC137886* | 137886 | XM_059929 | 1.306 |
| *LOC137886* | 137886 | XM_933092 | 1.21 |
| *LOC137886* | 137886 | XM_933097 | 1.385 |
| *LOC146909* | 146909 | XM_929923 | 2.012 |
| *LOC146909* | 146909 | XM_085634 | 1.798 |
| *LOC146909* | 146909 | XM_934078 | 1.348 |
| *LOC283050* | 283050 | XM_378238 | 1.214 |
| *LOC283932* | 283932 | NM_175901 | 1.249 |
| *LOC284417* | 284417 | XM_209187 | 1.522 |
| *LOC285643* | 285643 | XM_209695 | 2.151 |
| *LOC285643* | 285643 | XM_934740 | 2.004 |
| *LOC286467* | 286467 | XM_377041 | 1.812 |
| *LOC387895* | 387895 | XM_373553 | 1.505 |
| *LOC387934* | 387934 | XM_370729 | 1.059 |
| *LOC388152* | 388152 | BC096759 | 1.503 |
| *LOC388152* | 388152 | AK074778 | 1.476 |
| *LOC388152* | 388152 | NM_203426 | 1.395 |
| *LOC388814* | 388814 | XM_373926 | 1.211 |
| *LOC389834* | 389834 | NM_001013655 | 1.664 |
| *LOC400879* | 400879 | XM_379029 | 3.171 |
| *LOC400879* | 400879 | BX248778 | 2.45 |
| *LOC400927* | 400927 | XM_930401 | 1.077 |
| *LOC401720* | 401720 | NM_001013690 | 1.681 |
| *LOC402110* | 402110 | NM_001013695 | 1.724 |
| *LOC440080* | 440080 | XM_931790 | 2.006 |
| *LOC440080* | 440080 | XM_927026 | 1.914 |
| *LOC440080* | 440080 | XM_495907 | 2.067 |
| *LOC440080* | 440080 | XM_931817 | 1.841 |
| *LOC440080* | 440080 | XM_931801 | 1.886 |
| *LOC440080* | 440080 | XM_931785 | 1.859 |
| *LOC440080* | 440080 | XM_931806 | 1.979 |
| *LOC440080* | 440080 | XM_931776 | 1.926 |
| *LOC440080* | 440080 | XM_931795 | 1.853 |
| *LOC440080* | 440080 | XM_931813 | 1.761 |
| *LOC440080* | 440080 | XM_931759 | 1.349 |
| *LOC440080* | 440080 | XM_931765 | 1.105 |
| *LOC440157* | 440157 | NM_001013701 | 2.783 |
| *LOC440731* | 440731 | XM_498838 | 1.548 |
| *LOC440731* | 440731 | XM_933697 | 1.406 |
| *LOC440731* | 440731 | XM_933693 | 1.88 |
| *LOC440928* | 440928 | XM_498917 | 1.608 |
| *LOC441282* | 441282 | XM_935381 | 2.442 |
| *LOC441378* | 441378 | XM_499128 | 2.414 |
| *LOC442075* | 442075 | AK092352 | 1.22 |
| *LOC442448* | 442448 | XM_926714 | 2.01 |
| *LOC541471* | 541471 | BC014776 | 1.314 |
| *LOC642419* | 642419 | XM_925937 | 3.619 |
| *LOC642423* | 642423 | XM_931092 | 1.493 |
| *LOC642423* | 642423 | XM_931074 | 1.441 |
| *LOC642571* | 642571 | XM_926058 | 1.609 |
| *LOC642734* | 642734 | XM_931052 | 2.858 |
| *LOC642767* | 642767 | XM_926190 | 1.372 |
| *LOC642809* | 642809 | XM_926216 | 1.086 |
| *LOC642956* | 642956 | XM_926338 | 1.007 |
| *LOC642981* | 642981 | XM_927840 | 1.847 |
| *LOC643275* | 643275 | XM_370692 | 1.017 |
| *LOC643401* | 643401 | XM_932015 | 1.372 |
| *LOC643425* | 643425 | XM_926759 | 2.227 |
| *LOC643518* | 643518 | XM_926832 | 1.233 |
| *LOC643575* | 643575 | XM_926879 | 2.657 |
| *LOC643707* | 643707 | XM_928746 | 1.396 |
| *LOC643980* | 643980 | XM_933068 | 2.021 |
| *LOC644053* | 644053 | XM_927296 | 1.058 |
| *LOC644102* | 644102 | XM_927329 | 1.147 |
| *LOC644200* | 644200 | XM_932039 | 1.359 |
| *LOC644244* | 644244 | XM_932071 | 1.611 |
| *LOC644283* | 644283 | XM_927459 | 1.051 |
| *LOC644404* | 644404 | XM_932171 | 1.336 |
| *LOC644433* | 644433 | XM_927576 | 1.374 |
| *LOC644488* | 644488 | XM_927616 | 1.348 |
| *LOC644530* | 644530 | XM_927658 | 1.4 |
| *LOC644555* | 644555 | XM_927675 | 1.285 |
| *LOC644598* | 644598 | XM_927711 | 1.667 |
| *LOC644979* | 644979 | XM_928051 | 1.042 |
| *LOC645009* | 645009 | XM_934141 | 3.635 |
| *LOC645009* | 645009 | BC081536 | 3.461 |
| *LOC645009* | 645009 | XM_934143 | 3.496 |
| *LOC645009* | 645009 | XM_934146 | 3.153 |
| *LOC645009* | 645009 | XM_929977 | 3.278 |
| *LOC645021* | 645021 | XM_934155 | 3.218 |
| *LOC645043* | 645043 | XM_934184 | 3.29 |
| *LOC645043* | 645043 | XM_934187 | 3.424 |
| *LOC645073* | 645073 | XM_934222 | 3.306 |
| *LOC645083* | 645083 | XM_934235 | 3.91 |
| *LOC645083* | 645083 | XM_934231 | 3.447 |
| *LOC645084* | 645084 | XM_928123 | 1.119 |
| *LOC645093* | 645093 | XM_934239 | 3.219 |
| *LOC645093* | 645093 | XM_934244 | 3.329 |
| *LOC645146* | 645146 | XM_928177 | 1.298 |
| *LOC645200* | 645200 | XM_930432 | 1.733 |
| *LOC645472* | 645472 | XM_928498 | 1.133 |
| *LOC645931* | 645931 | XM_930517 | 1.128 |
| *LOC646014* | 646014 | XM_928978 | 1.049 |
| *LOC646096* | 646096 | XM_929060 | 1.038 |
| *LOC646129* | 646129 | XM_933432 | 2.17 |
| *LOC646469* | 646469 | XM_929391 | 1.042 |
| *LOC646855* | 646855 | XM_929812 | 1.574 |
| *LOC646962* | 646962 | NM_001039792 | 2.15 |
| *LOC647138* | 647138 | XM_930159 | 1.212 |
| *LOC647210* | 647210 | XM_930248 | 1.191 |
| *LOC647248* | 647248 | XM_930293 | 1.694 |
| *LOC647266* | 647266 | XM_934627 | 3.863 |
| *LOC647292* | 647292 | XM_930356 | 1.447 |
| *LOC653088* | 653088 | XM_930735 | 3.659 |
| *LOC653088* | 653088 | XM_925889 | 3.705 |
| *LOC653089* | 653089 | XM_925843 | 1.539 |
| *LOC653089* | 653089 | XM_930650 | 1.912 |
| *LOC653091* | 653091 | XM_926221 | 1.534 |
| *LOC653239* | 653239 | XM_932759 | 1.624 |
| *LOC653239* | 653239 | XM_932765 | 1.636 |
| *LOC653239* | 653239 | XM_932768 | 1.113 |
| *LOC653239* | 653239 | XM_932754 | 1.154 |
| *LOC653269* | 653269 | XM_932975 | 1.387 |
| *LOC653340* | 653340 | XM_935076 | 1.006 |
| *LOC653344* | 653344 | XM_933105 | 1.526 |
| *LOC653344* | 653344 | XM_928727 | 1.398 |
| *LOC653357* | 653357 | XM_928827 | 1.493 |
| *LOC653359* | 653359 | XM_933198 | 1.591 |
| *LOC653359* | 653359 | XM_928812 | 1.393 |
| *LOC653522* | 653522 | XM_927884 | 1.163 |
| *LOC653524* | 653524 | XM_930350 | 1.914 |
| *LOC653530* | 653530 | XM_933705 | 3.47 |
| *LOC653594* | 653594 | XM_928320 | 2.065 |
| *LOC653811* | 653811 | XM_930073 | 1.325 |
| *LOC653820* | 653820 | XM_935476 | 1.982 |
| *LOC653820* | 653820 | XM_930579 | 1.55 |
| *LOC90355* | 90355 | NM_033211 | 1.266 |
| *LOC92312* | 92312 | XM_044166 | 1.283 |
| *LQK1* | 642946 | XM_927142 | 2.033 |
| *LQK1* | 642946 | AY030238 | 1.853 |
| *LQK1* | 642946 | XM_931870 | 1.809 |
| *LRP11* | 84918 | BC025239 | 1.321 |
| *LRP12* | 29967 | BC017381 | 1.028 |
| *LSP1* | 654342 | XM_946374 | 1.49 |
| *LTA* | 4049 | NM_000595 | 1.018 |
| *MAD2L1* | 4085 | BC005945 | 1.725 |
| *MAD2L1* | 4085 | AF394735 | 1.941 |
| *MAD2L1* | 4085 | NM_002358 | 1.734 |
| *MAEL* | 84944 | NM_032858 | 1.642 |
| *MAGEA1* | 4100 | NM_004988 | 4.608 |
| *MAGEA2* | 4101 | BC112158 | 3.598 |
| *MAGEA3* | 4102 | NM_005362 | 3.808 |
| *MAGEA4* | 4103 | NM_001011548 | 2.636 |
| *MAGEA5* | 4104 | BC109187 | 3.997 |
| *MAGEA5* | 4104 | NM_021049 | 1.774 |
| *MAGEA6* | 4105 | NM_005363 | 4.103 |
| *MAGEA8* | 4107 | NM_005364 | 2.906 |
| *MAGEA9* | 4108 | NM_005365 | 1.852 |
| *MAP2* | 4133 | BC038857 | 2.443 |
| *MAP2* | 4133 | U89329 | 1.553 |
| *MAP2* | 4133 | NM_001039538 | 1.862 |
| *MAPK8IP2* | 23542 | NM_012324 | 1.157 |
| *MAPT* | 4137 | NM_005910 | 2.153 |
| *MCHR1* | 2847 | NM_005297 | 1.421 |
| *MCM10* | 55388 | BC009108 | 2.079 |
| *MCM10* | 55388 | NM_018518 | 2.075 |
| *MCM10* | 55388 | BC101727 | 1.66 |
| *MCM6* | 4175 | NM_005915 | 1.093 |
| *MCM8* | 84515 | NM_032485 | 1.418 |
| *MCM8* | 84515 | BC101054 | 1.135 |
| *MCM8* | 84515 | BC080656 | 1.216 |
| *MCM8* | 84515 | BC101055 | 1.251 |
| *MDK* | 4192 | NM_002391 | 2.44 |
| *MDK* | 4192 | BC011704 | 2.427 |
| *MDK* | 4192 | NM_001012333 | 2.409 |
| *MDK* | 4192 | NM_001012334 | 2.408 |
| *MEF2B* | 4207 | BC004449 | 1.1 |
| *MELK* | 9833 | NM_014791 | 2.799 |
| *MEOX2* | 4223 | BC017021 | 1.386 |
| *MGAT4A* | 11320 | NM_012214 | 1.335 |
| *MGC16597* | 339230 | XM_375500 | 1.123 |
| *MGC22793* | 221908 | NM_145030 | 1.138 |
| *MGC24665* | 116028 | BC031016 | 1.567 |
| *MGC24665* | 116028 | BC039361 | 1.537 |
| *MGC24665* | 116028 | NM_152308 | 1.668 |
| *MGC40489* | 146880 | XM_935202 | 1.005 |
| *MGC4677* | 112597 | NM_052871 | 1.42 |
| *MGC4677* | 112597 | BC009508 | 1.344 |
| *MICB* | 4277 | NM_005931 | 1.544 |
| *MICB* | 4277 | BC044218 | 1.39 |
| *MKRN3* | 7681 | BC044639 | 2.027 |
| *MKRN3* | 7681 | NM_005664 | 1.598 |
| *MLF1IP* | 79682 | BC031520 | 1.897 |
| *MLF1IP* | 79682 | NM_024629 | 1.85 |
| *MMP11* | 4320 | NM_005940 | 2.642 |
| *MMP12* | 4321 | BC112301 | 1.911 |
| *MMP12* | 4321 | NM_002426 | 1.876 |
| *MMP9* | 4318 | NM_004994 | 1.755 |
| *MND1* | 84057 | BC032142 | 2.393 |
| *MND1* | 84057 | NM_032117 | 2.45 |
| *MPZ* | 4359 | NM_000530 | 1.21 |
| *MRPS17* | 51373 | NM_015969 | 1.232 |
| *MRPS17* | 51373 | BC047445 | 1.006 |
| *MSH5* | 4439 | AJ245661 | 1.169 |
| *MSTO1* | 55154 | AF111708 | 1.026 |
| *MSTO1* | 55154 | BC002535 | 1.046 |
| *MSTO1* | 55154 | NM_018116 | 1.201 |
| *MSTO1* | 55154 | CR749791 | 1.131 |
| *MTBP* | 27085 | NM_022045 | 1.395 |
| *MTBP* | 27085 | AK022122 | 1.233 |
| *MYBL2* | 4605 | NM_002466 | 3.015 |
| *MYBPC1* | 4604 | NM_002465 | 1.36 |
| *MYBPC1* | 4604 | NM_206819 | 1.051 |
| *MYO18B* | 84700 | AY077700 | 3.313 |
| *NCDN* | 23154 | NM_001014839 | 1.004 |
| *NDUFA4L2* | 56901 | NM_020142 | 1.364 |
| *NEB* | 4703 | NM_004543 | 1.469 |
| *NEIL3* | 55247 | BC025954 | 1.921 |
| *NEIL3* | 55247 | NM_018248 | 2.028 |
| *NEK2* | 4751 | BC043502 | 2.771 |
| *NEK2* | 4751 | NM_002497 | 2.412 |
| *NENF* | 29937 | NM_013349 | 1.104 |
| *NFKBIL2* | 4796 | NM_013432 | 2.109 |
| *NMB* | 4828 | NM_021077 | 1.556 |
| *NMB* | 4828 | BC008603 | 1.675 |
| *NOVA1* | 4857 | NM_006491 | 1.046 |
| *NOX4* | 50507 | BC040105 | 2.133 |
| *NOX4* | 50507 | AJ704727 | 1.64 |
| *NOX4* | 50507 | NM_016931 | 1.642 |
| *NPM1* | 4869 | NM_001037738 | 1.307 |
| *NPM1* | 4869 | BC107754 | 1.159 |
| *NPM3* | 10360 | NM_006993 | 1.009 |
| *NQO1* | 1728 | BC007659 | 2.647 |
| *NT5DC2* | 64943 | BC014550 | 1.647 |
| *NT5DC2* | 64943 | NM_022908 | 1.69 |
| *NUDCD1* | 84955 | AF521133 | 1.252 |
| *NUDT1* | 4521 | NM_198948 | 1.787 |
| *NUDT1* | 4521 | NM_198949 | 1.788 |
| *NUDT1* | 4521 | NM_002452 | 1.771 |
| *NUDT1* | 4521 | BC014618 | 1.676 |
| *NUDT1* | 4521 | NM_198953 | 1.702 |
| *NUDT1* | 4521 | BC065367 | 1.579 |
| *NUP37* | 79023 | NM_024057 | 1.056 |
| *NUSAP1* | 51203 | NM_016359 | 1.817 |
| *ORC1L* | 4998 | NM_004153 | 1.495 |
| *ORC6L* | 23594 | NM_014321 | 1.783 |
| *OSBPL3* | 26031 | NM_015550 | 1.69 |
| *OSBPL3* | 26031 | AF491785 | 1.641 |
| *OSBPL3* | 26031 | BC017731 | 1.779 |
| *OSR2* | 116039 | AK074518 | 1.735 |
| *OSR2* | 116039 | NM_053001 | 1.522 |
| *OSTbeta* | 123264 | NM_178859 | 2.281 |
| *OSTbeta* | 123264 | BC103842 | 2.587 |
| *OVOS2* | 144203 | BC039117 | 1.828 |
| *OVOS2* | 144203 | AL831947 | 1.753 |
| *PAFAH1B3* | 5050 | NM_002573 | 1.652 |
| *PAG1* | 55824 | BC090931 | 1.017 |
| *PAGE1* | 8712 | NM_003785 | 2.735 |
| *PAQR4* | 124222 | NM_152341 | 1.253 |
| *PBK* | 55872 | NM_018492 | 2.448 |
| *PCDH17* | 27253 | NM_014459 | 1.453 |
| *PDAP1* | 11333 | NM_014891 | 1.069 |
| *PDE4C* | 5143 | NM_000923 | 1.116 |
| *PDSS1* | 23590 | NM_014317 | 1.12 |
| *PDSS1* | 23590 | BC063635 | 1.142 |
| *PEA15* | 8682 | BC010469 | 1.101 |
| *PEA15* | 8682 | NM_003768 | 1.003 |
| *PHF19* | 26147 | NM_001009936 | 1.276 |
| *PITX1* | 5307 | BC003685 | 1.405 |
| *PKMYT1* | 9088 | NM_004203 | 1.814 |
| *PLA2G1B* | 5319 | BC005386 | 1.156 |
| *PLAG1* | 5324 | NM_002655 | 1.767 |
| *PLCB1* | 23236 | NM_015192 | 1.568 |
| *PLCB1* | 23236 | BC069420 | 1.049 |
| *PLCE1* | 51196 | NM_016341 | 1.637 |
| *PLCE1* | 51196 | AB040949 | 1.462 |
| *PLK1* | 5347 | NM_005030 | 1.818 |
| *PLK4* | 10733 | BC036023 | 1.325 |
| *PLVAP* | 83483 | NM_031310 | 1.632 |
| *PMCH* | 5367 | NM_002674 | 1.773 |
| *PMCHL1* | 5369 | AY008410 | 1.879 |
| *PMFBP1* | 83449 | BC028423 | 1.571 |
| *PMFBP1* | 83449 | AK093285 | 1.44 |
| *PMFBP1* | 83449 | NM_031293 | 1.443 |
| *POLQ* | 10721 | AF090919 | 1.634 |
| *POLQ* | 10721 | NM_199420 | 2.082 |
| *POLR2K* | 5440 | BC018157 | 1.113 |
| *POTE14* | 404785 | NM_207513 | 1.583 |
| *POTE14* | 404785 | NM_001005356 | 1.504 |
| *POTE15* | 339010 | NM_207355 | 1.809 |
| *POTE2* | 445582 | NM_001005364 | 1.383 |
| *POTE2* | 445582 | AY462872 | 1.699 |
| *POTE2* | 445582 | NM_001004054 | 1.515 |
| *POTE8* | 340441 | NM_001002920 | 1.658 |
| *PPFIA3* | 8541 | NM_003660 | 2.052 |
| *PRC1* | 9055 | NM_199413 | 2.078 |
| *PRC1* | 9055 | NM_199414 | 1.752 |
| *PRC1* | 9055 | NM_003981 | 1.894 |
| *PRKAA2* | 5563 | BC069680 | 1.137 |
| *PRR7* | 80758 | BC004261 | 1.637 |
| *PRR7* | 80758 | NM_030567 | 1.597 |
| *PRR7* | 80758 | BC021240 | 1.392 |
| *PSMC3IP* | 29893 | NM_016556 | 1.152 |
| *PSRC1* | 84722 | NM_001032290 | 1.295 |
| *PTRH2* | 51651 | NM_001015509 | 1.396 |
| *PTTG1* | 9232 | NM_004219 | 3.452 |
| *PTTG1* | 9232 | BC101834 | 3.331 |
| *PTTG2* | 10744 | NM_006607 | 2.75 |
| *PVT1* | 5820 | XM_933361 | 2.058 |
| *PVT1* | 5820 | XM_933342 | 1.774 |
| *PVT1* | 5820 | XM_933357 | 1.546 |
| *PVT1* | 5820 | XM_928984 | 1.46 |
| *PVT1* | 5820 | XM_933351 | 1.704 |
| *PYCRL* | 65263 | NM_023078 | 1.148 |
| *RAB3B* | 5865 | NM_002867 | 3.554 |
| *RACGAP1* | 29127 | NM_013277 | 1.792 |
| *RACGAP1* | 29127 | BC032754 | 1.948 |
| *RAD51* | 5888 | BC001459 | 1.929 |
| *RAD51* | 5888 | AK131299 | 2.035 |
| *RAD51* | 5888 | NM_002875 | 2.061 |
| *RAD51AP1* | 10635 | NM_006479 | 1.556 |
| *RAD54L* | 8438 | NM_003579 | 2.127 |
| *RASD2* | 23551 | NM_014310 | 1.421 |
| *RASL12* | 51285 | BC053734 | 1.379 |
| *RBM24* | 221662 | AK095016 | 2.045 |
| *RBM24* | 221662 | NM_153020 | 1.629 |
| *RECQL4* | 9401 | NM_004260 | 2.513 |
| *RECQL4* | 9401 | BC013277 | 1.898 |
| *REG1A* | 5967 | NM_002909 | 2.502 |
| *REG1A* | 5967 | BC005350 | 2.517 |
| *REG1B* | 5968 | NM_006507 | 3.059 |
| *RFC4* | 5984 | NM_181573 | 1.152 |
| *RHEBL1* | 121268 | NM_144593 | 1.577 |
| *RIMS2* | 9699 | NM_014677 | 2.18 |
| *RIPK2* | 8767 | BC004553 | 1.016 |
| *RIPK2* | 8767 | NM_003821 | 1.055 |
| *RNASEH2A* | 10535 | NM_006397 | 1.255 |
| *RNASEH2A* | 10535 | BC011748 | 1.311 |
| *RNF157* | 114804 | AK091467 | 1.654 |
| *RNF157* | 114804 | NM_052916 | 1.542 |
| *ROBO1* | 6091 | NM_002941 | 2.248 |
| *ROBO1* | 6091 | BC112336 | 1.536 |
| *ROD1* | 9991 | AK001685 | 1.046 |
| *RP11-146D12.2* | 441424 | XM_497044 | 1.16 |
| *RP11-19J3.3* | 401541 | BC071726 | 1.007 |
| *RP11-312O7.1* | 653573 | XM_934226 | 1.889 |
| *RP13-401N8.2* | 388358 | XM_933752 | 1.096 |
| *RP13-401N8.2* | 388358 | XM_929548 | 1.079 |
| *RPL22L1* | 200916 | BC049823 | 1.701 |
| *RPL22L1* | 200916 | BC107708 | 1.649 |
| *RPL41* | 6171 | NM_001035267 | 1.056 |
| *RPRML* | 388394 | NM_203400 | 1.072 |
| *RPS6KL1* | 83694 | BC004540 | 1.919 |
| *RPS6KL1* | 83694 | NM_031464 | 1.798 |
| *RPS6KL1* | 83694 | AK131532 | 1.587 |
| *RPS6KL1* | 83694 | AK054800 | 1.535 |
| *RRAGD* | 58528 | NM_021244 | 1.018 |
| *RRM2* | 6241 | BC001886 | 2.635 |
| *RRM2* | 6241 | NM_001034 | 1.912 |
| *RRS1* | 23212 | NM_015169 | 1.284 |
| *RYR2* | 6262 | NM_001035 | 1.926 |
| *S100P* | 6286 | NM_005980 | 2.678 |
| *S100P* | 6286 | AY423724 | 2.594 |
| *S100P* | 6286 | BC006819 | 2.542 |
| *SAC3D1* | 29901 | NM_013299 | 1.173 |
| *SAP30* | 8819 | NM_003864 | 1.238 |
| *SCN4A* | 6329 | NM_000334 | 1.382 |
| *SCNM1* | 79005 | NM_024041 | 1.125 |
| *SEMA3G* | 56920 | NM_020163 | 1.149 |
| *SERF1A* | 8293 | BC021174 | 1.02 |
| *SERPINI1* | 5274 | NM_005025 | 1.539 |
| *SFN* | 2810 | BC001550 | 3.299 |
| *SFN* | 2810 | BC000995 | 3.472 |
| *SFN* | 2810 | NM_006142 | 3.324 |
| *SFN* | 2810 | BC023552 | 3.245 |
| *SGOL1* | 151648 | NM_001012409 | 2.061 |
| *SGOL1* | 151648 | NM_001012410 | 1.784 |
| *SGOL1* | 151648 | NM_001012411 | 1.753 |
| *SGOL1* | 151648 | NM_001012412 | 1.531 |
| *SGOL2* | 151246 | AK095291 | 1.779 |
| *SGOL2* | 151246 | NM_152524 | 1.677 |
| *SGOL2* | 151246 | BC110583 | 1.141 |
| *SHCBP1* | 79801 | NM_024745 | 1.426 |
| *SIX1* | 6495 | NM_005982 | 2.147 |
| *SLAMF8* | 56833 | BC109194 | 1.587 |
| *SLC26A2* | 1836 | AF190160 | 1.536 |
| *SLC26A2* | 1836 | NM_000112 | 1.423 |
| *SLC26A6* | 65010 | NM_134263 | 2.265 |
| *SLC26A6* | 65010 | AF288410 | 2.217 |
| *SLC26A6* | 65010 | NM_022911 | 2.275 |
| *SLC26A6* | 65010 | NM_134426 | 2.078 |
| *SLC36A1* | 206358 | NM_078483 | 1.098 |
| *SLC38A6* | 145389 | NM_153811 | 1.07 |
| *SLC7A11* | 23657 | NM_014331 | 2.579 |
| *SLC7A11* | 23657 | AB040875 | 2.197 |
| *SLCO5A1* | 81796 | BC016067 | 1.263 |
| *SLD5* | 84296 | BC027454 | 1.445 |
| *SMPX* | 23676 | BC005948 | 2.457 |
| *SMPX* | 23676 | NM_014332 | 1.667 |
| *SNRPD1* | 6632 | NM_006938 | 1.104 |
| *SNRPD2* | 6633 | BC000486 | 1.103 |
| *SNRPD2* | 6633 | NM_177542 | 1.055 |
| *SNRPE* | 6635 | NM_003094 | 1.081 |
| *SNRPE* | 6635 | BC002639 | 1.142 |
| *SNRPEL1* | 414153 | XM_034623 | 1.112 |
| *SNX5* | 27131 | BC002724 | 1.062 |
| *SORT1* | 6272 | NM_002959 | 1.384 |
| *SORT1* | 6272 | BC023542 | 1.28 |
| *SPATS2* | 65244 | NM_023071 | 1.302 |
| *SPATS2* | 65244 | BC048299 | 1.179 |
| *SPBC24* | 147841 | NM_182513 | 2.868 |
| *SPBC25* | 57405 | NM_020675 | 1.229 |
| *SPINK1* | 6690 | NM_003122 | 4.22 |
| *SPINK1* | 6690 | BC025790 | 4.832 |
| *SSX1* | 6756 | NM_005635 | 3.434 |
| *SSX1* | 6756 | BC001003 | 3.669 |
| *SSX2* | 6757 | NM_003147 | 3.832 |
| *SSX2* | 6757 | NM_175698 | 3.583 |
| *SSX2* | 6757 | BC103863 | 3.819 |
| *SSX2* | 6757 | BC069313 | 3.221 |
| *SSX3* | 10214 | BC103862 | 3.694 |
| *SSX3* | 10214 | NM_175711 | 2.762 |
| *SSX3* | 10214 | NM_021014 | 2.583 |
| *SSX4* | 6759 | NM_175729 | 3.514 |
| *SSX4B* | 548313 | NM_001034832 | 3.599 |
| *SSX5* | 6758 | NM_175723 | 2.939 |
| *SSX5* | 6758 | NM_021015 | 2.652 |
| *SSX6* | 280657 | NM_173357 | 3.214 |
| *SSX7* | 280658 | NM_173358 | 3.411 |
| *SSX8* | 280659 | NM_174961 | 3.497 |
| *SSX9* | 280660 | NM_174962 | 3.369 |
| *STAMBPL1* | 57559 | NM_020799 | 1.148 |
| *STARD9* | 57519 | CR627426 | 1.456 |
| *STC2* | 8614 | NM_003714 | 2.27 |
| *STC2* | 8614 | BC000658 | 1.708 |
| *STEAP2* | 261729 | NM_152999 | 1.688 |
| *STH* | 246744 | NM_001007532 | 1.374 |
| *STIL* | 6491 | NM_003035 | 1.436 |
| *STK39* | 27347 | NM_013233 | 2.337 |
| *STMN1* | 3925 | BC014353 | 1.296 |
| *STMN1* | 3925 | NM_005563 | 1.293 |
| *STMN1* | 3925 | BC082228 | 1.471 |
| *STMN1* | 3925 | NM_203399 | 1.288 |
| *SULT1C1* | 6819 | BC005353 | 3.268 |
| *SULT1C1* | 6819 | NM_001056 | 3.499 |
| *SUZ12P* | 440423 | XM_934910 | 1.004 |
| *TACC3* | 10460 | NM_006342 | 1.689 |
| *TAF15* | 8148 | BC046099 | 1.062 |
| *TAF15* | 8148 | NM_003487 | 1.194 |
| *TAF15* | 8148 | NM_139215 | 1.127 |
| *TARBP1* | 6894 | NM_005646 | 1.076 |
| *TBX19* | 9095 | NM_005149 | 1.229 |
| *tcag7.926* | 402715 | XM_926643 | 1.033 |
| *TCEB1* | 6921 | BC093065 | 1.181 |
| *TCEB1* | 6921 | NM_005648 | 1.101 |
| *TCEB1* | 6921 | BC100028 | 1.184 |
| *TCEB1* | 6921 | BC100283 | 1.044 |
| *TCF19* | 6941 | BC002493 | 1.787 |
| *TCF19* | 6941 | BC033086 | 1.547 |
| *TDRKH* | 11022 | AK056402 | 1.645 |
| *TDRKH* | 11022 | BC022467 | 1.645 |
| *TDRKH* | 11022 | BC032690 | 1.245 |
| *TERT* | 7015 | NM_198253 | 1.921 |
| *TERT* | 7015 | NM_003219 | 2.568 |
| *TGM3* | 7053 | BC109075 | 3.764 |
| *TGM3* | 7053 | NM_003245 | 2.96 |
| *THBS4* | 7060 | NM_003248 | 3.311 |
| *TINAG* | 27283 | NM_014464 | 2.195 |
| *TK1* | 7083 | BC007986 | 1.903 |
| *TK1* | 7083 | NM_003258 | 1.665 |
| *TLCD1* | 116238 | NM_138463 | 1.238 |
| *TLE6* | 79816 | NM_024760 | 1.566 |
| *TLX1* | 3195 | NM_005521 | 2.426 |
| *TM7SF4* | 81501 | NM_030788 | 1.13 |
| *TMC7* | 79905 | BC047719 | 1.221 |
| *TMEM118* | 84900 | BC011878 | 1.517 |
| *TMEM68* | 137695 | BC020835 | 1.553 |
| *TNNC1* | 7134 | BC030244 | 1.173 |
| *TOP2A* | 7153 | NM_001067 | 2.792 |
| *TP53I3* | 9540 | NM_004881 | 1.451 |
| *TPTE2* | 93492 | NM_130785 | 1.005 |
| *TPX2* | 22974 | AF287265 | 1.661 |
| *TRAF5* | 7188 | NM_001033910 | 1.234 |
| *TRAF5* | 7188 | BC029600 | 1.18 |
| *TRAIP* | 10293 | BC019283 | 1.997 |
| *TRAIP* | 10293 | NM_005879 | 1.871 |
| *TRIM16L* | 147166 | NM_001037330 | 1.627 |
| *TRIM31* | 11074 | NM_007028 | 2.211 |
| *TRIM45* | 80263 | NM_025188 | 1.287 |
| *TRIM55* | 84675 | NM_184087 | 2.015 |
| *TRIM55* | 84675 | NM_184086 | 2.822 |
| *TRIM55* | 84675 | BC007750 | 2.709 |
| *TRIM55* | 84675 | AJ291712 | 1.086 |
| *TRIP13* | 9319 | NM_004237 | 3 |
| *TROAP* | 10024 | NM_005480 | 2.686 |
| *TROAP* | 10024 | BC011597 | 1.882 |
| *TSPAN5* | 10098 | NM_005723 | 1.232 |
| *TTC13* | 79573 | NM_024525 | 1.034 |
| *TTF2* | 8458 | NM_003594 | 1.241 |
| *TTK* | 7272 | NM_003318 | 1.534 |
| *TTK* | 7272 | BC032858 | 1.818 |
| *TUFT1* | 7286 | NM_020127 | 1.247 |
| *TXNRD1* | 7296 | NM_003330 | 1.535 |
| *UBD* | 10537 | NM_006398 | 1.884 |
| *UBD* | 10537 | BC012472 | 1.766 |
| *UBE2C* | 11065 | NM_181800 | 3.134 |
| *UBE2C* | 11065 | NM_181803 | 2.409 |
| *UBE2C* | 11065 | NM_007019 | 3.446 |
| *UBE2C* | 11065 | BC016292 | 3.478 |
| *UBE2C* | 11065 | NM_181802 | 3.553 |
| *UBE2C* | 11065 | NM_181801 | 3.471 |
| *UBE2C* | 11065 | BC007656 | 3.509 |
| *UBE2C* | 11065 | NM_181799 | 1.686 |
| *UBE2S* | 27338 | NM_014501 | 1.506 |
| *UBE2S* | 27338 | BC066948 | 1.522 |
| *UBE2T* | 29089 | BC019284 | 2.789 |
| *UBE2T* | 29089 | NM_014176 | 2.488 |
| *UCHL1* | 7345 | NM_004181 | 1.391 |
| *UCK2* | 7371 | NM_012474 | 1.172 |
| *UCK2* | 7371 | BC002906 | 1.231 |
| *UHRF1* | 29128 | NM_013282 | 2.543 |
| *UHRF1* | 29128 | BC113875 | 2.179 |
| *VCX* | 26609 | NM_013452 | 2.106 |
| *VCX* | 26609 | BC098123 | 2.093 |
| *VCX2* | 51480 | BC104168 | 1.961 |
| *VCX2* | 51480 | BC104167 | 1.703 |
| *VCX3A* | 51481 | NM_016379 | 2.371 |
| *VCX3A* | 51481 | BC098149 | 2.424 |
| *VCX3A* | 51481 | AF159128 | 2.083 |
| *VCX3A* | 51481 | BC104154 | 1.599 |
| *VCX-C* | 425054 | BC098143 | 2.115 |
| *VCY* | 9084 | NM_004679 | 1.757 |
| *VDR* | 7421 | NM_000376 | 1.337 |
| *VRK1* | 7443 | BC112075 | 1.004 |
| *VWF* | 7450 | NM_000552 | 1.013 |
| *WASF1* | 8936 | NM_001024934 | 1.048 |
| *WDR62* | 284403 | NM_173636 | 1.695 |
| *WDR62* | 284403 | BX647726 | 1.446 |
| *WDR62* | 284403 | BC058939 | 1.26 |
| *WDR67* | 93594 | AK094612 | 1.688 |
| *WDR67* | 93594 | NM_145647 | 1.652 |
| *XAGE1* | 9503 | BC009538 | 3.509 |
| *XAGE1* | 9503 | NM_133431 | 4.165 |
| *XAGE1* | 9503 | NM_020411 | 3.827 |
| *XAGE1* | 9503 | NM_133430 | 3.221 |
| *XAGE3* | 170626 | NM_133179 | 1.421 |
| *XRCC3* | 7517 | NM_005432 | 1.051 |
| *ZBTB41* | 360023 | NM_194314 | 1.081 |
| *ZFP41* | 286128 | BC034608 | 1.294 |
| *ZFP41* | 286128 | NM_173832 | 1.217 |
| *ZIC1* | 7545 | BC104848 | 1.047 |
| *ZIC2* | 7546 | NM_007129 | 4.133 |
| *ZIC5* | 85416 | NM_033132 | 1.469 |
| *ZNF101* | 94039 | BC094808 | 1.008 |
| *ZNF193* | 7746 | NM_006299 | 1.102 |
| *ZNF342* | 162979 | NM_145288 | 1.023 |
| *ZNF532* | 55205 | NM_018181 | 1.101 |
| *ZNF572* | 137209 | NM_152412 | 1.35 |
| *ZNF580* | 51157 | NM_207115 | 1.055 |
| *ZNF581* | 51545 | NM_016535 | 1.057 |
| *ZNF581* | 51545 | BC071620 | 1.008 |
| *ZNF587* | 84914 | BC011243 | 1.008 |
| *ZNF696* | 79943 | NM_030895 | 1.161 |
| *ZNF714* | 148206 | NM_182515 | 1.016 |
| *ZP3* | 7784 | M60504 | 1.964 |
| *ZP3* | 7784 | NM_007155 | 1.559 |
| *ZSCAN2* | 54993 | NM_001007072 | 1.08 |
| *ZSWIM5* | 57643 | AB040944 | 2.007 |
| *ZWINT* | 11130 | NM_001005413 | 1.668 |
| *ZWINT* | 11130 | NM_001005414 | 1.697 |
| *ZWINT* | 11130 | NM_032997 | 1.383 |
