# Supplementary figures and images for: DNA methylation and gene expression profiles show novel regulatory pathways in hepatocellular carcinoma
Source: Clin Epigenetics. 2015 Apr 14;7(1):43. doi: 10.1186/s13148-015-0077-1 (PMC4419480; doi:10.1186/s13148-015-0077-1)

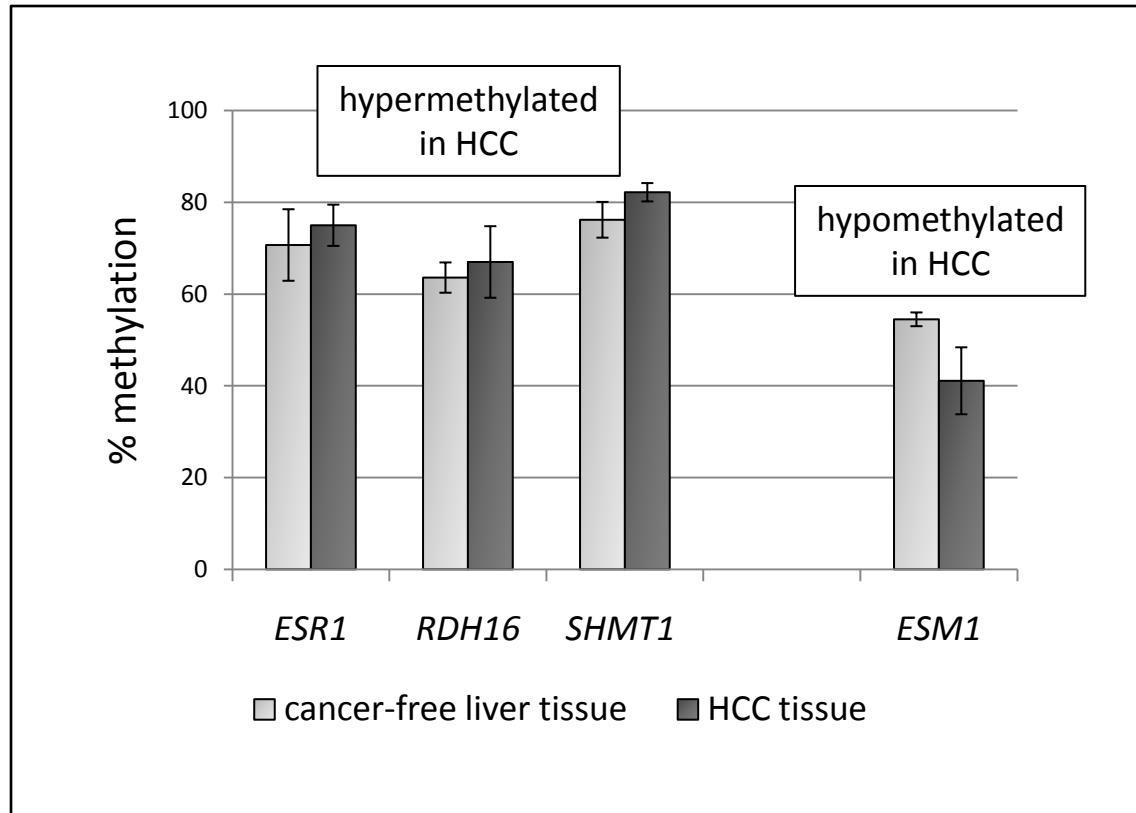

Figure S1. VALIDATION OF DNA METHYLATION RESULTS

Supplement: Additional file 5: Figure S1. — Validation of DNA methylation results: bisulfite Sanger sequencing of four differentially methylated genes in HCC and cancer-free liver tissue. [file 13148_2015_77_MOESM5_ESM.pdf]

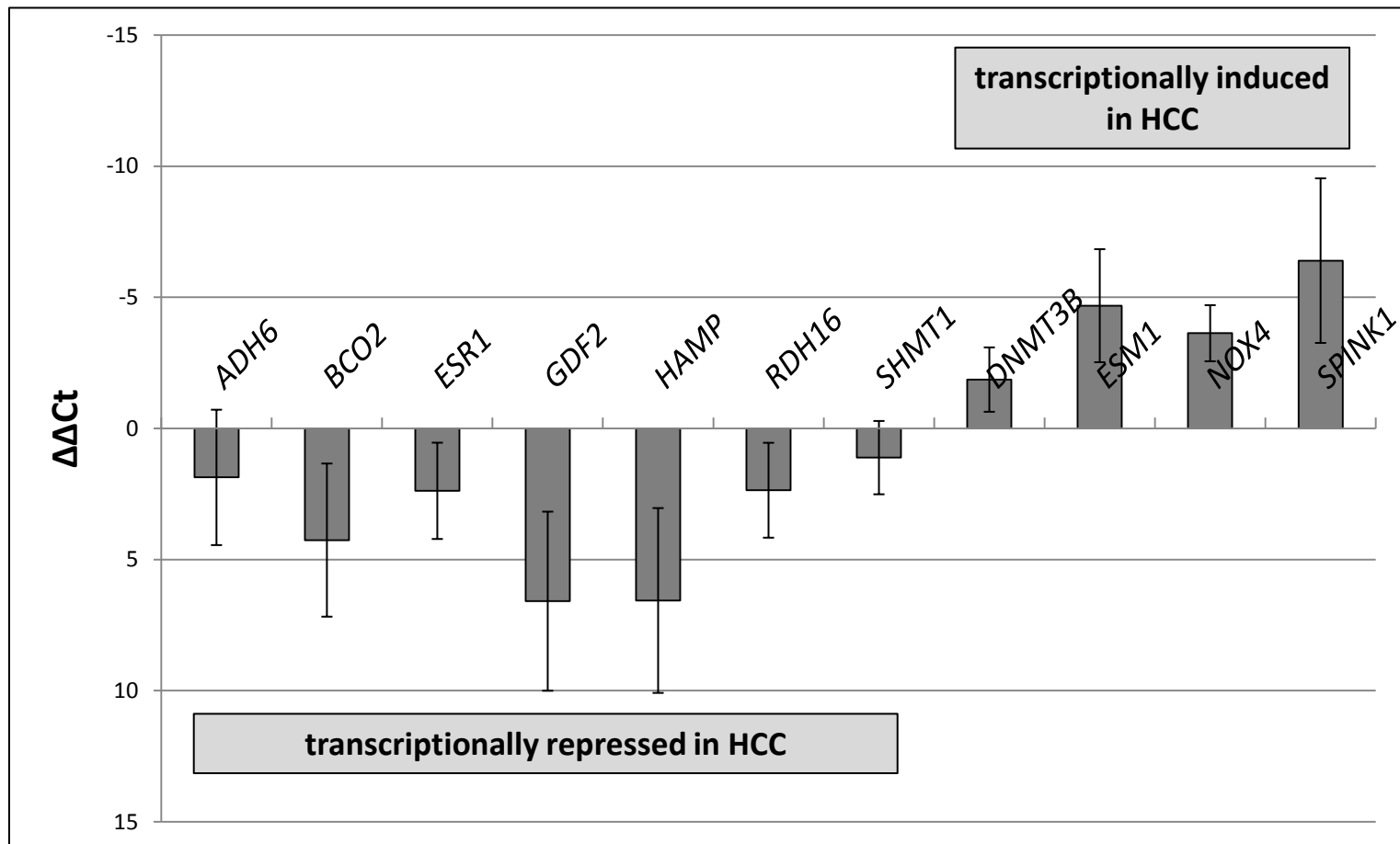

Figure S2. VALIDATION OF GENE EXPRESSION RESULTS

Supplement: Additional file 6: Figure S2. — Validation of gene expression results: RealTime RT-PCR data of seven repressed and four induced genes in HCC tissue. [file 13148_2015_77_MOESM6_ESM.pdf]
